# Measuring Burden of Unhealthy Behaviours Using a Multivariable Predictive Approach: Life Expectancy Lost in Canada Attributable to Smoking, Alcohol, Physical Inactivity, and Diet

**DOI:** 10.1371/journal.pmed.1002082

**Published:** 2016-08-16

**Authors:** Douglas G. Manuel, Richard Perez, Claudia Sanmartin, Monica Taljaard, Deirdre Hennessy, Kumanan Wilson, Peter Tanuseputro, Heather Manson, Carol Bennett, Meltem Tuna, Stacey Fisher, Laura C. Rosella

**Affiliations:** 1 Ottawa Hospital Research Institute, Ottawa, Ontario, Canada; 2 Institute for Clinical Evaluative Sciences, Ottawa and Toronto, Ontario, Canada; 3 Statistics Canada, Ottawa, Ontario, Canada; 4 Department of Family Medicine, University of Ottawa, Ottawa, Ontario, Canada; 5 School of Epidemiology, Public Health and Preventive Medicine, University of Ottawa, Ottawa, Ontario, Canada; 6 Bruyère Research Institute, Ottawa, Ontario, Canada; 7 Public Health Ontario, Toronto, Ontario, Canada; 8 University of Toronto, Toronto, Ontario, Canada; Stanford University, UNITED STATES

## Abstract

**Background:**

Behaviours such as smoking, poor diet, physical inactivity, and unhealthy alcohol consumption are leading risk factors for death. We assessed the Canadian burden attributable to these behaviours by developing, validating, and applying a multivariable predictive model for risk of all-cause death.

**Methods:**

A predictive algorithm for 5 y risk of death—the Mortality Population Risk Tool (MPoRT)—was developed and validated using the 2001 to 2008 Canadian Community Health Surveys. There were approximately 1 million person-years of follow-up and 9,900 deaths in the development and validation datasets. After validation, MPoRT was used to predict future mortality and estimate the burden of smoking, alcohol, physical inactivity, and poor diet in the presence of sociodemographic and other risk factors using the 2010 national survey (approximately 90,000 respondents). Canadian period life tables were generated using predicted risk of death from MPoRT. The burden of behavioural risk factors attributable to life expectancy was estimated using hazard ratios from the MPoRT risk model.

**Findings:**

The MPoRT 5 y mortality risk algorithms were discriminating (C-statistic: males 0.874 [95% CI: 0.867–0.881]; females 0.875 [0.868–0.882]) and well calibrated in all 58 predefined subgroups. Discrimination was maintained or improved in the validation cohorts. For the 2010 Canadian population, unhealthy behaviour attributable life expectancy lost was 6.0 years for both men and women (for men 95% CI: 5.8 to 6.3 for women 5.8 to 6.2). The Canadian life expectancy associated with health behaviour recommendations was 17.9 years (95% CI: 17.7 to 18.1) greater for people with the most favourable risk profile compared to those with the least favourable risk profile (88.2 years versus 70.3 years). Smoking, by itself, was associated with 32% to 39% of the difference in life expectancy across social groups (by education achieved or neighbourhood deprivation).

**Conclusions:**

Multivariable predictive algorithms such as MPoRT can be used to assess health burdens for sociodemographic groups or for small changes in population exposure to risks, thereby addressing some limitations of more commonly used measurement approaches. Unhealthy behaviours have a substantial collective burden on the life expectancy of the Canadian population.

## Abstract 2

To address limitations of commonly used burden-of-disease measurement approaches, Douglas G. Manuel and colleagues develop, validate, and apply a multivariable predictive model for all-cause death attributable to unhealthy behaviors.

## Author Summary

**Why Was This Study Done?:**

- The burden of unhealthy behaviours and other risk factors is commonly calculated using aggregate, indirectly linked data. Amongst other limitations, there are concerns that these data are poorly suited for assessing equity.
- Increasingly, population health surveys are being linked, at the individual level, to health outcomes. We examined the role of assessing the burden of unhealthy behaviours using individual, as opposed to aggregate, data.

**What Did the Researchers Do and Find?:**

- We generated a multivariable predictive algorithm of health behaviours and mortality (MPoRT) using the Ontario sample of the linked Canadian Community Health Survey (CCHS). There were approximately 1 million person-years of follow-up and 9,900 deaths in the development and validation datasets.
- Next, the burden of unhealthy behaviours was estimated by applying the MPoRT risk tool to the most current version of the CCHS.
- For the 2010 Canadian population, 6.0 y of life expectancy lost were attributable to unhealthy behaviours
- Smoking, by itself, contributed 32% to 39% of the difference in life expectancy across social groups.
- The Canadian life expectancy for people adhering to health behaviour recommendations was 17.9 y greater than for people with the least favourable risk profile.

**What Do These Findings Mean?:**

- Unhealthy behaviours contribute to a large burden of reduced life expectancy.
- Linked population health surveys create an opportunity to estimate burden of disease using individual-based data, as in our multivariable predictive approach, and to supplement existing aggregate approaches.
- Multivariable risk algorithms can be validated and calibrated for potential application in over 100 countries, potentially allowing widespread use of multivariable approaches.
- Multivariable risk algorithms are more complex than alternative approaches to measure burden from health behaviours, but their use can be facilitated by reporting the algorithm in machine-readable format (https://github.com/Ottawa-mHealth/predictive-algorithms) and/or by providing online calculators (https://www.projectbiglife.ca).

## Introduction

Unhealthy behaviours, including smoking, poor diet, physical inactivity, and unhealthy alcohol consumption, are leading risk factors for premature mortality worldwide [1–3]. Measuring the burden of health-behaviour–related deaths in populations is challenging because standard death certificates do not provide information about underlying risks factors for disease. Previous population burden studies have addressed this challenge using two methods. The most commonly used method—*aggregated data approach*, used in the Global Burden of Disease study and first described by Levin—starts with disease-specific mortality and indirectly attributes underlying risks to a fraction of deaths according to separately measured estimates of the association between the exposures and disease [2,4,5]. The second method, the *population cohort approach*, starts with population-based health surveys that individually ascertain exposures to different health behaviours [6,7]. Respondents are followed until death, with attribution of unhealthy behaviours estimated directly from a multivariable regression model.

We propose a method to estimate mortality attributable to unhealthy behaviour using risk algorithms that directly incorporate a variety of baseline characteristics and risk factors. We call this a baseline risk or *multivariable predictive approach* [8,9]. This approach combines aspects of aggregated data and population cohort approaches but has advantages that we describe briefly here and further in the Discussion. All three approaches use population-based health surveys to estimate population exposure to various behaviours. Such surveys are now being performed in over 100 countries, but only a few countries link respondents to death certificates; without this linkage, the cohort method—which requires respondent follow-up—cannot be used. In the multivariable predictive approach, validated risk algorithms can be applied to *unlinked* exposure data from population health surveys. This has the potential to provide mortality risk estimates for survey respondents in many different countries.

The multivariable predictive approach uses individual-level data to address several challenges with the current Global Burden of Disease study. Most importantly, the Global Burden of Disease study has acknowledged challenges in examining burden from an equity perspective, such as by socioeconomic position, and the authors have suggested that “capacity and methods to undertake this type of analysis need to be created or strengthened” [10]. Other challenges include estimating the burden from incremental changes in risk factors and adjusting for the joint distribution of risk factors and their interactions [10]. Furthermore, multivariable risk algorithms are characterized as the most discriminating and accurate approach to estimating baseline risk [11–13]. Baseline risk assessment has been a cornerstone of health planning in both population and clinical settings for over 30 y. In the words of Geoffrey Rose, “all policy decisions should be based on absolute (baseline) measures of risk.”

The example of burden attributable to smoking in Canada is illustrative of how the multivariable predictive approach can improve policy development and evaluation. Twenty years ago, policy action for smoking prevention was galvanized when smoking-attributable burden was estimated at 40,000 to 45,000 deaths annually [14]. Unfortunately, an equity assessment of smoking burden was not performed; the resulting inequitable uptake of smoking prevention and cessation strategies widened the socioeconomic gap in avoidable deaths [15]. Furthermore, smoking is more heavily concentrated in specific groups, including people with low socioeconomic position, who have additional health behaviour risks and/or comorbid conditions. Assuming all smokers of a particular age have the same risk of death and distribution of risk factors—an assumption of the aggregated data approach—results in poor risk discrimination and underestimation of the burden of smoking, particularly for people with low socioeconomic position [14]. Lastly, smoking consumption has been changing: in Canada there are fewer heavy smokers and more former smokers who have an earlier age of smoking cessation (longer time since quitting). Measuring the reduced burden of smoking from these incremental changes in smoking consumption is best calculated when the measurement of smoking exposure and hazards reflects changing smoking consumption. Typically, the aggregated data approach categorizes smoking burden into current, former, or never smoker, whereas the multivariable predictive approach is well suited to assess individual-level exposure using a wider range of measures, such as heavy and light smokers and recent and remote time since cessation.

We sought to estimate the mortality burden attributable to four behavioural risk factors in Canada (smoking, unhealthy alcohol consumption, physical inactivity, and poor diet) using a multivariable predictive approach. The study had two objectives: (1) to develop and validate a 5 y all-cause mortality risk prediction algorithm for a general population (Mortality Population Risk Tool, or MPoRT) using a population health survey, and (2) to apply MPoRT to a recent Canadian population health survey to estimate the life expectancy lost due to unhealthy behaviours. We also calculate life expectancy for people who smoke and smoking-attributable mortality by socioeconomic position to illustrate the model’s ability to examine health burden from an equity perspective.

## Methods

This study was approved by the Ottawa Health Science Network Research Ethics Board (formerly the Ottawa Hospital Research Ethics Board). Data were accessed at the Institute for Clinical Evaluative Sciences (Ontario data) and at Statistics Canada (national data).

The statistical plan was generated based on a previous study [16]. All exposures and outcomes were prespecified based on consultation taking into consideration both science and policy perspectives. In addition, policy actors requested that the model be assessed for predictive accuracy for a range of predetermined sociodemographic groups (see following sections). Analyses that were added based on reviewers’ comments were the calculation of smoking-attributable outcomes across sociodemographic groups. As well, we revised statistical confidence intervals to consider the combined error from health behaviour exposure ascertainment, MPoRT risk estimation, and estimation of life expectancy using period life tables.

### Study Data

Exposure data for this study were from the Canadian Community Health Survey (CCHS) cycles 1.1 (conducted in 2000–2001), 2.1 (2003–2004), 3.1 (2005–2006), 4.1 (2007–2008), and 5.1 (2009–2010) [17]. These surveys were used for three different purposes:

1. Derivation of the MPoRT algorithm—development of the algorithm with a focus on the hazard of death from unhealthy behaviours, adjusted for sociodemographic risk factors.
2. Validation of the MPoRT algorithm—external validation of the algorithm using data that are separate or distinct from the derivation data. Validation included assessment of the algorithm in a wide range of predefined subgroups based on sociodemographic and health behaviour characteristics.
3. Application of the MPoRT algorithm—a recent CCHS national data (2009–2010) was used to estimate life expectancy lost due to unhealthy behaviours. Risk of death for each survey respondent (whose data have not been linked to death certificates) was estimated using MPoRT.

The CCHS surveys represented 98% of the Canadian population over 12 y of age and attained an average response rate of 79.2%. The surveys were conducted through interviews by telephone and in person, and all responses were self-reported. Each Health Region had approximately 2,000–3,000 respondents per survey (regardless of the size of the underlying population of the region) to ensure sufficient power to provide Region-specific estimates. Excluded from the sampling frame were people living on First Nation Reserves and Crown Lands, institutional residents, and full-time members of the Canadian Forces. Households were selected through stratified multilevel cluster sampling of residences using local planning regions as the primary sampling unit. Selection of respondents from households depended on the household composition and was intended to increase the representation of the two age groups of special interest: youths and seniors. The details of the survey methodology have been previously published [17].

The study (derivation, validation, and application data) was limited to respondents between the ages of 20 and 99 y at the time of survey. The derivation and Ontario validation cohorts only included those respondents who had agreed to have their survey linked to health administrative databases (derivation cohort = 80.2%). Respondents were included once; if respondents participated in more than one survey cycle, the earliest record was retained. Respondents were also excluded from the cohort if, at the time of the survey, they were not eligible for the provincial health insurance program or they were pregnant (see also **S1 Fig** for study flow).

### Derivation Cohort

Three cycles of the linked CCHS Ontario subsample (2003–2004, 2005–2006, and 2007–2008) were combined to create a derivation cohort of respondents who were followed until death, loss to follow-up, or March 31, 2013, whichever was earlier. All consenting respondents were linked to the Registered Persons Database to ascertain deaths (>99.5% linkage rate to death certificates)—see https://datadictionary.ices.on.ca for details.

### Validation Cohorts

There were two validation cohorts, each with 5 y of follow-up. The first validation cohort was composed of Ontario respondents of the earliest linked CCHS survey (2000–2001). The second validation cohort, which became available late in the study (after MPoRT model development), was a preliminary linked *national* sample of 2003–2004 CCHS respondents, excluding Ontario respondents as they formed part of the derivation cohort. All national CCHS respondents were linked to death certificates using the Canadian Mortality Database (preliminary linkage: 0.04% false positives and 3.46% false negatives).

### Risk Factors for Death

We included the following pre-specified risk factors in the MPoRT algorithm: age, sex, four health behaviours (smoking, alcohol consumption, physical activity, and diet; see **Table 1**), sociodemographic factors (ethnicity, immigration status, and education), chronic conditions (self-report of physician-diagnosed diabetes, coronary heart disease, stroke, and cancer), and body mass index. We also included area-based measures: neighbourhood deprivation, local planning region, and rurality.

**Table 1:** Definitions of risk factors.^a^

Risk Factor	Definition
Smoking	
Heavy smoker	Current smoker (≥1 pack/day)
Light smoker	Current smoker (<1 pack/day)
Former heavy smoker	Former smoker (≥1 pack/day)
Former light smoker	Former smoker (<1 pack/day)
*Non-smoker*	*Never-smoker or former occasional smoker with <100 lifetime cigarettes*
Alcohol	
Heavy drinker	>21 (men) or >14 (women) drinks in the previous week, ≥5 drinks on any day in the previous week, or bingeing^b^ behaviour on a weekly basis
Moderate drinker	4 to 21 (men) or 3 to 14 (women) drinks/week
*Light or non-drinker*	*0 to 3 (men) or 0 to 2 (women) drinks/week*
Physical activity	Daily averaged MET derived from previous month’s self-reported history of leisure time physical activity
Diet score^c^	
Fruit and vegetable intake	1 point per daily frequency of fruit and vegetable consumption, excluding fruit juice (maximum 8 points)
High potato intake^d^	-2 points
No carrot intake	-2 points
High fruit juice intake	-2 points per daily frequency of fruit juice consumption greater than once/day (maximum -10 points)

Abbreviations: MET = metabolic equivalent of task, a measure of calories burned by type, duration, and frequency of physical activity.

^a^Reference group is in italics.

^b^Bingeing was defined as ≥5 drinks on any occasion.

^c^Diet score = 2 baseline points + summation of total points for diet attributes (negative overall scores are recoded to 0, resulting in a range from 0 to 10)

^d^≥7 (men) or ≥5 (women) times/week.

We examined health behaviours as both categorical and continuous measures of exposure. Continuous measures were assessed and preferentially included for two reasons: for improved predictive performance (see below) [13,18,19], and to allow counterfactual examination of small changes in population exposure [12,13].

*Smoking* behaviour was described by combining separate questions about smoking status, daily cigarette consumption, and past smoking behaviour. We categorized current smokers as heavy or light smokers (see **Table 1)**. Former smokers were also dichotomized as heavy or light with a continuous measure of time since quitting (see **S2 Fig**). *Alcohol* drinking behaviour was specified as heavy, moderate, and light/non using cut-points for daily alcohol consumption and the presence of bingeing behaviour (see **Table 1**). *Physical activity* was included as a continuous measure (see **S3 Fig**) using average metabolic equivalent of task (MET) per day derived from an aggregate list of leisure-time physical activities (frequency and duration) that were examined in each survey.

*Diet* was included using an a priori approach that considered the possibility that different dietary components could be either protective (fruit and vegetable and carrot consumption) or harmful (high potato or fruit juice consumption) following dietary recommendations and prior epidemiology studies [20–23]. The four CCHS dietary variables for weekly food intake were combined into an index (the Perez Diet Score) based on the individual relationship with mortality observed in previous studies using the CCHS linked data [8,24]. The index score varied between 0 and 10, with points added for each frequency and serving of fruit or vegetables, and points deducted for high potato consumption, no carrot consumption, or excessive juice consumption (see **Table 1**).

*Neighbourhood deprivation* was developed using the Deprivation Index originally published by Pampalon and Raymond [25]. The index, intended to serve as a proxy for individual-level measures, categorizes the smallest geo-statistical units of the Canadian census (dissemination areas) into two sets of quintile groups: one for the material components of deprivation (based on average income, percent without high school graduation, and the employment ratio) and the other for the social components (percent of single-parent families; percent of people living alone; and percent of people divorced, widowed, or separated) [26]. In each quintile group, Q1 represents the 20% least deprived and Q5 represents the 20% most deprived. These quintiles are cross-tabulated to create 25 distinct cells. Dissemination areas with material and social combinations in the first and second quintiles (4 cells) were categorized as having low neighbourhood deprivation. Dissemination areas with material and social combinations in the fourth and fifth quintiles (4 cells) were categorized as having high neighbourhood deprivation. All other dissemination areas were categorized as having moderate neighbourhood deprivation.

### Development of the MPoRT Algorithm

We used a Cox proportional hazards model to analyze time to death with 5 y risk of death as the outcome of interest. We then converted the proportional hazards model to generate the MPoRT risk algorithm, using the baseline risk and beta coefficients directly from the model. We sought to develop a predictive algorithm that was both well calibrated and discriminating, with an emphasis on calibration for behavioural risk factors and use in the community setting [27]. Calibration reflects an algorithm’s ability to produce predictive estimates that closely approximate observed risk [28].

We included age as a continuous time-dependent variable to account for potential violations of the proportional hazards assumption and for the non-linear increase of death hazard in older ages. Multicollinearity was assessed using the approach described by Sarle and Hoeffding [29,30]. We derived separate models for males and females.

Age and health behaviours were the primary predictors of interest and formed the base model. We modeled mortality as a function of age using spline functions. Additional sociodemographic, intermediate (body mass index), and proximal (chronic diseases) risk factors were added to improve calibration [31]. We predefined explicit criteria for choosing these risk factors and added them only if they met the criteria. First, we identified important subgroups and target populations through a structured consultation process with policy actors. This process considered all behavioural risk exposures, age groups, health planning regions, sociodemographic groups, body mass index, and chronic disease status. Next, the policy actors identified a 20% difference between the predicted and observed risk as “clinically important” for policy and planning. We therefore added a risk factor if, in the absence of that factor, the model had a greater than 20% difference between the predicted and observed risk. We only assessed subgroups that represented more than 5% of total deaths. To determine the model’s discrimination—its ability to differentiate individuals at high risk from those at low risk [28]—we used the C-statistic and 90:10 risk percentile ratios for survival data with time-dependent covariates [32].

We examined interactions between age and behavioural risk factors. Only respondents with complete records were included in analyses because missing values were infrequent (less than 5% for each of the variables under consideration) and we intended to apply the algorithm to similar population health data with infrequent missing data [33]. Further details of the exposure variables are provided in **S1 Table**.

### Validation of Predictive Accuracy and Assessment of Risk Hazards

This study applied MPoRT in two ways: to estimate the baseline risk of mortality and to estimate the attribution of health behaviours to mortality (life expectancy lost). To assess the predictive accuracy of baseline risk we examined the C-statistic and measures of calibration (observed versus predicted risk estimates in the two validation cohorts). Attribution of health behaviours was calculated by combining baseline risk with hazard ratios of the behavioural risk factors. We assessed the health behaviour hazard ratios within the development data as well as the national sample of CCHS (which included all provinces) by comparing the full model (age, health behaviours, sociodemographic indicators, and chronic conditions) to two alternative models: (1) age and health behaviours only, and (2) full model without the first 2 y of follow-up to allow for potential healthy respondent effect.

### Calculating Mortality Burden of Health Behaviour Risk in Canada 2009–2010

Mortality burden from unhealthy behaviours was defined as the difference between the *baseline* mortality risk (i.e., the mortality risk of the population based on current exposure patterns) and the *healthy reference* mortality risk. Baseline mortality risk was defined as mortality risk calculated using MPoRT for each CCHS respondent based on their reported health behaviours and other predictive risks. Healthy reference mortality risk was defined as the MPoRT-calculated mortality risk assuming respondents had an exposure that was at the reference or healthy level. For example, to calculate smoking-deleted mortality, we assumed all respondents, including current and former smokers, were non-smokers. The mortality burden of smoking was the difference between the baseline (actual) risk and the new healthy reference exposure. The procedure was repeated for physical activity (all respondents with less than 3 METs/day of leisure-time physical activity were recoded to have 3 METs/day, corresponding to recommendations in the Canadian physical activity guidelines [34]), diet (all respondents with a diet score of less than 8 were recoded to have a diet score of 8, corresponding to recommendations in Canada’s Food Guide [35]), and alcohol (heavy drinkers were recoded to light/non-drinker). The combined burden of the four unhealthy behaviours was estimated by assuming all respondents were in the healthy reference category for all four behaviours.

Prior to performing burden estimates, we calibrated the MPoRT algorithm by comparing the predicted 1 y risk of death for the Canadian CCHS 2009–2010 respondents to Canadian observed mortality rates (average 2010 and 2011), by sex and age [36]. The difference in the observed and predicted risk was used as an external unlinked calibration coefficient α^euc^_(age, sex)_ that was applied to the risk α from the MPoRT algorithm where α^euc^_(age, sex)_ is α x O_(age, sex)_/P_(age, sex)._ We used survey weights provided by Statistics Canada to account for survey design.

Confidence intervals and standard error for predicted mortality were calculated using the bootstrap approach of Kovacevic et al. for out-of-sample prediction with population health surveys [37]. These confidence intervals combined two sources of uncertainty: MPoRT model parameters and exposure variability (CCHS 2012). Confidence intervals for life expectancy were calculated using the approach of Chiang and the variances estimates for mortality, as described above [38]. See **S1 Text** for more details.

### Calculating Life Expectancy Lost from Unhealthy Behaviours in Canada 2009–2010

We calculated life expectancy lost from unhealthy behaviours using a cause-deleted period life table approach [39,40]. Similar to the approach for mortality burden, life expectancy lost was the difference between *baseline* life expectancy and *healthy reference* life expectancy.

National statistical agencies and the World Health Organization calculate life expectancy by generating period life tables using age- and sex-specific mortality rates for a specific period (e.g., 2009–2010), which are converted to age- and sex-specific mortality risk. This approach assumes a stationary population, meaning mortality rates for a specific period are applied over the entire life time (the stationary population). Burden of disease studies typically use these same age- and sex-specific life tables but delete risk-factor–attributable deaths. Life expectancy is then re-estimated with these cause-deleted mortality rates to generate a cause-deleted life expectancy.

We used the same approach as a typical period life table, but we used MPoRT-predicted mortality risk (i.e., multivariable predicted risk) for each CCHS respondent, instead of starting with observed age- and sex-specific mortality rates. Following, we generated weighted sex-specific 5 y abridged period life tables (20 to 99 y of age) [38]. These life tables were used to generate baseline life expectancy, which corresponds to baseline mortality risk (described above). Similarly, healthy reference life expectancy was generated using life tables with healthy reference mortality risk, as described in the previous section.

After calculating mortality risk for each CCHS respondent, it was straightforward to generate life tables for a wide range of health profiles (e.g., by smoking status or socioeconomic position) by aggregating MPoRT mortality risk and the corresponding profile.

### Calculating Life Expectancy for Different Health Behaviour Profiles in Canada 2009–2010

Canadian life expectancies were calculated for healthy and unhealthy risk profiles using the 2009–2010 CCHS. The healthy profile was defined as non-smoking; moderate, light, or non-drinking (men: 0 to 21 drinks/week; women: 0 to 14 drinks/week); active (≥3 METs/day); and consuming a high-quality diet (diet score ≥8). The unhealthy profile was defined as heavy smoking; heavy drinking (men: >21 drinks/week or weekly binge drinking; women: >14 drinks/week or weekly binge drinking); inactive (<1.5 METs/day); and with poor diet quality (diet score <2).

## Results

The final derivation cohort had 77,399 respondents (0.2% were lost to follow-up). Within the 598,913 person-years of follow-up (median time: 7.6 y), 6,142 deaths were observed, of which 2,953 occurred in males and 3,189 in females. The Ontario validation cohort had 24,729 respondents (127,403 person-years of follow-up; 1,383 deaths) and the Canadian validation cohort had 56,215 respondents (275,468 person-years of follow-up; 2,375 deaths). The application cohort of 89,984 respondents represented 25.3 million Canadians after applying survey weights.

Details of the characteristics of the derivation, validation, and application cohorts are presented in **Table 2** and **Table 3**. Generally, health behaviours improved slowly over time. For example, there is a reduction in the prevalence of heavy smokers and of high potato and fruit juice consumption. Similarly, there was an improvement in socioeconomic status with an increase in post-secondary education. **Table 4** shows the crude and age-standardized mortality rates for health behaviour risks (see **S2 Table** to **S4 Table** for crude and age-standardized mortality rates by other exposure variables). Heavy smokers had the highest age-standardized mortality rate per 10,000 person-years: 196.8 (95% confidence interval [CI]: 161.8, 237.1) for males and 193.8 (95% CI: 162.8, 229.0) for females. The most favourable health behaviour exposures had the lowest mortality rates for all exposures (range per 10,000 person-years: for men, 67.4 to 73.9 and for women, 60.3 to 69.9).

**Table 2:** Baseline characteristics of male study cohorts.^a^

Characteristics	Ontario Derivation	Ontario Validation	Non-Ontario Validation	Canada Application
	*n* = 35,507	*n* = 11,138	*n* = 25,577	*n* = 40,672 (12,400,000^b^)
Age^c^	48.0 (36.0–62.0)	46.0 (36.0–60.0)	48.0 (35.0–61.0)	46.0 (33.0–59.0)
**Health behaviours**				
Smoking status				
Heavy smoker	10.5	14.0	11.4	8.2
Light smoker	15.9	15.9	15.7	16.4
Former heavy smoker	19.0	18.7	21.7	14.7
Former light smoker	16.9	15.9	16.4	16.0
Non-smoker	37.8	35.5	34.8	44.7
Physical activity^c^	1.6 (0.6–3.1)	1.4 (0.5–3.0)	1.5 (0.6–3.0)	1.6 (0.6–3.2)
Diet				
Fruit and vegetable intake^c^	3.2 (2.2–4.5)	3.0 (2.0–4.2)	3.0 (2.0–4.30)	3.3 (2.2–4.8)
High potato intake	14.5	18.8	21.2	10.9
No carrot intake	12.9	12.5	9.2	10.9
High fruit juice intake	16.5	16.9	16.5	15.6
Alcohol consumption				
Heavy drinker	21.1	19.7	19.8	20.3
Moderate drinker	23.7	23.3	21.1	22.3
Light drinker	55.1	57.0	59.1	57.4
**Sociodemographic**				
Neighbourhood deprivation				
High	16.0	15.8	19.3	15.5
Moderate	63.7	63.3	71.1	66.9
Low	20.3	20.9	9.7	17.6
Education				
< High school	17.9	22.2	24.6	13.8
High school graduate	24.5	27.3	23.5	23.3
Post-secondary graduate	57.6	50.5	51.9	62.9
Years since immigration				
0 to 15	5.2	5.4	3.1	9.4
16 to 30	4.3	4.1	2.2	6.7
31 to 45	5.7	6.0	2.4	5.0
>45 or born in Canada	84.7	84.4	92.4	78.9
**Comorbidities**				
Heart disease	8.7	7.9	7.6	6.1
Stroke	1.6	1.3	1.4	1.0
Cancer	2.8	2.6	2.0	2.2
Diabetes	8.0	6.5	6.8	8.0
Body mass index ≥35	4.8	4.1	4.3	4.7

^a^Numbers are percentages unless otherwise indicated

^b^Represented population—estimated using the Canadian Community Health Survey sampling weights

^c^Median (IQR)

**Table 3:** Baseline characteristics of female study cohorts.^a^

Characteristics	Ontario Derivation	Ontario Validation	Non-Ontario Validation	Canada Application
	*n* = 41,892	*n* = 13,591	*n* = 30,638	*n* = 49,312 (12,900,000^b^)
Age^c^	51.0 (36.0–65.0)	47.0 (36.0–64.0)	50.0 (36.0–65.0)	47.0 (34.0–60.0)
**Health behaviours**				
Smoking status				
Heavy smoker	5.7	8.3	6.4	4.0
Light smoker	16.2	17.3	17.2	14.7
Former heavy smoker	9.1	9.2	11.3	7.6
Former light smoker	17.7	16.1	18.0	17.6
Non-smoker	51.3	49.1	47.2	56.1
Physical activity^c^	1.4 (0.5–2.7)	1.2 (0.4–2.5)	1.3 (0.5–2.6)	1.3 (0.5–2.7)
Diet				
Fruit and vegetable intake^c^	4.1 (2.8–5.6)	3.6 (2.6–5.0)	3.9 (2.7–5.5)	4.2 (2.9–6.0)
High potato intake	12.0	16.7	18.2	9.6
No carrot intake	9.7	9.4	6.5	8.6
High fruit juice intake	13.9	16.7	15.7	11.6
Alcohol consumption				
Heavy drinker	6.8	6.0	5.9	7.3
Moderate drinker	20.9	18.5	18.6	20.3
Light drinker	72.3	75.5	75.5	72.3
**Sociodemographic**				
Neighbourhood deprivation				
High	17.1	17.0	20.1	15.7
Moderate	63.7	63.9	71.1	67.3
Low	19.3	19.1	8.8	17.0
Education				
< High school	19.0	24.0	25.6	14.2
High school graduate	26.2	29.5	24.9	24.2
Post-secondary graduate	54.8	46.5	49.5	61.6
Years since immigration				
0 to 15	5.0	4.9	2.9	9.9
16 to 30	4.0	4.4	2.1	6.2
31 to 45	5.4	5.6	2.1	4.7
>45 or born in Canada	85.5	85.2	92.9	79.2
**Comorbidities**				
Heart disease	7.3	7.5	6.9	4.4
Stroke	1.5	1.5	1.4	1.1
Cancer	2.6	2.8	2.3	2.0
Diabetes	6.9	5.3	6.1	5.8
Body mass index ≥35	6.3	5.7	5.6	5.6

^a^Numbers are percentages unless otherwise indicated

^b^Represented population—estimated using the Canadian Community Health Survey sampling weights

^**c**^ **Median (IQR)**

**Table 4:** Crude and age-standardized death rates per 10,000 person-years for health behaviour groups.

	Males				Females			
	Person-years	Deaths	Crude rate	Age standardised rate (95% CI)	Person-years	Deaths	Crude rate	Age standardised rate (95% CI)
Total	285,035	3,766	132.1	96.05 (92.88, 99.30)	340,532	3,978	116.8	89.7 (86.7, 92.7)
Age Group								
20–29	39,267	27	6.9	-	45,530	20	4.4	-
30–39	55,448	59	10.6	-	59,597	54	9.1	-
40–49	55,584	152	27.4	-	56,166	128	22.8	-
50–59	53,168	390	73.4	-	64,289	364	56.6	-
60–69	44,074	802	182.0	-	53,446	645	120.7	-
70–79	28,925	1352	467.4	-	42,358	1,279	302.0	-
80–99	8,569	984	1148.4	-	19,146	1,488	777.2	-
Smoking Status								
Non-smoker	107,578	814	75.7	67.4 (62.8, 72.3)	173,216	1822	105.2	69.5 (66.2, 73.0)
Light	44,834	442	98.6	165.5 (137.4, 197.6)	54,855	515	93.9	141.3 (126.0, 158.0)
Former light (quit <20 y)	24,709	265	107.3	126.7 (99.5, 159.0)	34,468	322	93.4	107.9 (95.1, 122.0)
Former light (quit ≥20 y)	21,865	521	238.3	83.5 (56.6, 118.9)	24,291	411	169.2	75.7 (67.5, 84.6)
Heavy	29,975	414	138.1	196.8 (161.8, 237.1)	19,582	286	146.1	193.8 (162.7, 229.0)
Former heavy (quit <20 y)	29,691	525	176.8	120.4 (108.0, 133.8)	20,479	328	160.2	149.5 (122.6, 180.4)
Former heavy (quit ≥20 y)	23,138	655	283.1	90.0 (68.9, 115.5)	10,098	187	185.2	79.9 (67.7, 93.8)
Missing	3,245	130	400.6	106.6 (85.4, 131.5)	3,543	107	302.0	115.4 (92.1, 142.8)
Physical Activity (METs/day)								
0 to <1	93,866	1643	175.0	119.4 (113.5, 125.5)	129,514	2328	179.8	109.1 (104.4, 113.9)
1 to <2	63,688	680	106.8	80.7 (74.5, 87.2)	80,182	718	89.6	73.4 (67.7, 79.5)
2 to <3	44,137	489	110.8	79.1 (71.7, 87.0)	54,315	365	66.2	65.01 (56.37, 74.61)
≥3	77,713	617	79.4	67.7 (61.9, 73.8)	74,160	376	50.7	60.3 (52.1, 69.5)
Missing	5,631	337	598.5	206.9 (174.8, 243.2)	2,361	191	808.9	200.8 (168.3, 237.7)
Diet Score (Range: 0 to 10)								
0 to <2.5	45,731	523	114.4	119.9 (109.8, 130.7)	31,845	420	131.9	122.0 (110.4, 134.5)
2.5 to <5	119,640	1499	125.3	97.4 (92.3, 102.7)	103,174	1369	132.7	100.5 (95.0, 106.3)
5 to <7.5	84,519	1023	121.0	80.3 (75.1, 85.8)	128,211	1351	105.4	78.1 (73.7, 82.8)
7.5 to 10	23,281	226	97.1	70.4 (58.7, 83.7)	67,837	468	69.0	65.4 (58.3, 73.0)
Missing	11,863	495	417.3	164.9 (147.1, 184.2)	9,465	370	390.9	137.7 (120.5, 156.7)
Alcohol Consumption								
Unhealthy	58,958	395	67.0	102.01 (89.73, 115.52)	22,574	95	42.1	92.1 (69.6, 119.7)
Moderate	65,493	852	130.1	73.85 (68.52, 79.49)	69,006	528	76.5	69.9 (62.8, 77.6)
Light or non-drinker	155,008	2417	155.9	103.90 (99.64, 108.28)	244,876	3302	134.8	93.8 (67.5, 97.2)
Missing	5,576	102	182.9	88.27 (68.85, 111.47)	4,076	53	130.0	83.6 (57.1, 117.5)

**Fig 1:**
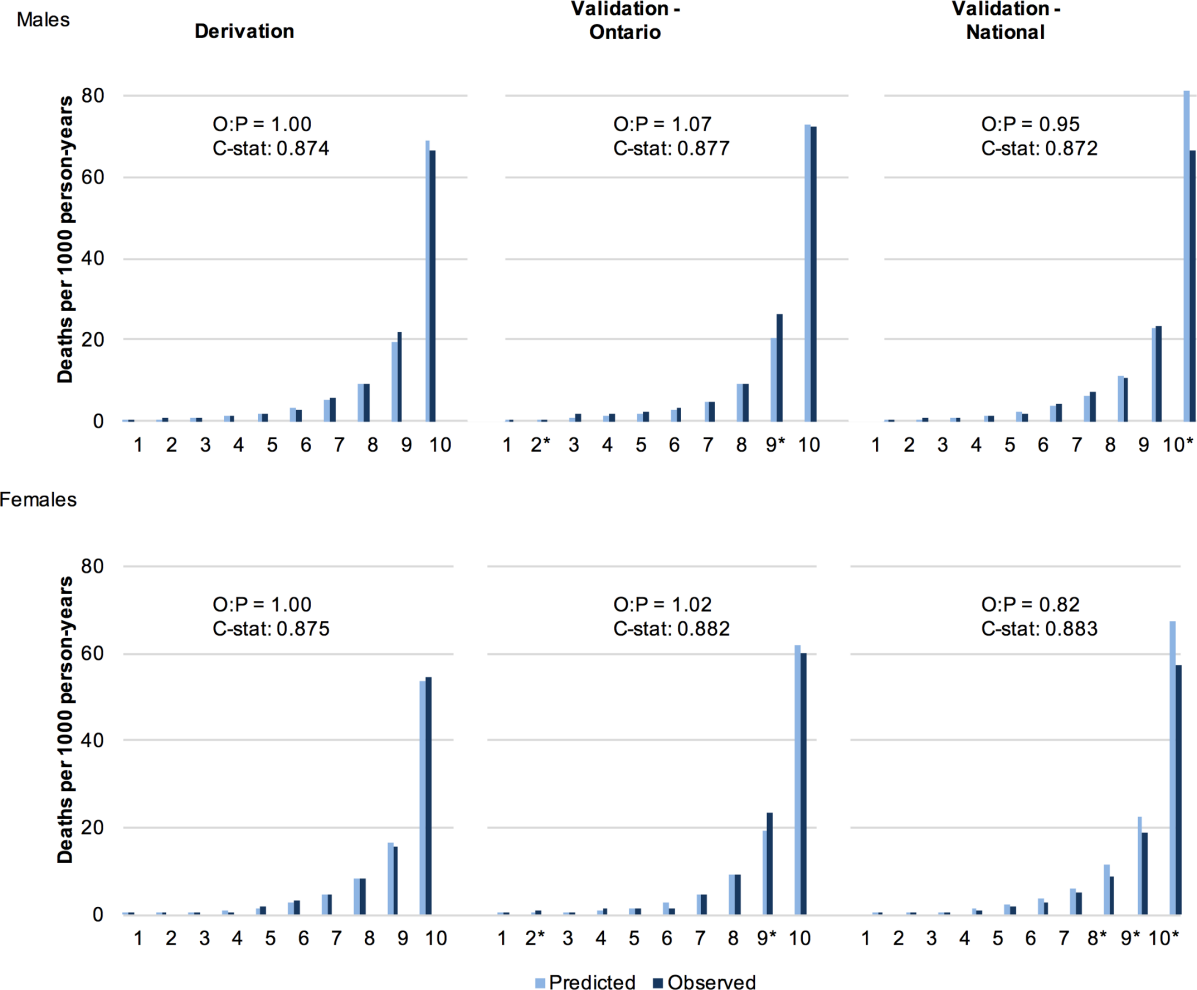
Observed and predicted 5 y risk of death by risk decile for males and females. Predicted deaths from full MPoRT model (incorporating age, health behaviours, sociodemographic, and disease indicators) *Statistically significant difference. Abbreviations: O = observed; P = predicted; C-stat = C-statistic.

**S5 Table** and **S6 Table** describe the characteristics of the multivariable risk model for males and females respectively. Discrimination of all models was high for both males and females (C-statistic in the final model: 0.874 [95% CI: 0.867–0.881] and 0.875 [0.868–0.882], respectively). Using age and behaviours as the only predictors, 6 of 58 predefined subgroups for each of male and female cohorts showed greater than 20% difference between the predicted and observed deaths. Calibration improved with the addition of sociodemographic and disease indicators, with observed and predicted deaths in none of the 58 subgroups showing greater than 20% difference for each sex. **Fig 1** illustrates the close approximation between predicted and observed deaths by risk decile.

Generally, there was a small attenuation of health behaviour hazards when sociodemographic and disease exposures were added to the model. For example, the male hazard ratio for heavy smoking decreased from 3.01 in the initial model (age and health behaviours only) to 2.83 in the full model (fully specified). Removing the first 2 y of study observation (sensitivity analysis) resulted in slight increases in health behaviour hazards except for physical activity, in which the hazard ratios were slightly attenuated.

### Evaluation of MPoRT Using Validation Data

The C-statistic in the Ontario validation cohort was 0.877 for males (95% CI: 0.864–0.889) and 0.882 for females (0.871–0.893). In the Canadian validation cohort, the C-statistic was 0.872 for males (95% CI: 0.865–0.879) and 0.883 for females (0.876–0.889). **Fig 1** shows close approximation between observed and predicted risks for both validation cohorts. **S5 Table** and **S6 Table** show that risk factor hazard ratios were similar for most behavioural factors and other exposures when examined using the national validation cohort. In general, there was slight attenuation of smoking hazard ratios (for heavy smoking, the hazard ratio decreased from 2.83 to 2.81 for males and 3.26 to 3.00 for females); however, the other behavioural factors showed slight increases. **S4 Fig** and **S5 Fig** show good calibration of the behaviour subgroups in the Ontario validation cohorts. For the national validation cohorts, there was modest over-prediction in females for most behaviour subgroups; however, calibration remained robust for males (**S4 Fig** and **S5 Fig**).

**S7 Table** and **S8 Table** show the final model parameters. MPoRT parameters, including variable description, derivation and calibration are available as Predictive Modelling Markup Language (PMML) and Lime questionnaire files (See: **S2 Text**, **S3 Text**, https://github.com/Ottawa-mHealth/predictive-algorithms) [41,42]. Online version of MPoRT is available at https://www.projectbiglife.ca

### The Burden of Health Behaviours in Canada, 2010

**Fig 2:**
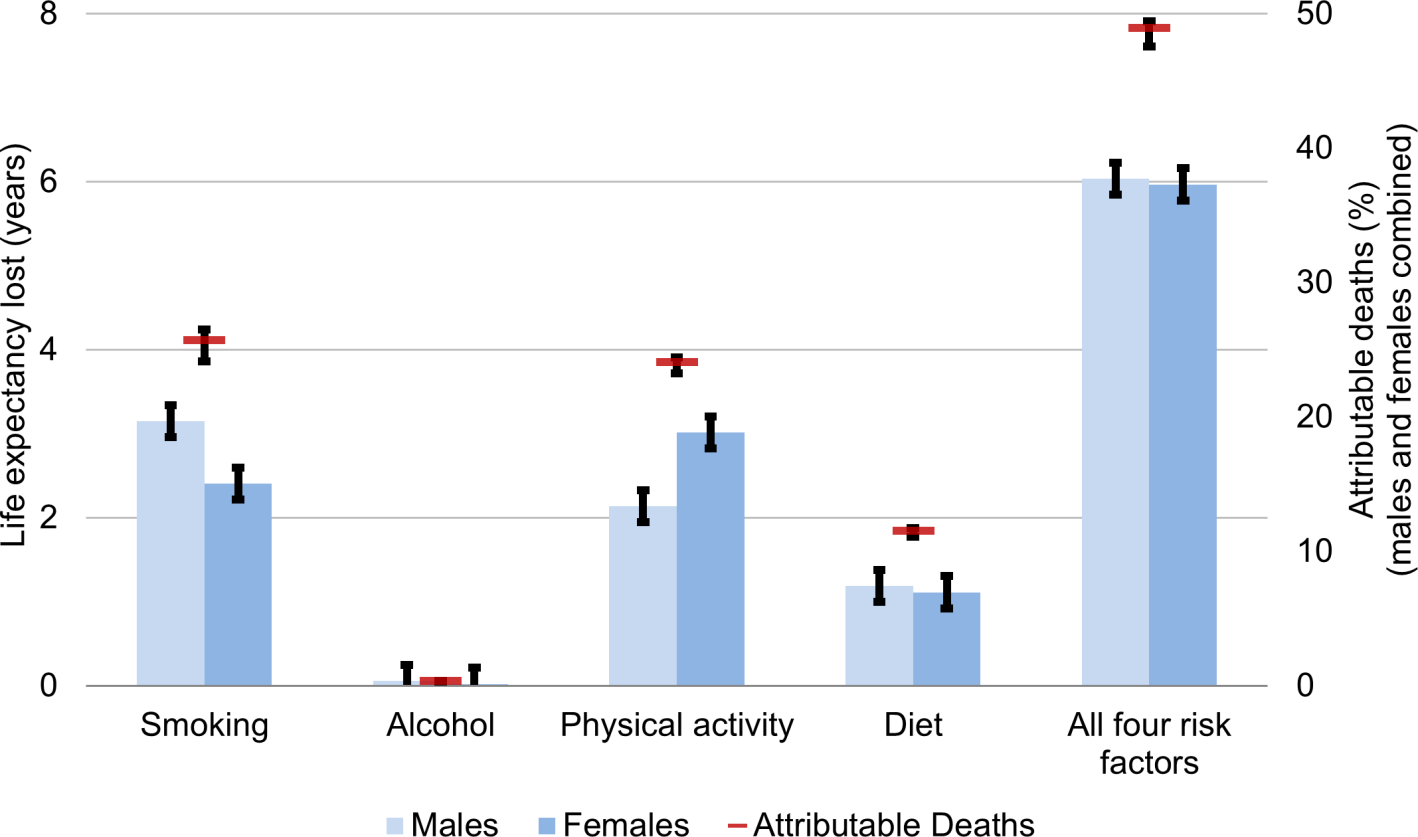
Unhealthy behaviour attribution to life expectancy lost and mortality for Canadians aged 20 and older, 2010. Life expectancy gain and attributable deaths from full MPoRT model (incorporating age, health behaviours, sociodemographic and disease indicators) applied to Canadian national cohort. Error bars represent 95% confidence intervals.

**Fig 2** shows the health behaviour attribution of deaths and life expectancy lost in Canada in 2010 after calibration to the observed Canadian death rates (predicted life expectancy from MPoRT was 79.0 y for males and 83.6 y for females; after re-calibration the predicted and observed life expectancy was 79.3 [males] and 83.4 [females] years). For the 2010 Canadian population, unhealthy-behaviour–attributable life expectancy lost was 6.0 y for both men and women (for men 95% CI: 5.8 to 6.3 for women 5.8 to 6.2). This estimate represents the estimate period life expectancy if there were no exposure to smoking, physical inactivity, poor diet, or unhealthy alcohol consumption. Canadian female unhealthy-behaviour–attributable life expectancy lost was also 6.0 y higher (95% CI: 5.8–6.2) (89.3 y, up from 83.4 y). In 2010, there were 189,000 deaths (males and females combined), of which the four unhealthy behaviours attributed 94,400 deaths (49.8% [95% CI: 48.5–50.4]). Smoking was the leading risk factor for males (smoking-attributable life expectancy loss of 3.1 y), and physical inactivity was the leading risk factor for females (physical inactivity attributable life expectancy loss of 3.0 y). Due to its lower risk hazard, diet had a smaller burden on life expectancy compared to smoking and physical inactivity. Excess alcohol had a small attribution to mortality and life expectancy lost.

**Fig 3:**
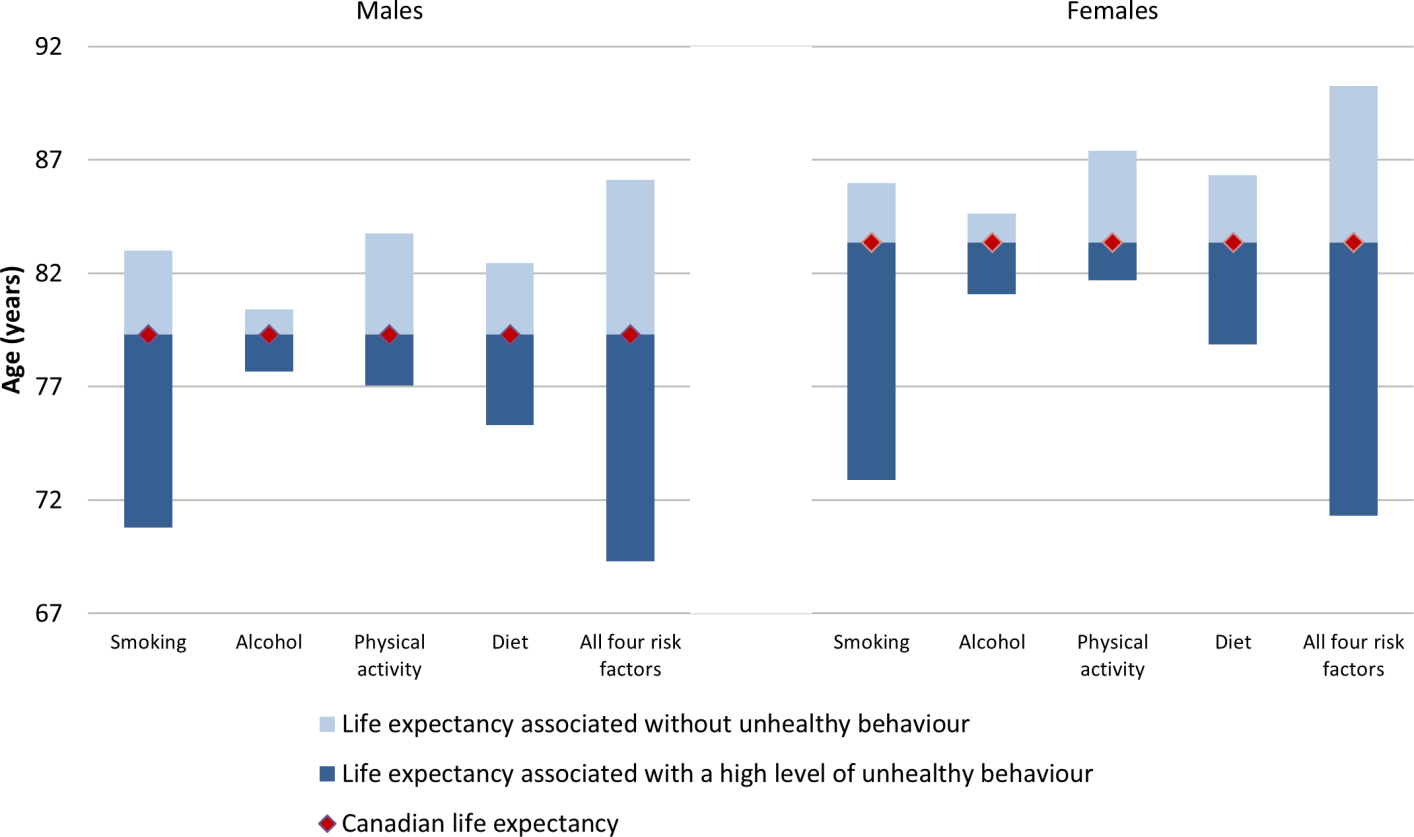
Life expectancy for Canadians aged 20 and older associated with healthy versus high level of unhealthy exposure for selected behaviours, relative to average Canadian life expectancy, 2010. Gain or loss of life expectancy from full MPoRT model (incorporating age, health behaviours, sociodemographic, and disease indicators) applied to Canadian national cohort.

**Fig 3** shows the life expectancy of people associated with a healthy reference exposure and exposure to all four unhealthy behaviours. For males, there was a 16.8 y life expectancy difference (86.1 versus 69.3 y). For females, there was an 18.9 y life expectancy difference (90.2 versus 71.3 y).

**Table 5** illustrates how burden can be estimated for equity purposes. Estimates are shown for smoking and two different measures of socioeconomic position, education and neighbourhood deprivation, but these estimates could be calculated for other socioeconomic exposures measured in the CCHS and for any combination of behavioural risk factors. Life expectancy for different social groups varied more for men than women (e.g., a 5.4 y difference in life expectancy between men with post-secondary education compared to less than high school education; for women this difference was 3.3 y). Smoking was associated with approximately 41% of the difference in life expectancy across education levels for men and 36% for women. After deleting smoking as a cause of death, life expectancy remained lowest for men and women with less than high school education, a reflection of unhealthy exposure to other risk factors.

**Table 5:** Smoking-attributable mortality by smoking status and sociodemographic groups for males and females.

Characteristics	Multivariable predictive approach			
	Attributable deaths	Life expectancy	Smoking-deleted life expectancy	Smoking attributable life expectancy lost
**Male—overall**	28%	79.3	82.4	3.1
**Smoking status**				
Heavy smoker	65%	70.8	80.3	9.5
Light smoker	59%	73.6	82.1	8.5
Former heavy smoker	27%	78.1	81.9	3.8
Former light smoker	20%	80.2	82.7	2.5
Non-smoker	0%	83.0	83.0	0
**Sociodemographic**				
Neighbourhood deprivation				
High	31%	76.4	80.5	4.2
Moderate	27%	79.4	82.5	3.1
Low	22%	82.0	84.1	2.1
Education				
< High school	29%	75.8	80.6	4.8
High school graduate	28%	78.9	82.2	3.3
Post-secondary graduate	27%	81.1	83.7	2.6
**Female—overall**	23%	83.4	85.8	2.4
**Smoking status**				
Heavy smoker	69%	72.9	86.0	13.2
Light smoker	55%	78.2	85.1	6.9
Former heavy smoker	46%	79.6	85.3	5.6
Former light smoker	29%	83.0	86.0	3.1
Non-smoker	0%	86.0	86.0	0
**Sociodemographic**				
Neighbourhood deprivation				
High	27%	81.0	84.2	3.2
Moderate	23%	83.6	85.9	2.4
Low	21%	85.2	87.3	2.1
Education				
< High school	22%	81.2	84.6	3.3
High school graduate	25%	83.3	85.9	2.6
Post-secondary graduate	24%	84.5	86.7	2.1

## Discussion

Our study estimated the burden of unhealthy behaviours in Canada using a multivariable predictive approach and the newly developed MPoRT algorithm. We developed the MPoRT algorithm using community health survey data individually linked to death records. We then applied the algorithm to Canada’s a recent population health survey and calculated mortality risk for each respondent.

Four unhealthy behaviours: smoking, physical inactivity, poor diet, and unhealthy alcohol consumption attributed 50% of deaths in Canada, equivalent to approximately 6 y of life expectancy lost. Smoking was the leading unhealthy behaviour contributing to deaths for men, despite a prevalence that continues to decrease in Canada (22% of Canadians were current smokers in 2010). The burdens from poor diet and physical inactivity closely follow, reflecting the high prevalence of Canadians who report unfavourable exposure to these risk factors. The burden of excess alcohol was small, which was a reflection of a low prevalence of heavy drinking except at younger ages, where baseline mortality risk is low.

Our study offers two main contributions to measuring the burden from unhealthy behaviours. First is the development of a population-based all-cause mortality risk algorithm focusing on health behaviours. Mortality risk algorithms are uncommonly developed and, to our knowledge, MPoRT is the only mortality risk algorithm based on a population health survey and/or behavioural risk factors [43]. MPoRT had very good predictive accuracy with high discrimination (the ability to distinguish between people at high and low risk). Second, this study showed that it is feasible to estimate the burden of unhealthy behaviours using a multivariable risk algorithm that offers flexibility for novel uses. In the process, we demonstrated that mortality risk can be used to calculate a range of intuitive measures such as overall life expectancy and life expectancy for people with different health and sociodemographic profiles. Attribution measures include attributable life expectancy lost and total mortality.

### Use in Different Population Settings

Population health surveys are available in many countries, and an increasing number of countries are able to link these surveys to death certificates. These linked surveys provide the opportunity for a more comprehensive assessment of MPoRT’s predictive accuracy in those countries [44,45]. However, MPoRT can also be used in settings without linked population health surveys.

When used in a new setting, a predictive algorithm such as MPoRT should meet two criteria: discrimination and calibration (accuracy). We expect that MPoRT will have high discrimination in other populations. Typically, discrimination erodes when an algorithm is validated in new populations, but we found that MPoRT’s discrimination remained high (and was slightly higher for women) in the national Canadian validation cohort compared to the provincial Ontario derivation cohort. This likely reflects a more heterogeneous distribution of mortality risk in the national population, as well as the strong influence of health behaviours and other included risk factors on all-cause mortality.

While discrimination often translates from development to application, the same cannot be said of calibration; this can be seen in the clinical setting, where risk algorithms have been largely developed and used [46,47]. An algorithm is considered to be well calibrated if predicted risk closely approximates observed risk, and good calibration of baseline risk is especially important for population health research [48]. Fortunately, unlike in the clinical setting, straightforward opportunities are available to re-calibrate population risk algorithms, as demonstrated in our study [36].

Prior to use in other settings, MPoRT will require an assessment of calibration; our recommendation is to re-calibrate it for most settings. In our study, MPoRT maintained very good calibration when we applied it to a recent national survey data (predicted risk and observed mortality were almost equal). As an illustration of the process that would be required in other settings, we re-calibrated the age- and sex-specific predicted risk to the observed deaths in the new population (Canada) [36]. Additional re-calibration for risk factor exposure can be performed by centering risks on the distribution of risk exposure in the target population. The need for re-calibration would not indicate that MPoRT has poor predictive accuracy; rather, it would signal that factors beyond those included in MPoRT are influencing baseline risk in the new population. Re-calibration adjusts for these factors—conserving the purpose of MPoRT to discriminate risk based on health behaviours (smoking, alcohol, diet, and exercise).

### Comparison with Existing Approaches

**Fig 4:**
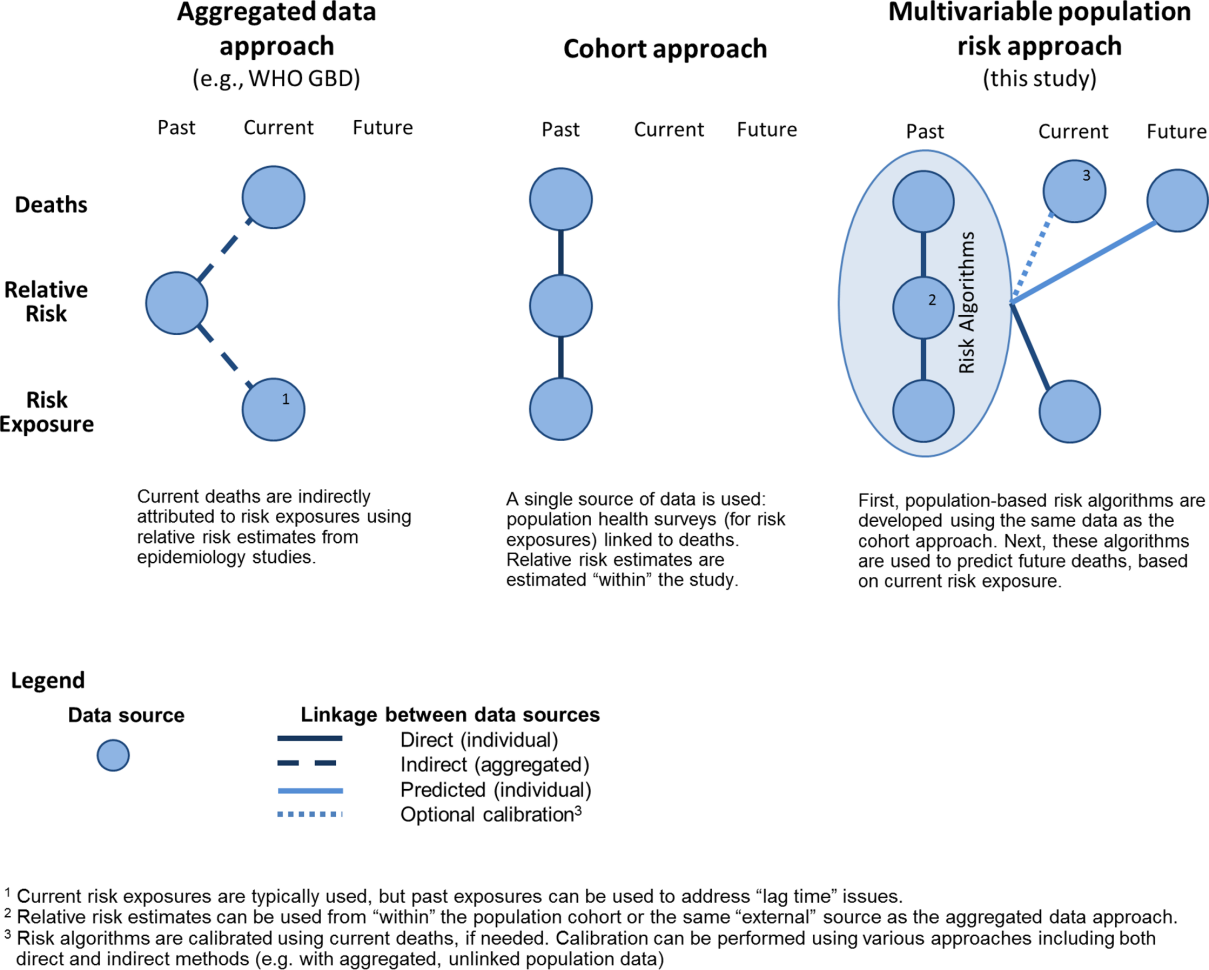
Comparison of approaches to estimate health behaviour burden.

The multivariable predictive approach combines characteristics of the two leading methods of estimating burden of health behaviours—aggregated data and population cohort approaches—and builds on them to provide the potential for a range of uses. The aggregated data approach is useful because it combines three data elements that are commonly available in many settings: incidence of outcomes, prevalence of risk factor exposure, and hazard ratios summarizing the association between risk factors and outcomes. Similarly, the multivariable predictive approach disaggregates the task of estimating burden into three data elements that are then combined (**Fig 4** and **S9 Table** provide additional comparison of the three approaches).

### Outcomes

Rather than using aggregated outcome data (deaths), we calculated the baseline risk of death by applying MPoRT to individual respondents of a population health survey. Unlike the cohort approach, which attributes health behaviour to death within a closed historic population, the aggregated data approach can be applied to observed deaths in external populations, making it widely amenable for the multicountry burden of disease studies and similar research [2,5]. The multivariable predictive approach also uses observed mortality rates, to ensure predicted mortality equals observed mortality.

We examined all-cause mortality, whereas the Global Burden of Disease study assessed disease-specific mortality and then aggregated that information across health behaviours to calculate total burden of health behaviour risks. Examining all-cause mortality typically results in higher burden estimates compared to burden estimated using specific causes when the exposures (in this case, unhealthy behaviours) affect health in many ways. This is the main reason why smoking-attributable deaths are higher in our study (26% of all deaths) than in the World Health Organization’s report (23% of all deaths), which examined only 15 causes of death [49]. There are increasing recommendations to consider all-cause mortality for smoking because, as the United States Surgeon General has stated, “Smoking impacts nearly every organ of the body” [50]. That said, it is feasible to use disease-specific risk predictive algorithms developed for population data [51,52]. As well, risk algorithms can be used for other outcomes, such as health care cost [24,53].

### Exposures

As noted in our Introduction, all three approaches use population-based health surveys to estimate population exposure to different health behaviours. The aggregated data approach uses aggregate estimates of exposure, whereas the cohort and multivariable predictive approach estimate exposure at the individual level—allowing for a range of methods to examine and adjust for how health behaviours are correlated. In addition, we use the same individual-level data to both predict outcomes and consider interactions between health behaviours and other factors, such as social determinants of health. Population-based surveys are a well-suited starting point for measuring health behaviours from an equity perspective because these surveys typically include sociodemographic questions such as education, work history, income, ethnicity, and immigrant status. Furthermore, the burden of low socioeconomic position can be estimated using the same approach that we used to estimate the burden from behavioural risks. Using individual-level data to examine the combined effect of socioeconomic position and health behaviours allows consideration of clustering or collinearity with fewer assumptions than use of aggregate data.

### Hazard Ratios

It is in the final of the three data components, namely risk factor hazard ratios, where the multivariable predictive approach straddles the other two approaches and further demonstrates its flexibility. An underlying assumption for all approaches is that burden estimates for behavioural risks reflect a causal understanding of those risks. The multivariable predictive approach can use estimated hazard ratios from any source, whether “within study” (i.e., from the same study data) or from other studies. Ascertaining relative risks or hazards separately (as in the aggregated data approach) has the potential advantage of being more generalizable across settings, especially when risk estimates are derived from meta-analyses or pooled cohort studies that focus specifically on assessing causal relationships.

We performed burden estimates using a single model, meaning we used MPoRT for both predictive and associative purposes (i.e., to estimate both baseline mortality risk and attributable burden from behavioural risks). However, it is also entirely reasonable to use MPoRT solely for the purpose of estimating the baseline risk of mortality and then to use behavioural risk hazards calculated from the same population health surveys, but specified for causal purposes. For transparency, we present hazard ratios from four alternative models with varying degrees of adjustment for confounding and inclusion of mediating risks (exposures that are on the pathway between health behaviours and mortality, such as body mass index and diabetes). We found that the hazard ratios for health behaviours were attenuated only slightly by the addition of potential confounders and mediators. This suggests that the model has appropriate specification and the burden estimates are robust. That said, it could be argued that using hazard ratios from a model with mediators resulted in over-adjustment and subsequent under-estimation of burden, and that we should have calculated burden using hazard ratios from our model, which did not include mediators (see **S5 Table** and **S6 Table**). Similarly, it was possible to generate burden estimates without adjusting for sociodemographic factors (our first model) or to estimate the burden of sociodemographic factors using hazards for sociodemographic factors. New mediation analyses are being developed that will allow further flexibility when estimating burden from health behaviours [54].

Nonetheless, the “within study” hazard ratio estimates we used had the same magnitude, dose-response, and rank order as hazard ratios from other studies and reviews that examined unhealthy behaviours from a causal perspective. Furthermore, as opposed to hazard ratios from external sources, the use of unhealthy behaviour hazards had the advantage of consistent ascertainment throughout the burden calculation, which has been shown to have an important influence on burden estimates [14,55]. Additionally, we were able to include age interaction and ascertain risk with greater specificity (e.g., physical activity as a continuous measure [METs] instead of the more common approach of measuring using three or four activity levels). Greater specificity of risk exposures allowed us to examine specific population targets, recommendations, or counterfactual burden estimates that considered small changes in risk exposure.

### Time

The three approaches to measure burden of unhealthy living have different time perspectives: the cohort approach has a historic perspective, the aggregated data approach has a recent or current perspective, and the multivariable predictive risk approach has a current or future perspective (see **Fig 4**). The historic cohort perspective reflects singular use of historical cohort data. The aggregated data approach answers the question: “What is the health behaviour attribution of current deaths, based on past health behaviours?” That said, the calculations usually combine current health behaviours with current health outcomes—disregarding the lag time between health behaviour exposure and many chronic disease outcomes [14]. The unique future perspective of the multivariable predictive approach answers the question: “Based on current health behaviours, what is the future risk of death?”

Life expectancy, a summary measure of mortality, is a related time concept. All three approaches are used to estimate period life expectancy—the main measure used in this study. Period life expectancy (also known as actuarial life expectancy), is calculated using “period,” or cross-sectional, mortality and holds an assumption of a stationary population, meaning mortality patterns do not change over time [38]. Period life expectancy should not be confused with cohort life expectancy. For population purposes, cohort life expectancy is typically calculated after everyone in the cohort dies and, therefore, is calculated for historic birth cohorts (for examples, see The Human Mortality Database) [56].

The population cohort and multivariable predictive approaches can estimate closed-cohort mortality risk; for example, MPoRT can be used to estimate 5 y age-specific mortality risk. An advantage of the multivariable predictive approach is the ability to estimate the future risk of mortality based on current health behaviours. The future health perspective is helpful for examining the potential effectiveness of preventive scenarios [8].

### Advantages of Using Individual Exposure Data

We argue that the multivariable predictive approach offers flexibility and transparency when calculating population burden. In addition to its limited ability to incorporate differences in risk by sociodemographic status, the aggregated data approach also has challenges addressing the joint distribution of risks, risk interactions, future perspective, and lag-time between exposures and outcomes. For the most part, these challenges arise because the aggregated data approach is macro- or cell-based as opposed to micro-based (using individual exposure data). For example, aggregated outcome data from vital statistics typically lack information about socioeconomic position or individual health behaviours. Deaths can be grouped by socioeconomic neighbourhood and then ecologic burden estimates can be replicated for each aggregated subpopulation [57]. However, this approach is not feasible in many jurisdictions. Furthermore, the multivariable predictive approach allows for examination and estimation of interactions between socioeconomic status, health behaviours, and mortality in ways that are not possible using ecologic, aggregated data [58]. In an era of more readily available micro-data (including so-called “big data”), we should move beyond an approach to burden estimates that has remained largely unchanged for the past 60 y [8].

### Limitations

There are several limitations in both the multivariable predictive approach and the development and application of MPoRT. Measuring risk using population health surveys has inherent limitations because these surveys are usually cross-sectional, telephone-based, self-reported and cover a wide range of topics that allow only brief ascertainment of any particular risk exposure. That stated, the ascertainment of prevalence of behavioural risk factors in population health surveys has become more consistent across countries and there is an increasing number of validation studies that indicate acceptable ascertainment bias [59]. Diet and alcohol are exceptions. For diet, there is considerable variation in ascertainment across population health surveys and few validation studies on brief ascertainment of diet. In the Canadian Community Health Survey (used to develop and apply MPoRT), diet was ascertained using fewer questions (five brief questions on fruit and vegetable intake) than is typical in population health surveys. The questions were converted to a scale of dietary quality shown in previous studies to be related to both mortality and hospital use [16,24]. It is likely that this brief dietary score underestimated burden from diet compared to other, more detailed diet exposure measures [60]. For alcohol, there is consistent under-ascertainment of consumption in most population health surveys. In Ontario the sum of self-reported alcohol consumption is about half the volume of alcohol sold [61]. It is possible to revise alcohol and other health behaviour exposures to adjust for misascertainment bias. Adjustment can be made through various multivariable imputation techniques that consider differential bias across socioeconomic position and other risk factors, whereas the aggregate method adjusts at the aggregate level [62–64]. Calibration of the burden outcome should be performed after adjusting exposure for misascertainment bias to ensure an unbiased overall burden estimate (as described above).

Calculating confidence or uncertainty intervals for burden studies is challenging because there are several sources of error. We calculated confidence intervals for burden estimates that considered stochastic error. We found the confidence intervals were small, reflecting the large sample size of the study data. The confidence intervals would be much larger had we adjusted for more sources of measurement error, including from survey sampling, exposure measurement, or model specification. In general, adjusting for bias from each of these errors will result in larger estimates of burden from unhealthy behaviours: survey sampling usually biases towards selecting healthy people; misclassification of health behaviours and behavioural hazards from an over-fitted survival model will result in conservative burden estimates. New methods are increasingly available to estimate uncertainty and adjust for bias, particularly for a multivariable predictive approach that uses individual-level data [65].

This study does not consider time-varying exposure or exposure mediation (other than sensitivity analyses of potential mediators). Murray et al. consider these issues in their conceptual framework for assessing risk factors, and recent advances have included marginal structural models and g-estimation when time-varying exposure is measured in longitudinal cohorts [66–68]. Unfortunately, population health surveys rarely incorporate longitudinal follow-up of exposures and, therefore, time-varying exposure is not considered in commonly used approaches to measuring burden, including the Global Burden of Disease study.

### Conclusions

Population health surveys are commonly conducted in many countries and can provide the basis of determining health behaviour exposure for burden of disease reporting. Increasingly, these surveys are linked to death and disease outcomes data. These linked health surveys create an opportunity to model burden of disease using individual-based approaches, such as the multivariable predictive approach, or to supplement existing attributable burden approaches. We show that a multivariable predictive approach to estimating burden from unhealthy behaviours is feasible and demonstrates a large burden of life expectancy lost in Canada. Lastly, we show that differential exposure to unhealthy behaviours contributes to large differences in the burden of mortality across socioeconomic groups.

## Supporting Information

S1 Fig Study flow. (TIF)

S2 Fig Mortality hazards for smoking, males, and females. (TIF)

S3 Fig Mortality hazards for physical activity, males, and females. (TIF)

S4 Fig Observed and predicted risk by health behaviour risk factors (smoking, alcohol, physical activity, and diet), male validation cohorts. Predicted deaths from full MPoRT model (incorporating age, health behaviours, sociodemographic and disease indicators). *Statistically significant difference.
(TIF)

S5 Fig Observed and predicted risk by health behaviour risk factors (smoking, alcohol, physical activity, and diet), female validation cohorts. Predicted deaths from full MPoRT model (incorporating age, health behaviours, sociodemographic, and disease indicators). *Statistically significant difference.
(TIF)

S1 Table Definitions for exposure variables. (PDF)

S2 Table Crude and age-standardized death rates per 10,000 person-years for sociodemographic groups. (PDF)

S3 Table Crude and age-standardized death rates per 10,000 person-years for disease groups. (PDF)

S4 Table Crude and age-standardized death rates per 10,000 person-years for geographic groups. (PDF)

S5 Table Male derivation, validation, and application data. (PDF)

S6 Table Female derivation, validation, and application data. (PDF)

S7 Table MPoRT formula for probability of 1 y survival for use in life tables. (PDF)

S8 Table MPoRT Beta coefficients for male and female models. (PDF)

S9 Table Comparison of three approaches to calculate the burden of health behaviours. (PDF)

S1 Text Calculation of confidence intervals. (PDF)

S2 Text Predictive Modelling Markup Language (PMML) file. (PDF)

S3 Text Lime questionnaire file. (PDF)

S4 Text STROBE Statement—checklist of items that should be included in reports of observational studies. (PDF)

## Abbreviations

CCHS: Canadian Community Health Survey
MET: metabolic equivalent of task
MPoRT: Mortality Population Risk Tool
PMML: Predictive Modelling Markup Language
