# Supplementary figures and images for: Measuring Burden of Unhealthy Behaviours Using a Multivariable Predictive Approach: Life Expectancy Lost in Canada Attributable to Smoking, Alcohol, Physical Inactivity, and Diet
Source: PLoS Med. 2016 Aug 16;13(8):e1002082. doi: 10.1371/journal.pmed.1002082 (PMC4986987; doi:10.1371/journal.pmed.1002082)

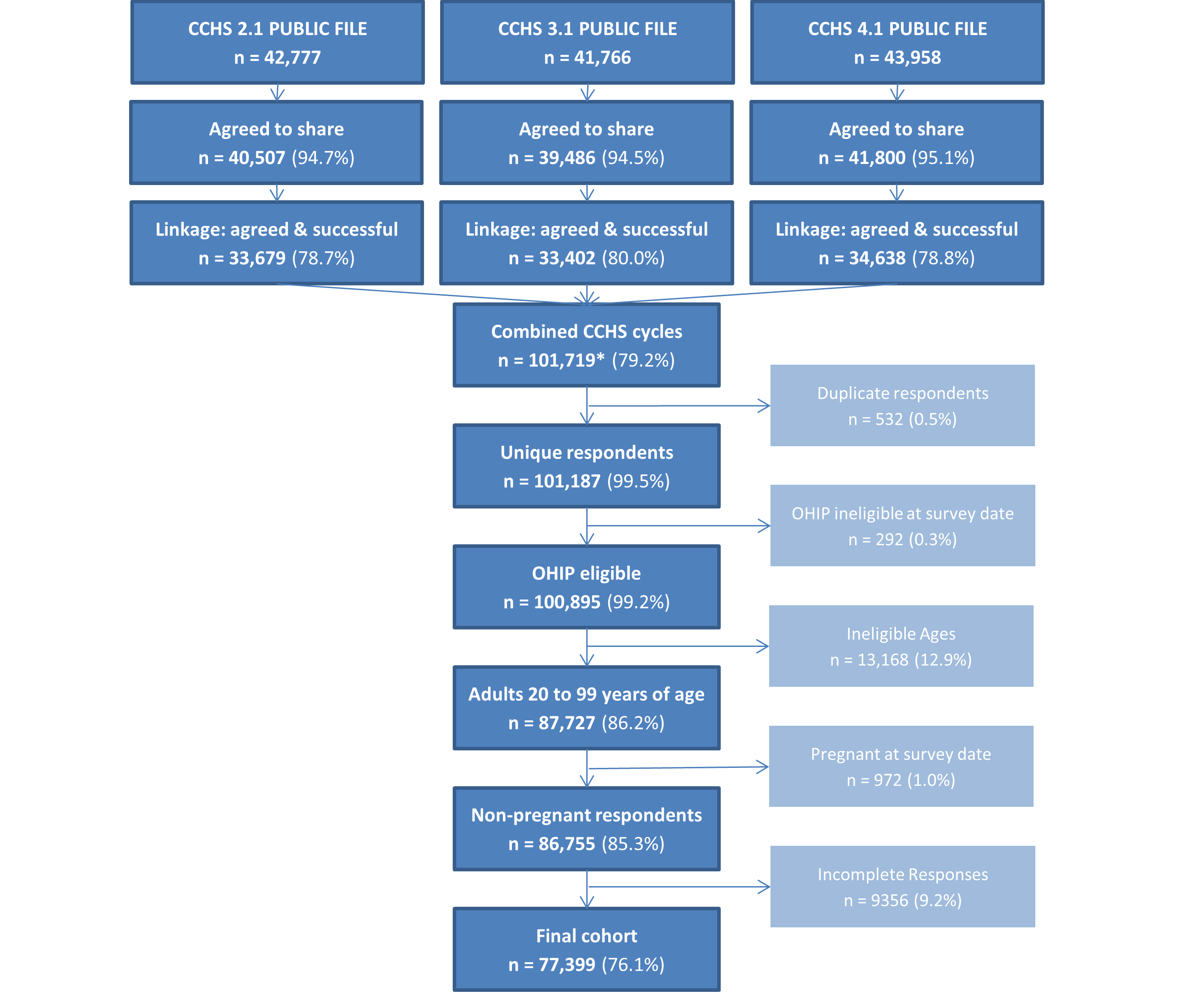

Supplement: S1 Fig — (TIF) [file pmed.1002082.s001.tif]

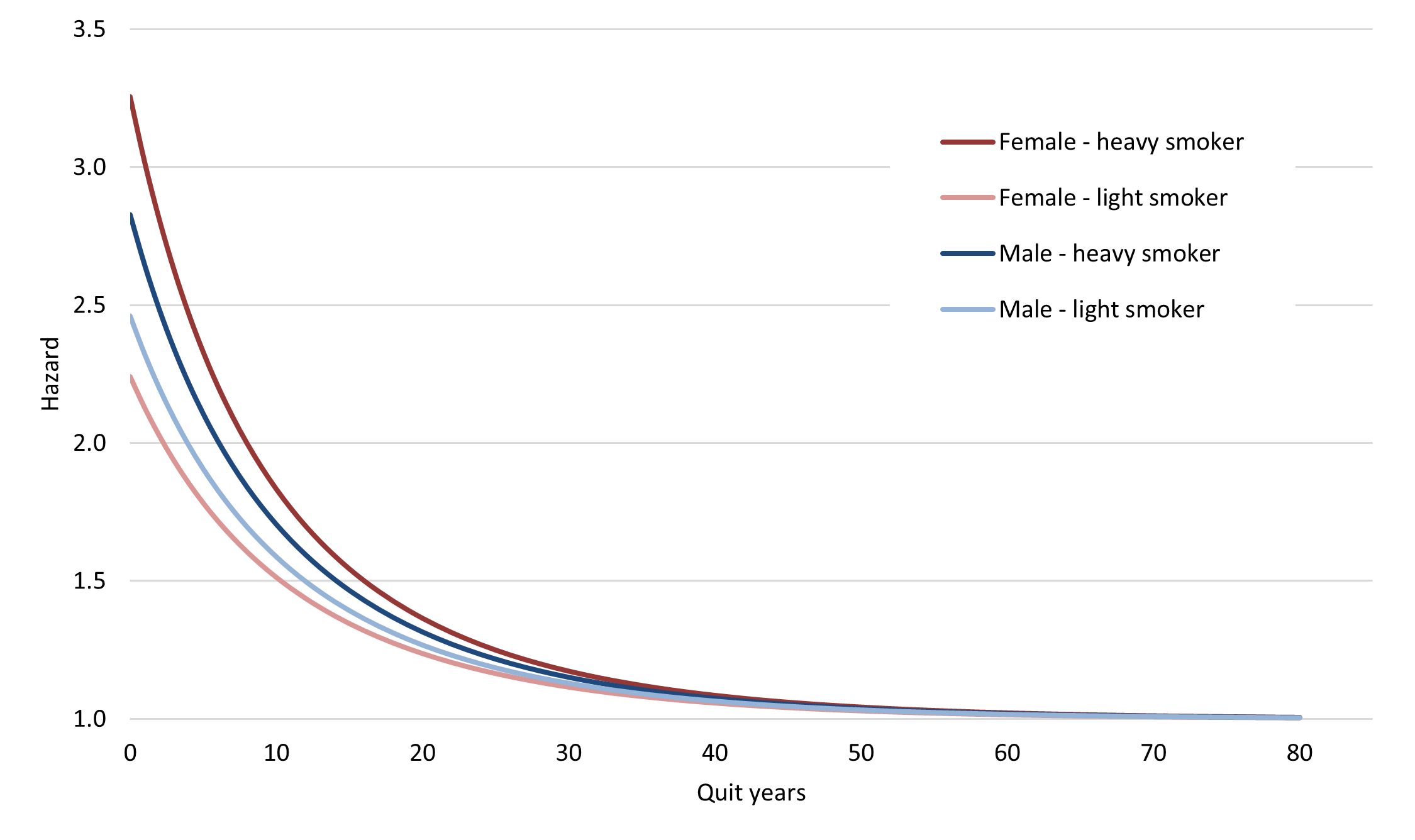

Supplement: S2 Fig — (TIF) [file pmed.1002082.s002.tif]

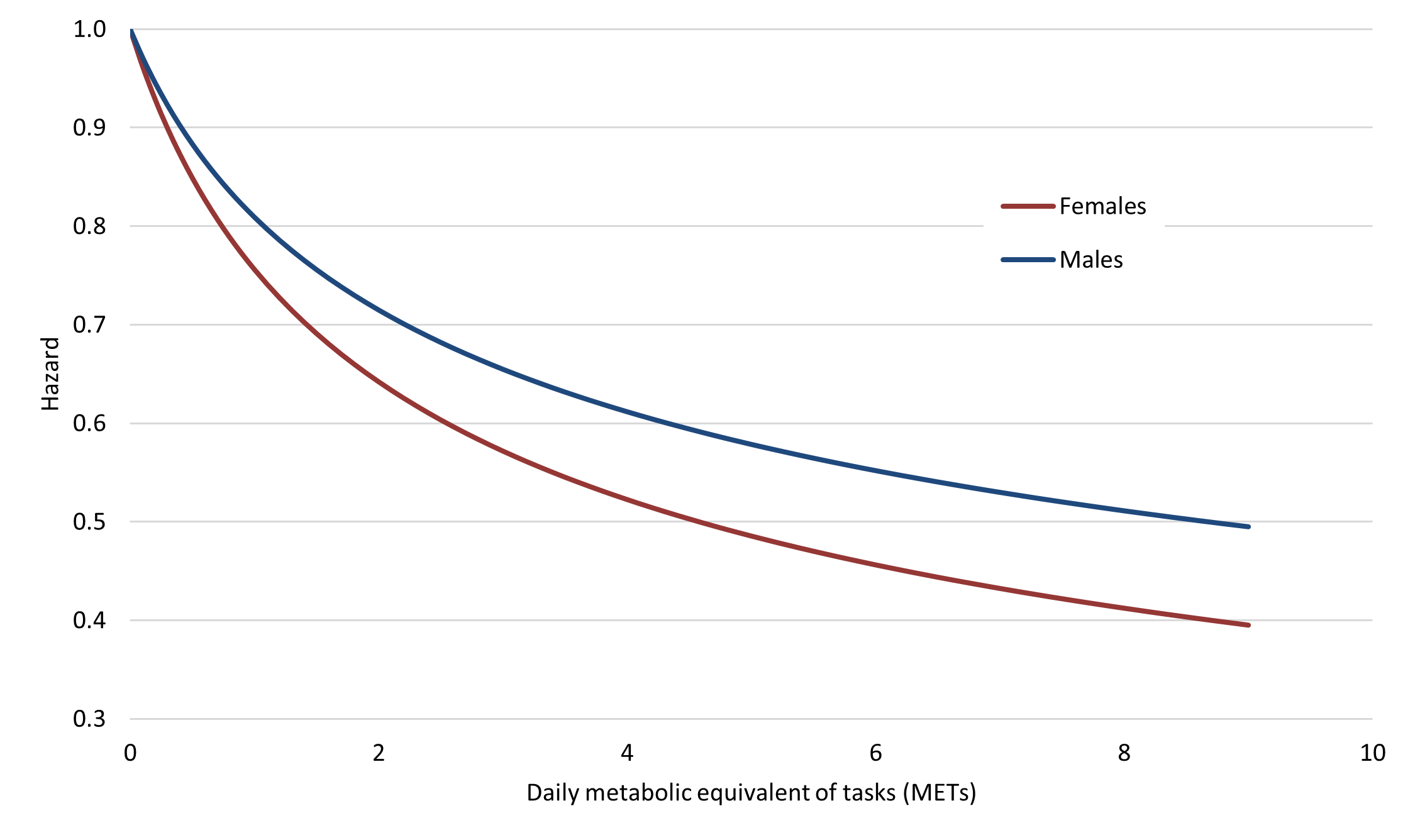

Supplement: S3 Fig — (TIF) [file pmed.1002082.s003.tif]

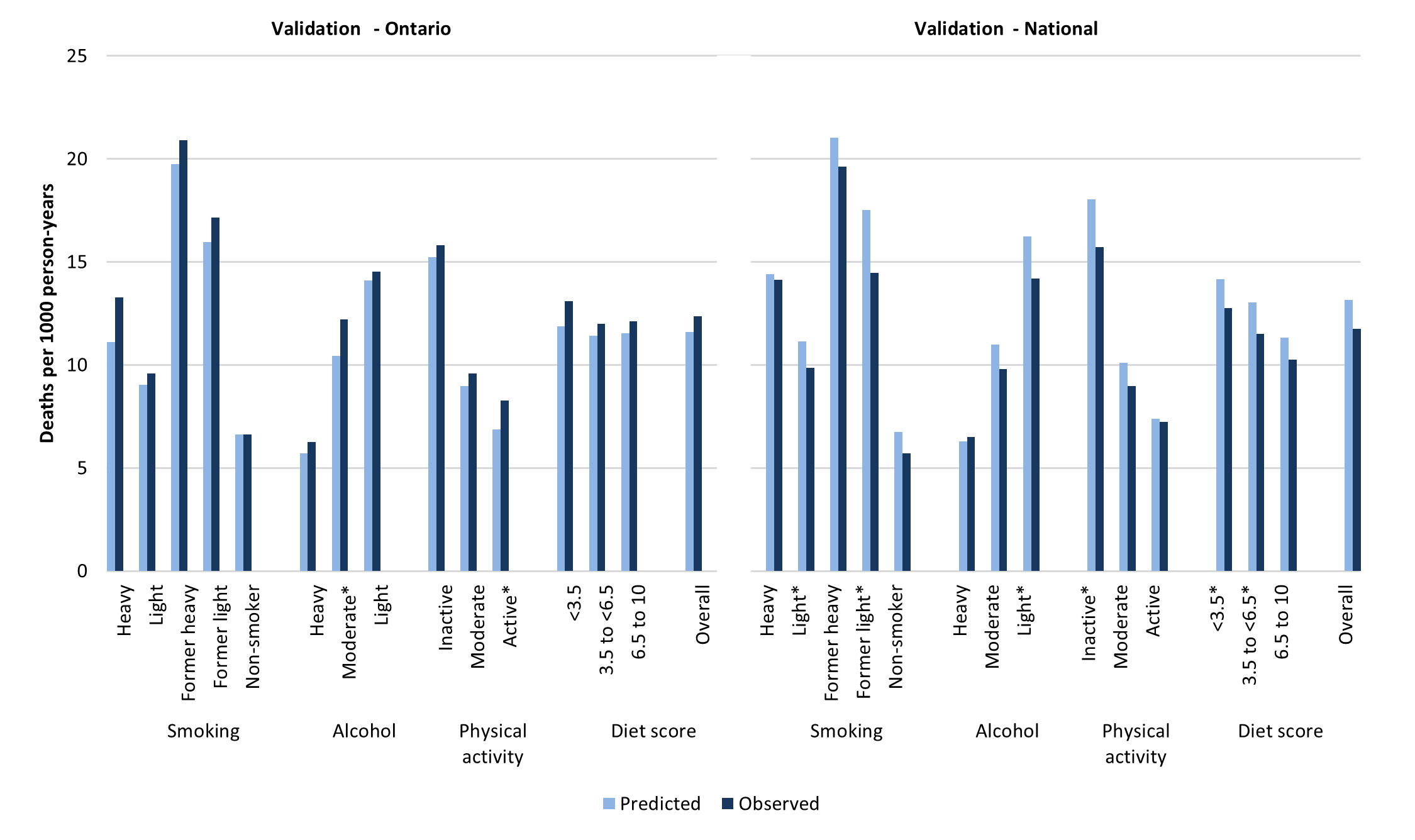

Supplement: S4 Fig — Predicted deaths from full MPoRT model (incorporating age, health behaviours, sociodemographic and disease indicators). *Statistically significant difference. (TIF) [file pmed.1002082.s004.tif]

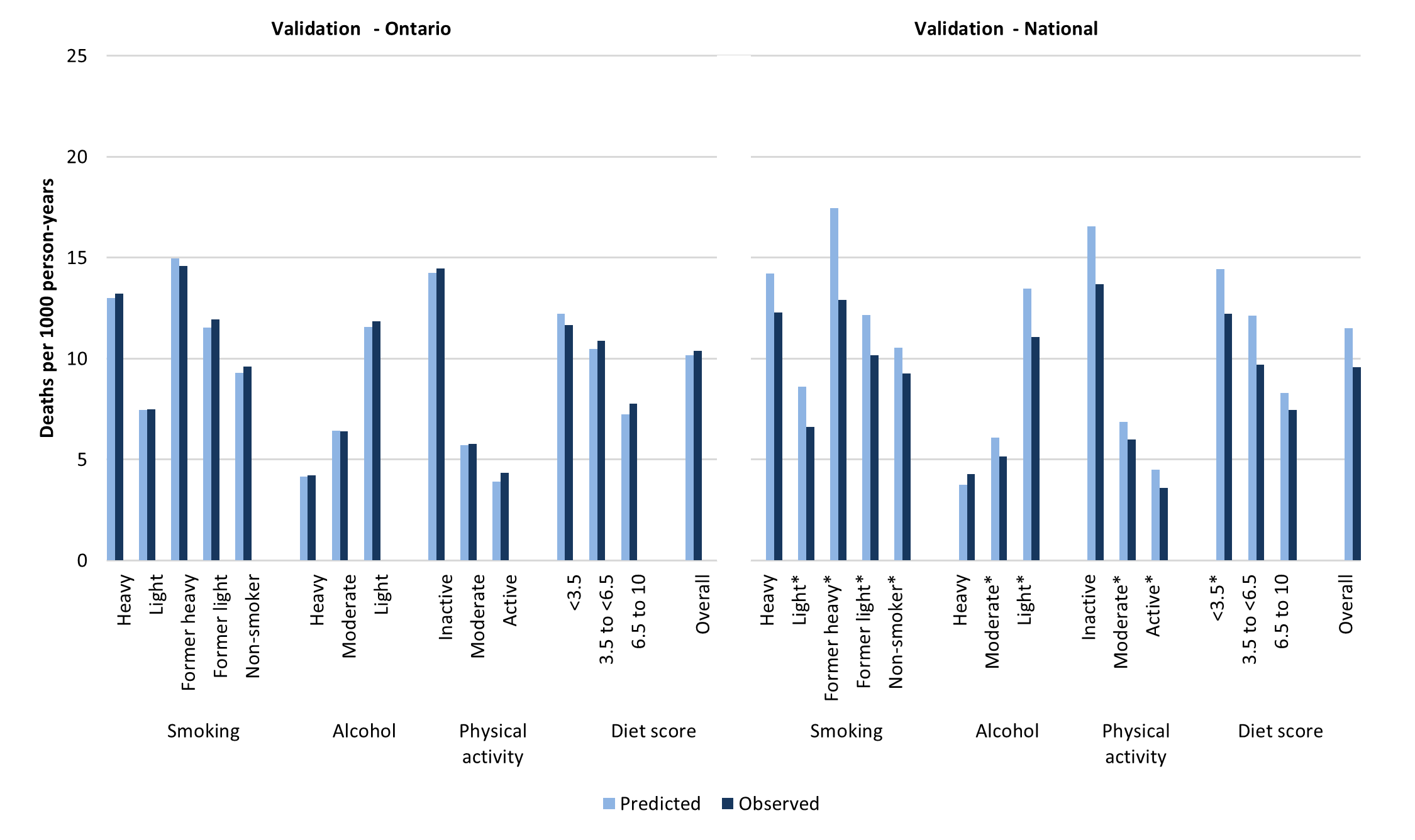

Supplement: S5 Fig — Predicted deaths from full MPoRT model (incorporating age, health behaviours, sociodemographic, and disease indicators). *Statistically significant difference. (TIF) [file pmed.1002082.s005.tif]
