# Supplementary material for: Measuring Burden of Unhealthy Behaviours Using a Multivariable Predictive Approach: Life Expectancy Lost in Canada Attributable to Smoking, Alcohol, Physical Inactivity, and Diet
Source: PLoS Med. 2016 Aug 16;13(8):e1002082. doi: 10.1371/journal.pmed.1002082 (PMC4986987; doi:10.1371/journal.pmed.1002082)
Supplement: S1 Table — (PDF) [file pmed.1002082.s006.pdf]

**S1 Table**. Definitions for exposure variables

| Concept | CCHS survey questions for exposure variables | Type |
|---|---|---|
| Smoking | Type of smoker | Derived |
| | How many cigarettes do you smoke each day now? (Daily smoker) | Collected |
| | How many cigarettes did you usually smoke each day? (Former daily smoker) | Collected |
| | Number of years since stopped smoking daily? (Former daily smoker) | Collected |
| | Number of years since stopped smoking? (Never daily smoker) | Collected |
| | In your lifetime, have you smoked a total of 100 or more cigarettes (about 4 packs)? | Collected |
| Alcohol | During the past 12 months, have you had a drink of beer, wine, liquor or any other alcoholic beverage? | Collected |
| | During the past 12 months, how often did you drink alcoholic beverages? | Collected |
| | How often in the past 12 months have you had 5 or more drinks on one occasion? | Collected |
| | Number of drinks - past week | Derived |
| | Starting with yesterday, that is [day name], how many drinks did you have? - Sunday | Collected |
| | Starting with yesterday, that is [day name], how many drinks did you have? - Monday | Collected |
| | Starting with yesterday, that is [day name], how many drinks did you have? -Tuesday | Collected |
| | Starting with yesterday, that is [day name], how many drinks did you have? - Wednesday | Collected |
| | Starting with yesterday, that is [day name], how many drinks did you have? - Thursday | Collected |
| | Starting with yesterday, that is [day name], how many drinks did you have? - Friday | Collected |
| | Starting with yesterday, that is [day name], how many drinks did you have? - Saturday | Collected |
| Diet | Daily consumption - carrots | Derived |
| | Daily consumption - potatoes | Derived |
| | Daily consumption - fruit juice | Derived |
| | Daily consumption - total fruits and vegetables | Derived |
| Physical activity | Activities / last 3 months | Derived |
| Heart disease | Do you have heart disease? | Collected |
| Diabetes | Do you have diabetes? | Collected |
| Cancer | Do you have cancer? | Collected |
| Stroke | Do you suffer from the effects of a stroke? | Collected |
| BMI | Body Mass Index (BMI) / self-report | Derived |
| | Height (metres) / self-reported | Collected |
| | Weight (kilograms) / self-reported | Collected |
| Education | What is the highest degree, certificate or diploma you have obtained? | Collected |
| Ethnicity | Are you white? | Collected |
| Immigrant | Immigrant Status | Derived |
| | Length of time in Canada since immigration | Derived |
| Rurality | Urban and Rural Areas - 7 levels | Derived |
| Local Health Integration Network | Identified through postal code conversion file using identified Dissemination Area in CCHS survey | Externally Linked |
| Deprivation Index | Identified using Dissemination Area in CCHS survey | Externally Linked |
