# Supplementary material for: Measuring Burden of Unhealthy Behaviours Using a Multivariable Predictive Approach: Life Expectancy Lost in Canada Attributable to Smoking, Alcohol, Physical Inactivity, and Diet
Source: PLoS Med. 2016 Aug 16;13(8):e1002082. doi: 10.1371/journal.pmed.1002082 (PMC4986987; doi:10.1371/journal.pmed.1002082)
Supplement: S2 Table — (PDF) [file pmed.1002082.s007.pdf]

S2 Table. Crude and age standardized death rates per 10000 person-years for sociodemographic groups

	Males				Females			
	Person-years	Deaths	Crude rate	Age standardised rate (95% CI)	Person-years	Deaths	Crude rate	Age standardised rate (95% CI)
Total	285,035	3,766	132.1	96.1 (92.9, 99.3)	340,532	3,978	116.8	89.7 (86.7, 92.7)
Neighbourhood Deprivation								
Low	57,529	580	100.8	78.5 (71.3, 86.2)	64,863	519	80.0	77.5 (70.0, 85.6)
Moderate	177,395	2,359	133.0	95.2 (91.3, 99.3)	211,730	2,476	116.9	88.1 (84.4, 91.9)
High	43,934	722	164.3	123.6 (114.5, 133.2)	56,063	845	150.7	109.4 (101.8, 117.3)
Missing	6,176	105	170.0	105.7 (85.91, 128.66)	7,876	138	175.2	96.8 (79.5, 116.7)
Education								
Less than secondary school	50,340	1,474	292.8	125.6 (117.5, 134.0)	65,182	1,715	263.1	107.6 (100.7, 114.7)
Secondary school	70,331	714	101.5	93.2 (86.3, 100.5)	89,642	957	106.8	87.3 (81.5, 93.5)
Post-secondary	161,625	1,505	93.1	81.8 (77.5, 86.3)	183,369	1,245	66.9	77.5 (72.8, 82.5)
Missing	2,739	73	266.5	126.8 (93.3, 168.4)	2,339	61	260.8	109.9 (76.4, 153.0)
Immigration Status								
Non-immigrant or>45 years in Canada	241,213	3,456	143.3	99.2 (95.8, 102.7)	290,950	3,709	127.5	91.7 (88.6, 94.9)
Immigrated <15 years	14,074	24	17.1	40.8 (24.3, 64.2)	16,265	27	16.6	105.9 (22.6, 304.8)
Immigrated 16 to 30 years	12,980	68	52.4	78.5 (56.7, 106.0)	13,838	69	49.9	79.8 (58.9,105.8)
Immigrated 31 to 45 years	16,427	208	126.6	79.5 (67.0, 93.7)	19,025	157	82.5	78.0 (55.2,107.0)
Missing	341	10	293.5	130.1 (57.2, 253.4)	455	16	351.5	157.3 (83.6, 269.3)
Ethnicity								
White	252,293	3,555	140.9	97.3 (94.0, 100.7)	304,395	3,809	125.1	90.5 (87.4, 93.6)
Non-white	31,793	187	58.8	78.9 (67.4, 91.8)	35,131	157	44.7	75.2 (61.7, 90.7)
Missing	948	24	253.1	117.3 (71.3, 181.8)	1006	12	119.3	79.7 (39.8, 142.6)
