# Supplementary material for: Measuring Burden of Unhealthy Behaviours Using a Multivariable Predictive Approach: Life Expectancy Lost in Canada Attributable to Smoking, Alcohol, Physical Inactivity, and Diet
Source: PLoS Med. 2016 Aug 16;13(8):e1002082. doi: 10.1371/journal.pmed.1002082 (PMC4986987; doi:10.1371/journal.pmed.1002082)
Supplement: S3 Table — (PDF) [file pmed.1002082.s008.pdf]

S3 Table. Crude and age standardized death rates per 10000 person-years for disease groups

	Males				Females			
	Person-years	Deaths	Crude rate	Age standardised rate (95% CI)	Person-years	Deaths	Crude rate	Age standardised rate (95% CI)
Total	285,035	3,766	132.1	96.1 (92.9, 99.3)	340,532	3,978	116.8	89.7 (86.7, 92.7)
Heart Disease								
Yes	23,037	1,079	468.4	161.5 (134.1, 192.9)	23,476	1,007	429.0	162.6 (134.8, 194.5)
No	261,534	2,671	102.1	86.7 (83.3, 90.2)	316,518	2,954	93.3	81.4 (78.3, 84.7)
Missing	464	16	345.1	95.4 (52.5, 159.4)	538	17	315.9	226.8 (112.3, 408.1)
Suffers from Stroke								
Yes	4,510	289	640.8	203.9 (135.0, 295.6)	4,975	277	556.8	195.3 (159.9, 236.3)
No	280,351	3,474	123.9	93.1 (89.9, 96.4)	335,270	3,694	110.2	87.0 (84.0, 90.0)
Missing	174	3	172.7	67.6 (9.4, 231.9)	287	7	243.7	128.7 (39.0, 312.0)
Cancer								
Yes	6,857	427	622.7	221.9 (190.1, 257.5)	7,571	353	466.2	294.7 (241.7, 355.9)
No	277,902	3,329	119.8	91.1 (87.9, 94.4)	332,560	3,617	108.8	84.8 (81.9, 87.8)
Missing	276	10	362.6	135.6 (60.2, 262.2)	401	8	199.7	290.9 (102.7, 647.5)
Diabetes								
Yes	21,117	805	381.2	183.3 (154.9, 215.3)	22,365	712	318.4	153.4 (137.5, 170.6)
No	263,670	2,955	112.1	88.3 (85.1, 91.7)	317,984	3,265	102.7	83.3 (81.0, 86.3)
Missing	247	6	242.9	218.5 (53.0, 592.6)	183	1	54.5	56.3 (1.4, 313.8)
Morbidly Obese								
Yes	13,160	171	129.9	131.4 (105.3, 162.0)	20,325	223	109.7	106.3 (89.0, 126.1)
No	269,270	3,460	128.5	92.8 (89.6, 96.1)	309,847	3,551	114.6	86.6 (83.6, 89.7)
Missing	2,604	135	518.4	184.9 (146.6, 230.2)	10,360	204	196.9	134.2 (115.7, 154.8)
