# Supplementary material for: Measuring Burden of Unhealthy Behaviours Using a Multivariable Predictive Approach: Life Expectancy Lost in Canada Attributable to Smoking, Alcohol, Physical Inactivity, and Diet
Source: PLoS Med. 2016 Aug 16;13(8):e1002082. doi: 10.1371/journal.pmed.1002082 (PMC4986987; doi:10.1371/journal.pmed.1002082)
Supplement: S4 Table — (PDF) [file pmed.1002082.s009.pdf]

S4 Table. Crude and age standardized death rates per 10000 person-years for geographic groups

	Males				Females			
	Person-years	Deaths	Crude rate	Age standardised rate (95% CI)	Person-years	Deaths	Crude rate	Age standardised rate (95% CI)
Total	285,035	3,766	132.1	96.1 (92.9, 99.3)	340,532	3,978	116.8	89.7 (86.7, 92.7)
Local Health Integration Network								
Erie St. Clair	20,135	274	136.1	109.4 (95.2, 125.1)	23,927	333	139.2	98.1 (87.3, 109.8)
South West	35,950	512	142.4	95.5 (87.0, 104.6)	42,684	544	127.5	91.3 (83.3, 100.0)
Waterloo Wellington	17,548	196	111.7	84.5 (72.5, 98.0)	20,539	204	99.3	84.1 (70.6, 99.4)
Hamilton Niagara Haldimand Brant	35,907	527	146.8	99.6 (90.8, 109.1)	43,523	557	128.0	92.3 (84.1, 101.0)
Central West	9,292	93	100.1	103.2 (80.9, 129.9)	10,188	93	91.3	90.1 (72.3, 111.1)
Mississauga Halton	14,178	121	85.3	73.3 (58.5, 90.6)	15,935	135	84.7	81.7 (68.1, 97.2)
Toronto Central	10,738	86	80.1	79.3 (62.9, 98.6)	12,474	121	97.0	87.8 (72.1, 106.0)
Central	16,725	138	82.5	68.5 (57.2, 81.2)	19,045	165	86.6	74.6 (63.0, 87.9)
Central East	27,008	364	134.8	93.4 (83.7, 103.9)	33,274	389	116.9	88.8 (79.9, 98.5)
South East	17,612	308	174.9	112.4 (99.4, 126.6)	22,164	283	127.7	91.7 (80.7, 103.9)
Champlain	26,230	336	128.1	96.1 (85.8, 107.2)	32,931	342	103.9	83.2 (73.7, 93.5)
North Simcoe Muskoka	12,525	152	121.4	84.8 (71.3, 100.2)	14,461	191	132.1	90.0 (77.1, 104.4)
North East	28,868	477	165.2	116.1 (105.1, 127.9)	35,506	448	126.2	96.5 (86.6, 107.2)
North West	12,231	182	148.8	109.5 (93.2, 127.9)	13,822	173	125.2	88.6 (75.5, 103.4)
Missing	87	0	0.0	0.0 (0.0, 0.0)	60	0	0.00	0.0 (0.0, 0.0)
Census Metropolitan Area (CMA)								
CMA: ≥1.5 million	56,703	489	86.2	75.2 (68.5, 82.5)	64,771	588	90.8	82.4 (75.7, 89.6)
CMA: 500,000-1,499,999	29,488	345	117.0	92.2 (82.6, 102.7)	36,136	355	98.2	80.2 (71.2, 90.1)
CMA: 100,000- 499,999	90,931	1,225	134.7	101.1 (95.2, 107.3)	110,499	1,366	123.6	92.9 (87.7, 98.3)
CMA: 10,000- 99,999	45,592	770	168.9	110.3 (102.2, 118.8)	56,718	766	135.1	97.0 (89.5, 105.0)
Non-CMA: strong MIZ[a]	21,303	299	140.4	95.2 (84.5, 107.0)	24,728	258	104.3	82.1 (71.8, 93.5)
Non-CMA: moderate MIZ[a]	24,402	401	164.3	102.1 (91.0, 114.2)	28,845	398	138.0	92.7 (83.2, 103.0)
Other	16,616	237	142.6	95.0 (82.6, 108.7)	18,835	247	131.1	95.7 (83.3, 109.4)

[a]Metropolitan Influenced Zone
