# Supplementary material for: Measuring Burden of Unhealthy Behaviours Using a Multivariable Predictive Approach: Life Expectancy Lost in Canada Attributable to Smoking, Alcohol, Physical Inactivity, and Diet
Source: PLoS Med. 2016 Aug 16;13(8):e1002082. doi: 10.1371/journal.pmed.1002082 (PMC4986987; doi:10.1371/journal.pmed.1002082)
Supplement: S6 Table — (PDF) [file pmed.1002082.s011.pdf]

S6 Table. Female derivation, validation and application data

	Model Development (Deaths=3189)			Canadian national cohort	Sensitivity Analysis
	Model 1<br>Age & Behaviours	Model 2<br>Model 1 + Sociodemographic Indicators	Full Model<br>Model 2 + Disease Indicators	Full Model Using National Cohort (CCHS 2.1)<br>(Deaths=1756)	Full Model Without Years 1 and 2 of Follow-Up<br>(Deaths=2622)
Age	1.09 (1.09, 1.10)	1.09 (1.09, 1.10)	1.09 (1.09, 1.10)	1.10 (1.09, 1.10)	1.09 (1.08, 1.10)
Age above spline knot	1.02 (1.01, 1.03)	1.02 (1.01, 1.03)	1.03 (1.02, 1.04)	1.03 (1.01, 1.04)	1.03 (1.02, 1.04)
Smoking					
Heavy	3.50 (3.09, 3.96)	3.33 (2.94, 3.78)	3.26 (2.87, 3.70)	3.00 (2.56, 3.52)	3.38 (2.94, 3.90)
Former heavy (quit 10 years)	1.90 (1.78, 2.03)	1.85 (1.74, 1.98)	1.83 (1.72, 1.96)	1.76 (1.62, 1.91)	1.87 (1.74, 2.01)
Former heavy (quit 20 years)	1.39 (1.35, 1.44)	1.37 (1.33, 1.42)	1.36 (1.32, 1.41)	1.34 (1.28, 1.39)	1.38 (1.33, 1.43)
Former heavy (quit 30 years)	1.18 (1.17, 1.20)	1.18 (1.16, 1.20)	1.17 (1.15, 1.19)	1.16 (1.14, 1.19)	1.18 (1.16, 1.20)
Light	2.32 (2.09, 2.58)	2.25 (2.03, 2.50)	2.24 (2.02, 2.49)	1.95 (1.69, 2.26)	2.28 (2.03, 2.57)
Former light (quit 10 years)	1.54 (1.46, 1.63)	1.52 (1.44, 1.60)	1.51 (1.43, 1.60)	1.41 (1.31, 1.52)	1.53 (1.44, 1.62)
Former light (quit 20 years)	1.25 (1.21, 1.28)	1.24 (1.20, 1.27)	1.24 (1.20, 1.27)	1.19 (1.15, 1.24)	1.24 (1.22, 1.35)
Former light (quit 30 years)	1.12 (1.11, 1.14)	1.12 (1.10, 1.13)	1.12 (1.10, 1.13)	1.09 (1.07, 1.12)	1.12 (1.10, 1.14)
Non-smoker	Reference	Reference	Reference	Reference	Reference
Physical activity (METs/day)					
0	1.90 (1.73, 2.10)	1.89 (1.71, 2.08)	1.75 (1.39, 1.67)	1.81 (1.58, 2.07)	1.65 (1.48, 1.84)
1	1.38 (1.31, 1.45)	1.37 (1.31, 1.44)	1.32 (1.18, 1.29)	1.35 (1.26, 1.44)	1.28 (1.22, 1.35)
2	1.14 (1.12, 1.17)	1.14 (1.12, 1.16)	1.12 (1.07, 1.11)	1.13 (1.10, 1.16)	1.11 (1.08, 1.13)
3	Reference	Reference	Reference	Reference	Reference
Diet score					
0	1.55 (1.31, 1.84)	1.50 (1.27, 1.77)	1.55 (1.31, 1.83)	1.75 (1.39, 2.20)	1.49 (1.24, 1.79)
2	1.42 (1.24, 1.63)	1.38 (1.21, 1.45)	1.42 (1.24, 1.62)	1.56 (1.30, 1.88)	1.38 (1.19, 1.60)
4	1.30 (1.18, 1.44)	1.27 (1.15, 1.41)	1.30 (1.18, 1.44)	1.40 (1.22, 1.60)	1.27 (1.14, 1.42)
6	1.19 (1.12, 1.28)	1.18 (1.10, 1.26)	1.19 (1.11, 1.27)	1.25 (1.14, 1.37)	1.17 (1.09, 1.26)
8	1.09 (1.06, 1.13)	1.08 (1.05, 1.12)	1.09 (1.06, 1.13)	1.12 (1.07, 1.17)	1.08 (1.04, 1.12)
10	Reference	Reference	Reference	Reference	Reference
Alcohol					
Heavy drinker	0.98 (0.80, 1.23)	1.00 (0.81, 1.24)	1.08 (0.87, 1.34)	1.37 (1.01, 1.87)	1.09 (0.86, 1.38)
Moderate drinker	0.75 (0.68, 0.83)	0.77 (0.70, 0.85)	0.81 (0.74, 0.91)	0.89 (0.76, 1.03)	0.82 (0.66, 1.02)
Light drinker	Reference	Reference	Reference	Reference	Reference
Neighbourhood deprivation					
High		1.30 (1.15, 1.47)	1.23 (1.09, 1.16)	1.43 (1.16, 1.76)	1.18 (1.03, 1.35)
Moderate		1.07 (0.96, 1.19)	1.05 (0.94, 1.13)	1.29 (1.06, 1.56)	1.01 (0.90, 1.14)
Low		Reference	Reference	Reference	Reference
Education					

< High school		1.10 (1.01, 1.19)	1.07 (0.98, 1.16)	1.07 (0.95, 1.20)	1.14 (1.04, 1.25)
High school graduate		1.02 (0.93, 1.12)	1.03 (0.94, 1.13)	1.06 (0.92, 1.21)	1.04 (0.94, 1.16)
Post-secondary graduate		Reference	Reference	Reference	Reference
Years since immigration					
0 to 15		0.49 (0.27, 0.89)	0.55 (0.30, 0.99)	0.54 (0.22, 1.30)	0.34 (0.27, 1.09)
16 to 30		0.80 (0.60, 1.06)	0.80 (0.60, 1.06)	0.79 (0.50, 1.26)	0.77 (0.55, 1.06)
31 to 45		0.85 (0.70, 1.02)	0.88 (0.73, 1.06)	1.20 (0.90, 1.60)	0.82 (0.66, 1.02)
>45 or born in Canada		Reference	Reference	Reference	Reference
Heart disease					
Yes			1.41 (1.29, 1.53)	1.41 (1.26, 1.58)	1.39 (1.27, 1.53)
No			Reference	Reference	Reference
Suffers from previous stroke					
Yes			1.27 (1.09, 1.47)	1.37 (1.11, 1.67)	1.27 (1.07, 1.50)
No			Reference	Reference	Reference
Cancer					
Yes			2.27** (2.00, 2.58)	2.84** (2.41, 3.35)	1.91** (1.65, 2.22)
No			Reference	Reference	Reference
Diabetes					
Yes			1.55** (1.41, 1.70)	1.64** (1.45, 1.86)	1.53** (1.37, 1.69)
No			Reference	Reference	Reference
Body mass index (kg/m^2)					
45			1.27 (1.04, 1.56)	1.13 (0.84, 1.52)	1.35 (1.09, 1.67)
40			1.13 (1.02, 1.25)	1.06 (0.92, 1.23)	1.16 (1.04, 1.29)
<35			Reference	Reference	Reference
**Model Assessment**					
Discrimination					
C-stat (95% CI)	0.860(0.853-0.868)	0.861 (0.854-0.869)	0.875 (0.868-0.882)		
Ratio 90th : 10th risk percentile	301.85	313.00	401.22		
Calibration					
Subgroups with >20% difference	6 (10.3%)	4 (6.9%)	0 (0.0%)		
Subgroups with >10% difference	9 (15.5%)	7 (12.2%)	0 (0.0%)		
