# Supplementary material for: Measuring Burden of Unhealthy Behaviours Using a Multivariable Predictive Approach: Life Expectancy Lost in Canada Attributable to Smoking, Alcohol, Physical Inactivity, and Diet
Source: PLoS Med. 2016 Aug 16;13(8):e1002082. doi: 10.1371/journal.pmed.1002082 (PMC4986987; doi:10.1371/journal.pmed.1002082)
Supplement: S7 Table — (PDF) [file pmed.1002082.s012.pdf]

S7 Table. MPoRT formula for probability of 1-year survival for use in life tables

MPoRT risk score:

Score(t) = β_{Age_cont}*Age_cont(t) + $\beta_{AgeSpline}$*Age_spline(t) + $\beta_{QSLight\_df}$*QSLight_df (t) + $\beta_{QSHeavy\_df}$*QSHeavy_df (t) + $\beta_{PhysicalActivity\_cont}$*PhysicalActivity_cont + $\beta_{DietScore\_cont}$*DietScore_cont + $\beta_{AlcoholHeavy\_cat}$*AlcoholHeavy_cat + $\beta_{AlcoholMod\_cat}$*AlccoholMod_cat + $\beta_{DepIndHigh\_cat}$*DepIndHigh_cat + $\beta_{DepIndMod\_cat}$*DepIndMod_cat + $\beta_{EduNoGrad\_cat}$*EduNoGrad_cat + $\beta_{EduHSGrad\_cat}$*EduHSGrad_cat + $\beta_{ImEth0To15\_cat}$*ImEth0To15_cat(t) + $\beta_{ImEth16To30\_cat}$*ImEth16To30_cat(t) + $\beta_{ImEth31To45\_cat}$*ImEth31To45_cat(t) + $\beta_{HeartDis\_cat}$*HeartDis_cat + β_{Stroke_cat}*Stroke_cat + β_{BMI_Spline}*BMI_spline + β_{Cancer_cat}*Cancer_cat + $\beta_{Diabetes\_cat}$*Diabetes_cat + $\beta_{CancerAge\_int}$*CancerAge_int(t) + $\beta_{DiabetesAge\_int}$*DiabetesAge_int(t)

Where:

(t) denotes time-varying functions (see time-varying functions below)

MPoRT formula for probability of 1-year survival for use in life tables:

P(1 Year)= $S_t(t|t\text{-}1)$ = 1-H_t= 1- h_{Adj}*exp[Score(t)]

Where:

$\mathbf{h_{Adj}}$ is an adjusted baseline hazard of death specific to the MPoRT 1-year survival formula

Baseline Hazards

Baseline ($h_{t=Year}$)	**Male**	**Female**
h_1	0.0000306623	0.0000148553
h_2	0.0000332830	0.0000202821
h_3	0.0000387073	0.0000232968
h_4	0.0000367520	0.0000254905
h_5	0.0000364115	0.0000226450
h_{Adj}	0.0000372903	0.0000238108

MPoRT Time-Varying Functions

**Function (*t* = year of follow-up)**	**Value**	**Condition**
Age_cont	$\alpha + (t-1)$	-
Age_spline	$\alpha + (t-1) - \gamma$	$\alpha + (t-1) > \gamma$
	0	$\alpha + (t-1) \leq \gamma$
QSLight_df	1	Light smoker
	$e^{\left(-\frac{Q+(t-1)}{\sigma}\right)}$	Former light smoker
	0	Other
QSHeavy_df	1	Heavy Smoker
	$e^{\left(-\frac{Q+(t-1)}{\sigma}\right)}$	Former heavy smoker
	0	Other
ImEth0To15_cat	1	$\alpha + (t-1) \leq 15$
	0	Other
ImEth16To30_cat	1	$15 < \alpha + (t-1) \leq 30$
	0	Other
ImEth31To45_cat	1	$30 < \alpha + (t-1) \leq 45$
	0	Other

α =Baseline Age, γ = (65 for Males; 80 for Females), Q= Baseline Years since Quitting Smoking, σ= (15 for Males; 26 for Females)

Calibration factors for 2010/2011 Canadian population

Age	Male	Female
20	2.354209014	3.823088042
21	2.016337124	3.282910033
22	1.718052312	2.882047074
23	1.734238245	2.903409198
24	1.314500143	2.344531956
25	1.221399533	2.263170315
26	1.356216507	1.961105167
27	1.337225125	1.454895777
28	1.078884372	1.497582481
29	1.48786685	1.314113858
30	1.210963092	1.313472941
31	0.893762494	1.560295841
32	1.128807096	1.411348259
33	0.965129269	1.200454274
34	1.233528483	1.259303358
35	1.057104339	1.234581111
36	1.141099837	1.18886681
37	0.920729249	1.198078321
38	0.986157542	0.978235627
39	0.923450703	0.993915308
40	1.132960673	0.897080474
41	1.042644514	1.068567429
42	0.942454684	1.006285817
43	0.923656492	0.907627453
44	0.954033464	1.078264321
45	0.762469132	1.025634012
46	0.905135485	0.945870642
47	0.900091555	1.002453721
48	0.744641087	0.872867743
49	0.925286005	0.944956195
50	0.900839115	0.923698656
51	0.966926773	0.961721949
52	0.933357972	0.864629676
53	0.866888135	0.894664296
54	0.855435349	0.893975109
55	0.882486099	0.891727876
56	0.873716969	1.027377093
57	0.908533103	0.859818773
58	0.83265157	0.858011333
59	0.817024208	0.885155121
60	0.811131923	0.887804603
61	0.868185865	0.897126062
62	0.787218405	0.878019217
63	0.789532481	0.953011909
64	0.864418667	1.002944465
65	0.888861357	0.975065902
66	0.863587112	1.04221205
67	0.838942833	0.917506697
68	0.850223428	0.939844946
69	0.904198935	0.842562275
70	0.848577116	0.934395808
71	0.929191795	0.939013631
72	0.862528768	0.925404194
73	0.952189763	0.901381861
74	0.902019252	0.984245233
75	0.978043577	0.980214694

76	0.996203403	0.880926139
77	0.975884893	0.924849089
78	1.104238413	0.902701239
79	1.112726158	0.930542259
80	1.120063165	0.877734961
81	1.210026543	0.876297589
82	1.208262637	0.96138406
83	1.125703462	0.903028401
84	1.120008568	0.975210712
85	1.179237993	0.935677643
86	1.194701564	0.946865539
87	1.263346885	1.126947222
88	1.233872864	1.040656686
89	1.247921111	1.063096838
90	1.428112453	0.943679724
91	1.302845767	1.025108406
92	1.262716918	1.057746921
93	0.989538361	1.340560414
94	1.364036488	1.122366943
95	1.36063887	0.977600073
96	1.493154367	1.101023591
97	1.578342601	1.102691585
98	1.574789591	1.232942615
99	1.483284896	1.258405001
