# Supplementary material for: Measuring Burden of Unhealthy Behaviours Using a Multivariable Predictive Approach: Life Expectancy Lost in Canada Attributable to Smoking, Alcohol, Physical Inactivity, and Diet
Source: PLoS Med. 2016 Aug 16;13(8):e1002082. doi: 10.1371/journal.pmed.1002082 (PMC4986987; doi:10.1371/journal.pmed.1002082)
Supplement: S8 Table — (PDF) [file pmed.1002082.s013.pdf]

S8 Table. MPoRT beta coefficients for male and female models

Parameter	Description	Male	Female
Age_cont	Time-varying age (see S11 Table)	0.08314	0.08919
Age_spline	Time-varying age above knot (see S11 Table)	0.03286	0.02922
QSLight_df	Time-varying function for light and former light smokers (see S11 Table)	0.90032	0.80685
QSHeavy_df	Time-varying function for heavy and former heavy smokers (see S11 Table)	1.03950	1.18030
PhysicalActivity_cont	Log transformation of physical activity[¥]	-0.70311	-0.92835
DietScore_cont	Diet score (see S5 Table)	-0.03441	-0.04367
AlcoholHeavy_cat	Heavy drinker (see Table 1)	0.05088	0.07970
AlcoholMod_cat	Moderate drinker (see Table 1)	-0.19270	-0.20063
	Light or non-drinker (see Table 1)	Reference	Reference
DepIndHigh_cat	High neighbourhood deprivation	0.22010	0.20488
DepIndMod_cat	Moderate neighbourhood deprivation	0.06906	0.04659
	Low neighbourhood deprivation	Reference	Reference
EduNoGrad_cat	Less than high school education	0.18571	0.06313
EduHSGrad_cat	High school graduate	0.08632	0.03271
	Post-secondary graduate	Reference	Reference
ImEth0To15_cat	Immigrated to Canada ≤15 years ago	-0.98263	-0.60422
ImEth16To30_cat	Immigrated to Canada 16 to ≤30 years ago	-0.40429	-0.22603
ImEth31To45_cat	Immigrated to Canada 31 to ≤45 years ago	-0.11708	-0.13251
	Non-immigrant or in Canada >45 years	Reference	Reference
HeartDis_cat	Has heart disease	0.37945	0.34187
Stroke_cat	Suffers from a previous stroke	0.22416	0.23591
Cancer_cat	Has cancer	4.40894	4.85672
Diabetes_cat	Has diabetes	1.98474	1.16018
BMI_spline	Units of BMI above 35	0.03048	0.02407
CancerAge_int	Interaction between cancer status and time-varying age	-0.04978	-0.05256
DiabetesAge_int	Interaction between diabetes status and time-varying age	-0.02097	-0.00943

[¥]Daily measures of physical activity exceeding 10 METs were coded as 10 METs before log transformation
