# Supplementary material for: Measuring Burden of Unhealthy Behaviours Using a Multivariable Predictive Approach: Life Expectancy Lost in Canada Attributable to Smoking, Alcohol, Physical Inactivity, and Diet
Source: PLoS Med. 2016 Aug 16;13(8):e1002082. doi: 10.1371/journal.pmed.1002082 (PMC4986987; doi:10.1371/journal.pmed.1002082)
Supplement: S9 Table — (PDF) [file pmed.1002082.s014.pdf]

S9 Table. Comparison of three approaches to calculate the burden of health behaviours

	Multivariable risk approach (This study)	Levin's approach (e.g., Global Burden of Disease)	Cohort approach
**Overall**			
Setting	Countries where population health surveys are available. Over 100 countries.	Worldwide. 200 countries.	Countries where population health surveys are linked to health outcomes (uncommon).
Start with	Exposure (health behaviours)	Outcome (deaths)	Same as multivariable risk approach.
Calculate burden using	Individual data – population sample of people exposed.	Aggregated data – by disease group, age and sex.	Same as multivariable risk approach.
For the time period	Future	Current	Historic
**Relative Risk/Hazards**			
Source	"External" meta-analyses or from "Within" the study cohort.	"External".	"Within".
Exposure levels	Multiple exposure levels, including continuous exposure. ["Within" hazards]	Usually binary or limited number of categories.	Same as multivariable risk approach.
**Exposure**			
Source (for health behaviours)	Population health surveys.	Same as multivariable risk approach.	Same as multivariable risk approach
Correlated risks	Calculated for individual level. Considers health behaviours and sociodemographic risks.	Calculated at the aggregate level.	Same as multivariable risk approach.
**Outcome**			
Source	Predicted. Calibrate to observed data, if needed.	Observed.	Observed.
Burden measures	Total population or for people exposed to risk factors (i.e. life expectancy for people who smoke).	Total population.	Same as multivariable risk approach.
