# Supplementary material for: Measuring Burden of Unhealthy Behaviours Using a Multivariable Predictive Approach: Life Expectancy Lost in Canada Attributable to Smoking, Alcohol, Physical Inactivity, and Diet
Source: PLoS Med. 2016 Aug 16;13(8):e1002082. doi: 10.1371/journal.pmed.1002082 (PMC4986987; doi:10.1371/journal.pmed.1002082)
Supplement: S1 Text — (PDF) [file pmed.1002082.s015.pdf]

S1 Text. Calculation of confidence intervals

We calculated confidence intervals and standard error for predicted mortality using the approach of Kovacevic et al. for out-of-sample prediction with population health surveys.[1] These confidence intervals combined two sources of uncertainty: MPoRT model parameters and exposure variability (CCHS 2012). First, 500 MPoRT models from bootstrap samples of the derivation cohort were created to estimate the MPoRT model uncertainty. The distance between the risk calculated with the models from bootstrap samples and the original model is the estimate for parameter uncertainty. Next, 500 bootstrap samples of the application data were selected. Original MPoRT model was applied to these samples to generate 500 mortality risk estimates. Exposure variability was the difference between bootstrap mortality risks and mortality risk from application data. Following, age-specific variance estimates calculated to account for both parameter and exposure variability as described by Kovacevic et al.

Confidence intervals for life expectancy were calculated using the approach of Chiang and the variances estimates for mortality, as described above. The confidence intervals for cause deleted life expectancy were estimated using a liberal assumption that they were the same variance as the original life expectancy. This is a conservative estimate since cause-deleted life expectancy is based on mortality risk estimates that have less variance than the original estimates. This allowed a straightforward conservative estimate of the confidence intervals for burden estimates (the difference of life expectancy and cause-deleted life expectancy) as $\sqrt{2}$ times the standard error of the life expectancy confidence intervals, given the covariance between the original and cause-deleted estimates is a positive value.

1. Kovacevic M, Mach L, Roberts G, editors. Bootstrap variance estimation for predicted indivdual and population-average risks. Proceedings of the Survey Research Methods Section, American Statistical Association; 2008.
