# Supplementary material for: Measuring Burden of Unhealthy Behaviours Using a Multivariable Predictive Approach: Life Expectancy Lost in Canada Attributable to Smoking, Alcohol, Physical Inactivity, and Diet
Source: PLoS Med. 2016 Aug 16;13(8):e1002082. doi: 10.1371/journal.pmed.1002082 (PMC4986987; doi:10.1371/journal.pmed.1002082)
Supplement: S2 Text — (PDF) [file pmed.1002082.s016.pdf]

**S2 Text.** Predictive Modelling Markup Language (PMML) file

For most current PMML files see: https://github.com/OHRImHealth/predictive-algorithms

**MPoRT male model**


```
<PMML version="1.0" xmlns="http://www.dmg.org/PMML-4_2"
xmlns:xsi="http://www.w3.org/2001/XMLSchema-instance"
xsi:schemaLocation="http://www.dmg.org/PMML-4_2 http://www.dmg.org/v4-
2/pmml-4-2.xsd">
 <Header copyright="Copyright (c) 2016 The Ottawa Hospital"
description="MPoRT Male V1.0.0">
<Annotation>Mortality (all-cause) Population Risk Tool (MPoRT)
  <Extension name="author">Doug Manuel</Extension>
</Annotation>
  <Extension name="user" value=" theottawahospital" extender="COXPH"/>
  <Application name="COXPH" version="1.4"/>
  <Timestamp>2016-05-26 16:56:50</Timestamp>
 </Header>
 <DataDictionary numberOfFields="26">
  <DataField name="survival" optype="continuous" dataType="double"/>
  <DataField name="Age_cont" optype="continuous" dataType="double"/>
  <DataField name="Age_spline" optype="continuous" dataType="double"/>
  <DataField name="QSLight_df" optype="continuous" dataType="double"/>
  <DataField name="QSHeavy_df" optype="continuous" dataType="double"/>
  <DataField name="PhysicalActivity_cont" optype="continuous"
dataType="double"/>
  <DataField name="DietScore_cont" optype="continuous"
dataType="double"/>
  <DataField name="AlcoholHeavy_cat" optype="categorical"
dataType="string"/>
  <DataField name="AlcoholMod_cat" optype="categorical"
dataType="string"/>
  <DataField name="DepIndMod_cat" optype="categorical"
dataType="string"/>
  <DataField name="DepIndHigh_cat" optype="categorical"
dataType="string"/>
  <DataField name="EduNoGrad_cat" optype="categorical"
dataType="string"/>
  <DataField name="EduHSGrad_cat" optype="categorical"
dataType="string"/>
  <DataField name="ImEth0To15_cat" optype="categorical"
dataType="string"/>
  <DataField name="ImEth16To30_cat" optype="categorical"
dataType="string"/>
  <DataField name="ImEth31To45_cat" optype="categorical"
dataType="string"/>
  <DataField name="HeartDis_cat" optype="categorical" dataType="string"/>
  <DataField name="Stroke_cat" optype="categorical" dataType="string"/>
  <DataField name="Cancer_cat" optype="categorical" dataType="string"/>
  <DataField name="Diabetes_cat" optype="categorical" dataType="string"/>
  <DataField name="BMI_spline" optype="continuous" dataType="double"/>
  <DataField name="CancerAge_Int" optype="continuous" dataType="double"/>
  <DataField name="DiabetesAge_Int" optype="continuous"
dataType="double"/>
```

```
  <DataField name="start" optype="continuous" dataType="double"/>
  <DataField name="stop" optype="continuous" dataType="double"/>
  <DataField name="EventDeath" optype="continuous" dataType="double"/>
 </DataDictionary>
<LocalTransformations>
 <DerivedField name="Sex" dataType="double" optype="continuous">
<Apply function="if">
  <Apply function="equal">
    <FieldRef field="sex"/>
    <Constant dataType="string">fem</Constant>
  </Apply>
  <Constant dataType="double">1</Constant>
  <Apply function="if">
    <Apply function="equal">
      <FieldRef field="sex"/>
      <Constant dataType="string">male</Constant>
    </Apply>
    <Constant dataType="double">0</Constant>
  </Apply>
</Apply>
 </DerivedField>
 <DerivedField name="Age_spline" dataType="double" optype="continuous">
<Apply function="if">
  <Apply function="and">
    <Apply function="equal">
      <FieldRef field="Sex"/>
      <Constant dataType="string">fem</Constant>
    </Apply>
    <Apply function="greaterThan">
      <FieldRef field="Age"/>
      <Constant dataType="double">80</Constant>
    </Apply>
  </Apply>
  <Apply function="-">
    <FieldRef field="Age"/>
    <Constant dataType="double">80</Constant>
  </Apply>
  <Apply function="if">
    <Apply function="equal">
      <FieldRef field="Sex"/>
      <Constant dataType="string">fem</Constant>
    </Apply>
    <Constant dataType="double">0</Constant>
    <Apply function="if">
      <Apply function="and">
        <Apply function="equal">
          <FieldRef field="Sex"/>
          <Constant dataType="string">male</Constant>
        </Apply>
        <Apply function="greaterThan">
          <FieldRef field="Age"/>
          <Constant dataType="double">65</Constant>
        </Apply>
      </Apply>
```

```
      <Apply function="-">
        <FieldRef field="Age"/>
        <Constant dataType="double">65</Constant>
      </Apply>
      <Constant dataType="double">0</Constant>
    </Apply>
  </Apply>
</Apply>
 </DerivedField>
 <DerivedField name="EduNoGrad_cat" dataType="double"
optype="continuous">
<Apply function="if">
  <Apply function="and">
    <Apply function="equal">
      <FieldRef field="hs"/>
      <Constant dataType="string">hs2</Constant>
    </Apply>
    <Apply function="equal">
      <FieldRef field="ed"/>
      <Constant dataType="string">ed2</Constant>
    </Apply>
  </Apply>
  <Constant dataType="double">1</Constant>
  <Constant dataType="double">0</Constant>
</Apply>
 </DerivedField>
 <DerivedField name="EduHSGrad_cat" dataType="double"
optype="continuous">
<Apply function="if">
  <Apply function="or">
    <Apply function="and">
      <Apply function="equal">
        <FieldRef field="hs"/>
        <Constant dataType="string">hs1</Constant>
      </Apply>
      <Apply function="equal">
        <FieldRef field="ed"/>
        <Constant dataType="string">ed2</Constant>
      </Apply>
    </Apply>
    <Apply function="and">
      <Apply function="and">
        <Apply function="equal">
          <FieldRef field="hs"/>
          <Constant dataType="string">hs1</Constant>
        </Apply>
        <Apply function="equal">
          <FieldRef field="ed"/>
          <Constant dataType="string">ed1</Constant>
        </Apply>
      </Apply>
      <Apply function="equal">
        <FieldRef field="hdg"/>
        <Constant dataType="string">hdg1</Constant>
```

```
      </Apply>
    </Apply>
  </Apply>
  <Constant dataType="double">1</Constant>
  <Constant dataType="double">0</Constant>
</Apply>
 </DerivedField>
 <DerivedField name="DepIndHigh_cat" dataType="double"
optype="continuous">
<Apply function="if">
  <Apply function="equal">
    <FieldRef field="dep"/>
    <Constant dataType="string">dep1</Constant>
  </Apply>
  <Constant dataType="double">1</Constant>
  <Constant dataType="double">0</Constant>
</Apply>
 </DerivedField>
 <DerivedField name="DepIndMod_cat" dataType="double"
optype="continuous">
<Apply function="if">
  <Apply function="equal">
    <FieldRef field="dep"/>
    <Constant dataType="string">dep2</Constant>
  </Apply>
  <Constant dataType="double">1</Constant>
  <Constant dataType="double">0</Constant>
</Apply>
 </DerivedField>
 <DerivedField name="ImEth0To15_cat" dataType="double"
optype="continuous">
<Apply function="if">
  <Apply function="and">
    <Apply function="and">
      <Apply function="equal">
        <FieldRef field="imm"/>
        <Constant dataType="string">imm2</Constant>
      </Apply>
      <Apply function="greaterOrEqual">
        <FieldRef field="imyr"/>
        <Constant dataType="double">1</Constant>
      </Apply>
    </Apply>
    <Apply function="lessOrEqual">
      <FieldRef field="imyr"/>
      <Constant dataType="double">15</Constant>
    </Apply>
  </Apply>
  <Constant dataType="double">1</Constant>
  <Apply function="if">
    <Apply function="or">
      <Apply function="equal">
        <FieldRef field="imm"/>
        <Constant dataType="string">imm1</Constant>
```

```
      </Apply>
      <Apply function="greaterThan">
        <FieldRef field="imyr"/>
        <Constant dataType="double">15</Constant>
      </Apply>
    </Apply>
    <Constant dataType="double">0</Constant>
    <Constant dataType="double">0</Constant>
  </Apply>
</Apply>
 </DerivedField>
 <DerivedField name="ImEth16To30_cat" dataType="double"
optype="continuous">
<Apply function="if">
  <Apply function="and">
    <Apply function="and">
      <Apply function="equal">
        <FieldRef field="imm"/>
        <Constant dataType="string">imm2</Constant>
      </Apply>
      <Apply function="greaterThan">
        <FieldRef field="imyr"/>
        <Constant dataType="double">15</Constant>
      </Apply>
    </Apply>
    <Apply function="lessOrEqual">
      <FieldRef field="imyr"/>
      <Constant dataType="double">30</Constant>
    </Apply>
  </Apply>
  <Constant dataType="double">1</Constant>
  <Apply function="if">
    <Apply function="or">
      <Apply function="or">
        <Apply function="equal">
          <FieldRef field="imm"/>
          <Constant dataType="string">imm1</Constant>
        </Apply>
        <Apply function="lessOrEqual">
          <FieldRef field="imyr"/>
          <Constant dataType="double">15</Constant>
        </Apply>
      </Apply>
      <Apply function="greaterThan">
        <FieldRef field="imyr"/>
        <Constant dataType="double">30</Constant>
      </Apply>
    </Apply>
    <Constant dataType="double">0</Constant>
    <Constant dataType="double">0</Constant>
  </Apply>
</Apply>
 </DerivedField>
```

```
 <DerivedField name="ImEth31To45_cat" dataType="double"
optype="continuous">
<Apply function="if">
  <Apply function="and">
    <Apply function="and">
      <Apply function="equal">
        <FieldRef field="imm"/>
        <Constant dataType="string">imm2</Constant>
      </Apply>
      <Apply function="greaterThan">
        <FieldRef field="imyr"/>
        <Constant dataType="double">30</Constant>
      </Apply>
    </Apply>
    <Apply function="lessOrEqual">
      <FieldRef field="imyr"/>
      <Constant dataType="double">45</Constant>
    </Apply>
  </Apply>
  <Constant dataType="double">1</Constant>
  <Apply function="if">
    <Apply function="or">
      <Apply function="or">
        <Apply function="equal">
          <FieldRef field="imm"/>
          <Constant dataType="string">imm1</Constant>
        </Apply>
        <Apply function="lessOrEqual">
          <FieldRef field="imyr"/>
          <Constant dataType="double">30</Constant>
        </Apply>
      </Apply>
      <Apply function="greaterThan">
        <FieldRef field="imyr"/>
        <Constant dataType="double">45</Constant>
      </Apply>
    </Apply>
    <Constant dataType="double">0</Constant>
    <Constant dataType="double">0</Constant>
  </Apply>
</Apply>
 </DerivedField>
 <DerivedField name="formerlightflag" dataType="double"
optype="continuous">
<Apply function="if">
  <Apply function="and">
    <Apply function="and">
      <Apply function="equal">
        <FieldRef field="smk"/>
        <Constant dataType="string">smk3</Constant>
      </Apply>
      <Apply function="equal">
        <FieldRef field="evdn"/>
        <Constant dataType="string">evdn1</Constant>
```

```
      </Apply>
    </Apply>
    <Apply function="lessThan">
      <FieldRef field="cigdayf"/>
      <Constant dataType="double">20</Constant>
    </Apply>
  </Apply>
  <Constant dataType="double">1</Constant>
  <Apply function="if">
    <Apply function="and">
      <Apply function="and">
        <Apply function="equal">
          <FieldRef field="smk"/>
          <Constant dataType="string">smk3</Constant>
        </Apply>
        <Apply function="equal">
          <FieldRef field="evdn"/>
          <Constant dataType="string">evdn2</Constant>
        </Apply>
      </Apply>
      <Apply function="equal">
        <FieldRef field="s100"/>
        <Constant dataType="string">s1001</Constant>
      </Apply>
    </Apply>
    <Constant dataType="double">1</Constant>
    <Constant dataType="double">0</Constant>
  </Apply>
</Apply>
 </DerivedField>
 <DerivedField name="formerheavyflag" dataType="double"
optype="continuous">
<Apply function="if">
  <Apply function="and">
    <Apply function="and">
      <Apply function="equal">
        <FieldRef field="smk"/>
        <Constant dataType="string">smk3</Constant>
      </Apply>
      <Apply function="equal">
        <FieldRef field="evdn"/>
        <Constant dataType="string">evdn1</Constant>
      </Apply>
    </Apply>
    <Apply function="greaterOrEqual">
      <FieldRef field="cigdayf"/>
      <Constant dataType="double">20</Constant>
    </Apply>
  </Apply>
  <Constant dataType="double">1</Constant>
  <Constant dataType="double">0</Constant>
</Apply>
 </DerivedField>
 <DerivedField name="quittime" dataType="double" optype="continuous">
```

```
<Apply function="if">
  <Apply function="or">
    <Apply function="equal">
      <FieldRef field="formerlightflag"/>
      <Constant dataType="double">0</Constant>
    </Apply>
    <Apply function="equal">
      <FieldRef field="formerheavyflag"/>
      <Constant dataType="double">0</Constant>
    </Apply>
  </Apply>
  <Constant dataType="double">0</Constant>
  <Apply function="if">
    <Apply function="or">
      <Apply function="equal">
        <FieldRef field="smk"/>
        <Constant dataType="string">smk1</Constant>
      </Apply>
      <Apply function="=">
        <Apply function="and">
          <Apply function="and">
            <Apply function="equal">
              <FieldRef field="smk"/>
              <Constant dataType="string">smk3</Constant>
            </Apply>
            <Apply function="equal">
              <FieldRef field="evdn"/>
              <Constant dataType="string">evdn2</Constant>
            </Apply>
          </Apply>
          <FieldRef field="s100"/>
        </Apply>
        <Constant dataType="string">s1002</Constant>
      </Apply>
    </Apply>
    <Constant dataType="double">0</Constant>
    <Apply function="if">
      <Apply function="equal">
        <FieldRef field="stpn"/>
        <Constant dataType="string">stpn1</Constant>
      </Apply>
      <Constant dataType="double">0</Constant>
      <Apply function="if">
        <Apply function="equal">
          <FieldRef field="stpn"/>
          <Constant dataType="string">stpn2</Constant>
        </Apply>
        <Constant dataType="double">1</Constant>
        <Apply function="if">
          <Apply function="equal">
            <FieldRef field="stpn"/>
            <Constant dataType="string">stpn3</Constant>
          </Apply>
          <Constant dataType="double">2</Constant>
```

```
          &lt;Apply function="if"&gt;
            &lt;Apply function="equal"&gt;
              &lt;FieldRef field="stpn"/&gt;
              &lt;Constant dataType="string"&gt;stpn4&lt;/Constant&gt;
            &lt;/Apply&gt;
            &lt;FieldRef field="stpny"/&gt;
            &lt;FieldRef field="NA"/&gt;
          &lt;/Apply&gt;
        &lt;/Apply&gt;
      &lt;/Apply&gt;
    &lt;/Apply&gt;
  &lt;/Apply&gt;
&lt;/Apply&gt;
 &lt;/DerivedField&gt;
 &lt;DerivedField name="smk_lightraw" dataType="double" optype="continuous"&gt;
&lt;Apply function="if"&gt;
  &lt;Apply function="and"&gt;
    &lt;Apply function="equal"&gt;
      &lt;FieldRef field="smk"/&gt;
      &lt;Constant dataType="string"&gt;smk1&lt;/Constant&gt;
    &lt;/Apply&gt;
    &lt;Apply function="lessThan"&gt;
      &lt;FieldRef field="cigdayd"/&gt;
      &lt;Constant dataType="double"&gt;20&lt;/Constant&gt;
    &lt;/Apply&gt;
  &lt;/Apply&gt;
  &lt;Constant dataType="double"&gt;1&lt;/Constant&gt;
  &lt;Apply function="if"&gt;
    &lt;Apply function="equal"&gt;
      &lt;FieldRef field="smk"/&gt;
      &lt;Constant dataType="string"&gt;smk2&lt;/Constant&gt;
    &lt;/Apply&gt;
    &lt;Constant dataType="double"&gt;1&lt;/Constant&gt;
    &lt;Apply function="if"&gt;
      &lt;Apply function="and"&gt;
        &lt;Apply function="and"&gt;
          &lt;Apply function="equal"&gt;
            &lt;FieldRef field="smk"/&gt;
            &lt;Constant dataType="string"&gt;smk3&lt;/Constant&gt;
          &lt;/Apply&gt;
          &lt;Apply function="equal"&gt;
            &lt;FieldRef field="evdn"/&gt;
            &lt;Constant dataType="string"&gt;evdn1&lt;/Constant&gt;
          &lt;/Apply&gt;
        &lt;/Apply&gt;
        &lt;Apply function="lessThan"&gt;
          &lt;FieldRef field="cigdayf"/&gt;
          &lt;Constant dataType="double"&gt;20&lt;/Constant&gt;
        &lt;/Apply&gt;
      &lt;/Apply&gt;
      &lt;Constant dataType="double"&gt;1&lt;/Constant&gt;
      &lt;Apply function="if"&gt;
        &lt;Apply function="and"&gt;
          &lt;Apply function="and"&gt;
```

```
            <Apply function="equal">
              <FieldRef field="smk"/>
              <Constant dataType="string">smk3</Constant>
            </Apply>
            <Apply function="equal">
              <FieldRef field="evdn"/>
              <Constant dataType="string">evdn2</Constant>
            </Apply>
          </Apply>
          <Apply function="equal">
            <FieldRef field="s100"/>
            <Constant dataType="string">s1001</Constant>
          </Apply>
        </Apply>
        <Constant dataType="double">1</Constant>
        <Constant dataType="double">0</Constant>
      </Apply>
    </Apply>
  </Apply>
</Apply>
 </DerivedField>
 <DerivedField name="smk_heavyraw" dataType="double" optype="continuous">
<Apply function="if">
  <Apply function="and">
    <Apply function="equal">
      <FieldRef field="smk"/>
      <Constant dataType="string">smk1</Constant>
    </Apply>
    <Apply function="greaterOrEqual">
      <FieldRef field="cigdayd"/>
      <Constant dataType="double">20</Constant>
    </Apply>
  </Apply>
  <Constant dataType="double">1</Constant>
  <Apply function="if">
    <Apply function="and">
      <Apply function="and">
        <Apply function="equal">
          <FieldRef field="smk"/>
          <Constant dataType="string">smk3</Constant>
        </Apply>
        <Apply function="equal">
          <FieldRef field="evdn"/>
          <Constant dataType="string">evdn1</Constant>
        </Apply>
      </Apply>
      <Apply function="greaterOrEqual">
        <FieldRef field="cigdayf"/>
        <Constant dataType="double">20</Constant>
      </Apply>
    </Apply>
    <Constant dataType="double">1</Constant>
    <Constant dataType="double">0</Constant>
  </Apply>
```

```
</Apply>
 </DerivedField>
 <DerivedField name="QSLight_df" dataType="double" optype="continuous">
<Apply function="if">
  <Apply function="and">
    <Apply function="equal">
      <FieldRef field="formerlightflag"/>
      <Constant dataType="double">1</Constant>
    </Apply>
    <Apply function="equal">
      <FieldRef field="sex"/>
      <Constant dataType="string">fem</Constant>
    </Apply>
  </Apply>
  <Apply function="exp">
    <Apply function="/">
      <FieldRef field="quittime"/>
      <Constant dataType="double">26</Constant>
    </Apply>
  </Apply>
  <Apply function="if">
    <Apply function="and">
      <Apply function="equal">
        <FieldRef field="formerlightflag"/>
        <Constant dataType="double">1</Constant>
      </Apply>
      <Apply function="equal">
        <FieldRef field="sex"/>
        <Constant dataType="string">male</Constant>
      </Apply>
    </Apply>
    <Apply function="exp">
      <Apply function="/">
        <FieldRef field="quittime"/>
        <Constant dataType="double">15</Constant>
      </Apply>
    </Apply>
    <FieldRef field="smk_lightraw"/>
  </Apply>
</Apply>
 </DerivedField>
 <DerivedField name="QSHeavy_df" dataType="double" optype="continuous">
<Apply function="if">
  <Apply function="and">
    <Apply function="equal">
      <FieldRef field="formerheavyflag"/>
      <Constant dataType="double">1</Constant>
    </Apply>
    <Apply function="equal">
      <FieldRef field="sex"/>
      <Constant dataType="string">fem</Constant>
    </Apply>
  </Apply>
  <Apply function="exp">
```

```
      <Apply function="/">
        <FieldRef field="quittime"/>
        <Constant dataType="double">26</Constant>
      </Apply>
    </Apply>
    <Apply function="if">
      <Apply function="and">
        <Apply function="equal">
          <FieldRef field="formerheavyflag"/>
          <Constant dataType="double">1</Constant>
        </Apply>
        <Apply function="equal">
          <FieldRef field="sex"/>
          <Constant dataType="string">male</Constant>
        </Apply>
      </Apply>
      <Apply function="exp">
        <Apply function="/">
          <FieldRef field="quittime"/>
          <Constant dataType="double">15</Constant>
        </Apply>
      </Apply>
      <FieldRef field="smk_heavyraw"/>
    </Apply>
  </Apply>
   </DerivedField>
   <DerivedField name="walking" dataType="double" optype="continuous">
  <Apply function="if">
    <Apply function="equal">
      <FieldRef field="lpa_lpa1"/>
      <Constant dataType="string">Yes</Constant>
    </Apply>
    <Constant dataType="double">1</Constant>
    <Constant dataType="double">0</Constant>
  </Apply>
   </DerivedField>
   <DerivedField name="walking_t" dataType="double" optype="continuous">
  <Apply function="if">
    <Apply function="is.na">
      <FieldRef field="lpat_lpa1"/>
    </Apply>
    <Constant dataType="double">0</Constant>
    <FieldRef field="lpat_lpa1"/>
  </Apply>
   </DerivedField>
   <DerivedField name="walking_h" dataType="double" optype="continuous">
  <Apply function="if">
    <Apply function="is.na">
      <FieldRef field="lpam_lpa1"/>
    </Apply>
    <Constant dataType="double">0</Constant>
    <Apply function="if">
      <Apply function="equal">
        <FieldRef field="lpam_lpa1"/>
```

```
        <Constant dataType="string">lpa1</Constant>
      </Apply>
      <Constant dataType="double">0.2167</Constant>
      <Apply function="if">
        <Apply function="equal">
          <FieldRef field="lpam_lpa1"/>
          <Constant dataType="string">lpa2</Constant>
        </Apply>
        <Constant dataType="double">0.3833</Constant>
        <Apply function="if">
          <Apply function="equal">
            <FieldRef field="lpam_lpa1"/>
            <Constant dataType="string">lpa3</Constant>
          </Apply>
          <Constant dataType="double">0.75</Constant>
          <Apply function="if">
            <Apply function="equal">
              <FieldRef field="lpam_lpa1"/>
              <Constant dataType="string">lpa4</Constant>
            </Apply>
            <Constant dataType="double">1</Constant>
            <Constant dataType="double">0</Constant>
          </Apply>
        </Apply>
      </Apply>
    </Apply>
  </Apply>
 </DerivedField>
 <DerivedField name="garden" dataType="double" optype="continuous">
<Apply function="if">
  <Apply function="equal">
    <FieldRef field="lpa_lpa2"/>
    <Constant dataType="string">Yes</Constant>
  </Apply>
  <Constant dataType="double">1</Constant>
  <Constant dataType="double">0</Constant>
</Apply>
 </DerivedField>
 <DerivedField name="garden_t" dataType="double" optype="continuous">
<Apply function="if">
  <Apply function="is.na">
    <FieldRef field="lpat_lpa2"/>
  </Apply>
  <Constant dataType="double">0</Constant>
  <FieldRef field="lpat_lpa2"/>
</Apply>
 </DerivedField>
 <DerivedField name="garden_h" dataType="double" optype="continuous">
<Apply function="if">
  <Apply function="is.na">
    <FieldRef field="lpam_lpa2"/>
  </Apply>
  <Constant dataType="double">0</Constant>
  <Apply function="if">
```

```
    <Apply function="equal">
      <FieldRef field="lpam_lpa2"/>
      <Constant dataType="string">lpa15</Constant>
    </Apply>
    <Constant dataType="double">0.2167</Constant>
    <Apply function="if">
      <Apply function="equal">
        <FieldRef field="lpam_lpa2"/>
        <Constant dataType="string">lpa30</Constant>
      </Apply>
      <Constant dataType="double">0.3833</Constant>
      <Apply function="if">
        <Apply function="equal">
          <FieldRef field="lpam_lpa2"/>
          <Constant dataType="string">lpa60</Constant>
        </Apply>
        <Constant dataType="double">0.75</Constant>
        <Apply function="if">
          <Apply function="equal">
            <FieldRef field="lpam_lpa2"/>
            <Constant dataType="string">lpa61</Constant>
          </Apply>
          <Constant dataType="double">1</Constant>
          <Constant dataType="double">0</Constant>
        </Apply>
      </Apply>
    </Apply>
  </Apply>
</Apply>
 </DerivedField>
 <DerivedField name="swim" dataType="double" optype="continuous">
<Apply function="if">
  <Apply function="equal">
    <FieldRef field="lpa_lpa3"/>
    <Constant dataType="string">Yes</Constant>
  </Apply>
  <Constant dataType="double">1</Constant>
  <Constant dataType="double">0</Constant>
</Apply>
 </DerivedField>
 <DerivedField name="swim_t" dataType="double" optype="continuous">
<Apply function="if">
  <Apply function="is.na">
    <FieldRef field="lpat_lpa3"/>
  </Apply>
  <Constant dataType="double">0</Constant>
  <FieldRef field="lpat_lpa3"/>
</Apply>
 </DerivedField>
 <DerivedField name="swim_h" dataType="double" optype="continuous">
<Apply function="if">
  <Apply function="is.na">
    <FieldRef field="lpam_lpa3"/>
  </Apply>
```

```
  <Constant dataType="double">0</Constant>
  <Apply function="if">
    <Apply function="equal">
      <FieldRef field="lpam_lpa3"/>
      <Constant dataType="string">lpa15</Constant>
    </Apply>
    <Constant dataType="double">0.2167</Constant>
    <Apply function="if">
      <Apply function="equal">
        <FieldRef field="lpam_lpa3"/>
        <Constant dataType="string">lpa30</Constant>
      </Apply>
      <Constant dataType="double">0.3833</Constant>
      <Apply function="if">
        <Apply function="equal">
          <FieldRef field="lpam_lpa3"/>
          <Constant dataType="string">lpa60</Constant>
        </Apply>
        <Constant dataType="double">0.75</Constant>
        <Apply function="if">
          <Apply function="equal">
            <FieldRef field="lpam_lpa3"/>
            <Constant dataType="string">lpa61</Constant>
          </Apply>
          <Constant dataType="double">1</Constant>
          <Constant dataType="double">0</Constant>
        </Apply>
      </Apply>
    </Apply>
  </Apply>
</Apply>
 </DerivedField>
 <DerivedField name="bike" dataType="double" optype="continuous">
<Apply function="if">
  <Apply function="equal">
    <FieldRef field="lpa_lpa4"/>
    <Constant dataType="string">Yes</Constant>
  </Apply>
  <Constant dataType="double">1</Constant>
  <Constant dataType="double">0</Constant>
</Apply>
 </DerivedField>
 <DerivedField name="bike_t" dataType="double" optype="continuous">
<Apply function="if">
  <Apply function="is.na">
    <FieldRef field="lpat_lpa4"/>
  </Apply>
  <Constant dataType="double">0</Constant>
  <FieldRef field="lpat_lpa4"/>
</Apply>
 </DerivedField>
 <DerivedField name="bike_h" dataType="double" optype="continuous">
<Apply function="if">
  <Apply function="is.na">
```

```
    &lt;FieldRef field="lpam_lpa4"/&gt;
  &lt;/Apply&gt;
  &lt;Constant dataType="double"&gt;0&lt;/Constant&gt;
  &lt;Apply function="if"&gt;
    &lt;Apply function="equal"&gt;
      &lt;FieldRef field="lpam_lpa4"/&gt;
      &lt;Constant dataType="string"&gt;lpa15&lt;/Constant&gt;
    &lt;/Apply&gt;
    &lt;Constant dataType="double"&gt;0.2167&lt;/Constant&gt;
    &lt;Apply function="if"&gt;
      &lt;Apply function="equal"&gt;
        &lt;FieldRef field="lpam_lpa4"/&gt;
        &lt;Constant dataType="string"&gt;lpa30&lt;/Constant&gt;
      &lt;/Apply&gt;
      &lt;Constant dataType="double"&gt;0.3833&lt;/Constant&gt;
      &lt;Apply function="if"&gt;
        &lt;Apply function="equal"&gt;
          &lt;FieldRef field="lpam_lpa4"/&gt;
          &lt;Constant dataType="string"&gt;lpa60&lt;/Constant&gt;
        &lt;/Apply&gt;
        &lt;Constant dataType="double"&gt;0.75&lt;/Constant&gt;
        &lt;Apply function="if"&gt;
          &lt;Apply function="equal"&gt;
            &lt;FieldRef field="lpam_lpa4"/&gt;
            &lt;Constant dataType="string"&gt;lpa61&lt;/Constant&gt;
          &lt;/Apply&gt;
          &lt;Constant dataType="double"&gt;1&lt;/Constant&gt;
          &lt;Constant dataType="double"&gt;0&lt;/Constant&gt;
        &lt;/Apply&gt;
      &lt;/Apply&gt;
    &lt;/Apply&gt;
  &lt;/Apply&gt;
&lt;/Apply&gt;
 &lt;/DerivedField&gt;
 &lt;DerivedField name="dance" dataType="double" optype="continuous"&gt;
&lt;Apply function="if"&gt;
  &lt;Apply function="equal"&gt;
    &lt;FieldRef field="lpa_lpa5"/&gt;
    &lt;Constant dataType="string"&gt;Yes&lt;/Constant&gt;
  &lt;/Apply&gt;
  &lt;Constant dataType="double"&gt;1&lt;/Constant&gt;
  &lt;Constant dataType="double"&gt;0&lt;/Constant&gt;
&lt;/Apply&gt;
 &lt;/DerivedField&gt;
 &lt;DerivedField name="dance_t" dataType="double" optype="continuous"&gt;
&lt;Apply function="if"&gt;
  &lt;Apply function="is.na"&gt;
    &lt;FieldRef field="lpat_lpa5"/&gt;
  &lt;/Apply&gt;
  &lt;Constant dataType="double"&gt;0&lt;/Constant&gt;
  &lt;FieldRef field="lpat_lpa5"/&gt;
&lt;/Apply&gt;
 &lt;/DerivedField&gt;
 &lt;DerivedField name="dance_h" dataType="double" optype="continuous"&gt;
```

```
<Apply function="if">
  <Apply function="is.na">
    <FieldRef field="lpam_lpa5"/>
  </Apply>
  <Constant dataType="double">0</Constant>
  <Apply function="if">
    <Apply function="equal">
      <FieldRef field="lpam_lpa5"/>
      <Constant dataType="string">lpa15</Constant>
    </Apply>
    <Constant dataType="double">0.2167</Constant>
    <Apply function="if">
      <Apply function="equal">
        <FieldRef field="lpam_lpa5"/>
        <Constant dataType="string">lpa30</Constant>
      </Apply>
      <Constant dataType="double">0.3833</Constant>
      <Apply function="if">
        <Apply function="equal">
          <FieldRef field="lpam_lpa5"/>
          <Constant dataType="string">lpa60</Constant>
        </Apply>
        <Constant dataType="double">0.75</Constant>
        <Apply function="if">
          <Apply function="equal">
            <FieldRef field="lpam_lpa5"/>
            <Constant dataType="string">lpa61</Constant>
          </Apply>
          <Constant dataType="double">1</Constant>
          <Constant dataType="double">0</Constant>
        </Apply>
      </Apply>
    </Apply>
  </Apply>
</Apply>
 </DerivedField>
 <DerivedField name="hexercises" dataType="double" optype="continuous">
<Apply function="if">
  <Apply function="equal">
    <FieldRef field="lpa_lpa6"/>
    <Constant dataType="string">Yes</Constant>
  </Apply>
  <Constant dataType="double">1</Constant>
  <Constant dataType="double">0</Constant>
</Apply>
 </DerivedField>
 <DerivedField name="hexercises_t" dataType="double" optype="continuous">
<Apply function="if">
  <Apply function="is.na">
    <FieldRef field="lpat_lpa6"/>
  </Apply>
  <Constant dataType="double">0</Constant>
  <FieldRef field="lpat_lpa6"/>
</Apply>
```

```
 </DerivedField>
 <DerivedField name="hexercises_h" dataType="double" optype="continuous">
<Apply function="if">
  <Apply function="is.na">
    <FieldRef field="lpam_lpa6"/>
  </Apply>
  <Constant dataType="double">0</Constant>
  <Apply function="if">
    <Apply function="equal">
      <FieldRef field="lpam_lpa6"/>
      <Constant dataType="string">lpa15</Constant>
    </Apply>
    <Constant dataType="double">0.2167</Constant>
    <Apply function="if">
      <Apply function="equal">
        <FieldRef field="lpam_lpa6"/>
        <Constant dataType="string">lpa30</Constant>
      </Apply>
      <Constant dataType="double">0.3833</Constant>
      <Apply function="if">
        <Apply function="equal">
          <FieldRef field="lpam_lpa6"/>
          <Constant dataType="string">lpa60</Constant>
        </Apply>
        <Constant dataType="double">0.75</Constant>
        <Apply function="if">
          <Apply function="equal">
            <FieldRef field="lpam_lpa6"/>
            <Constant dataType="string">lpa61</Constant>
          </Apply>
          <Constant dataType="double">1</Constant>
          <Constant dataType="double">0</Constant>
        </Apply>
      </Apply>
    </Apply>
  </Apply>
</Apply>
 </DerivedField>
 <DerivedField name="hockey" dataType="double" optype="continuous">
<Apply function="if">
  <Apply function="equal">
    <FieldRef field="lpa_lpa7"/>
    <Constant dataType="string">Yes</Constant>
  </Apply>
  <Constant dataType="double">1</Constant>
  <Constant dataType="double">0</Constant>
</Apply>
 </DerivedField>
 <DerivedField name="hockey_t" dataType="double" optype="continuous">
<Apply function="if">
  <Apply function="is.na">
    <FieldRef field="lpat_lpa7"/>
  </Apply>
  <Constant dataType="double">0</Constant>
```

```
  <FieldRef field="lpat_lpa7"/>
</Apply>
 </DerivedField>
 <DerivedField name="hockey_h" dataType="double" optype="continuous">
<Apply function="if">
  <Apply function="is.na">
    <FieldRef field="lpam_lpa7"/>
  </Apply>
  <Constant dataType="double">0</Constant>
  <Apply function="if">
    <Apply function="equal">
      <FieldRef field="lpam_lpa7"/>
      <Constant dataType="string">lpa15</Constant>
    </Apply>
    <Constant dataType="double">0.2167</Constant>
    <Apply function="if">
      <Apply function="equal">
        <FieldRef field="lpam_lpa7"/>
        <Constant dataType="string">lpa30</Constant>
      </Apply>
      <Constant dataType="double">0.3833</Constant>
      <Apply function="if">
        <Apply function="equal">
          <FieldRef field="lpam_lpa7"/>
          <Constant dataType="string">lpa60</Constant>
        </Apply>
        <Constant dataType="double">0.75</Constant>
        <Apply function="if">
          <Apply function="equal">
            <FieldRef field="lpam_lpa7"/>
            <Constant dataType="string">lpa61</Constant>
          </Apply>
          <Constant dataType="double">1</Constant>
          <Constant dataType="double">0</Constant>
        </Apply>
      </Apply>
    </Apply>
  </Apply>
</Apply>
 </DerivedField>
 <DerivedField name="skate" dataType="double" optype="continuous">
<Apply function="if">
  <Apply function="equal">
    <FieldRef field="lpa_lpa8"/>
    <Constant dataType="string">Yes</Constant>
  </Apply>
  <Constant dataType="double">1</Constant>
  <Constant dataType="double">0</Constant>
</Apply>
 </DerivedField>
 <DerivedField name="skate_t" dataType="double" optype="continuous">
<Apply function="if">
  <Apply function="is.na">
    <FieldRef field="lpat_lpa8"/>
```

```
  </Apply>
  <Constant dataType="double">0</Constant>
  <FieldRef field="lpat_lpa8"/>
</Apply>
 </DerivedField>
 <DerivedField name="skate_h" dataType="double" optype="continuous">
<Apply function="if">
  <Apply function="is.na">
    <FieldRef field="lpam_lpa8"/>
  </Apply>
  <Constant dataType="double">0</Constant>
  <Apply function="if">
    <Apply function="equal">
      <FieldRef field="lpam_lpa8"/>
      <Constant dataType="string">lpa15</Constant>
    </Apply>
    <Constant dataType="double">0.2167</Constant>
    <Apply function="if">
      <Apply function="equal">
        <FieldRef field="lpam_lpa8"/>
        <Constant dataType="string">lpa30</Constant>
      </Apply>
      <Constant dataType="double">0.3833</Constant>
      <Apply function="if">
        <Apply function="equal">
          <FieldRef field="lpam_lpa8"/>
          <Constant dataType="string">lpa60</Constant>
        </Apply>
        <Constant dataType="double">0.75</Constant>
        <Apply function="if">
          <Apply function="equal">
            <FieldRef field="lpam_lpa8"/>
            <Constant dataType="string">lpa61</Constant>
          </Apply>
          <Constant dataType="double">1</Constant>
          <Constant dataType="double">0</Constant>
        </Apply>
      </Apply>
    </Apply>
  </Apply>
</Apply>
 </DerivedField>
 <DerivedField name="inline" dataType="double" optype="continuous">
<Apply function="if">
  <Apply function="equal">
    <FieldRef field="lpa_lpa9"/>
    <Constant dataType="string">Yes</Constant>
  </Apply>
  <Constant dataType="double">1</Constant>
  <Constant dataType="double">0</Constant>
</Apply>
 </DerivedField>
 <DerivedField name="inline_t" dataType="double" optype="continuous">
<Apply function="if">
```

```
    <Apply function="is.na">
      <FieldRef field="lpat_lpa9"/>
    </Apply>
    <Constant dataType="double">0</Constant>
    <FieldRef field="lpat_lpa9"/>
  </Apply>
   </DerivedField>
   <DerivedField name="inline_h" dataType="double" optype="continuous">
  <Apply function="if">
    <Apply function="is.na">
      <FieldRef field="lpam_lpa9"/>
    </Apply>
    <Constant dataType="double">0</Constant>
    <Apply function="if">
      <Apply function="equal">
        <FieldRef field="lpam_lpa9"/>
        <Constant dataType="string">lpa15</Constant>
      </Apply>
      <Constant dataType="double">0.2167</Constant>
      <Apply function="if">
        <Apply function="equal">
          <FieldRef field="lpam_lpa9"/>
          <Constant dataType="string">lpa30</Constant>
        </Apply>
        <Constant dataType="double">0.3833</Constant>
        <Apply function="if">
          <Apply function="equal">
            <FieldRef field="lpam_lpa9"/>
            <Constant dataType="string">lpa60</Constant>
          </Apply>
          <Constant dataType="double">0.75</Constant>
          <Apply function="if">
            <Apply function="equal">
              <FieldRef field="lpat_lpa9"/>
              <Constant dataType="string">lpa61</Constant>
            </Apply>
            <Constant dataType="double">1</Constant>
            <Constant dataType="double">0</Constant>
          </Apply>
        </Apply>
      </Apply>
    </Apply>
  </Apply>
   </DerivedField>
   <DerivedField name="jogrun" dataType="double" optype="continuous">
  <Apply function="if">
    <Apply function="equal">
      <FieldRef field="lpa_lpa10"/>
      <Constant dataType="string">Yes</Constant>
    </Apply>
    <Constant dataType="double">1</Constant>
    <Constant dataType="double">0</Constant>
  </Apply>
   </DerivedField>
```

```
 <DerivedField name="jogrun_t" dataType="double" optype="continuous">
<Apply function="if">
  <Apply function="is.na">
    <FieldRef field="lpat_lpa10"/>
  </Apply>
  <Constant dataType="double">0</Constant>
  <FieldRef field="lpat_lpa10"/>
</Apply>
 </DerivedField>
 <DerivedField name="jogrun_h" dataType="double" optype="continuous">
<Apply function="if">
  <Apply function="is.na">
    <FieldRef field="lpat_lpa10"/>
  </Apply>
  <Constant dataType="double">0</Constant>
  <Apply function="if">
    <Apply function="equal">
      <FieldRef field="lpat_lpa10"/>
      <Constant dataType="string">lpa15</Constant>
    </Apply>
    <Constant dataType="double">0.2167</Constant>
    <Apply function="if">
      <Apply function="equal">
        <FieldRef field="lpat_lpa10"/>
        <Constant dataType="string">lpa30</Constant>
      </Apply>
      <Constant dataType="double">0.3833</Constant>
      <Apply function="if">
        <Apply function="equal">
          <FieldRef field="lpat_lpa10"/>
          <Constant dataType="string">lpa60</Constant>
        </Apply>
        <Constant dataType="double">0.75</Constant>
        <Apply function="if">
          <Apply function="equal">
            <FieldRef field="lpat_lpa10"/>
            <Constant dataType="string">lpa61</Constant>
          </Apply>
          <Constant dataType="double">1</Constant>
          <Constant dataType="double">0</Constant>
        </Apply>
      </Apply>
    </Apply>
  </Apply>
</Apply>
 </DerivedField>
 <DerivedField name="golf" dataType="double" optype="continuous">
<Apply function="if">
  <Apply function="equal">
    <FieldRef field="lpa_lpa11"/>
    <Constant dataType="string">Yes</Constant>
  </Apply>
  <Constant dataType="double">1</Constant>
  <Constant dataType="double">0</Constant>
```

```
</Apply>
 </DerivedField>
 <DerivedField name="golf_t" dataType="double" optype="continuous">
<Apply function="if">
  <Apply function="is.na">
    <FieldRef field="lpat_lpa11"/>
  </Apply>
  <Constant dataType="double">0</Constant>
  <FieldRef field="lpat_lpa11"/>
</Apply>
 </DerivedField>
 <DerivedField name="golf_h" dataType="double" optype="continuous">
<Apply function="if">
  <Apply function="is.na">
    <FieldRef field="lpam_lpa11"/>
  </Apply>
  <Constant dataType="double">0</Constant>
  <Apply function="if">
    <Apply function="equal">
      <FieldRef field="lpam_lpa11"/>
      <Constant dataType="string">lpa15</Constant>
    </Apply>
    <Constant dataType="double">0.2167</Constant>
    <Apply function="if">
      <Apply function="equal">
        <FieldRef field="lpam_lpa11"/>
        <Constant dataType="string">lpa30</Constant>
      </Apply>
      <Constant dataType="double">0.3833</Constant>
      <Apply function="if">
        <Apply function="equal">
          <FieldRef field="lpam_lpa11"/>
          <Constant dataType="string">lpa60</Constant>
        </Apply>
        <Constant dataType="double">0.75</Constant>
        <Apply function="if">
          <Apply function="equal">
            <FieldRef field="lpam_lpa11"/>
            <Constant dataType="string">lpa61</Constant>
          </Apply>
          <Constant dataType="double">1</Constant>
          <Constant dataType="double">0</Constant>
        </Apply>
      </Apply>
    </Apply>
  </Apply>
</Apply>
 </DerivedField>
 <DerivedField name="aerobics" dataType="double" optype="continuous">
<Apply function="if">
  <Apply function="equal">
    <FieldRef field="lpa_lpa12"/>
    <Constant dataType="string">Yes</Constant>
  </Apply>
```

```
  &lt;Constant dataType="double"&gt;1&lt;/Constant&gt;
  &lt;Constant dataType="double"&gt;0&lt;/Constant&gt;
&lt;/Apply&gt;
 &lt;/DerivedField&gt;
 &lt;DerivedField name="aerobics_t" dataType="double" optype="continuous"&gt;
&lt;Apply function="if"&gt;
  &lt;Apply function="is.na"&gt;
    &lt;FieldRef field="lpat_lpa12"/&gt;
  &lt;/Apply&gt;
  &lt;Constant dataType="double"&gt;0&lt;/Constant&gt;
  &lt;FieldRef field="lpat_lpa12"/&gt;
&lt;/Apply&gt;
 &lt;/DerivedField&gt;
 &lt;DerivedField name="aerobics_h" dataType="double" optype="continuous"&gt;
&lt;Apply function="if"&gt;
  &lt;Apply function="is.na"&gt;
    &lt;FieldRef field="lpam_lpa12"/&gt;
  &lt;/Apply&gt;
  &lt;Constant dataType="double"&gt;0&lt;/Constant&gt;
  &lt;Apply function="if"&gt;
    &lt;Apply function="equal"&gt;
      &lt;FieldRef field="lpam_lpa12"/&gt;
      &lt;Constant dataType="string"&gt;lpa15&lt;/Constant&gt;
    &lt;/Apply&gt;
    &lt;Constant dataType="double"&gt;0.2167&lt;/Constant&gt;
    &lt;Apply function="if"&gt;
      &lt;Apply function="equal"&gt;
        &lt;FieldRef field="lpam_lpa12"/&gt;
        &lt;Constant dataType="string"&gt;lpa30&lt;/Constant&gt;
      &lt;/Apply&gt;
      &lt;Constant dataType="double"&gt;0.3833&lt;/Constant&gt;
      &lt;Apply function="if"&gt;
        &lt;Apply function="equal"&gt;
          &lt;FieldRef field="lpam_lpa12"/&gt;
          &lt;Constant dataType="string"&gt;lpa60&lt;/Constant&gt;
        &lt;/Apply&gt;
        &lt;Constant dataType="double"&gt;0.75&lt;/Constant&gt;
        &lt;Apply function="if"&gt;
          &lt;Apply function="equal"&gt;
            &lt;FieldRef field="lpam_lpa12"/&gt;
            &lt;Constant dataType="string"&gt;lpa61&lt;/Constant&gt;
          &lt;/Apply&gt;
          &lt;Constant dataType="double"&gt;1&lt;/Constant&gt;
          &lt;Constant dataType="double"&gt;0&lt;/Constant&gt;
        &lt;/Apply&gt;
      &lt;/Apply&gt;
    &lt;/Apply&gt;
  &lt;/Apply&gt;
&lt;/Apply&gt;
 &lt;/DerivedField&gt;
 &lt;DerivedField name="ski" dataType="double" optype="continuous"&gt;
&lt;Apply function="if"&gt;
  &lt;Apply function="equal"&gt;
    &lt;FieldRef field="lpa_lpa13"/&gt;
```

```
    <Constant dataType="string">Yes</Constant>
  </Apply>
  <Constant dataType="double">1</Constant>
  <Constant dataType="double">0</Constant>
</Apply>
 </DerivedField>
 <DerivedField name="ski_t" dataType="double" optype="continuous">
<Apply function="if">
  <Apply function="is.na">
    <FieldRef field="lpat_lpa13"/>
  </Apply>
  <Constant dataType="double">0</Constant>
  <FieldRef field="lpat_lpa13"/>
</Apply>
 </DerivedField>
 <DerivedField name="ski_h" dataType="double" optype="continuous">
<Apply function="if">
  <Apply function="is.na">
    <FieldRef field="lpam_lpa13"/>
  </Apply>
  <Constant dataType="double">0</Constant>
  <Apply function="if">
    <Apply function="equal">
      <FieldRef field="lpam_lpa13"/>
      <Constant dataType="string">lpa15</Constant>
    </Apply>
    <Constant dataType="double">0.2167</Constant>
    <Apply function="if">
      <Apply function="equal">
        <FieldRef field="lpam_lpa13"/>
        <Constant dataType="string">lpa30</Constant>
      </Apply>
      <Constant dataType="double">0.3833</Constant>
      <Apply function="if">
        <Apply function="equal">
          <FieldRef field="lpam_lpa13"/>
          <Constant dataType="string">lpa60</Constant>
        </Apply>
        <Constant dataType="double">0.75</Constant>
        <Apply function="if">
          <Apply function="equal">
            <FieldRef field="lpam_lpa13"/>
            <Constant dataType="string">lpa61</Constant>
          </Apply>
          <Constant dataType="double">1</Constant>
          <Constant dataType="double">0</Constant>
        </Apply>
      </Apply>
    </Apply>
  </Apply>
</Apply>
 </DerivedField>
 <DerivedField name="bowl" dataType="double" optype="continuous">
<Apply function="if">
```

```
  <Apply function="equal">
    <FieldRef field="lpa_lpa14"/>
    <Constant dataType="string">Yes</Constant>
  </Apply>
  <Constant dataType="double">1</Constant>
  <Constant dataType="double">0</Constant>
</Apply>
 </DerivedField>
 <DerivedField name="bowl_t" dataType="double" optype="continuous">
<Apply function="if">
  <Apply function="is.na">
    <FieldRef field="lpat_lpa14"/>
  </Apply>
  <Constant dataType="double">0</Constant>
  <FieldRef field="lpat_lpa14"/>
</Apply>
 </DerivedField>
 <DerivedField name="bowl_h" dataType="double" optype="continuous">
<Apply function="if">
  <Apply function="is.na">
    <FieldRef field="lpam_lpa14"/>
  </Apply>
  <Constant dataType="double">0</Constant>
  <Apply function="if">
    <Apply function="equal">
      <FieldRef field="lpam_lpa14"/>
      <Constant dataType="string">lpa15</Constant>
    </Apply>
    <Constant dataType="double">0.2167</Constant>
    <Apply function="if">
      <Apply function="equal">
        <FieldRef field="lpam_lpa14"/>
        <Constant dataType="string">lpa30</Constant>
      </Apply>
      <Constant dataType="double">0.3833</Constant>
      <Apply function="if">
        <Apply function="equal">
          <FieldRef field="lpam_lpa14"/>
          <Constant dataType="string">lpa60</Constant>
        </Apply>
        <Constant dataType="double">0.75</Constant>
        <Apply function="if">
          <Apply function="equal">
            <FieldRef field="lpam_lpa14"/>
            <Constant dataType="string">lpa61</Constant>
          </Apply>
          <Constant dataType="double">1</Constant>
          <Constant dataType="double">0</Constant>
        </Apply>
      </Apply>
    </Apply>
  </Apply>
</Apply>
 </DerivedField>
```

```
 <DerivedField name="baseball" dataType="double" optype="continuous">
<Apply function="if">
  <Apply function="equal">
    <FieldRef field="lpa_lpa15"/>
    <Constant dataType="string">Yes</Constant>
  </Apply>
  <Constant dataType="double">1</Constant>
  <Constant dataType="double">0</Constant>
</Apply>
 </DerivedField>
 <DerivedField name="baseball_t" dataType="double" optype="continuous">
<Apply function="if">
  <Apply function="is.na">
    <FieldRef field="lpat_lpa15"/>
  </Apply>
  <Constant dataType="double">0</Constant>
  <FieldRef field="lpat_lpa15"/>
</Apply>
 </DerivedField>
 <DerivedField name="baseball_h" dataType="double" optype="continuous">
<Apply function="if">
  <Apply function="is.na">
    <FieldRef field="lpam_lpa15"/>
  </Apply>
  <Constant dataType="double">0</Constant>
  <Apply function="if">
    <Apply function="equal">
      <FieldRef field="lpam_lpa15"/>
      <Constant dataType="string">lpa15</Constant>
    </Apply>
    <Constant dataType="double">0.2167</Constant>
    <Apply function="if">
      <Apply function="equal">
        <FieldRef field="lpam_lpa15"/>
        <Constant dataType="string">lpa30</Constant>
      </Apply>
      <Constant dataType="double">0.3833</Constant>
      <Apply function="if">
        <Apply function="equal">
          <FieldRef field="lpam_lpa15"/>
          <Constant dataType="string">lpa60</Constant>
        </Apply>
        <Constant dataType="double">0.75</Constant>
        <Apply function="if">
          <Apply function="equal">
            <FieldRef field="lpam_lpa15"/>
            <Constant dataType="string">lpa61</Constant>
          </Apply>
          <Constant dataType="double">1</Constant>
          <Constant dataType="double">0</Constant>
        </Apply>
      </Apply>
    </Apply>
  </Apply>
```

```
</Apply>
 </DerivedField>
 <DerivedField name="tennis" dataType="double" optype="continuous">
<Apply function="if">
  <Apply function="equal">
    <FieldRef field="lpa_lpa16"/>
    <Constant dataType="string">Yes</Constant>
  </Apply>
  <Constant dataType="double">1</Constant>
  <Constant dataType="double">0</Constant>
</Apply>
 </DerivedField>
 <DerivedField name="tennis_t" dataType="double" optype="continuous">
<Apply function="if">
  <Apply function="is.na">
    <FieldRef field="lpat_lpa16"/>
  </Apply>
  <Constant dataType="double">0</Constant>
  <FieldRef field="lpat_lpa16"/>
</Apply>
 </DerivedField>
 <DerivedField name="tennis_h" dataType="double" optype="continuous">
<Apply function="if">
  <Apply function="is.na">
    <FieldRef field="lpam_lpa16"/>
  </Apply>
  <Constant dataType="double">0</Constant>
  <Apply function="if">
    <Apply function="equal">
      <FieldRef field="lpam_lpa16"/>
      <Constant dataType="string">lpa15</Constant>
    </Apply>
    <Constant dataType="double">0.2167</Constant>
    <Apply function="if">
      <Apply function="equal">
        <FieldRef field="lpam_lpa16"/>
        <Constant dataType="string">lpa30</Constant>
      </Apply>
      <Constant dataType="double">0.3833</Constant>
      <Apply function="if">
        <Apply function="equal">
          <FieldRef field="lpam_lpa16"/>
          <Constant dataType="string">lpa60</Constant>
        </Apply>
        <Constant dataType="double">0.75</Constant>
        <Apply function="if">
          <Apply function="equal">
            <FieldRef field="lpam_lpa16"/>
            <Constant dataType="string">lpa61</Constant>
          </Apply>
          <Constant dataType="double">1</Constant>
          <Constant dataType="double">0</Constant>
        </Apply>
      </Apply>
```

```
    </Apply>
  </Apply>
</Apply>
 </DerivedField>
 <DerivedField name="weights" dataType="double" optype="continuous">
<Apply function="if">
  <Apply function="equal">
    <FieldRef field="lpa_lpa17"/>
    <Constant dataType="string">Yes</Constant>
  </Apply>
  <Constant dataType="double">1</Constant>
  <Constant dataType="double">0</Constant>
</Apply>
 </DerivedField>
 <DerivedField name="weights_t" dataType="double" optype="continuous">
<Apply function="if">
  <Apply function="is.na">
    <FieldRef field="lpat_lpa17"/>
  </Apply>
  <Constant dataType="double">0</Constant>
  <FieldRef field="lpat_lpa17"/>
</Apply>
 </DerivedField>
 <DerivedField name="weights_h" dataType="double" optype="continuous">
<Apply function="if">
  <Apply function="is.na">
    <FieldRef field="lpam_lpa17"/>
  </Apply>
  <Constant dataType="double">0</Constant>
  <Apply function="if">
    <Apply function="equal">
      <FieldRef field="lpam_lpa17"/>
      <Constant dataType="string">lpa15</Constant>
    </Apply>
    <Constant dataType="double">0.2167</Constant>
    <Apply function="if">
      <Apply function="equal">
        <FieldRef field="lpam_lpa17"/>
        <Constant dataType="string">lpa30</Constant>
      </Apply>
      <Constant dataType="double">0.3833</Constant>
      <Apply function="if">
        <Apply function="equal">
          <FieldRef field="lpam_lpa17"/>
          <Constant dataType="string">lpa60</Constant>
        </Apply>
        <Constant dataType="double">0.75</Constant>
        <Apply function="if">
          <Apply function="equal">
            <FieldRef field="lpam_lpa17"/>
            <Constant dataType="string">lpa61</Constant>
          </Apply>
          <Constant dataType="double">1</Constant>
          <Constant dataType="double">0</Constant>
```

```
        </Apply>
      </Apply>
    </Apply>
  </Apply>
</Apply>
 </DerivedField>
 <DerivedField name="fishing" dataType="double" optype="continuous">
<Apply function="if">
  <Apply function="equal">
    <FieldRef field="lpa_lpa18"/>
    <Constant dataType="string">Yes</Constant>
  </Apply>
  <Constant dataType="double">1</Constant>
  <Constant dataType="double">0</Constant>
</Apply>
 </DerivedField>
 <DerivedField name="fishing_t" dataType="double" optype="continuous">
<Apply function="if">
  <Apply function="is.na">
    <FieldRef field="lpat_lpa18"/>
  </Apply>
  <Constant dataType="double">0</Constant>
  <FieldRef field="lpat_lpa18"/>
</Apply>
 </DerivedField>
 <DerivedField name="fishing_h" dataType="double" optype="continuous">
<Apply function="if">
  <Apply function="is.na">
    <FieldRef field="lpam_lpa18"/>
  </Apply>
  <Constant dataType="double">0</Constant>
  <Apply function="if">
    <Apply function="equal">
      <FieldRef field="lpam_lpa18"/>
      <Constant dataType="string">lpa15</Constant>
    </Apply>
    <Constant dataType="double">0.2167</Constant>
    <Apply function="if">
      <Apply function="equal">
        <FieldRef field="lpam_lpa18"/>
        <Constant dataType="string">lpa30</Constant>
      </Apply>
      <Constant dataType="double">0.3833</Constant>
      <Apply function="if">
        <Apply function="equal">
          <FieldRef field="lpam_lpa18"/>
          <Constant dataType="string">lpa60</Constant>
        </Apply>
        <Constant dataType="double">0.75</Constant>
        <Apply function="if">
          <Apply function="equal">
            <FieldRef field="lpam_lpa18"/>
            <Constant dataType="string">lpa61</Constant>
          </Apply>
```

```
          <Constant dataType="double">1</Constant>
          <Constant dataType="double">0</Constant>
        </Apply>
      </Apply>
    </Apply>
  </Apply>
</Apply>
 </DerivedField>
 <DerivedField name="volleyball" dataType="double" optype="continuous">
<Apply function="if">
  <Apply function="equal">
    <FieldRef field="lpa_lpa19"/>
    <Constant dataType="string">Yes</Constant>
  </Apply>
  <Constant dataType="double">1</Constant>
  <Constant dataType="double">0</Constant>
</Apply>
 </DerivedField>
 <DerivedField name="volleyball_t" dataType="double" optype="continuous">
<Apply function="if">
  <Apply function="is.na">
    <FieldRef field="lpat_lpa19"/>
  </Apply>
  <Constant dataType="double">0</Constant>
  <FieldRef field="lpat_lpa19"/>
</Apply>
 </DerivedField>
 <DerivedField name="volleyball_h" dataType="double" optype="continuous">
<Apply function="if">
  <Apply function="is.na">
    <FieldRef field="lpam_lpa19"/>
  </Apply>
  <Constant dataType="double">0</Constant>
  <Apply function="if">
    <Apply function="equal">
      <FieldRef field="lpam_lpa19"/>
      <Constant dataType="string">lpa15</Constant>
    </Apply>
    <Constant dataType="double">0.2167</Constant>
    <Apply function="if">
      <Apply function="equal">
        <FieldRef field="lpam_lpa19"/>
        <Constant dataType="string">lpa30</Constant>
      </Apply>
      <Constant dataType="double">0.3833</Constant>
      <Apply function="if">
        <Apply function="equal">
          <FieldRef field="lpam_lpa19"/>
          <Constant dataType="string">lpa60</Constant>
        </Apply>
        <Constant dataType="double">0.75</Constant>
        <Apply function="if">
          <Apply function="equal">
            <FieldRef field="lpam_lpa19"/>
```

```
            <Constant dataType="string">lpa61</Constant>
          </Apply>
          <Constant dataType="double">1</Constant>
          <Constant dataType="double">0</Constant>
        </Apply>
      </Apply>
    </Apply>
  </Apply>
</Apply>
 </DerivedField>
 <DerivedField name="basketball" dataType="double" optype="continuous">
<Apply function="if">
  <Apply function="equal">
    <FieldRef field="lpa_lpa20"/>
    <Constant dataType="string">Yes</Constant>
  </Apply>
  <Constant dataType="double">1</Constant>
  <Constant dataType="double">0</Constant>
</Apply>
 </DerivedField>
 <DerivedField name="basketball_t" dataType="double" optype="continuous">
<Apply function="if">
  <Apply function="is.na">
    <FieldRef field="lpat_lpa20"/>
  </Apply>
  <Constant dataType="double">0</Constant>
  <FieldRef field="lpat_lpa20"/>
</Apply>
 </DerivedField>
 <DerivedField name="basketball_h" dataType="double" optype="continuous">
<Apply function="if">
  <Apply function="is.na">
    <FieldRef field="lpam_lpa20"/>
  </Apply>
  <Constant dataType="double">0</Constant>
  <Apply function="if">
    <Apply function="equal">
      <FieldRef field="lpam_lpa20"/>
      <Constant dataType="string">lpa15</Constant>
    </Apply>
    <Constant dataType="double">0.2167</Constant>
    <Apply function="if">
      <Apply function="equal">
        <FieldRef field="lpam_lpa20"/>
        <Constant dataType="string">lpa30</Constant>
      </Apply>
      <Constant dataType="double">0.3833</Constant>
      <Apply function="if">
        <Apply function="equal">
          <FieldRef field="lpam_lpa20"/>
          <Constant dataType="string">lpa60</Constant>
        </Apply>
        <Constant dataType="double">0.75</Constant>
        <Apply function="if">
```

```
          <Apply function="equal">
            <FieldRef field="lpam_lpa20"/>
            <Constant dataType="string">lpa61</Constant>
          </Apply>
          <Constant dataType="double">1</Constant>
          <Constant dataType="double">0</Constant>
        </Apply>
      </Apply>
    </Apply>
  </Apply>
</Apply>
 </DerivedField>
 <DerivedField name="soccer" dataType="double" optype="continuous">
<Apply function="if">
  <Apply function="equal">
    <FieldRef field="lpa_lpa21"/>
    <Constant dataType="string">Yes</Constant>
  </Apply>
  <Constant dataType="double">1</Constant>
  <Constant dataType="double">0</Constant>
</Apply>
 </DerivedField>
 <DerivedField name="soccer_t" dataType="double" optype="continuous">
<Apply function="if">
  <Apply function="is.na">
    <FieldRef field="lpat_lpa21"/>
  </Apply>
  <Constant dataType="double">0</Constant>
  <FieldRef field="lpat_lpa21"/>
</Apply>
 </DerivedField>
 <DerivedField name="soccer_h" dataType="double" optype="continuous">
<Apply function="if">
  <Apply function="is.na">
    <FieldRef field="lpam_lpa21"/>
  </Apply>
  <Constant dataType="double">0</Constant>
  <Apply function="if">
    <Apply function="equal">
      <FieldRef field="lpam_lpa21"/>
      <Constant dataType="string">lpa15</Constant>
    </Apply>
    <Constant dataType="double">0.2167</Constant>
    <Apply function="if">
      <Apply function="equal">
        <FieldRef field="lpam_lpa21"/>
        <Constant dataType="string">lpa30</Constant>
      </Apply>
      <Constant dataType="double">0.3833</Constant>
      <Apply function="if">
        <Apply function="equal">
          <FieldRef field="lpam_lpa21"/>
          <Constant dataType="string">lpa60</Constant>
        </Apply>
```

```
        <Constant dataType="double">0.75</Constant>
        <Apply function="if">
          <Apply function="equal">
            <FieldRef field="lpam_lpa21"/>
            <Constant dataType="string">lpa61</Constant>
          </Apply>
          <Constant dataType="double">1</Constant>
          <Constant dataType="double">0</Constant>
        </Apply>
      </Apply>
    </Apply>
  </Apply>
</Apply>
 </DerivedField>
 <DerivedField name="other" dataType="double" optype="continuous">
<Apply function="if">
  <Apply function="equal">
    <FieldRef field="lpa_lpa22"/>
    <Constant dataType="string">Yes</Constant>
  </Apply>
  <Constant dataType="double">1</Constant>
  <Constant dataType="double">0</Constant>
</Apply>
 </DerivedField>
 <DerivedField name="other_t" dataType="double" optype="continuous">
<Apply function="if">
  <Apply function="is.na">
    <FieldRef field="lpat_lpa22"/>
  </Apply>
  <Constant dataType="double">0</Constant>
  <FieldRef field="lpat_lpa22"/>
</Apply>
 </DerivedField>
 <DerivedField name="other_h" dataType="double" optype="continuous">
<Apply function="if">
  <Apply function="is.na">
    <FieldRef field="lpam_lpa22"/>
  </Apply>
  <Constant dataType="double">0</Constant>
  <Apply function="if">
    <Apply function="equal">
      <FieldRef field="lpam_lpa22"/>
      <Constant dataType="string">lpa15</Constant>
    </Apply>
    <Constant dataType="double">0.2167</Constant>
    <Apply function="if">
      <Apply function="equal">
        <FieldRef field="lpat_lpa22"/>
        <Constant dataType="string">lpa30</Constant>
      </Apply>
      <Constant dataType="double">0.3833</Constant>
      <Apply function="if">
        <Apply function="equal">
          <FieldRef field="lpam_lpa22"/>
```

```
          <Constant dataType="string">lpa60</Constant>
        </Apply>
        <Constant dataType="double">0.75</Constant>
        <Apply function="if">
          <Apply function="equal">
            <FieldRef field="lpam_lpa22"/>
            <Constant dataType="string">lpa61</Constant>
          </Apply>
          <Constant dataType="double">1</Constant>
          <Constant dataType="double">0</Constant>
        </Apply>
      </Apply>
    </Apply>
  </Apply>
</Apply>
 </DerivedField>
 <DerivedField name="PhysicalActivityraw1" dataType="double"
optype="continuous">
<Apply function="+">
  <Apply function="+">
    <Apply function="+">
      <Apply function="+">
        <Apply function="+">
          <Apply function="+">
            <Apply function="+">
              <Apply function="+">
                <Apply function="+">
                  <Apply function="+">
                    <Apply function="+">
                      <Apply function="+">
                        <Apply function="+">
                          <Apply function="+">
                            <Apply function="+">
                              <Apply function="+">
                                <Apply function="+">
                                  <Apply function="+">
                                    <Apply function="+">
                                      <Apply function="+">
                                        <Apply function="+">
                                          <Apply function="/">
                                            <Apply function="*">
                                              <Apply function="*">
                                                <Apply function="*">
                                                  <FieldRef
field="walking"/>

                                                  <FieldRef
field="walking_h"/>

                                                </Apply>
                                                <Constant
dataType="double">3</Constant>

                                              </Apply>
                                              <FieldRef
field="walking_t"/>

                                            </Apply>
```

```
                                              <Constant
dataType="double">90</Constant>
                                            </Apply>
                                            <Apply function="/">
                                              <Apply function="*">
                                                <Apply function="*">
                                                  <Apply function="*">
                                                    <FieldRef
field="garden"/>
                                                    <FieldRef
field="garden_h"/>
                                                  </Apply>
                                                  <Constant
dataType="double">3</Constant>
                                                </Apply>
                                                <FieldRef
field="garden_t"/>
                                              </Apply>
                                              <Constant
dataType="double">90</Constant>
                                            </Apply>
                                          </Apply>
                                          <Apply function="/">
                                            <Apply function="*">
                                              <Apply function="*">
                                                <Apply function="*">
                                                  <FieldRef field="swim"/>
                                                  <FieldRef
field="swim_h"/>
                                                </Apply>
                                                <Constant
dataType="double">3</Constant>
                                              </Apply>
                                              <FieldRef field="swim_t"/>
                                            </Apply>
                                            <Constant
dataType="double">90</Constant>
                                          </Apply>
                                        </Apply>
                                        <Apply function="/">
                                          <Apply function="*">
                                            <Apply function="*">
                                              <Apply function="*">
                                                <FieldRef field="bike"/>
                                                <FieldRef field="bike_h"/>
                                              </Apply>
                                              <Constant
dataType="double">4</Constant>
                                            </Apply>
                                            <FieldRef field="bike_t"/>
                                          </Apply>
                                          <Constant
dataType="double">90</Constant>
                                        </Apply>
```

```
                                    </Apply>
                                    <Apply function="/">
                                      <Apply function="*">
                                        <Apply function="*">
                                          <Apply function="*">
                                            <FieldRef field="dance"/>
                                            <FieldRef field="dance_h"/>
                                          </Apply>
                                          <Constant
dataType="double">3</Constant>
                                        </Apply>
                                        <FieldRef field="dance_t"/>
                                      </Apply>
                                      <Constant
dataType="double">90</Constant>
                                    </Apply>
                                  </Apply>
                                  <Apply function="/">
                                    <Apply function="*">
                                      <Apply function="*">
                                        <Apply function="*">
                                          <FieldRef field="hexercises"/>
                                          <FieldRef
field="hexercises_h"/>
                                        </Apply>
                                        <Constant
dataType="double">3</Constant>
                                      </Apply>
                                      <FieldRef field="hexercises_t"/>
                                    </Apply>
                                    <Constant
dataType="double">90</Constant>
                                  </Apply>
                                </Apply>
                                <Apply function="/">
                                  <Apply function="*">
                                    <Apply function="*">
                                      <Apply function="*">
                                        <FieldRef field="hockey"/>
                                        <FieldRef field="hockey_h"/>
                                      </Apply>
                                      <Constant
dataType="double">6</Constant>
                                    </Apply>
                                    <FieldRef field="hockey_t"/>
                                  </Apply>
                                  <Constant
dataType="double">90</Constant>
                                </Apply>
                              </Apply>
                              <Apply function="/">
                                <Apply function="*">
                                  <Apply function="*">
                                    <Apply function="*">
```

```
                                      <FieldRef field="skate"/>
                                      <FieldRef field="skate_h"/>
                                    </Apply>
                                    <Constant
dataType="double">4</Constant>
                                  </Apply>
                                  <FieldRef field="skate_t"/>
                                </Apply>
                                <Constant dataType="double">90</Constant>
                              </Apply>
                            </Apply>
                            <Apply function="/">
                              <Apply function="*">
                                <Apply function="*">
                                  <Apply function="*">
                                    <FieldRef field="inline"/>
                                    <FieldRef field="inline_h"/>
                                  </Apply>
                                  <Constant
dataType="double">5</Constant>
                                </Apply>
                                <FieldRef field="inline_t"/>
                              </Apply>
                              <Constant dataType="double">90</Constant>
                            </Apply>
                          </Apply>
                          <Apply function="/">
                            <Apply function="*">
                              <Apply function="*">
                                <Apply function="*">
                                  <FieldRef field="jogrun"/>
                                  <FieldRef field="jogrun_h"/>
                                </Apply>
                                <Constant
dataType="double">9.5</Constant>
                              </Apply>
                              <FieldRef field="jogrun_t"/>
                            </Apply>
                            <Constant dataType="double">90</Constant>
                          </Apply>
                        </Apply>
                        <Apply function="/">
                          <Apply function="*">
                            <Apply function="*">
                              <Apply function="*">
                                <FieldRef field="golf"/>
                                <FieldRef field="golf_h"/>
                              </Apply>
                              <Constant dataType="double">4</Constant>
                            </Apply>
                            <FieldRef field="golf_t"/>
                          </Apply>
                          <Constant dataType="double">90</Constant>
                        </Apply>
```

```
      </Apply>
      <Apply function="/">
        <Apply function="*">
          <Apply function="*">
            <Apply function="*">
              <FieldRef field="aerobics"/>
              <FieldRef field="aerobics_h"/>
            </Apply>
            <Constant dataType="double">4</Constant>
          </Apply>
          <FieldRef field="aerobics_t"/>
        </Apply>
        <Constant dataType="double">90</Constant>
      </Apply>
    </Apply>
    <Apply function="/">
      <Apply function="*">
        <Apply function="*">
          <Apply function="*">
            <FieldRef field="ski"/>
            <FieldRef field="ski_h"/>
          </Apply>
          <Constant dataType="double">4</Constant>
        </Apply>
        <FieldRef field="ski_t"/>
      </Apply>
      <Constant dataType="double">90</Constant>
    </Apply>
  </Apply>
  <Apply function="/">
    <Apply function="*">
      <Apply function="*">
        <Apply function="*">
          <FieldRef field="bowl"/>
          <FieldRef field="bowl_h"/>
        </Apply>
        <Constant dataType="double">2</Constant>
      </Apply>
      <FieldRef field="bowl_t"/>
    </Apply>
    <Constant dataType="double">90</Constant>
  </Apply>
</Apply>
<Apply function="/">
  <Apply function="*">
    <Apply function="*">
      <Apply function="*">
        <FieldRef field="baseball"/>
        <FieldRef field="baseball_h"/>
      </Apply>
      <Constant dataType="double">3</Constant>
    </Apply>
    <FieldRef field="baseball_t"/>
  </Apply>
```

```
          <Constant dataType="double">90</Constant>
        </Apply>
      </Apply>
      <Apply function="/">
        <Apply function="*">
          <Apply function="*">
            <Apply function="*">
              <FieldRef field="tennis"/>
              <FieldRef field="tennis_h"/>
            </Apply>
            <Constant dataType="double">4</Constant>
          </Apply>
          <FieldRef field="tennis_t"/>
        </Apply>
        <Constant dataType="double">90</Constant>
      </Apply>
    </Apply>
    <Apply function="/">
      <Apply function="*">
        <Apply function="*">
          <Apply function="*">
            <FieldRef field="weights"/>
            <FieldRef field="weights_h"/>
          </Apply>
          <Constant dataType="double">3</Constant>
        </Apply>
        <FieldRef field="weights_t"/>
      </Apply>
      <Constant dataType="double">90</Constant>
    </Apply>
  </Apply>
  <Apply function="/">
    <Apply function="*">
      <Apply function="*">
        <Apply function="*">
          <FieldRef field="fishing"/>
          <FieldRef field="fishing_h"/>
        </Apply>
        <Constant dataType="double">3</Constant>
      </Apply>
      <FieldRef field="fishing_t"/>
    </Apply>
    <Constant dataType="double">90</Constant>
  </Apply>
</Apply>
<Apply function="/">
  <Apply function="*">
    <Apply function="*">
      <Apply function="*">
        <FieldRef field="volleyball"/>
        <FieldRef field="volleyball_h"/>
      </Apply>
      <Constant dataType="double">5</Constant>
    </Apply>
```

```
            <FieldRef field="volleyball_t"/>
          </Apply>
          <Constant dataType="double">90</Constant>
        </Apply>
      </Apply>
      <Apply function="/">
        <Apply function="*">
          <Apply function="*">
            <Apply function="*">
              <FieldRef field="basketball"/>
              <FieldRef field="basketball_h"/>
            </Apply>
            <Constant dataType="double">6</Constant>
          </Apply>
          <FieldRef field="basketball_t"/>
        </Apply>
        <Constant dataType="double">90</Constant>
      </Apply>
    </Apply>
    <Apply function="/">
      <Apply function="*">
        <Apply function="*">
          <Apply function="*">
            <FieldRef field="soccer"/>
            <FieldRef field="soccer_h"/>
          </Apply>
          <Constant dataType="double">5</Constant>
        </Apply>
        <FieldRef field="soccer_t"/>
      </Apply>
      <Constant dataType="double">90</Constant>
    </Apply>
  </Apply>
  <Apply function="/">
    <Apply function="*">
      <Apply function="*">
        <Apply function="*">
          <FieldRef field="other"/>
          <FieldRef field="other_h"/>
        </Apply>
        <Constant dataType="double">4</Constant>
      </Apply>
      <FieldRef field="other_t"/>
    </Apply>
    <Constant dataType="double">90</Constant>
  </Apply>
</Apply>
 </DerivedField>
 <DerivedField name="PhysicalActivityraw2" dataType="double"
optype="continuous">
<Apply function="if">
  <Apply function="greaterThan">
    <FieldRef field="PhysicalActivityraw1"/>
    <Constant dataType="double">10</Constant>
```

```
  </Apply>
  <Constant dataType="double">10</Constant>
  <FieldRef field="PhysicalActivityraw1"/>
</Apply>
 </DerivedField>
 <DerivedField name="PhysicalActivity_cont" dataType="double"
optype="continuous">
<Apply function="ln">
  <FieldRef field="PhysicalActivityraw2"/>
</Apply>
 </DerivedField>
 <DerivedField name="weeklyalc" dataType="double" optype="continuous">
<Apply function="if">
  <Apply function="equal">
    <FieldRef field="dev"/>
    <Constant dataType="string">dev2</Constant>
  </Apply>
  <FieldRef field="NA"/>
  <Apply function="if">
    <Apply function="and">
      <Apply function="equal">
        <FieldRef field="dev"/>
        <Constant dataType="string">dev1</Constant>
      </Apply>
      <Apply function="equal">
        <FieldRef field="dany"/>
        <Constant dataType="string">dany1</Constant>
      </Apply>
    </Apply>
    <Apply function="+">
      <Apply function="+">
        <Apply function="+">
          <Apply function="+">
            <Apply function="+">
              <Apply function="+">
                <FieldRef field="drk_drkm"/>
                <FieldRef field="drk_drkt"/>
              </Apply>
              <FieldRef field="drk_drkw"/>
            </Apply>
            <FieldRef field="drk_drkr"/>
          </Apply>
          <FieldRef field="drk_drkf"/>
        </Apply>
        <FieldRef field="drk_drksa"/>
      </Apply>
      <FieldRef field="drk_drksu"/>
    </Apply>
    <Constant dataType="double">0</Constant>
  </Apply>
</Apply>
 </DerivedField>
 <DerivedField name="bingeflag" dataType="double" optype="continuous">
<Apply function="if">
```

```
<Apply function="equal">
  <FieldRef field="dev"/>
  <Constant dataType="string">dev2</Constant>
</Apply>
<FieldRef field="NA"/>
<Apply function="if">
  <Apply function="and">
    <Apply function="not">
      <Apply function="is.na">
        <FieldRef field="db"/>
      </Apply>
    </Apply>
    <Apply function="or">
      <Apply function="equal">
        <FieldRef field="db"/>
        <Constant dataType="string">db5</Constant>
      </Apply>
      <Apply function="equal">
        <FieldRef field="db"/>
        <Constant dataType="string">db6</Constant>
      </Apply>
    </Apply>
  </Apply>
  <Constant dataType="double">1</Constant>
  <Apply function="if">
    <Apply function="and">
      <Apply function="not">
        <Apply function="is.na">
          <FieldRef field="drk_drkm"/>
        </Apply>
      </Apply>
      <Apply function="greaterOrEqual">
        <FieldRef field="drk_drkm"/>
        <Constant dataType="double">5</Constant>
      </Apply>
    </Apply>
    <Constant dataType="double">1</Constant>
    <Apply function="if">
      <Apply function="and">
        <Apply function="not">
          <Apply function="is.na">
            <FieldRef field="drk_drkt"/>
          </Apply>
        </Apply>
        <FieldRef field="drk_drkt"/>
      </Apply>
      <Constant dataType="double">1</Constant>
      <Apply function="if">
        <Apply function="and">
          <Apply function="not">
            <Apply function="is.na">
              <FieldRef field="drk_drkw"/>
            </Apply>
          </Apply>
```

```
  <Apply function="greaterOrEqual">
    <FieldRef field="drk_drkw"/>
    <Constant dataType="double">5</Constant>
  </Apply>
</Apply>
<Constant dataType="double">1</Constant>
<Apply function="if">
  <Apply function="and">
    <Apply function="not">
      <Apply function="is.na">
        <FieldRef field="drk_drkr"/>
      </Apply>
    </Apply>
    <Apply function="greaterOrEqual">
      <FieldRef field="drk_drkr"/>
      <Constant dataType="double">5</Constant>
    </Apply>
  </Apply>
  <Constant dataType="double">1</Constant>
  <Apply function="if">
    <Apply function="and">
      <Apply function="not">
        <Apply function="is.na">
          <FieldRef field="drk_drkf"/>
        </Apply>
      </Apply>
      <Apply function="greaterOrEqual">
        <FieldRef field="drk_drkf"/>
        <Constant dataType="double">5</Constant>
      </Apply>
    </Apply>
    <Constant dataType="double">1</Constant>
    <Apply function="if">
      <Apply function="and">
        <Apply function="not">
          <Apply function="is.na">
            <FieldRef field="drk_drksa"/>
          </Apply>
        </Apply>
        <Apply function="greaterOrEqual">
          <FieldRef field="drk_drksa"/>
          <Constant dataType="double">5</Constant>
        </Apply>
      </Apply>
      <Constant dataType="double">1</Constant>
      <Apply function="if">
        <Apply function="and">
          <Apply function="not">
            <Apply function="is.na">
              <FieldRef field="drk_drksu"/>
            </Apply>
          </Apply>
          <Apply function="greaterOrEqual">
            <FieldRef field="drk_drksu"/>
```

```
                      <Constant dataType="double">5</Constant>
                    </Apply>
                  </Apply>
                  <Constant dataType="double">1</Constant>
                  <FieldRef field="NA"/>
                </Apply>
              </Apply>
            </Apply>
          </Apply>
        </Apply>
      </Apply>
    </Apply>
  </Apply>
</Apply>
 </DerivedField>
 <DerivedField name="AlcoholMod_cat" dataType="double"
optype="continuous">
<Apply function="if">
  <Apply function="and">
    <Apply function="not">
      <Apply function="is.na">
        <FieldRef field="bingeflag"/>
      </Apply>
    </Apply>
    <Apply function="equal">
      <FieldRef field="bingeflag"/>
      <Constant dataType="double">1</Constant>
    </Apply>
  </Apply>
  <Constant dataType="double">0</Constant>
  <Apply function="if">
    <Apply function="and">
      <Apply function="and">
        <Apply function="and">
          <Apply function="and">
            <Apply function="equal">
              <FieldRef field="Sex"/>
              <Constant dataType="double">0</Constant>
            </Apply>
            <Apply function="equal">
              <FieldRef field="dev"/>
              <Constant dataType="string">dev1</Constant>
            </Apply>
          </Apply>
          <Apply function="equal">
            <FieldRef field="dany"/>
            <Constant dataType="string">dany1</Constant>
          </Apply>
        </Apply>
        <Apply function="greaterThan">
          <FieldRef field="weeklyalc"/>
          <Constant dataType="double">3</Constant>
        </Apply>
      </Apply>
```

```
    <Apply function="lessOrEqual">
      <FieldRef field="weeklyalc"/>
      <Constant dataType="double">21</Constant>
    </Apply>
  </Apply>
  <Constant dataType="double">1</Constant>
  <Apply function="if">
    <Apply function="and">
      <Apply function="and">
        <Apply function="and">
          <Apply function="and">
            <Apply function="equal">
              <FieldRef field="Sex"/>
              <Constant dataType="double">1</Constant>
            </Apply>
            <Apply function="equal">
              <FieldRef field="dev"/>
              <Constant dataType="string">dev1</Constant>
            </Apply>
          </Apply>
          <Apply function="equal">
            <FieldRef field="dany"/>
            <Constant dataType="string">dany1</Constant>
          </Apply>
        </Apply>
        <Apply function="greaterThan">
          <FieldRef field="weeklyalc"/>
          <Constant dataType="double">2</Constant>
        </Apply>
      </Apply>
      <Apply function="lessOrEqual">
        <FieldRef field="weeklyalc"/>
        <Constant dataType="double">14</Constant>
      </Apply>
    </Apply>
    <Constant dataType="double">1</Constant>
    <Apply function="if">
      <Apply function="and">
        <Apply function="and">
          <Apply function="and">
            <Apply function="equal">
              <FieldRef field="Sex"/>
              <Constant dataType="double">0</Constant>
            </Apply>
            <Apply function="equal">
              <FieldRef field="dev"/>
              <Constant dataType="string">dev1</Constant>
            </Apply>
          </Apply>
          <Apply function="equal">
            <FieldRef field="dany"/>
            <Constant dataType="string">dany1</Constant>
          </Apply>
        </Apply>
```

```
    <Apply function="lessOrEqual">
      <FieldRef field="weeklyalc"/>
      <Constant dataType="double">3</Constant>
    </Apply>
  </Apply>
  <Constant dataType="double">0</Constant>
  <Apply function="if">
    <Apply function="and">
      <Apply function="and">
        <Apply function="and">
          <Apply function="equal">
            <FieldRef field="Sex"/>
            <Constant dataType="double">1</Constant>
          </Apply>
          <Apply function="equal">
            <FieldRef field="dev"/>
            <Constant dataType="string">dev1</Constant>
          </Apply>
        </Apply>
        <Apply function="equal">
          <FieldRef field="dany"/>
          <Constant dataType="string">dany1</Constant>
        </Apply>
      </Apply>
      <Apply function="lessOrEqual">
        <FieldRef field="weeklyalc"/>
        <Constant dataType="double">2</Constant>
      </Apply>
    </Apply>
    <Constant dataType="double">0</Constant>
    <Apply function="if">
      <Apply function="and">
        <Apply function="and">
          <Apply function="and">
            <Apply function="equal">
              <FieldRef field="Sex"/>
              <Constant dataType="double">0</Constant>
            </Apply>
            <Apply function="equal">
              <FieldRef field="dev"/>
              <Constant dataType="string">dev1</Constant>
            </Apply>
          </Apply>
          <Apply function="equal">
            <FieldRef field="dany"/>
            <Constant dataType="string">dany1</Constant>
          </Apply>
        </Apply>
        <Apply function="greaterThan">
          <FieldRef field="weeklyalc"/>
          <Constant dataType="double">21</Constant>
        </Apply>
      </Apply>
      <Constant dataType="double">0</Constant>
```

```
            <Apply function="if">
              <Apply function="and">
                <Apply function="and">
                  <Apply function="and">
                    <Apply function="equal">
                      <FieldRef field="Sex"/>
                      <Constant dataType="double">1</Constant>
                    </Apply>
                    <Apply function="equal">
                      <FieldRef field="dev"/>
                      <Constant dataType="string">dev1</Constant>
                    </Apply>
                  </Apply>
                  <Apply function="equal">
                    <FieldRef field="dany"/>
                    <Constant dataType="string">dany1</Constant>
                  </Apply>
                </Apply>
                <Apply function="greaterThan">
                  <FieldRef field="weeklyalc"/>
                  <Constant dataType="double">14</Constant>
                </Apply>
              </Apply>
              <Constant dataType="double">0</Constant>
              <Apply function="if">
                <Apply function="or">
                  <Apply function="equal">
                    <FieldRef field="dev"/>
                    <Constant dataType="string">dev2</Constant>
                  </Apply>
                  <Apply function="equal">
                    <FieldRef field="dany"/>
                    <Constant dataType="string">dany2</Constant>
                  </Apply>
                </Apply>
                <Constant dataType="double">0</Constant>
                <FieldRef field="NA"/>
              </Apply>
            </Apply>
          </Apply>
        </Apply>
      </Apply>
    </Apply>
  </Apply>
</Apply>
 </DerivedField>
 <DerivedField name="AlcoholHeavy_cat" dataType="double"
optype="continuous">
<Apply function="if">
  <Apply function="or">
    <Apply function="and">
      <Apply function="not">
        <Apply function="is.na">
          <FieldRef field="bingeflag"/>
```

```
        </Apply>
      </Apply>
      <Apply function="equal">
        <FieldRef field="bingeflag"/>
        <Constant dataType="double">1</Constant>
      </Apply>
    </Apply>
    <Apply function="and">
      <Apply function="and">
        <Apply function="and">
          <Apply function="equal">
            <FieldRef field="Sex"/>
            <Constant dataType="double">0</Constant>
          </Apply>
          <Apply function="equal">
            <FieldRef field="dev"/>
            <Constant dataType="string">dev1</Constant>
          </Apply>
        </Apply>
        <Apply function="equal">
          <FieldRef field="dany"/>
          <Constant dataType="string">dany1</Constant>
        </Apply>
      </Apply>
      <Apply function="greaterThan">
        <FieldRef field="weeklyalc"/>
        <Constant dataType="double">21</Constant>
      </Apply>
    </Apply>
  </Apply>
  <Constant dataType="double">1</Constant>
  <Apply function="if">
    <Apply function="and">
      <Apply function="and">
        <Apply function="and">
          <Apply function="equal">
            <FieldRef field="Sex"/>
            <Constant dataType="double">1</Constant>
          </Apply>
          <Apply function="equal">
            <FieldRef field="dev"/>
            <Constant dataType="string">dev1</Constant>
          </Apply>
        </Apply>
        <Apply function="equal">
          <FieldRef field="dany"/>
          <Constant dataType="string">dany1</Constant>
        </Apply>
      </Apply>
      <Apply function="greaterThan">
        <FieldRef field="weeklyalc"/>
        <Constant dataType="double">14</Constant>
      </Apply>
    </Apply>
```

```
<Constant dataType="double">1</Constant>
<Apply function="if">
  <Apply function="and">
    <Apply function="and">
      <Apply function="and">
        <Apply function="equal">
          <FieldRef field="Sex"/>
          <Constant dataType="double">0</Constant>
        </Apply>
        <Apply function="equal">
          <FieldRef field="dev"/>
          <Constant dataType="string">dev1</Constant>
        </Apply>
      </Apply>
      <Apply function="equal">
        <FieldRef field="dany"/>
        <Constant dataType="string">dany1</Constant>
      </Apply>
    </Apply>
    <Apply function="lessOrEqual">
      <FieldRef field="weeklyalc"/>
      <Constant dataType="double">21</Constant>
    </Apply>
  </Apply>
  <Constant dataType="double">0</Constant>
  <Apply function="if">
    <Apply function="and">
      <Apply function="and">
        <Apply function="and">
          <Apply function="equal">
            <FieldRef field="Sex"/>
            <Constant dataType="double">1</Constant>
          </Apply>
          <Apply function="equal">
            <FieldRef field="dev"/>
            <Constant dataType="string">dev1</Constant>
          </Apply>
        </Apply>
        <Apply function="equal">
          <FieldRef field="dany"/>
          <Constant dataType="string">dany1</Constant>
        </Apply>
      </Apply>
      <Apply function="lessOrEqual">
        <FieldRef field="weeklyalc"/>
        <Constant dataType="double">14</Constant>
      </Apply>
    </Apply>
    <Constant dataType="double">0</Constant>
    <Apply function="if">
      <Apply function="or">
        <Apply function="equal">
          <FieldRef field="dev"/>
          <Constant dataType="string">dev2</Constant>
```

```
            </Apply>
            <Apply function="equal">
              <FieldRef field="dany"/>
              <Constant dataType="string">dany2</Constant>
            </Apply>
          </Apply>
          <Constant dataType="double">0</Constant>
          <FieldRef field="NA"/>
        </Apply>
      </Apply>
    </Apply>
  </Apply>
</Apply>
 </DerivedField>
 <DerivedField name="djuice" dataType="double" optype="continuous">
<Apply function="if">
  <Apply function="equal">
    <FieldRef field="juiu"/>
    <Constant dataType="string">juid</Constant>
  </Apply>
  <FieldRef field="jui"/>
  <Apply function="if">
    <Apply function="equal">
      <FieldRef field="juiu"/>
      <Constant dataType="string">juiw</Constant>
    </Apply>
    <Apply function="/">
      <FieldRef field="jui"/>
      <Constant dataType="double">7</Constant>
    </Apply>
    <Apply function="if">
      <Apply function="equal">
        <FieldRef field="juiu"/>
        <Constant dataType="string">juim</Constant>
      </Apply>
      <Apply function="/">
        <FieldRef field="jui"/>
        <Constant dataType="double">30</Constant>
      </Apply>
      <Apply function="if">
        <Apply function="equal">
          <FieldRef field="juiu"/>
          <Constant dataType="string">juiy</Constant>
        </Apply>
        <Apply function="/">
          <FieldRef field="jui"/>
          <Constant dataType="double">365</Constant>
        </Apply>
        <Constant dataType="double">0</Constant>
      </Apply>
    </Apply>
  </Apply>
</Apply>
 </DerivedField>
```

```
 <DerivedField name="dfruit" dataType="double" optype="continuous">
<Apply function="if">
  <Apply function="equal">
    <FieldRef field="frtu"/>
    <Constant dataType="string">frtd</Constant>
  </Apply>
  <FieldRef field="frt"/>
  <Apply function="if">
    <Apply function="equal">
      <FieldRef field="frtu"/>
      <Constant dataType="string">frtw</Constant>
    </Apply>
    <Apply function="/">
      <FieldRef field="frt"/>
      <Constant dataType="double">7</Constant>
    </Apply>
    <Apply function="if">
      <Apply function="equal">
        <FieldRef field="frtu"/>
        <Constant dataType="string">frtm</Constant>
      </Apply>
      <Apply function="/">
        <FieldRef field="frt"/>
        <Constant dataType="double">30</Constant>
      </Apply>
      <Apply function="if">
        <Apply function="equal">
          <FieldRef field="frtu"/>
          <Constant dataType="string">frty</Constant>
        </Apply>
        <Apply function="/">
          <FieldRef field="frt"/>
          <Constant dataType="double">365</Constant>
        </Apply>
        <Constant dataType="double">0</Constant>
      </Apply>
    </Apply>
  </Apply>
</Apply>
 </DerivedField>
 <DerivedField name="dsalad" dataType="double" optype="continuous">
<Apply function="if">
  <Apply function="equal">
    <FieldRef field="salu"/>
    <Constant dataType="string">sald</Constant>
  </Apply>
  <FieldRef field="sal"/>
  <Apply function="if">
    <Apply function="equal">
      <FieldRef field="salu"/>
      <Constant dataType="string">salw</Constant>
    </Apply>
    <Apply function="/">
      <FieldRef field="sal"/>
```

```
        <Constant dataType="double">7</Constant>
      </Apply>
      <Apply function="if">
        <Apply function="equal">
          <FieldRef field="salu"/>
          <Constant dataType="string">salm</Constant>
        </Apply>
        <Apply function="/">
          <FieldRef field="sal"/>
          <Constant dataType="double">30</Constant>
        </Apply>
        <Apply function="if">
          <Apply function="equal">
            <FieldRef field="salu"/>
            <Constant dataType="string">saly</Constant>
          </Apply>
          <Apply function="/">
            <FieldRef field="sal"/>
            <Constant dataType="double">365</Constant>
          </Apply>
          <Constant dataType="double">0</Constant>
        </Apply>
      </Apply>
    </Apply>
  </Apply>
 </DerivedField>
 <DerivedField name="dpotato" dataType="double" optype="continuous">
  <Apply function="if">
    <Apply function="equal">
      <FieldRef field="potu"/>
      <Constant dataType="string">potd</Constant>
    </Apply>
    <FieldRef field="pot"/>
    <Apply function="if">
      <Apply function="equal">
        <FieldRef field="potu"/>
        <Constant dataType="string">potw</Constant>
      </Apply>
      <Apply function="/">
        <FieldRef field="pot"/>
        <Constant dataType="double">7</Constant>
      </Apply>
      <Apply function="if">
        <Apply function="equal">
          <FieldRef field="potu"/>
          <Constant dataType="string">potm</Constant>
        </Apply>
        <Apply function="/">
          <FieldRef field="pot"/>
          <Constant dataType="double">30</Constant>
        </Apply>
        <Apply function="if">
          <Apply function="equal">
            <FieldRef field="potu"/>
```

```
          <Constant dataType="string">poty</Constant>
        </Apply>
        <Apply function="/">
          <FieldRef field="pot"/>
          <Constant dataType="double">365</Constant>
        </Apply>
        <Constant dataType="double">0</Constant>
      </Apply>
    </Apply>
  </Apply>
</Apply>
 </DerivedField>
 <DerivedField name="dcarrot" dataType="double" optype="continuous">
<Apply function="if">
  <Apply function="equal">
    <FieldRef field="caru"/>
    <Constant dataType="string">card</Constant>
  </Apply>
  <FieldRef field="car"/>
  <Apply function="if">
    <Apply function="equal">
      <FieldRef field="caru"/>
      <Constant dataType="string">carw</Constant>
    </Apply>
    <Apply function="/">
      <FieldRef field="car"/>
      <Constant dataType="double">7</Constant>
    </Apply>
    <Apply function="if">
      <Apply function="equal">
        <FieldRef field="caru"/>
        <Constant dataType="string">carm</Constant>
      </Apply>
      <Apply function="/">
        <FieldRef field="car"/>
        <Constant dataType="double">30</Constant>
      </Apply>
      <Apply function="if">
        <Apply function="equal">
          <FieldRef field="caru"/>
          <Constant dataType="string">cary</Constant>
        </Apply>
        <Apply function="/">
          <FieldRef field="car"/>
          <Constant dataType="double">365</Constant>
        </Apply>
        <Constant dataType="double">0</Constant>
      </Apply>
    </Apply>
  </Apply>
</Apply>
 </DerivedField>
 <DerivedField name="dveg" dataType="double" optype="continuous">
<Apply function="if">
```

```
  <Apply function="equal">
    <FieldRef field="vegu"/>
    <Constant dataType="string">vegd</Constant>
  </Apply>
  <FieldRef field="veg"/>
  <Apply function="if">
    <Apply function="equal">
      <FieldRef field="vegu"/>
      <Constant dataType="string">vegw</Constant>
    </Apply>
    <Apply function="/">
      <FieldRef field="veg"/>
      <Constant dataType="double">7</Constant>
    </Apply>
    <Apply function="if">
      <Apply function="equal">
        <FieldRef field="vegu"/>
        <Constant dataType="string">vegm</Constant>
      </Apply>
      <Apply function="/">
        <FieldRef field="veg"/>
        <Constant dataType="double">30</Constant>
      </Apply>
      <Apply function="if">
        <Apply function="equal">
          <FieldRef field="vegu"/>
          <Constant dataType="string">vegy</Constant>
        </Apply>
        <Apply function="/">
          <FieldRef field="veg"/>
          <Constant dataType="double">365</Constant>
        </Apply>
        <Constant dataType="double">0</Constant>
      </Apply>
    </Apply>
  </Apply>
</Apply>
 </DerivedField>
 <DerivedField name="fruitnvegraw" dataType="double" optype="continuous">
<Apply function="+">
  <Apply function="+">
    <Apply function="+">
      <Apply function="+">
        <FieldRef field="dfruit"/>
        <FieldRef field="dsalad"/>
      </Apply>
      <FieldRef field="dpotato"/>
    </Apply>
    <FieldRef field="dcarrot"/>
  </Apply>
  <FieldRef field="dveg"/>
</Apply>
 </DerivedField>
 <DerivedField name="fruitnveg" dataType="double" optype="continuous">
```

```
<Apply function="if">
  <Apply function="and">
    <Apply function="greaterThan">
      <FieldRef field="fruitnvegraw"/>
      <Constant dataType="double">8</Constant>
    </Apply>
    <Apply function="lessThan">
      <FieldRef field="fruitnvegraw"/>
      <Constant dataType="double">98</Constant>
    </Apply>
  </Apply>
  <Constant dataType="double">8</Constant>
  <FieldRef field="fruitnvegraw"/>
</Apply>
 </DerivedField>
 <DerivedField name="nocarrotflag" dataType="double" optype="continuous">
<Apply function="if">
  <Apply function="equal">
    <Apply function="*">
      <FieldRef field="dcarrot"/>
      <Constant dataType="double">7</Constant>
    </Apply>
    <Constant dataType="double">0</Constant>
  </Apply>
  <Constant dataType="double">1</Constant>
  <Apply function="if">
    <Apply function="greaterOrEqual">
      <Apply function="*">
        <FieldRef field="dcarrot"/>
        <Constant dataType="double">7</Constant>
      </Apply>
      <Constant dataType="double">1</Constant>
    </Apply>
    <Constant dataType="double">0</Constant>
    <Constant dataType="double">0</Constant>
  </Apply>
</Apply>
 </DerivedField>
 <DerivedField name="highpotatoflag" dataType="double"
optype="continuous">
<Apply function="if">
  <Apply function="and">
    <Apply function="equal">
      <FieldRef field="Sex"/>
      <Constant dataType="double">0</Constant>
    </Apply>
    <Apply function="greaterOrEqual">
      <Apply function="*">
        <FieldRef field="dpotato"/>
        <Constant dataType="double">7</Constant>
      </Apply>
      <Constant dataType="double">7</Constant>
    </Apply>
  </Apply>
```

```
  <Constant dataType="double">1</Constant>
  <Apply function="if">
    <Apply function="and">
      <Apply function="equal">
        <FieldRef field="Sex"/>
        <Constant dataType="double">0</Constant>
      </Apply>
      <Apply function="greaterOrEqual">
        <Apply function="*">
          <FieldRef field="dpotato"/>
          <Constant dataType="double">7</Constant>
        </Apply>
        <Constant dataType="double">5</Constant>
      </Apply>
    </Apply>
    <Constant dataType="double">1</Constant>
    <Constant dataType="double">0</Constant>
  </Apply>
</Apply>
 </DerivedField>
 <DerivedField name="highjuice" dataType="double" optype="continuous">
<Apply function="if">
  <Apply function="equal">
    <FieldRef field="djuice"/>
    <Constant dataType="double">2</Constant>
  </Apply>
  <Constant dataType="double">1</Constant>
  <Apply function="if">
    <Apply function="equal">
      <FieldRef field="djuice"/>
      <Constant dataType="double">3</Constant>
    </Apply>
    <Constant dataType="double">2</Constant>
    <Apply function="if">
      <Apply function="equal">
        <FieldRef field="djuice"/>
        <Constant dataType="double">4</Constant>
      </Apply>
      <Constant dataType="double">3</Constant>
      <Apply function="if">
        <Apply function="equal">
          <FieldRef field="djuice"/>
          <Constant dataType="double">5</Constant>
        </Apply>
        <Constant dataType="double">4</Constant>
        <Apply function="if">
          <Apply function="equal">
            <FieldRef field="djuice"/>
            <Constant dataType="double">5</Constant>
          </Apply>
          <Constant dataType="double">4</Constant>
          <Apply function="if">
            <Apply function="equal">
              <FieldRef field="djuice"/>
```

```
            <Constant dataType="double">6</Constant>
          </Apply>
          <Constant dataType="double">5</Constant>
          <Apply function="if">
            <Apply function="equal">
              <FieldRef field="djuice"/>
              <Constant dataType="double">1</Constant>
            </Apply>
            <Constant dataType="double">0</Constant>
            <Apply function="if">
              <Apply function="equal">
                <FieldRef field="djuice"/>
                <Constant dataType="double">0</Constant>
              </Apply>
              <Constant dataType="double">0</Constant>
              <Constant dataType="double">0</Constant>
            </Apply>
          </Apply>
        </Apply>
      </Apply>
    </Apply>
  </Apply>
 </Apply>
</Apply>
 </DerivedField>
 <DerivedField name="dietraw" dataType="double" optype="continuous">
<Apply function="-">
  <Apply function="-">
    <Apply function="-">
      <FieldRef field="fruitnveg"/>
      <Apply function="*">
        <Constant dataType="double">2</Constant>
        <FieldRef field="highpotatoflag"/>
      </Apply>
    </Apply>
    <Apply function="*">
      <Constant dataType="double">2</Constant>
      <FieldRef field="nocarrotflag"/>
    </Apply>
  </Apply>
  <Apply function="*">
    <Constant dataType="double">2</Constant>
    <FieldRef field="highjuice"/>
  </Apply>
</Apply>
 </DerivedField>
 <DerivedField name="DietScore_cont" dataType="double"
optype="continuous">
<Apply function="if">
  <Apply function="lessThan">
    <FieldRef field="dietraw"/>
    <Constant dataType="double">0</Constant>
  </Apply>
  <Constant dataType="double">0</Constant>
```

```
  <Apply function="if">
    <Apply function="greaterThan">
      <FieldRef field="dietraw"/>
      <Constant dataType="double">10</Constant>
    </Apply>
    <Constant dataType="double">10</Constant>
    <FieldRef field="dietraw"/>
  </Apply>
</Apply>
 </DerivedField>
 <DerivedField name="HeartDis_cat" dataType="double" optype="continuous">
<Apply function="if">
  <Apply function="equal">
    <FieldRef field="hd"/>
    <Constant dataType="string">hd1</Constant>
  </Apply>
  <Constant dataType="double">1</Constant>
  <Apply function="if">
    <Apply function="equal">
      <FieldRef field="hd"/>
      <Constant dataType="string">hd2</Constant>
    </Apply>
    <Constant dataType="double">0</Constant>
    <FieldRef field="NA"/>
  </Apply>
</Apply>
 </DerivedField>
 <DerivedField name="Diabetes_cat" dataType="double" optype="continuous">
<Apply function="if">
  <Apply function="equal">
    <FieldRef field="diab"/>
    <Constant dataType="string">diab1</Constant>
  </Apply>
  <Constant dataType="double">1</Constant>
  <Apply function="if">
    <Apply function="equal">
      <FieldRef field="diab"/>
      <Constant dataType="string">diab2</Constant>
    </Apply>
    <Constant dataType="double">0</Constant>
    <FieldRef field="NA"/>
  </Apply>
</Apply>
 </DerivedField>
 <DerivedField name="Stroke_cat" dataType="double" optype="continuous">
<Apply function="if">
  <Apply function="equal">
    <FieldRef field="stk"/>
    <Constant dataType="string">stk1</Constant>
  </Apply>
  <Constant dataType="double">1</Constant>
  <Apply function="if">
    <Apply function="equal">
      <FieldRef field="stk"/>
```

```
      &lt;Constant dataType="string"&gt;stk2&lt;/Constant&gt;
    &lt;/Apply&gt;
    &lt;Constant dataType="double"&gt;0&lt;/Constant&gt;
    &lt;FieldRef field="NA"/&gt;
  &lt;/Apply&gt;
&lt;/Apply&gt;
 &lt;/DerivedField&gt;
 &lt;DerivedField name="Cancer_cat" dataType="double" optype="continuous"&gt;
&lt;Apply function="if"&gt;
  &lt;Apply function="equal"&gt;
    &lt;FieldRef field="can"/&gt;
    &lt;Constant dataType="string"&gt;can1&lt;/Constant&gt;
  &lt;/Apply&gt;
  &lt;Constant dataType="double"&gt;1&lt;/Constant&gt;
  &lt;Apply function="if"&gt;
    &lt;Apply function="equal"&gt;
      &lt;FieldRef field="can"/&gt;
      &lt;Constant dataType="string"&gt;can2&lt;/Constant&gt;
    &lt;/Apply&gt;
    &lt;Constant dataType="double"&gt;0&lt;/Constant&gt;
    &lt;FieldRef field="NA"/&gt;
  &lt;/Apply&gt;
&lt;/Apply&gt;
 &lt;/DerivedField&gt;
 <DerivedField name="DiabetesAge_int" dataType="double"
optype="continuous">
&lt;Apply function="*"&gt;
  &lt;FieldRef field="Diabetes_cat"/&gt;
  &lt;FieldRef field="Age_cont"/&gt;
&lt;/Apply&gt;
 &lt;/DerivedField&gt;
 <DerivedField name="CancerAge_int" dataType="double"
optype="continuous">
&lt;Apply function="*"&gt;
  &lt;FieldRef field="Cancer_cat"/&gt;
  &lt;FieldRef field="Age_cont"/&gt;
&lt;/Apply&gt;
 &lt;/DerivedField&gt;
 &lt;DerivedField name="weightkg" dataType="double" optype="continuous"&gt;
&lt;Apply function="/"&gt;
  &lt;FieldRef field="weightlb"/&gt;
  &lt;Constant dataType="double"&gt;2.2046226218&lt;/Constant&gt;
&lt;/Apply&gt;
 &lt;/DerivedField&gt;
 &lt;DerivedField name="heightm" dataType="double" optype="continuous"&gt;
&lt;Apply function="/"&gt;
  &lt;Apply function="+"&gt;
    &lt;Apply function="*"&gt;
      &lt;FieldRef field="heightin_hft"/&gt;
      &lt;Constant dataType="double"&gt;12&lt;/Constant&gt;
    &lt;/Apply&gt;
    &lt;FieldRef field="heightin_hin"/&gt;
  &lt;/Apply&gt;
  &lt;Constant dataType="double"&gt;39.3701&lt;/Constant&gt;
```

```
</Apply>
 </DerivedField>
 <DerivedField name="BMI_spline" dataType="double" optype="continuous">
<Apply function="if">
  <Apply function="lessOrEqual">
    <Apply function="-">
      <Apply function="/">
        <FieldRef field="weightkg"/>
        <Apply function="*">
          <FieldRef field="heightm"/>
          <FieldRef field="heightm"/>
        </Apply>
      </Apply>
      <Constant dataType="double">35</Constant>
    </Apply>
    <Constant dataType="double">0</Constant>
  </Apply>
  <Constant dataType="double">0</Constant>
  <Apply function="if">
    <Apply function="greaterThan">
      <Apply function="-">
        <Apply function="/">
          <FieldRef field="weightkg"/>
          <Apply function="*">
            <FieldRef field="heightm"/>
            <FieldRef field="heightm"/>
          </Apply>
        </Apply>
        <Constant dataType="double">35</Constant>
      </Apply>
      <Constant dataType="double">0</Constant>
    </Apply>
    <Apply function="-">
      <Apply function="/">
        <FieldRef field="weightkg"/>
        <Apply function="*">
          <FieldRef field="heightm"/>
          <FieldRef field="heightm"/>
        </Apply>
      </Apply>
      <Constant dataType="double">35</Constant>
    </Apply>
  </Apply>
</Apply>
 </DerivedField>
</LocalTransformations>
 <GeneralRegressionModel modelType="CoxRegression"
modelName="MPoRT_MaleModel" functionName="regression"
algorithmName="coxph" startTimeVariable="start" endTimeVariable="stop"
statusVariable="EventDeath">
  <MiningSchema>
   <MiningField name="survival" usageType="predicted"/>
   <MiningField name="Age_cont" usageType="active"/>
   <MiningField name="Age_spline" usageType="active"/>
```

```
    <MiningField name="QSLight_df" usageType="active"/>
    <MiningField name="QSHeavy_df" usageType="active"/>
    <MiningField name="PhysicalActivity_cont" usageType="active"/>
    <MiningField name="DietScore_cont" usageType="active"/>
    <MiningField name="AlcoholHeavy_cat" usageType="active"/>
    <MiningField name="AlcoholMod_cat" usageType="active"/>
    <MiningField name="DepIndMod_cat" usageType="active"/>
    <MiningField name="DepIndHigh_cat" usageType="active"/>
    <MiningField name="EduNoGrad_cat" usageType="active"/>
    <MiningField name="EduHSGrad_cat" usageType="active"/>
    <MiningField name="ImEth0To15_cat" usageType="active"/>
    <MiningField name="ImEth16To30_cat" usageType="active"/>
    <MiningField name="ImEth31To45_cat" usageType="active"/>
    <MiningField name="HeartDis_cat" usageType="active"/>
    <MiningField name="Stroke_cat" usageType="active"/>
    <MiningField name="Cancer_cat" usageType="active"/>
    <MiningField name="Diabetes_cat" usageType="active"/>
    <MiningField name="BMI_spline" usageType="active"/>
    <MiningField name="CancerAge_Int" usageType="active"/>
    <MiningField name="DiabetesAge_Int" usageType="active"/>
    <MiningField name="start" usageType="active"/>
    <MiningField name="stop" usageType="active"/>
    <MiningField name="EventDeath" usageType="active"/>
   </MiningSchema>
   <Output>
    <OutputField name="Predicted_survival" feature="predictedValue"/>
    <OutputField name="cumulativeHazard" feature="transformedValue">
     <Apply function="*">
      <Constant>-1.0</Constant>
      <Apply function="ln">
       <FieldRef field="Predicted_survival"/>
      </Apply>
     </Apply>
    </OutputField>
   </Output>
   <ParameterList>
    <Parameter name="p0" label="Age_cont"
referencePoint="51.9855867845952"/>
    <Parameter name="p1" label="Age_spline"
referencePoint="2.23085276362985"/>
    <Parameter name="p2" label="QSLight_df"
referencePoint="0.215339125424739"/>
    <Parameter name="p3" label="QSHeavy_df"
referencePoint="0.169791058200741"/>
    <Parameter name="p4" label="PhysicalActivity_cont"
referencePoint="0.423291673033803"/>
    <Parameter name="p5" label="DietScore_cont"
referencePoint="4.45588356813016"/>
    <Parameter name="p6" label="AlcoholHeavy_cat1"
referencePoint="0.214680858065295"/>
    <Parameter name="p7" label="AlcoholMod_cat1"
referencePoint="0.237017319990201"/>
    <Parameter name="p8" label="DepIndMod_cat1"
referencePoint="0.635300311884138"/>
```

```
   <Parameter name="p9" label="DepIndHigh_cat1"
referencePoint="0.157419222739388"/>
   <Parameter name="p10" label="EduNoGrad_cat1"
referencePoint="0.171463149773857"/>
   <Parameter name="p11" label="EduHSGrad_cat1"
referencePoint="0.249920475029159"/>
   <Parameter name="p12" label="ImEth0To15_cat1"
referencePoint="0.0394443855370586"/>
   <Parameter name="p13" label="ImEth16To30_cat1"
referencePoint="0.0481756788873085"/>
   <Parameter name="p14" label="ImEth31To45_cat1"
referencePoint="0.0583804693984256"/>
   <Parameter name="p15" label="HeartDis_cat1"
referencePoint="0.0782160081024062"/>
   <Parameter name="p16" label="Stroke_cat1"
referencePoint="0.0133748203832555"/>
   <Parameter name="p17" label="Cancer_cat1"
referencePoint="0.0234370143949338"/>
   <Parameter name="p18" label="Diabetes_cat1"
referencePoint="0.0720953275880351"/>
   <Parameter name="p19" label="BMI_spline"
referencePoint="0.185952928529901"/>
   <Parameter name="p20" label="CancerAge_Int"
referencePoint="1.62081762638986"/>
   <Parameter name="p21" label="DiabetesAge_Int"
referencePoint="4.6754430546364"/>
  </ParameterList>
  <FactorList>
   <Predictor name="AlcoholHeavy_cat"/>
   <Predictor name="AlcoholMod_cat"/>
   <Predictor name="DepIndMod_cat"/>
   <Predictor name="DepIndHigh_cat"/>
   <Predictor name="EduNoGrad_cat"/>
   <Predictor name="EduHSGrad_cat"/>
   <Predictor name="ImEth0To15_cat"/>
   <Predictor name="ImEth16To30_cat"/>
   <Predictor name="ImEth31To45_cat"/>
   <Predictor name="HeartDis_cat"/>
   <Predictor name="Stroke_cat"/>
   <Predictor name="Cancer_cat"/>
   <Predictor name="Diabetes_cat"/>
  </FactorList>
  <CovariateList>
   <Predictor name="Age_cont"/>
   <Predictor name="Age_spline"/>
   <Predictor name="QSLight_df"/>
   <Predictor name="QSHeavy_df"/>
   <Predictor name="PhysicalActivity_cont"/>
   <Predictor name="DietScore_cont"/>
   <Predictor name="BMI_spline"/>
   <Predictor name="CancerAge_Int"/>
   <Predictor name="DiabetesAge_Int"/>
  </CovariateList>
  <PPMatrix>
```

<PPCell value="1" predictorName="Age_cont" parameterName="p0"/>
<PPCell value="1" predictorName="Age_spline" parameterName="p1"/>
<PPCell value="1" predictorName="QSLight_df" parameterName="p2"/>
<PPCell value="1" predictorName="QSHeavy_df" parameterName="p3"/>
<PPCell value="1" predictorName="PhysicalActivity_cont"
parameterName="p4"/>
<PPCell value="1" predictorName="DietScore_cont" parameterName="p5"/>
<PPCell value="1" predictorName="AlcoholHeavy_cat"
parameterName="p6"/>
<PPCell value="1" predictorName="AlcoholMod_cat" parameterName="p7"/>
<PPCell value="1" predictorName="DepIndMod_cat" parameterName="p8"/>
<PPCell value="1" predictorName="DepIndHigh_cat" parameterName="p9"/>
<PPCell value="1" predictorName="EduNoGrad_cat" parameterName="p10"/>
<PPCell value="1" predictorName="EduHSGrad_cat" parameterName="p11"/>
<PPCell value="1" predictorName="ImEth0To15_cat" parameterName="p12"/>
<PPCell value="1" predictorName="ImEth16To30_cat"
parameterName="p13"/>
<PPCell value="1" predictorName="ImEth31To45_cat"
parameterName="p14"/>
<PPCell value="1" predictorName="HeartDis_cat" parameterName="p15"/>
<PPCell value="1" predictorName="Stroke_cat" parameterName="p16"/>
<PPCell value="1" predictorName="Cancer_cat" parameterName="p17"/>
<PPCell value="1" predictorName="Diabetes_cat" parameterName="p18"/>
<PPCell value="1" predictorName="BMI_spline" parameterName="p19"/>
<PPCell value="1" predictorName="CancerAge_Int" parameterName="p20"/>
<PPCell value="1" predictorName="DiabetesAge_Int"
parameterName="p21"/>
</PPMatrix>
<ParamMatrix>
<PCell parameterName="p0" df="1" beta="0.0831368085304069"/>
<PCell parameterName="p1" df="1" beta="0.032863950554692"/>
<PCell parameterName="p2" df="1" beta="0.900307809717107"/>
<PCell parameterName="p3" df="1" beta="1.03948313110627"/>
<PCell parameterName="p4" df="1" beta="-0.703145562727891"/>
<PCell parameterName="p5" df="1" beta="-0.0344106524801484"/>
<PCell parameterName="p6" df="1" beta="0.0508703699519003"/>
<PCell parameterName="p7" df="1" beta="-0.192706462213306"/>
<PCell parameterName="p8" df="1" beta="0.0690614098959036"/>
<PCell parameterName="p9" df="1" beta="0.220101731941223"/>
<PCell parameterName="p10" df="1" beta="0.185703949355632"/>
<PCell parameterName="p11" df="1" beta="0.0863226867368134"/>
<PCell parameterName="p12" df="1" beta="-0.982657657310987"/>
<PCell parameterName="p13" df="1" beta="-0.404318147113174"/>
<PCell parameterName="p14" df="1" beta="-0.117087334405182"/>
<PCell parameterName="p15" df="1" beta="0.379459368136119"/>
<PCell parameterName="p16" df="1" beta="0.224159490570495"/>
<PCell parameterName="p17" df="1" beta="4.40896143527765"/>
<PCell parameterName="p18" df="1" beta="1.98499540516865"/>
<PCell parameterName="p19" df="1" beta="0.0303768206375058"/>
<PCell parameterName="p20" df="1" beta="-0.0497850790110705"/>
<PCell parameterName="p21" df="1" beta="-0.0209732533287309"/>
</ParamMatrix>
<BaseCumHazardTables maxTime="11">
<BaselineCell time="1" cumHazard="0.00270416361034808"/>

```
    <BaselineCell time="2" cumHazard="0.00564932797323397"/>
    <BaselineCell time="3" cumHazard="0.00909212820474581"/>
    <BaselineCell time="4" cumHazard="0.0123586445984695"/>
    <BaselineCell time="5" cumHazard="0.0155935081953894"/>
    <BaselineCell time="6" cumHazard="0.0184560410825005"/>
    <BaselineCell time="7" cumHazard="0.0213841200205223"/>
    <BaselineCell time="8" cumHazard="0.0240273358307661"/>
    <BaselineCell time="9" cumHazard="0.0264290994375949"/>
    <BaselineCell time="10" cumHazard="0.0284100984083658"/>
    <BaselineCell time="11" cumHazard="0.0285749622160038"/>
   </BaseCumHazardTables>
  </GeneralRegressionModel>
</PMML>
```


**MPoRT female model**


```
<PMML version="1.0" xmlns="http://www.dmg.org/PMML-4_2"
xmlns:xsi="http://www.w3.org/2001/XMLSchema-instance"
xsi:schemaLocation="http://www.dmg.org/PMML-4_2 http://www.dmg.org/v4-
2/pmml-4-2.xsd">
<Header copyright="Copyright (c) 2016 The Ottawa Hospital"
description="MPoRT Female V1.0.0">
<Annotation>Mortality (all-cause) Population Risk Tool (MPoRT)
  <Extension name="author">Doug Manuel</Extension>
</Annotation>
<Extension name="user" value="theottawahospital" extender="COXPH"/>
  <Application name="COXPH" version="1.4"/>
  <Timestamp>2016-05-26 17:01:13</Timestamp>
 </Header>
 <DataDictionary numberOfFields="26">
  <DataField name="survival" optype="continuous" dataType="double"/>
  <DataField name="Age_cont" optype="continuous" dataType="double"/>
  <DataField name="Age_spline" optype="continuous" dataType="double"/>
  <DataField name="QSLight_df" optype="continuous" dataType="double"/>
  <DataField name="QSHeavy_df" optype="continuous" dataType="double"/>
  <DataField name="PhysicalActivity_cont" optype="continuous"
dataType="double"/>
  <DataField name="DietScore_cont" optype="continuous"
dataType="double"/>
  <DataField name="AlcoholHeavy_cat" optype="categorical"
dataType="string"/>
  <DataField name="AlcoholMod_cat" optype="categorical"
dataType="string"/>
  <DataField name="DepIndMod_cat" optype="categorical"
dataType="string"/>
  <DataField name="DepIndHigh_cat" optype="categorical"
dataType="string"/>
  <DataField name="EduNoGrad_cat" optype="categorical"
dataType="string"/>
  <DataField name="EduHSGrad_cat" optype="categorical"
dataType="string"/>
  <DataField name="ImEth0To15_cat" optype="categorical"
dataType="string"/>
```

```
  <DataField name="ImEth16To30_cat" optype="categorical"
dataType="string"/>
  <DataField name="ImEth31To45_cat" optype="categorical"
dataType="string"/>
  <DataField name="HeartDis_cat" optype="categorical" dataType="string"/>
  <DataField name="Stroke_cat" optype="categorical" dataType="string"/>
  <DataField name="Cancer_cat" optype="categorical" dataType="string"/>
  <DataField name="Diabetes_cat" optype="categorical" dataType="string"/>
  <DataField name="BMI_spline" optype="continuous" dataType="double"/>
  <DataField name="CancerAge_Int" optype="continuous" dataType="double"/>
  <DataField name="DiabetesAge_Int" optype="continuous"
dataType="double"/>
  <DataField name="start" optype="continuous" dataType="double"/>
  <DataField name="stop" optype="continuous" dataType="double"/>
  <DataField name="EventDeath" optype="continuous" dataType="double"/>
 </DataDictionary>
<LocalTransformations>
 <DerivedField name="Sex" dataType="double" optype="continuous">
<Apply function="if">
  <Apply function="equal">
    <FieldRef field="sex"/>
    <Constant dataType="string">fem</Constant>
  </Apply>
  <Constant dataType="double">1</Constant>
  <Apply function="if">
    <Apply function="equal">
      <FieldRef field="sex"/>
      <Constant dataType="string">male</Constant>
    </Apply>
    <Constant dataType="double">0</Constant>
  </Apply>
</Apply>
 </DerivedField>
 <DerivedField name="Age_spline" dataType="double" optype="continuous">
<Apply function="if">
  <Apply function="and">
    <Apply function="equal">
      <FieldRef field="Sex"/>
      <Constant dataType="string">fem</Constant>
    </Apply>
    <Apply function="greaterThan">
      <FieldRef field="Age"/>
      <Constant dataType="double">80</Constant>
    </Apply>
  </Apply>
  <Apply function="-">
    <FieldRef field="Age"/>
    <Constant dataType="double">80</Constant>
  </Apply>
  <Apply function="if">
    <Apply function="equal">
      <FieldRef field="Sex"/>
      <Constant dataType="string">fem</Constant>
    </Apply>
```

```
    <Constant dataType="double">0</Constant>
    <Apply function="if">
      <Apply function="and">
        <Apply function="equal">
          <FieldRef field="Sex"/>
          <Constant dataType="string">male</Constant>
        </Apply>
        <Apply function="greaterThan">
          <FieldRef field="Age"/>
          <Constant dataType="double">65</Constant>
        </Apply>
      </Apply>
      <Apply function="-">
        <FieldRef field="Age"/>
        <Constant dataType="double">65</Constant>
      </Apply>
      <Constant dataType="double">0</Constant>
    </Apply>
  </Apply>
</Apply>
 </DerivedField>
 <DerivedField name="EduNoGrad_cat" dataType="double"
optype="continuous">
<Apply function="if">
  <Apply function="and">
    <Apply function="equal">
      <FieldRef field="hs"/>
      <Constant dataType="string">hs2</Constant>
    </Apply>
    <Apply function="equal">
      <FieldRef field="ed"/>
      <Constant dataType="string">ed2</Constant>
    </Apply>
  </Apply>
  <Constant dataType="double">1</Constant>
  <Constant dataType="double">0</Constant>
</Apply>
 </DerivedField>
 <DerivedField name="EduHSGrad_cat" dataType="double"
optype="continuous">
<Apply function="if">
  <Apply function="or">
    <Apply function="and">
      <Apply function="equal">
        <FieldRef field="hs"/>
        <Constant dataType="string">hs1</Constant>
      </Apply>
      <Apply function="equal">
        <FieldRef field="ed"/>
        <Constant dataType="string">ed2</Constant>
      </Apply>
    </Apply>
    <Apply function="and">
      <Apply function="and">
```

```
        <Apply function="equal">
          <FieldRef field="hs"/>
          <Constant dataType="string">hs1</Constant>
        </Apply>
        <Apply function="equal">
          <FieldRef field="ed"/>
          <Constant dataType="string">ed1</Constant>
        </Apply>
      </Apply>
      <Apply function="equal">
        <FieldRef field="hdg"/>
        <Constant dataType="string">hdg1</Constant>
      </Apply>
    </Apply>
  </Apply>
  <Constant dataType="double">1</Constant>
  <Constant dataType="double">0</Constant>
</Apply>
 </DerivedField>
 <DerivedField name="DepIndHigh_cat" dataType="double"
optype="continuous">
<Apply function="if">
  <Apply function="equal">
    <FieldRef field="dep"/>
    <Constant dataType="string">dep1</Constant>
  </Apply>
  <Constant dataType="double">1</Constant>
  <Constant dataType="double">0</Constant>
</Apply>
 </DerivedField>
 <DerivedField name="DepIndMod_cat" dataType="double"
optype="continuous">
<Apply function="if">
  <Apply function="equal">
    <FieldRef field="dep"/>
    <Constant dataType="string">dep2</Constant>
  </Apply>
  <Constant dataType="double">1</Constant>
  <Constant dataType="double">0</Constant>
</Apply>
 </DerivedField>
 <DerivedField name="ImEth0To15_cat" dataType="double"
optype="continuous">
<Apply function="if">
  <Apply function="and">
    <Apply function="and">
      <Apply function="equal">
        <FieldRef field="imm"/>
        <Constant dataType="string">imm2</Constant>
      </Apply>
      <Apply function="greaterOrEqual">
        <FieldRef field="imyr"/>
        <Constant dataType="double">1</Constant>
      </Apply>
```

```
    </Apply>
    <Apply function="lessOrEqual">
      <FieldRef field="imyr"/>
      <Constant dataType="double">15</Constant>
    </Apply>
  </Apply>
  <Constant dataType="double">1</Constant>
  <Apply function="if">
    <Apply function="or">
      <Apply function="equal">
        <FieldRef field="imm"/>
        <Constant dataType="string">imm1</Constant>
      </Apply>
      <Apply function="greaterThan">
        <FieldRef field="imyr"/>
        <Constant dataType="double">15</Constant>
      </Apply>
    </Apply>
    <Constant dataType="double">0</Constant>
    <Constant dataType="double">0</Constant>
  </Apply>
</Apply>
 </DerivedField>
 <DerivedField name="ImEth16To30_cat" dataType="double"
optype="continuous">
<Apply function="if">
  <Apply function="and">
    <Apply function="and">
      <Apply function="equal">
        <FieldRef field="imm"/>
        <Constant dataType="string">imm2</Constant>
      </Apply>
      <Apply function="greaterThan">
        <FieldRef field="imyr"/>
        <Constant dataType="double">15</Constant>
      </Apply>
    </Apply>
    <Apply function="lessOrEqual">
      <FieldRef field="imyr"/>
      <Constant dataType="double">30</Constant>
    </Apply>
  </Apply>
  <Constant dataType="double">1</Constant>
  <Apply function="if">
    <Apply function="or">
      <Apply function="or">
        <Apply function="equal">
          <FieldRef field="imm"/>
          <Constant dataType="string">imm1</Constant>
        </Apply>
        <Apply function="lessOrEqual">
          <FieldRef field="imyr"/>
          <Constant dataType="double">15</Constant>
        </Apply>
```

```
      </Apply>
      <Apply function="greaterThan">
        <FieldRef field="imyr"/>
        <Constant dataType="double">30</Constant>
      </Apply>
    </Apply>
    <Constant dataType="double">0</Constant>
    <Constant dataType="double">0</Constant>
  </Apply>
</Apply>
 </DerivedField>
 <DerivedField name="ImEth31To45_cat" dataType="double"
optype="continuous">
<Apply function="if">
  <Apply function="and">
    <Apply function="and">
      <Apply function="equal">
        <FieldRef field="imm"/>
        <Constant dataType="string">imm2</Constant>
      </Apply>
      <Apply function="greaterThan">
        <FieldRef field="imyr"/>
        <Constant dataType="double">30</Constant>
      </Apply>
    </Apply>
    <Apply function="lessOrEqual">
      <FieldRef field="imyr"/>
      <Constant dataType="double">45</Constant>
    </Apply>
  </Apply>
  <Constant dataType="double">1</Constant>
  <Apply function="if">
    <Apply function="or">
      <Apply function="or">
        <Apply function="equal">
          <FieldRef field="imm"/>
          <Constant dataType="string">imm1</Constant>
        </Apply>
        <Apply function="lessOrEqual">
          <FieldRef field="imyr"/>
          <Constant dataType="double">30</Constant>
        </Apply>
      </Apply>
      <Apply function="greaterThan">
        <FieldRef field="imyr"/>
        <Constant dataType="double">45</Constant>
      </Apply>
    </Apply>
    <Constant dataType="double">0</Constant>
    <Constant dataType="double">0</Constant>
  </Apply>
</Apply>
 </DerivedField>
```

```
 <DerivedField name="formerlightflag" dataType="double"
optype="continuous">
<Apply function="if">
  <Apply function="and">
    <Apply function="and">
      <Apply function="equal">
        <FieldRef field="smk"/>
        <Constant dataType="string">smk3</Constant>
      </Apply>
      <Apply function="equal">
        <FieldRef field="evdn"/>
        <Constant dataType="string">evdn1</Constant>
      </Apply>
    </Apply>
    <Apply function="lessThan">
      <FieldRef field="cigdayf"/>
      <Constant dataType="double">20</Constant>
    </Apply>
  </Apply>
  <Constant dataType="double">1</Constant>
  <Apply function="if">
    <Apply function="and">
      <Apply function="and">
        <Apply function="equal">
          <FieldRef field="smk"/>
          <Constant dataType="string">smk3</Constant>
        </Apply>
        <Apply function="equal">
          <FieldRef field="evdn"/>
          <Constant dataType="string">evdn2</Constant>
        </Apply>
      </Apply>
      <Apply function="equal">
        <FieldRef field="s100"/>
        <Constant dataType="string">s1001</Constant>
      </Apply>
    </Apply>
    <Constant dataType="double">1</Constant>
    <Constant dataType="double">0</Constant>
  </Apply>
</Apply>
 </DerivedField>
 <DerivedField name="formerheavyflag" dataType="double"
optype="continuous">
<Apply function="if">
  <Apply function="and">
    <Apply function="and">
      <Apply function="equal">
        <FieldRef field="smk"/>
        <Constant dataType="string">smk3</Constant>
      </Apply>
      <Apply function="equal">
        <FieldRef field="evdn"/>
        <Constant dataType="string">evdn1</Constant>
```

```
        </Apply>
      </Apply>
      <Apply function="greaterOrEqual">
        <FieldRef field="cigdayf"/>
        <Constant dataType="double">20</Constant>
      </Apply>
    </Apply>
    <Constant dataType="double">1</Constant>
    <Constant dataType="double">0</Constant>
  </Apply>
   </DerivedField>
   <DerivedField name="quittime" dataType="double" optype="continuous">
  <Apply function="if">
    <Apply function="or">
      <Apply function="equal">
        <FieldRef field="formerlightflag"/>
        <Constant dataType="double">0</Constant>
      </Apply>
      <Apply function="equal">
        <FieldRef field="formerheavyflag"/>
        <Constant dataType="double">0</Constant>
      </Apply>
    </Apply>
    <Constant dataType="double">0</Constant>
    <Apply function="if">
      <Apply function="or">
        <Apply function="equal">
          <FieldRef field="smk"/>
          <Constant dataType="string">smk1</Constant>
        </Apply>
        <Apply function="=">
          <Apply function="and">
            <Apply function="and">
              <Apply function="equal">
                <FieldRef field="smk"/>
                <Constant dataType="string">smk3</Constant>
              </Apply>
              <Apply function="equal">
                <FieldRef field="evdn"/>
                <Constant dataType="string">evdn2</Constant>
              </Apply>
            </Apply>
            <FieldRef field="s100"/>
          </Apply>
          <Constant dataType="string">s1002</Constant>
        </Apply>
      </Apply>
      <Constant dataType="double">0</Constant>
      <Apply function="if">
        <Apply function="equal">
          <FieldRef field="stpn"/>
          <Constant dataType="string">stpn1</Constant>
        </Apply>
        <Constant dataType="double">0</Constant>
```

```
      <Apply function="if">
        <Apply function="equal">
          <FieldRef field="stpn"/>
          <Constant dataType="string">stpn2</Constant>
        </Apply>
        <Constant dataType="double">1</Constant>
        <Apply function="if">
          <Apply function="equal">
            <FieldRef field="stpn"/>
            <Constant dataType="string">stpn3</Constant>
          </Apply>
          <Constant dataType="double">2</Constant>
          <Apply function="if">
            <Apply function="equal">
              <FieldRef field="stpn"/>
              <Constant dataType="string">stpn4</Constant>
            </Apply>
            <FieldRef field="stpny"/>
            <FieldRef field="NA"/>
          </Apply>
        </Apply>
      </Apply>
    </Apply>
  </Apply>
</Apply>
 </DerivedField>
 <DerivedField name="smk_lightraw" dataType="double" optype="continuous">
<Apply function="if">
  <Apply function="and">
    <Apply function="equal">
      <FieldRef field="smk"/>
      <Constant dataType="string">smk1</Constant>
    </Apply>
    <Apply function="lessThan">
      <FieldRef field="cigdayd"/>
      <Constant dataType="double">20</Constant>
    </Apply>
  </Apply>
  <Constant dataType="double">1</Constant>
  <Apply function="if">
    <Apply function="equal">
      <FieldRef field="smk"/>
      <Constant dataType="string">smk2</Constant>
    </Apply>
    <Constant dataType="double">1</Constant>
    <Apply function="if">
      <Apply function="and">
        <Apply function="and">
          <Apply function="equal">
            <FieldRef field="smk"/>
            <Constant dataType="string">smk3</Constant>
          </Apply>
          <Apply function="equal">
            <FieldRef field="evdn"/>
```

```
              <Constant dataType="string">evdn1</Constant>
            </Apply>
          </Apply>
          <Apply function="lessThan">
            <FieldRef field="cigdayf"/>
            <Constant dataType="double">20</Constant>
          </Apply>
        </Apply>
        <Constant dataType="double">1</Constant>
        <Apply function="if">
          <Apply function="and">
            <Apply function="and">
              <Apply function="equal">
                <FieldRef field="smk"/>
                <Constant dataType="string">smk3</Constant>
              </Apply>
              <Apply function="equal">
                <FieldRef field="evdn"/>
                <Constant dataType="string">evdn2</Constant>
              </Apply>
            </Apply>
            <Apply function="equal">
              <FieldRef field="s100"/>
              <Constant dataType="string">s1001</Constant>
            </Apply>
          </Apply>
          <Constant dataType="double">1</Constant>
          <Constant dataType="double">0</Constant>
        </Apply>
      </Apply>
    </Apply>
  </Apply>
 </DerivedField>
 <DerivedField name="smk_heavyraw" dataType="double" optype="continuous">
<Apply function="if">
  <Apply function="and">
    <Apply function="equal">
      <FieldRef field="smk"/>
      <Constant dataType="string">smk1</Constant>
    </Apply>
    <Apply function="greaterOrEqual">
      <FieldRef field="cigdayd"/>
      <Constant dataType="double">20</Constant>
    </Apply>
  </Apply>
  <Constant dataType="double">1</Constant>
  <Apply function="if">
    <Apply function="and">
      <Apply function="and">
        <Apply function="equal">
          <FieldRef field="smk"/>
          <Constant dataType="string">smk3</Constant>
        </Apply>
        <Apply function="equal">
```

```
          <FieldRef field="evdn"/>
          <Constant dataType="string">evdn1</Constant>
        </Apply>
      </Apply>
      <Apply function="greaterOrEqual">
        <FieldRef field="cigdayf"/>
        <Constant dataType="double">20</Constant>
      </Apply>
    </Apply>
    <Constant dataType="double">1</Constant>
    <Constant dataType="double">0</Constant>
  </Apply>
</Apply>
 </DerivedField>
 <DerivedField name="QSLight_df" dataType="double" optype="continuous">
<Apply function="if">
  <Apply function="and">
    <Apply function="equal">
      <FieldRef field="formerlightflag"/>
      <Constant dataType="double">1</Constant>
    </Apply>
    <Apply function="equal">
      <FieldRef field="sex"/>
      <Constant dataType="string">fem</Constant>
    </Apply>
  </Apply>
  <Apply function="exp">
    <Apply function="/">
      <FieldRef field="quittime"/>
      <Constant dataType="double">26</Constant>
    </Apply>
  </Apply>
  <Apply function="if">
    <Apply function="and">
      <Apply function="equal">
        <FieldRef field="formerlightflag"/>
        <Constant dataType="double">1</Constant>
      </Apply>
      <Apply function="equal">
        <FieldRef field="sex"/>
        <Constant dataType="string">male</Constant>
      </Apply>
    </Apply>
    <Apply function="exp">
      <Apply function="/">
        <FieldRef field="quittime"/>
        <Constant dataType="double">15</Constant>
      </Apply>
    </Apply>
    <FieldRef field="smk_lightraw"/>
  </Apply>
</Apply>
 </DerivedField>
 <DerivedField name="QSHeavy_df" dataType="double" optype="continuous">
```

```
&lt;Apply function="if"&gt;
  &lt;Apply function="and"&gt;
    &lt;Apply function="equal"&gt;
      &lt;FieldRef field="formerheavyflag"/&gt;
      &lt;Constant dataType="double"&gt;1&lt;/Constant&gt;
    &lt;/Apply&gt;
    &lt;Apply function="equal"&gt;
      &lt;FieldRef field="sex"/&gt;
      &lt;Constant dataType="string"&gt;fem&lt;/Constant&gt;
    &lt;/Apply&gt;
  &lt;/Apply&gt;
  &lt;Apply function="exp"&gt;
    &lt;Apply function="/"&gt;
      &lt;FieldRef field="quittime"/&gt;
      &lt;Constant dataType="double"&gt;26&lt;/Constant&gt;
    &lt;/Apply&gt;
  &lt;/Apply&gt;
  &lt;Apply function="if"&gt;
    &lt;Apply function="and"&gt;
      &lt;Apply function="equal"&gt;
        &lt;FieldRef field="formerheavyflag"/&gt;
        &lt;Constant dataType="double"&gt;1&lt;/Constant&gt;
      &lt;/Apply&gt;
      &lt;Apply function="equal"&gt;
        &lt;FieldRef field="sex"/&gt;
        &lt;Constant dataType="string"&gt;male&lt;/Constant&gt;
      &lt;/Apply&gt;
    &lt;/Apply&gt;
    &lt;Apply function="exp"&gt;
      &lt;Apply function="/"&gt;
        &lt;FieldRef field="quittime"/&gt;
        &lt;Constant dataType="double"&gt;15&lt;/Constant&gt;
      &lt;/Apply&gt;
    &lt;/Apply&gt;
    &lt;FieldRef field="smk_heavyraw"/&gt;
  &lt;/Apply&gt;
&lt;/Apply&gt;
 &lt;/DerivedField&gt;
 &lt;DerivedField name="walking" dataType="double" optype="continuous"&gt;
&lt;Apply function="if"&gt;
  &lt;Apply function="equal"&gt;
    &lt;FieldRef field="lpa_lpa1"/&gt;
    &lt;Constant dataType="string"&gt;Yes&lt;/Constant&gt;
  &lt;/Apply&gt;
  &lt;Constant dataType="double"&gt;1&lt;/Constant&gt;
  &lt;Constant dataType="double"&gt;0&lt;/Constant&gt;
&lt;/Apply&gt;
 &lt;/DerivedField&gt;
 &lt;DerivedField name="walking_t" dataType="double" optype="continuous"&gt;
&lt;Apply function="if"&gt;
  &lt;Apply function="is.na"&gt;
    &lt;FieldRef field="lpat_lpa1"/&gt;
  &lt;/Apply&gt;
  &lt;Constant dataType="double"&gt;0&lt;/Constant&gt;
```

```
  <FieldRef field="lpat_lpa1"/>
</Apply>
 </DerivedField>
 <DerivedField name="walking_h" dataType="double" optype="continuous">
<Apply function="if">
  <Apply function="is.na">
    <FieldRef field="lpam_lpa1"/>
  </Apply>
  <Constant dataType="double">0</Constant>
  <Apply function="if">
    <Apply function="equal">
      <FieldRef field="lpam_lpa1"/>
      <Constant dataType="string">lpa1</Constant>
    </Apply>
    <Constant dataType="double">0.2167</Constant>
    <Apply function="if">
      <Apply function="equal">
        <FieldRef field="lpam_lpa1"/>
        <Constant dataType="string">lpa2</Constant>
      </Apply>
      <Constant dataType="double">0.3833</Constant>
      <Apply function="if">
        <Apply function="equal">
          <FieldRef field="lpam_lpa1"/>
          <Constant dataType="string">lpa3</Constant>
        </Apply>
        <Constant dataType="double">0.75</Constant>
        <Apply function="if">
          <Apply function="equal">
            <FieldRef field="lpam_lpa1"/>
            <Constant dataType="string">lpa4</Constant>
          </Apply>
          <Constant dataType="double">1</Constant>
          <Constant dataType="double">0</Constant>
        </Apply>
      </Apply>
    </Apply>
  </Apply>
</Apply>
 </DerivedField>
 <DerivedField name="garden" dataType="double" optype="continuous">
<Apply function="if">
  <Apply function="equal">
    <FieldRef field="lpa_lpa2"/>
    <Constant dataType="string">Yes</Constant>
  </Apply>
  <Constant dataType="double">1</Constant>
  <Constant dataType="double">0</Constant>
</Apply>
 </DerivedField>
 <DerivedField name="garden_t" dataType="double" optype="continuous">
<Apply function="if">
  <Apply function="is.na">
    <FieldRef field="lpat_lpa2"/>
```

```
    </Apply>
    <Constant dataType="double">0</Constant>
    <FieldRef field="lpat_lpa2"/>
  </Apply>
   </DerivedField>
   <DerivedField name="garden_h" dataType="double" optype="continuous">
  <Apply function="if">
    <Apply function="is.na">
      <FieldRef field="lpam_lpa2"/>
    </Apply>
    <Constant dataType="double">0</Constant>
    <Apply function="if">
      <Apply function="equal">
        <FieldRef field="lpam_lpa2"/>
        <Constant dataType="string">lpa15</Constant>
      </Apply>
      <Constant dataType="double">0.2167</Constant>
      <Apply function="if">
        <Apply function="equal">
          <FieldRef field="lpam_lpa2"/>
          <Constant dataType="string">lpa30</Constant>
        </Apply>
        <Constant dataType="double">0.3833</Constant>
        <Apply function="if">
          <Apply function="equal">
            <FieldRef field="lpam_lpa2"/>
            <Constant dataType="string">lpa60</Constant>
          </Apply>
          <Constant dataType="double">0.75</Constant>
          <Apply function="if">
            <Apply function="equal">
              <FieldRef field="lpam_lpa2"/>
              <Constant dataType="string">lpa61</Constant>
            </Apply>
            <Constant dataType="double">1</Constant>
            <Constant dataType="double">0</Constant>
          </Apply>
        </Apply>
      </Apply>
    </Apply>
  </Apply>
   </DerivedField>
   <DerivedField name="swim" dataType="double" optype="continuous">
  <Apply function="if">
    <Apply function="equal">
      <FieldRef field="lpa_lpa3"/>
      <Constant dataType="string">Yes</Constant>
    </Apply>
    <Constant dataType="double">1</Constant>
    <Constant dataType="double">0</Constant>
  </Apply>
   </DerivedField>
   <DerivedField name="swim_t" dataType="double" optype="continuous">
  <Apply function="if">
```

```
  &lt;Apply function="is.na"&gt;
    &lt;FieldRef field="lpat_lpa3"/&gt;
  &lt;/Apply&gt;
  &lt;Constant dataType="double"&gt;0&lt;/Constant&gt;
  &lt;FieldRef field="lpat_lpa3"/&gt;
&lt;/Apply&gt;
 &lt;/DerivedField&gt;
 &lt;DerivedField name="swim_h" dataType="double" optype="continuous"&gt;
&lt;Apply function="if"&gt;
  &lt;Apply function="is.na"&gt;
    &lt;FieldRef field="lpam_lpa3"/&gt;
  &lt;/Apply&gt;
  &lt;Constant dataType="double"&gt;0&lt;/Constant&gt;
  &lt;Apply function="if"&gt;
    &lt;Apply function="equal"&gt;
      &lt;FieldRef field="lpam_lpa3"/&gt;
      &lt;Constant dataType="string"&gt;lpa15&lt;/Constant&gt;
    &lt;/Apply&gt;
    &lt;Constant dataType="double"&gt;0.2167&lt;/Constant&gt;
    &lt;Apply function="if"&gt;
      &lt;Apply function="equal"&gt;
        &lt;FieldRef field="lpam_lpa3"/&gt;
        &lt;Constant dataType="string"&gt;lpa30&lt;/Constant&gt;
      &lt;/Apply&gt;
      &lt;Constant dataType="double"&gt;0.3833&lt;/Constant&gt;
      &lt;Apply function="if"&gt;
        &lt;Apply function="equal"&gt;
          &lt;FieldRef field="lpam_lpa3"/&gt;
          &lt;Constant dataType="string"&gt;lpa60&lt;/Constant&gt;
        &lt;/Apply&gt;
        &lt;Constant dataType="double"&gt;0.75&lt;/Constant&gt;
        &lt;Apply function="if"&gt;
          &lt;Apply function="equal"&gt;
            &lt;FieldRef field="lpam_lpa3"/&gt;
            &lt;Constant dataType="string"&gt;lpa61&lt;/Constant&gt;
          &lt;/Apply&gt;
          &lt;Constant dataType="double"&gt;1&lt;/Constant&gt;
          &lt;Constant dataType="double"&gt;0&lt;/Constant&gt;
        &lt;/Apply&gt;
      &lt;/Apply&gt;
    &lt;/Apply&gt;
  &lt;/Apply&gt;
&lt;/Apply&gt;
 &lt;/DerivedField&gt;
 &lt;DerivedField name="bike" dataType="double" optype="continuous"&gt;
&lt;Apply function="if"&gt;
  &lt;Apply function="equal"&gt;
    &lt;FieldRef field="lpa_lpa4"/&gt;
    &lt;Constant dataType="string"&gt;Yes&lt;/Constant&gt;
  &lt;/Apply&gt;
  &lt;Constant dataType="double"&gt;1&lt;/Constant&gt;
  &lt;Constant dataType="double"&gt;0&lt;/Constant&gt;
&lt;/Apply&gt;
 &lt;/DerivedField&gt;
```

```xml
 <DerivedField name="bike_t" dataType="double" optype="continuous">
<Apply function="if">
  <Apply function="is.na">
    <FieldRef field="lpat_lpa4"/>
  </Apply>
  <Constant dataType="double">0</Constant>
  <FieldRef field="lpat_lpa4"/>
</Apply>
 </DerivedField>
 <DerivedField name="bike_h" dataType="double" optype="continuous">
<Apply function="if">
  <Apply function="is.na">
    <FieldRef field="lpam_lpa4"/>
  </Apply>
  <Constant dataType="double">0</Constant>
  <Apply function="if">
    <Apply function="equal">
      <FieldRef field="lpam_lpa4"/>
      <Constant dataType="string">lpa15</Constant>
    </Apply>
    <Constant dataType="double">0.2167</Constant>
    <Apply function="if">
      <Apply function="equal">
        <FieldRef field="lpam_lpa4"/>
        <Constant dataType="string">lpa30</Constant>
      </Apply>
      <Constant dataType="double">0.3833</Constant>
      <Apply function="if">
        <Apply function="equal">
          <FieldRef field="lpam_lpa4"/>
          <Constant dataType="string">lpa60</Constant>
        </Apply>
        <Constant dataType="double">0.75</Constant>
        <Apply function="if">
          <Apply function="equal">
            <FieldRef field="lpam_lpa4"/>
            <Constant dataType="string">lpa61</Constant>
          </Apply>
          <Constant dataType="double">1</Constant>
          <Constant dataType="double">0</Constant>
        </Apply>
      </Apply>
    </Apply>
  </Apply>
</Apply>
 </DerivedField>
 <DerivedField name="dance" dataType="double" optype="continuous">
<Apply function="if">
  <Apply function="equal">
    <FieldRef field="lpa_lpa5"/>
    <Constant dataType="string">Yes</Constant>
  </Apply>
  <Constant dataType="double">1</Constant>
  <Constant dataType="double">0</Constant>
```

```
</Apply>
 </DerivedField>
 <DerivedField name="dance_t" dataType="double" optype="continuous">
<Apply function="if">
  <Apply function="is.na">
    <FieldRef field="lpat_lpa5"/>
  </Apply>
  <Constant dataType="double">0</Constant>
  <FieldRef field="lpat_lpa5"/>
</Apply>
 </DerivedField>
 <DerivedField name="dance_h" dataType="double" optype="continuous">
<Apply function="if">
  <Apply function="is.na">
    <FieldRef field="lpam_lpa5"/>
  </Apply>
  <Constant dataType="double">0</Constant>
  <Apply function="if">
    <Apply function="equal">
      <FieldRef field="lpam_lpa5"/>
      <Constant dataType="string">lpa15</Constant>
    </Apply>
    <Constant dataType="double">0.2167</Constant>
    <Apply function="if">
      <Apply function="equal">
        <FieldRef field="lpam_lpa5"/>
        <Constant dataType="string">lpa30</Constant>
      </Apply>
      <Constant dataType="double">0.3833</Constant>
      <Apply function="if">
        <Apply function="equal">
          <FieldRef field="lpam_lpa5"/>
          <Constant dataType="string">lpa60</Constant>
        </Apply>
        <Constant dataType="double">0.75</Constant>
        <Apply function="if">
          <Apply function="equal">
            <FieldRef field="lpam_lpa5"/>
            <Constant dataType="string">lpa61</Constant>
          </Apply>
          <Constant dataType="double">1</Constant>
          <Constant dataType="double">0</Constant>
        </Apply>
      </Apply>
    </Apply>
  </Apply>
</Apply>
 </DerivedField>
 <DerivedField name="hexercises" dataType="double" optype="continuous">
<Apply function="if">
  <Apply function="equal">
    <FieldRef field="lpa_lpa6"/>
    <Constant dataType="string">Yes</Constant>
  </Apply>
```

```
  <Constant dataType="double">1</Constant>
  <Constant dataType="double">0</Constant>
</Apply>
 </DerivedField>
 <DerivedField name="hexercises_t" dataType="double" optype="continuous">
<Apply function="if">
  <Apply function="is.na">
    <FieldRef field="lpat_lpa6"/>
  </Apply>
  <Constant dataType="double">0</Constant>
  <FieldRef field="lpat_lpa6"/>
</Apply>
 </DerivedField>
 <DerivedField name="hexercises_h" dataType="double" optype="continuous">
<Apply function="if">
  <Apply function="is.na">
    <FieldRef field="lpam_lpa6"/>
  </Apply>
  <Constant dataType="double">0</Constant>
  <Apply function="if">
    <Apply function="equal">
      <FieldRef field="lpam_lpa6"/>
      <Constant dataType="string">lpa15</Constant>
    </Apply>
    <Constant dataType="double">0.2167</Constant>
    <Apply function="if">
      <Apply function="equal">
        <FieldRef field="lpam_lpa6"/>
        <Constant dataType="string">lpa30</Constant>
      </Apply>
      <Constant dataType="double">0.3833</Constant>
      <Apply function="if">
        <Apply function="equal">
          <FieldRef field="lpam_lpa6"/>
          <Constant dataType="string">lpa60</Constant>
        </Apply>
        <Constant dataType="double">0.75</Constant>
        <Apply function="if">
          <Apply function="equal">
            <FieldRef field="lpam_lpa6"/>
            <Constant dataType="string">lpa61</Constant>
          </Apply>
          <Constant dataType="double">1</Constant>
          <Constant dataType="double">0</Constant>
        </Apply>
      </Apply>
    </Apply>
  </Apply>
</Apply>
 </DerivedField>
 <DerivedField name="hockey" dataType="double" optype="continuous">
<Apply function="if">
  <Apply function="equal">
    <FieldRef field="lpa_lpa7"/>
```

```
    <Constant dataType="string">Yes</Constant>
  </Apply>
  <Constant dataType="double">1</Constant>
  <Constant dataType="double">0</Constant>
</Apply>
 </DerivedField>
 <DerivedField name="hockey_t" dataType="double" optype="continuous">
<Apply function="if">
  <Apply function="is.na">
    <FieldRef field="lpat_lpa7"/>
  </Apply>
  <Constant dataType="double">0</Constant>
  <FieldRef field="lpat_lpa7"/>
</Apply>
 </DerivedField>
 <DerivedField name="hockey_h" dataType="double" optype="continuous">
<Apply function="if">
  <Apply function="is.na">
    <FieldRef field="lpam_lpa7"/>
  </Apply>
  <Constant dataType="double">0</Constant>
  <Apply function="if">
    <Apply function="equal">
      <FieldRef field="lpam_lpa7"/>
      <Constant dataType="string">lpa15</Constant>
    </Apply>
    <Constant dataType="double">0.2167</Constant>
    <Apply function="if">
      <Apply function="equal">
        <FieldRef field="lpam_lpa7"/>
        <Constant dataType="string">lpa30</Constant>
      </Apply>
      <Constant dataType="double">0.3833</Constant>
      <Apply function="if">
        <Apply function="equal">
          <FieldRef field="lpam_lpa7"/>
          <Constant dataType="string">lpa60</Constant>
        </Apply>
        <Constant dataType="double">0.75</Constant>
        <Apply function="if">
          <Apply function="equal">
            <FieldRef field="lpam_lpa7"/>
            <Constant dataType="string">lpa61</Constant>
          </Apply>
          <Constant dataType="double">1</Constant>
          <Constant dataType="double">0</Constant>
        </Apply>
      </Apply>
    </Apply>
  </Apply>
</Apply>
 </DerivedField>
 <DerivedField name="skate" dataType="double" optype="continuous">
<Apply function="if">
```

```
  <Apply function="equal">
    <FieldRef field="lpa_lpa8"/>
    <Constant dataType="string">Yes</Constant>
  </Apply>
  <Constant dataType="double">1</Constant>
  <Constant dataType="double">0</Constant>
</Apply>
 </DerivedField>
 <DerivedField name="skate_t" dataType="double" optype="continuous">
<Apply function="if">
  <Apply function="is.na">
    <FieldRef field="lpat_lpa8"/>
  </Apply>
  <Constant dataType="double">0</Constant>
  <FieldRef field="lpat_lpa8"/>
</Apply>
 </DerivedField>
 <DerivedField name="skate_h" dataType="double" optype="continuous">
<Apply function="if">
  <Apply function="is.na">
    <FieldRef field="lpam_lpa8"/>
  </Apply>
  <Constant dataType="double">0</Constant>
  <Apply function="if">
    <Apply function="equal">
      <FieldRef field="lpam_lpa8"/>
      <Constant dataType="string">lpa15</Constant>
    </Apply>
    <Constant dataType="double">0.2167</Constant>
    <Apply function="if">
      <Apply function="equal">
        <FieldRef field="lpam_lpa8"/>
        <Constant dataType="string">lpa30</Constant>
      </Apply>
      <Constant dataType="double">0.3833</Constant>
      <Apply function="if">
        <Apply function="equal">
          <FieldRef field="lpam_lpa8"/>
          <Constant dataType="string">lpa60</Constant>
        </Apply>
        <Constant dataType="double">0.75</Constant>
        <Apply function="if">
          <Apply function="equal">
            <FieldRef field="lpam_lpa8"/>
            <Constant dataType="string">lpa61</Constant>
          </Apply>
          <Constant dataType="double">1</Constant>
          <Constant dataType="double">0</Constant>
        </Apply>
      </Apply>
    </Apply>
  </Apply>
</Apply>
 </DerivedField>
```

```
 <DerivedField name="inline" dataType="double" optype="continuous">
<Apply function="if">
  <Apply function="equal">
    <FieldRef field="lpa_lpa9"/>
    <Constant dataType="string">Yes</Constant>
  </Apply>
  <Constant dataType="double">1</Constant>
  <Constant dataType="double">0</Constant>
</Apply>
 </DerivedField>
 <DerivedField name="inline_t" dataType="double" optype="continuous">
<Apply function="if">
  <Apply function="is.na">
    <FieldRef field="lpat_lpa9"/>
  </Apply>
  <Constant dataType="double">0</Constant>
  <FieldRef field="lpat_lpa9"/>
</Apply>
 </DerivedField>
 <DerivedField name="inline_h" dataType="double" optype="continuous">
<Apply function="if">
  <Apply function="is.na">
    <FieldRef field="lpam_lpa9"/>
  </Apply>
  <Constant dataType="double">0</Constant>
  <Apply function="if">
    <Apply function="equal">
      <FieldRef field="lpam_lpa9"/>
      <Constant dataType="string">lpa15</Constant>
    </Apply>
    <Constant dataType="double">0.2167</Constant>
    <Apply function="if">
      <Apply function="equal">
        <FieldRef field="lpam_lpa9"/>
        <Constant dataType="string">lpa30</Constant>
      </Apply>
      <Constant dataType="double">0.3833</Constant>
      <Apply function="if">
        <Apply function="equal">
          <FieldRef field="lpam_lpa9"/>
          <Constant dataType="string">lpa60</Constant>
        </Apply>
        <Constant dataType="double">0.75</Constant>
        <Apply function="if">
          <Apply function="equal">
            <FieldRef field="lpat_lpa9"/>
            <Constant dataType="string">lpa61</Constant>
          </Apply>
          <Constant dataType="double">1</Constant>
          <Constant dataType="double">0</Constant>
        </Apply>
      </Apply>
    </Apply>
  </Apply>
```

```
</Apply>
 </DerivedField>
 <DerivedField name="jogrun" dataType="double" optype="continuous">
<Apply function="if">
  <Apply function="equal">
    <FieldRef field="lpa_lpa10"/>
    <Constant dataType="string">Yes</Constant>
  </Apply>
  <Constant dataType="double">1</Constant>
  <Constant dataType="double">0</Constant>
</Apply>
 </DerivedField>
 <DerivedField name="jogrun_t" dataType="double" optype="continuous">
<Apply function="if">
  <Apply function="is.na">
    <FieldRef field="lpat_lpa10"/>
  </Apply>
  <Constant dataType="double">0</Constant>
  <FieldRef field="lpat_lpa10"/>
</Apply>
 </DerivedField>
 <DerivedField name="jogrun_h" dataType="double" optype="continuous">
<Apply function="if">
  <Apply function="is.na">
    <FieldRef field="lpat_lpa10"/>
  </Apply>
  <Constant dataType="double">0</Constant>
  <Apply function="if">
    <Apply function="equal">
      <FieldRef field="lpat_lpa10"/>
      <Constant dataType="string">lpa15</Constant>
    </Apply>
    <Constant dataType="double">0.2167</Constant>
    <Apply function="if">
      <Apply function="equal">
        <FieldRef field="lpat_lpa10"/>
        <Constant dataType="string">lpa30</Constant>
      </Apply>
      <Constant dataType="double">0.3833</Constant>
      <Apply function="if">
        <Apply function="equal">
          <FieldRef field="lpat_lpa10"/>
          <Constant dataType="string">lpa60</Constant>
        </Apply>
        <Constant dataType="double">0.75</Constant>
        <Apply function="if">
          <Apply function="equal">
            <FieldRef field="lpat_lpa10"/>
            <Constant dataType="string">lpa61</Constant>
          </Apply>
          <Constant dataType="double">1</Constant>
          <Constant dataType="double">0</Constant>
        </Apply>
      </Apply>
```

```
    </Apply>
  </Apply>
</Apply>
 </DerivedField>
 <DerivedField name="golf" dataType="double" optype="continuous">
<Apply function="if">
  <Apply function="equal">
    <FieldRef field="lpa_lpa11"/>
    <Constant dataType="string">Yes</Constant>
  </Apply>
  <Constant dataType="double">1</Constant>
  <Constant dataType="double">0</Constant>
</Apply>
 </DerivedField>
 <DerivedField name="golf_t" dataType="double" optype="continuous">
<Apply function="if">
  <Apply function="is.na">
    <FieldRef field="lpat_lpa11"/>
  </Apply>
  <Constant dataType="double">0</Constant>
  <FieldRef field="lpat_lpa11"/>
</Apply>
 </DerivedField>
 <DerivedField name="golf_h" dataType="double" optype="continuous">
<Apply function="if">
  <Apply function="is.na">
    <FieldRef field="lpam_lpa11"/>
  </Apply>
  <Constant dataType="double">0</Constant>
  <Apply function="if">
    <Apply function="equal">
      <FieldRef field="lpam_lpa11"/>
      <Constant dataType="string">lpa15</Constant>
    </Apply>
    <Constant dataType="double">0.2167</Constant>
    <Apply function="if">
      <Apply function="equal">
        <FieldRef field="lpam_lpa11"/>
        <Constant dataType="string">lpa30</Constant>
      </Apply>
      <Constant dataType="double">0.3833</Constant>
      <Apply function="if">
        <Apply function="equal">
          <FieldRef field="lpam_lpa11"/>
          <Constant dataType="string">lpa60</Constant>
        </Apply>
        <Constant dataType="double">0.75</Constant>
        <Apply function="if">
          <Apply function="equal">
            <FieldRef field="lpam_lpa11"/>
            <Constant dataType="string">lpa61</Constant>
          </Apply>
          <Constant dataType="double">1</Constant>
          <Constant dataType="double">0</Constant>
```

```
        </Apply>
      </Apply>
    </Apply>
  </Apply>
</Apply>
 </DerivedField>
 <DerivedField name="aerobics" dataType="double" optype="continuous">
<Apply function="if">
  <Apply function="equal">
    <FieldRef field="lpa_lpa12"/>
    <Constant dataType="string">Yes</Constant>
  </Apply>
  <Constant dataType="double">1</Constant>
  <Constant dataType="double">0</Constant>
</Apply>
 </DerivedField>
 <DerivedField name="aerobics_t" dataType="double" optype="continuous">
<Apply function="if">
  <Apply function="is.na">
    <FieldRef field="lpat_lpa12"/>
  </Apply>
  <Constant dataType="double">0</Constant>
  <FieldRef field="lpat_lpa12"/>
</Apply>
 </DerivedField>
 <DerivedField name="aerobics_h" dataType="double" optype="continuous">
<Apply function="if">
  <Apply function="is.na">
    <FieldRef field="lpam_lpa12"/>
  </Apply>
  <Constant dataType="double">0</Constant>
  <Apply function="if">
    <Apply function="equal">
      <FieldRef field="lpam_lpa12"/>
      <Constant dataType="string">lpa15</Constant>
    </Apply>
    <Constant dataType="double">0.2167</Constant>
    <Apply function="if">
      <Apply function="equal">
        <FieldRef field="lpam_lpa12"/>
        <Constant dataType="string">lpa30</Constant>
      </Apply>
      <Constant dataType="double">0.3833</Constant>
      <Apply function="if">
        <Apply function="equal">
          <FieldRef field="lpam_lpa12"/>
          <Constant dataType="string">lpa60</Constant>
        </Apply>
        <Constant dataType="double">0.75</Constant>
        <Apply function="if">
          <Apply function="equal">
            <FieldRef field="lpam_lpa12"/>
            <Constant dataType="string">lpa61</Constant>
          </Apply>
```

```
          <Constant dataType="double">1</Constant>
          <Constant dataType="double">0</Constant>
        </Apply>
      </Apply>
    </Apply>
  </Apply>
</Apply>
 </DerivedField>
 <DerivedField name="ski" dataType="double" optype="continuous">
<Apply function="if">
  <Apply function="equal">
    <FieldRef field="lpa_lpa13"/>
    <Constant dataType="string">Yes</Constant>
  </Apply>
  <Constant dataType="double">1</Constant>
  <Constant dataType="double">0</Constant>
</Apply>
 </DerivedField>
 <DerivedField name="ski_t" dataType="double" optype="continuous">
<Apply function="if">
  <Apply function="is.na">
    <FieldRef field="lpat_lpa13"/>
  </Apply>
  <Constant dataType="double">0</Constant>
  <FieldRef field="lpat_lpa13"/>
</Apply>
 </DerivedField>
 <DerivedField name="ski_h" dataType="double" optype="continuous">
<Apply function="if">
  <Apply function="is.na">
    <FieldRef field="lpam_lpa13"/>
  </Apply>
  <Constant dataType="double">0</Constant>
  <Apply function="if">
    <Apply function="equal">
      <FieldRef field="lpam_lpa13"/>
      <Constant dataType="string">lpa15</Constant>
    </Apply>
    <Constant dataType="double">0.2167</Constant>
    <Apply function="if">
      <Apply function="equal">
        <FieldRef field="lpam_lpa13"/>
        <Constant dataType="string">lpa30</Constant>
      </Apply>
      <Constant dataType="double">0.3833</Constant>
      <Apply function="if">
        <Apply function="equal">
          <FieldRef field="lpam_lpa13"/>
          <Constant dataType="string">lpa60</Constant>
        </Apply>
        <Constant dataType="double">0.75</Constant>
        <Apply function="if">
          <Apply function="equal">
            <FieldRef field="lpam_lpa13"/>
```

```
            <Constant dataType="string">lpa61</Constant>
          </Apply>
          <Constant dataType="double">1</Constant>
          <Constant dataType="double">0</Constant>
        </Apply>
      </Apply>
    </Apply>
  </Apply>
</Apply>
 </DerivedField>
 <DerivedField name="bowl" dataType="double" optype="continuous">
<Apply function="if">
  <Apply function="equal">
    <FieldRef field="lpa_lpa14"/>
    <Constant dataType="string">Yes</Constant>
  </Apply>
  <Constant dataType="double">1</Constant>
  <Constant dataType="double">0</Constant>
</Apply>
 </DerivedField>
 <DerivedField name="bowl_t" dataType="double" optype="continuous">
<Apply function="if">
  <Apply function="is.na">
    <FieldRef field="lpat_lpa14"/>
  </Apply>
  <Constant dataType="double">0</Constant>
  <FieldRef field="lpat_lpa14"/>
</Apply>
 </DerivedField>
 <DerivedField name="bowl_h" dataType="double" optype="continuous">
<Apply function="if">
  <Apply function="is.na">
    <FieldRef field="lpam_lpa14"/>
  </Apply>
  <Constant dataType="double">0</Constant>
  <Apply function="if">
    <Apply function="equal">
      <FieldRef field="lpam_lpa14"/>
      <Constant dataType="string">lpa15</Constant>
    </Apply>
    <Constant dataType="double">0.2167</Constant>
    <Apply function="if">
      <Apply function="equal">
        <FieldRef field="lpam_lpa14"/>
        <Constant dataType="string">lpa30</Constant>
      </Apply>
      <Constant dataType="double">0.3833</Constant>
      <Apply function="if">
        <Apply function="equal">
          <FieldRef field="lpam_lpa14"/>
          <Constant dataType="string">lpa60</Constant>
        </Apply>
        <Constant dataType="double">0.75</Constant>
        <Apply function="if">
```

```
            <Apply function="equal">
              <FieldRef field="lpam_lpa14"/>
              <Constant dataType="string">lpa61</Constant>
            </Apply>
            <Constant dataType="double">1</Constant>
            <Constant dataType="double">0</Constant>
          </Apply>
        </Apply>
      </Apply>
    </Apply>
  </Apply>
   </DerivedField>
   <DerivedField name="baseball" dataType="double" optype="continuous">
  <Apply function="if">
    <Apply function="equal">
      <FieldRef field="lpa_lpa15"/>
      <Constant dataType="string">Yes</Constant>
    </Apply>
    <Constant dataType="double">1</Constant>
    <Constant dataType="double">0</Constant>
  </Apply>
   </DerivedField>
   <DerivedField name="baseball_t" dataType="double" optype="continuous">
  <Apply function="if">
    <Apply function="is.na">
      <FieldRef field="lpat_lpa15"/>
    </Apply>
    <Constant dataType="double">0</Constant>
    <FieldRef field="lpat_lpa15"/>
  </Apply>
   </DerivedField>
   <DerivedField name="baseball_h" dataType="double" optype="continuous">
  <Apply function="if">
    <Apply function="is.na">
      <FieldRef field="lpam_lpa15"/>
    </Apply>
    <Constant dataType="double">0</Constant>
    <Apply function="if">
      <Apply function="equal">
        <FieldRef field="lpam_lpa15"/>
        <Constant dataType="string">lpa15</Constant>
      </Apply>
      <Constant dataType="double">0.2167</Constant>
      <Apply function="if">
        <Apply function="equal">
          <FieldRef field="lpam_lpa15"/>
          <Constant dataType="string">lpa30</Constant>
        </Apply>
        <Constant dataType="double">0.3833</Constant>
        <Apply function="if">
          <Apply function="equal">
            <FieldRef field="lpam_lpa15"/>
            <Constant dataType="string">lpa60</Constant>
          </Apply>
```

```
        <Constant dataType="double">0.75</Constant>
        <Apply function="if">
          <Apply function="equal">
            <FieldRef field="lpam_lpa15"/>
            <Constant dataType="string">lpa61</Constant>
          </Apply>
          <Constant dataType="double">1</Constant>
          <Constant dataType="double">0</Constant>
        </Apply>
      </Apply>
    </Apply>
  </Apply>
</Apply>
 </DerivedField>
 <DerivedField name="tennis" dataType="double" optype="continuous">
<Apply function="if">
  <Apply function="equal">
    <FieldRef field="lpa_lpa16"/>
    <Constant dataType="string">Yes</Constant>
  </Apply>
  <Constant dataType="double">1</Constant>
  <Constant dataType="double">0</Constant>
</Apply>
 </DerivedField>
 <DerivedField name="tennis_t" dataType="double" optype="continuous">
<Apply function="if">
  <Apply function="is.na">
    <FieldRef field="lpat_lpa16"/>
  </Apply>
  <Constant dataType="double">0</Constant>
  <FieldRef field="lpat_lpa16"/>
</Apply>
 </DerivedField>
 <DerivedField name="tennis_h" dataType="double" optype="continuous">
<Apply function="if">
  <Apply function="is.na">
    <FieldRef field="lpam_lpa16"/>
  </Apply>
  <Constant dataType="double">0</Constant>
  <Apply function="if">
    <Apply function="equal">
      <FieldRef field="lpam_lpa16"/>
      <Constant dataType="string">lpa15</Constant>
    </Apply>
    <Constant dataType="double">0.2167</Constant>
    <Apply function="if">
      <Apply function="equal">
        <FieldRef field="lpam_lpa16"/>
        <Constant dataType="string">lpa30</Constant>
      </Apply>
      <Constant dataType="double">0.3833</Constant>
      <Apply function="if">
        <Apply function="equal">
          <FieldRef field="lpam_lpa16"/>
```

```
          <Constant dataType="string">lpa60</Constant>
        </Apply>
        <Constant dataType="double">0.75</Constant>
        <Apply function="if">
          <Apply function="equal">
            <FieldRef field="lpam_lpa16"/>
            <Constant dataType="string">lpa61</Constant>
          </Apply>
          <Constant dataType="double">1</Constant>
          <Constant dataType="double">0</Constant>
        </Apply>
      </Apply>
    </Apply>
  </Apply>
</Apply>
 </DerivedField>
 <DerivedField name="weights" dataType="double" optype="continuous">
<Apply function="if">
  <Apply function="equal">
    <FieldRef field="lpa_lpa17"/>
    <Constant dataType="string">Yes</Constant>
  </Apply>
  <Constant dataType="double">1</Constant>
  <Constant dataType="double">0</Constant>
</Apply>
 </DerivedField>
 <DerivedField name="weights_t" dataType="double" optype="continuous">
<Apply function="if">
  <Apply function="is.na">
    <FieldRef field="lpat_lpa17"/>
  </Apply>
  <Constant dataType="double">0</Constant>
  <FieldRef field="lpat_lpa17"/>
</Apply>
 </DerivedField>
 <DerivedField name="weights_h" dataType="double" optype="continuous">
<Apply function="if">
  <Apply function="is.na">
    <FieldRef field="lpam_lpa17"/>
  </Apply>
  <Constant dataType="double">0</Constant>
  <Apply function="if">
    <Apply function="equal">
      <FieldRef field="lpam_lpa17"/>
      <Constant dataType="string">lpa15</Constant>
    </Apply>
    <Constant dataType="double">0.2167</Constant>
    <Apply function="if">
      <Apply function="equal">
        <FieldRef field="lpam_lpa17"/>
        <Constant dataType="string">lpa30</Constant>
      </Apply>
      <Constant dataType="double">0.3833</Constant>
      <Apply function="if">
```

```
        <Apply function="equal">
          <FieldRef field="lpam_lpa17"/>
          <Constant dataType="string">lpa60</Constant>
        </Apply>
        <Constant dataType="double">0.75</Constant>
        <Apply function="if">
          <Apply function="equal">
            <FieldRef field="lpam_lpa17"/>
            <Constant dataType="string">lpa61</Constant>
          </Apply>
          <Constant dataType="double">1</Constant>
          <Constant dataType="double">0</Constant>
        </Apply>
      </Apply>
    </Apply>
  </Apply>
</Apply>
 </DerivedField>
 <DerivedField name="fishing" dataType="double" optype="continuous">
<Apply function="if">
  <Apply function="equal">
    <FieldRef field="lpa_lpa18"/>
    <Constant dataType="string">Yes</Constant>
  </Apply>
  <Constant dataType="double">1</Constant>
  <Constant dataType="double">0</Constant>
</Apply>
 </DerivedField>
 <DerivedField name="fishing_t" dataType="double" optype="continuous">
<Apply function="if">
  <Apply function="is.na">
    <FieldRef field="lpat_lpa18"/>
  </Apply>
  <Constant dataType="double">0</Constant>
  <FieldRef field="lpat_lpa18"/>
</Apply>
 </DerivedField>
 <DerivedField name="fishing_h" dataType="double" optype="continuous">
<Apply function="if">
  <Apply function="is.na">
    <FieldRef field="lpam_lpa18"/>
  </Apply>
  <Constant dataType="double">0</Constant>
  <Apply function="if">
    <Apply function="equal">
      <FieldRef field="lpam_lpa18"/>
      <Constant dataType="string">lpa15</Constant>
    </Apply>
    <Constant dataType="double">0.2167</Constant>
    <Apply function="if">
      <Apply function="equal">
        <FieldRef field="lpam_lpa18"/>
        <Constant dataType="string">lpa30</Constant>
      </Apply>
```

```
      <Constant dataType="double">0.3833</Constant>
      <Apply function="if">
        <Apply function="equal">
          <FieldRef field="lpam_lpa18"/>
          <Constant dataType="string">lpa60</Constant>
        </Apply>
        <Constant dataType="double">0.75</Constant>
        <Apply function="if">
          <Apply function="equal">
            <FieldRef field="lpam_lpa18"/>
            <Constant dataType="string">lpa61</Constant>
          </Apply>
          <Constant dataType="double">1</Constant>
          <Constant dataType="double">0</Constant>
        </Apply>
      </Apply>
    </Apply>
  </Apply>
</Apply>
 </DerivedField>
 <DerivedField name="volleyball" dataType="double" optype="continuous">
<Apply function="if">
  <Apply function="equal">
    <FieldRef field="lpa_lpa19"/>
    <Constant dataType="string">Yes</Constant>
  </Apply>
  <Constant dataType="double">1</Constant>
  <Constant dataType="double">0</Constant>
</Apply>
 </DerivedField>
 <DerivedField name="volleyball_t" dataType="double" optype="continuous">
<Apply function="if">
  <Apply function="is.na">
    <FieldRef field="lpat_lpa19"/>
  </Apply>
  <Constant dataType="double">0</Constant>
  <FieldRef field="lpat_lpa19"/>
</Apply>
 </DerivedField>
 <DerivedField name="volleyball_h" dataType="double" optype="continuous">
<Apply function="if">
  <Apply function="is.na">
    <FieldRef field="lpam_lpa19"/>
  </Apply>
  <Constant dataType="double">0</Constant>
  <Apply function="if">
    <Apply function="equal">
      <FieldRef field="lpam_lpa19"/>
      <Constant dataType="string">lpa15</Constant>
    </Apply>
    <Constant dataType="double">0.2167</Constant>
    <Apply function="if">
      <Apply function="equal">
        <FieldRef field="lpam_lpa19"/>
```

```
        <Constant dataType="string">lpa30</Constant>
      </Apply>
      <Constant dataType="double">0.3833</Constant>
      <Apply function="if">
        <Apply function="equal">
          <FieldRef field="lpam_lpa19"/>
          <Constant dataType="string">lpa60</Constant>
        </Apply>
        <Constant dataType="double">0.75</Constant>
        <Apply function="if">
          <Apply function="equal">
            <FieldRef field="lpam_lpa19"/>
            <Constant dataType="string">lpa61</Constant>
          </Apply>
          <Constant dataType="double">1</Constant>
          <Constant dataType="double">0</Constant>
        </Apply>
      </Apply>
    </Apply>
  </Apply>
</Apply>
 </DerivedField>
 <DerivedField name="basketball" dataType="double" optype="continuous">
<Apply function="if">
  <Apply function="equal">
    <FieldRef field="lpa_lpa20"/>
    <Constant dataType="string">Yes</Constant>
  </Apply>
  <Constant dataType="double">1</Constant>
  <Constant dataType="double">0</Constant>
</Apply>
 </DerivedField>
 <DerivedField name="basketball_t" dataType="double" optype="continuous">
<Apply function="if">
  <Apply function="is.na">
    <FieldRef field="lpat_lpa20"/>
  </Apply>
  <Constant dataType="double">0</Constant>
  <FieldRef field="lpat_lpa20"/>
</Apply>
 </DerivedField>
 <DerivedField name="basketball_h" dataType="double" optype="continuous">
<Apply function="if">
  <Apply function="is.na">
    <FieldRef field="lpam_lpa20"/>
  </Apply>
  <Constant dataType="double">0</Constant>
  <Apply function="if">
    <Apply function="equal">
      <FieldRef field="lpam_lpa20"/>
      <Constant dataType="string">lpa15</Constant>
    </Apply>
    <Constant dataType="double">0.2167</Constant>
    <Apply function="if">
```

```
        <Apply function="equal">
          <FieldRef field="lpam_lpa20"/>
          <Constant dataType="string">lpa30</Constant>
        </Apply>
        <Constant dataType="double">0.3833</Constant>
        <Apply function="if">
          <Apply function="equal">
            <FieldRef field="lpam_lpa20"/>
            <Constant dataType="string">lpa60</Constant>
          </Apply>
          <Constant dataType="double">0.75</Constant>
          <Apply function="if">
            <Apply function="equal">
              <FieldRef field="lpam_lpa20"/>
              <Constant dataType="string">lpa61</Constant>
            </Apply>
            <Constant dataType="double">1</Constant>
            <Constant dataType="double">0</Constant>
          </Apply>
        </Apply>
      </Apply>
    </Apply>
  </Apply>
 </DerivedField>
 <DerivedField name="soccer" dataType="double" optype="continuous">
<Apply function="if">
  <Apply function="equal">
    <FieldRef field="lpa_lpa21"/>
    <Constant dataType="string">Yes</Constant>
  </Apply>
  <Constant dataType="double">1</Constant>
  <Constant dataType="double">0</Constant>
</Apply>
 </DerivedField>
 <DerivedField name="soccer_t" dataType="double" optype="continuous">
<Apply function="if">
  <Apply function="is.na">
    <FieldRef field="lpat_lpa21"/>
  </Apply>
  <Constant dataType="double">0</Constant>
  <FieldRef field="lpat_lpa21"/>
</Apply>
 </DerivedField>
 <DerivedField name="soccer_h" dataType="double" optype="continuous">
<Apply function="if">
  <Apply function="is.na">
    <FieldRef field="lpam_lpa21"/>
  </Apply>
  <Constant dataType="double">0</Constant>
  <Apply function="if">
    <Apply function="equal">
      <FieldRef field="lpam_lpa21"/>
      <Constant dataType="string">lpa15</Constant>
    </Apply>
```

```
    <Constant dataType="double">0.2167</Constant>
    <Apply function="if">
      <Apply function="equal">
        <FieldRef field="lpam_lpa21"/>
        <Constant dataType="string">lpa30</Constant>
      </Apply>
      <Constant dataType="double">0.3833</Constant>
      <Apply function="if">
        <Apply function="equal">
          <FieldRef field="lpam_lpa21"/>
          <Constant dataType="string">lpa60</Constant>
        </Apply>
        <Constant dataType="double">0.75</Constant>
        <Apply function="if">
          <Apply function="equal">
            <FieldRef field="lpam_lpa21"/>
            <Constant dataType="string">lpa61</Constant>
          </Apply>
          <Constant dataType="double">1</Constant>
          <Constant dataType="double">0</Constant>
        </Apply>
      </Apply>
    </Apply>
  </Apply>
</Apply>
 </DerivedField>
 <DerivedField name="other" dataType="double" optype="continuous">
<Apply function="if">
  <Apply function="equal">
    <FieldRef field="lpa_lpa22"/>
    <Constant dataType="string">Yes</Constant>
  </Apply>
  <Constant dataType="double">1</Constant>
  <Constant dataType="double">0</Constant>
</Apply>
 </DerivedField>
 <DerivedField name="other_t" dataType="double" optype="continuous">
<Apply function="if">
  <Apply function="is.na">
    <FieldRef field="lpat_lpa22"/>
  </Apply>
  <Constant dataType="double">0</Constant>
  <FieldRef field="lpat_lpa22"/>
</Apply>
 </DerivedField>
 <DerivedField name="other_h" dataType="double" optype="continuous">
<Apply function="if">
  <Apply function="is.na">
    <FieldRef field="lpam_lpa22"/>
  </Apply>
  <Constant dataType="double">0</Constant>
  <Apply function="if">
    <Apply function="equal">
      <FieldRef field="lpam_lpa22"/>
```

```
      <Constant dataType="string">lpa15</Constant>
    </Apply>
    <Constant dataType="double">0.2167</Constant>
    <Apply function="if">
      <Apply function="equal">
        <FieldRef field="lpat_lpa22"/>
        <Constant dataType="string">lpa30</Constant>
      </Apply>
      <Constant dataType="double">0.3833</Constant>
      <Apply function="if">
        <Apply function="equal">
          <FieldRef field="lpam_lpa22"/>
          <Constant dataType="string">lpa60</Constant>
        </Apply>
        <Constant dataType="double">0.75</Constant>
        <Apply function="if">
          <Apply function="equal">
            <FieldRef field="lpam_lpa22"/>
            <Constant dataType="string">lpa61</Constant>
          </Apply>
          <Constant dataType="double">1</Constant>
          <Constant dataType="double">0</Constant>
        </Apply>
      </Apply>
    </Apply>
  </Apply>
</Apply>
 </DerivedField>
 <DerivedField name="PhysicalActivityraw1" dataType="double"
optype="continuous">
<Apply function="+">
  <Apply function="+">
    <Apply function="+">
      <Apply function="+">
        <Apply function="+">
          <Apply function="+">
            <Apply function="+">
              <Apply function="+">
                <Apply function="+">
                  <Apply function="+">
                    <Apply function="+">
                      <Apply function="+">
                        <Apply function="+">
                          <Apply function="+">
                            <Apply function="+">
                              <Apply function="+">
                                <Apply function="+">
                                  <Apply function="+">
                                    <Apply function="+">
                                      <Apply function="+">
                                        <Apply function="+">
                                          <Apply function="/">
                                            <Apply function="*">
                                              <Apply function="*">
```

```
                              <Apply function="*">
                                <FieldRef
field="walking"/>
                                <FieldRef
field="walking_h"/>
                              </Apply>
                              <Constant
dataType="double">3</Constant>
                            </Apply>
                            <FieldRef
field="walking_t"/>
                          </Apply>
                          <Constant
dataType="double">90</Constant>
                        </Apply>
                        <Apply function="/">
                          <Apply function="*">
                            <Apply function="*">
                              <Apply function="*">
                                <FieldRef
field="garden"/>
                                <FieldRef
field="garden_h"/>
                              </Apply>
                              <Constant
dataType="double">3</Constant>
                            </Apply>
                            <FieldRef
field="garden_t"/>
                          </Apply>
                          <Constant
dataType="double">90</Constant>
                        </Apply>
                      </Apply>
                      <Apply function="/">
                        <Apply function="*">
                          <Apply function="*">
                            <Apply function="*">
                              <FieldRef field="swim"/>
                              <FieldRef
field="swim_h"/>
                            </Apply>
                            <Constant
dataType="double">3</Constant>
                          </Apply>
                          <FieldRef field="swim_t"/>
                        </Apply>
                        <Constant
dataType="double">90</Constant>
                      </Apply>
                    </Apply>
                    <Apply function="/">
                      <Apply function="*">
                        <Apply function="*">
```

```
                                            <Apply function="*">
                                              <FieldRef field="bike"/>
                                              <FieldRef field="bike_h"/>
                                            </Apply>
                                            <Constant
dataType="double">4</Constant>
                                          </Apply>
                                          <FieldRef field="bike_t"/>
                                        </Apply>
                                        <Constant
dataType="double">90</Constant>
                                      </Apply>
                                    </Apply>
                                    <Apply function="/">
                                      <Apply function="*">
                                        <Apply function="*">
                                          <Apply function="*">
                                            <FieldRef field="dance"/>
                                            <FieldRef field="dance_h"/>
                                          </Apply>
                                          <Constant
dataType="double">3</Constant>
                                        </Apply>
                                        <FieldRef field="dance_t"/>
                                      </Apply>
                                      <Constant
dataType="double">90</Constant>
                                    </Apply>
                                  </Apply>
                                  <Apply function="/">
                                    <Apply function="*">
                                      <Apply function="*">
                                        <Apply function="*">
                                          <FieldRef field="hexercises"/>
                                          <FieldRef
field="hexercises_h"/>
                                        </Apply>
                                        <Constant
dataType="double">3</Constant>
                                      </Apply>
                                      <FieldRef field="hexercises_t"/>
                                    </Apply>
                                    <Constant
dataType="double">90</Constant>
                                  </Apply>
                                </Apply>
                                <Apply function="/">
                                  <Apply function="*">
                                    <Apply function="*">
                                      <Apply function="*">
                                        <FieldRef field="hockey"/>
                                        <FieldRef field="hockey_h"/>
                                      </Apply>
```

```
                                      <Constant
dataType="double">6</Constant>
                                    </Apply>
                                    <FieldRef field="hockey_t"/>
                                  </Apply>
                                  <Constant
dataType="double">90</Constant>
                                </Apply>
                              </Apply>
                              <Apply function="/">
                                <Apply function="*">
                                  <Apply function="*">
                                    <Apply function="*">
                                      <FieldRef field="skate"/>
                                      <FieldRef field="skate_h"/>
                                    </Apply>
                                    <Constant
dataType="double">4</Constant>
                                  </Apply>
                                  <FieldRef field="skate_t"/>
                                </Apply>
                                <Constant dataType="double">90</Constant>
                              </Apply>
                            </Apply>
                            <Apply function="/">
                              <Apply function="*">
                                <Apply function="*">
                                  <Apply function="*">
                                    <FieldRef field="inline"/>
                                    <FieldRef field="inline_h"/>
                                  </Apply>
                                  <Constant
dataType="double">5</Constant>
                                </Apply>
                                <FieldRef field="inline_t"/>
                              </Apply>
                              <Constant dataType="double">90</Constant>
                            </Apply>
                          </Apply>
                          <Apply function="/">
                            <Apply function="*">
                              <Apply function="*">
                                <Apply function="*">
                                  <FieldRef field="jogrun"/>
                                  <FieldRef field="jogrun_h"/>
                                </Apply>
                                <Constant
dataType="double">9.5</Constant>
                              </Apply>
                              <FieldRef field="jogrun_t"/>
                            </Apply>
                            <Constant dataType="double">90</Constant>
                          </Apply>
                        </Apply>
```

```
        <Apply function="/">
          <Apply function="*">
            <Apply function="*">
              <Apply function="*">
                <FieldRef field="golf"/>
                <FieldRef field="golf_h"/>
              </Apply>
              <Constant dataType="double">4</Constant>
            </Apply>
            <FieldRef field="golf_t"/>
          </Apply>
          <Constant dataType="double">90</Constant>
        </Apply>
      </Apply>
      <Apply function="/">
        <Apply function="*">
          <Apply function="*">
            <Apply function="*">
              <FieldRef field="aerobics"/>
              <FieldRef field="aerobics_h"/>
            </Apply>
            <Constant dataType="double">4</Constant>
          </Apply>
          <FieldRef field="aerobics_t"/>
        </Apply>
        <Constant dataType="double">90</Constant>
      </Apply>
    </Apply>
    <Apply function="/">
      <Apply function="*">
        <Apply function="*">
          <Apply function="*">
            <FieldRef field="ski"/>
            <FieldRef field="ski_h"/>
          </Apply>
          <Constant dataType="double">4</Constant>
        </Apply>
        <FieldRef field="ski_t"/>
      </Apply>
      <Constant dataType="double">90</Constant>
    </Apply>
  </Apply>
  <Apply function="/">
    <Apply function="*">
      <Apply function="*">
        <Apply function="*">
          <FieldRef field="bowl"/>
          <FieldRef field="bowl_h"/>
        </Apply>
        <Constant dataType="double">2</Constant>
      </Apply>
      <FieldRef field="bowl_t"/>
    </Apply>
    <Constant dataType="double">90</Constant>
```

```
          </Apply>
        </Apply>
        <Apply function="/">
          <Apply function="*">
            <Apply function="*">
              <Apply function="*">
                <FieldRef field="baseball"/>
                <FieldRef field="baseball_h"/>
              </Apply>
              <Constant dataType="double">3</Constant>
            </Apply>
            <FieldRef field="baseball_t"/>
          </Apply>
          <Constant dataType="double">90</Constant>
        </Apply>
      </Apply>
      <Apply function="/">
        <Apply function="*">
          <Apply function="*">
            <Apply function="*">
              <FieldRef field="tennis"/>
              <FieldRef field="tennis_h"/>
            </Apply>
            <Constant dataType="double">4</Constant>
          </Apply>
          <FieldRef field="tennis_t"/>
        </Apply>
        <Constant dataType="double">90</Constant>
      </Apply>
    </Apply>
    <Apply function="/">
      <Apply function="*">
        <Apply function="*">
          <Apply function="*">
            <FieldRef field="weights"/>
            <FieldRef field="weights_h"/>
          </Apply>
          <Constant dataType="double">3</Constant>
        </Apply>
        <FieldRef field="weights_t"/>
      </Apply>
      <Constant dataType="double">90</Constant>
    </Apply>
  </Apply>
  <Apply function="/">
    <Apply function="*">
      <Apply function="*">
        <Apply function="*">
          <FieldRef field="fishing"/>
          <FieldRef field="fishing_h"/>
        </Apply>
        <Constant dataType="double">3</Constant>
      </Apply>
      <FieldRef field="fishing_t"/>
```

```
            &lt;/Apply&gt;
            &lt;Constant dataType="double"&gt;90&lt;/Constant&gt;
          &lt;/Apply&gt;
        &lt;/Apply&gt;
        &lt;Apply function="/"&gt;
          &lt;Apply function="*"&gt;
            &lt;Apply function="*"&gt;
              &lt;Apply function="*"&gt;
                &lt;FieldRef field="volleyball"/&gt;
                &lt;FieldRef field="volleyball_h"/&gt;
              &lt;/Apply&gt;
              &lt;Constant dataType="double"&gt;5&lt;/Constant&gt;
            &lt;/Apply&gt;
            &lt;FieldRef field="volleyball_t"/&gt;
          &lt;/Apply&gt;
          &lt;Constant dataType="double"&gt;90&lt;/Constant&gt;
        &lt;/Apply&gt;
      &lt;/Apply&gt;
      &lt;Apply function="/"&gt;
        &lt;Apply function="*"&gt;
          &lt;Apply function="*"&gt;
            &lt;Apply function="*"&gt;
              &lt;FieldRef field="basketball"/&gt;
              &lt;FieldRef field="basketball_h"/&gt;
            &lt;/Apply&gt;
            &lt;Constant dataType="double"&gt;6&lt;/Constant&gt;
          &lt;/Apply&gt;
          &lt;FieldRef field="basketball_t"/&gt;
        &lt;/Apply&gt;
        &lt;Constant dataType="double"&gt;90&lt;/Constant&gt;
      &lt;/Apply&gt;
    &lt;/Apply&gt;
    &lt;Apply function="/"&gt;
      &lt;Apply function="*"&gt;
        &lt;Apply function="*"&gt;
          &lt;Apply function="*"&gt;
            &lt;FieldRef field="soccer"/&gt;
            &lt;FieldRef field="soccer_h"/&gt;
          &lt;/Apply&gt;
          &lt;Constant dataType="double"&gt;5&lt;/Constant&gt;
        &lt;/Apply&gt;
        &lt;FieldRef field="soccer_t"/&gt;
      &lt;/Apply&gt;
      &lt;Constant dataType="double"&gt;90&lt;/Constant&gt;
    &lt;/Apply&gt;
  &lt;/Apply&gt;
  &lt;Apply function="/"&gt;
    &lt;Apply function="*"&gt;
      &lt;Apply function="*"&gt;
        &lt;Apply function="*"&gt;
          &lt;FieldRef field="other"/&gt;
          &lt;FieldRef field="other_h"/&gt;
        &lt;/Apply&gt;
        &lt;Constant dataType="double"&gt;4&lt;/Constant&gt;
```

```
      </Apply>
      <FieldRef field="other_t"/>
    </Apply>
    <Constant dataType="double">90</Constant>
  </Apply>
</Apply>
 </DerivedField>
 <DerivedField name="PhysicalActivityraw2" dataType="double"
optype="continuous">
<Apply function="if">
  <Apply function="greaterThan">
    <FieldRef field="PhysicalActivityraw1"/>
    <Constant dataType="double">10</Constant>
  </Apply>
  <Constant dataType="double">10</Constant>
  <FieldRef field="PhysicalActivityraw1"/>
</Apply>
 </DerivedField>
 <DerivedField name="PhysicalActivity_cont" dataType="double"
optype="continuous">
<Apply function="ln">
  <FieldRef field="PhysicalActivityraw2"/>
</Apply>
 </DerivedField>
 <DerivedField name="weeklyalc" dataType="double" optype="continuous">
<Apply function="if">
  <Apply function="equal">
    <FieldRef field="dev"/>
    <Constant dataType="string">dev2</Constant>
  </Apply>
  <FieldRef field="NA"/>
  <Apply function="if">
    <Apply function="and">
      <Apply function="equal">
        <FieldRef field="dev"/>
        <Constant dataType="string">dev1</Constant>
      </Apply>
      <Apply function="equal">
        <FieldRef field="dany"/>
        <Constant dataType="string">dany1</Constant>
      </Apply>
    </Apply>
    <Apply function="+">
      <Apply function="+">
        <Apply function="+">
          <Apply function="+">
            <Apply function="+">
              <Apply function="+">
                <FieldRef field="drk_drkm"/>
                <FieldRef field="drk_drkt"/>
              </Apply>
              <FieldRef field="drk_drkw"/>
            </Apply>
            <FieldRef field="drk_drkr"/>
```

```
          </Apply>
          <FieldRef field="drk_drkf"/>
        </Apply>
        <FieldRef field="drk_drksa"/>
      </Apply>
      <FieldRef field="drk_drksu"/>
    </Apply>
    <Constant dataType="double">0</Constant>
  </Apply>
</Apply>
 </DerivedField>
 <DerivedField name="bingeflag" dataType="double" optype="continuous">
<Apply function="if">
  <Apply function="equal">
    <FieldRef field="dev"/>
    <Constant dataType="string">dev2</Constant>
  </Apply>
  <FieldRef field="NA"/>
  <Apply function="if">
    <Apply function="and">
      <Apply function="not">
        <Apply function="is.na">
          <FieldRef field="db"/>
        </Apply>
      </Apply>
      <Apply function="or">
        <Apply function="equal">
          <FieldRef field="db"/>
          <Constant dataType="string">db5</Constant>
        </Apply>
        <Apply function="equal">
          <FieldRef field="db"/>
          <Constant dataType="string">db6</Constant>
        </Apply>
      </Apply>
    </Apply>
    <Constant dataType="double">1</Constant>
    <Apply function="if">
      <Apply function="and">
        <Apply function="not">
          <Apply function="is.na">
            <FieldRef field="drk_drkm"/>
          </Apply>
        </Apply>
        <Apply function="greaterOrEqual">
          <FieldRef field="drk_drkm"/>
          <Constant dataType="double">5</Constant>
        </Apply>
      </Apply>
      <Constant dataType="double">1</Constant>
      <Apply function="if">
        <Apply function="and">
          <Apply function="not">
            <Apply function="is.na">
```

```
      &lt;FieldRef field="drk_drkt"/&gt;
    &lt;/Apply&gt;
  &lt;/Apply&gt;
  &lt;FieldRef field="drk_drkt"/&gt;
&lt;/Apply&gt;
&lt;Constant dataType="double"&gt;1&lt;/Constant&gt;
&lt;Apply function="if"&gt;
  &lt;Apply function="and"&gt;
    &lt;Apply function="not"&gt;
      &lt;Apply function="is.na"&gt;
        &lt;FieldRef field="drk_drkw"/&gt;
      &lt;/Apply&gt;
    &lt;/Apply&gt;
    &lt;Apply function="greaterOrEqual"&gt;
      &lt;FieldRef field="drk_drkw"/&gt;
      &lt;Constant dataType="double"&gt;5&lt;/Constant&gt;
    &lt;/Apply&gt;
  &lt;/Apply&gt;
  &lt;Constant dataType="double"&gt;1&lt;/Constant&gt;
  &lt;Apply function="if"&gt;
    &lt;Apply function="and"&gt;
      &lt;Apply function="not"&gt;
        &lt;Apply function="is.na"&gt;
          &lt;FieldRef field="drk_drkr"/&gt;
        &lt;/Apply&gt;
      &lt;/Apply&gt;
      &lt;Apply function="greaterOrEqual"&gt;
        &lt;FieldRef field="drk_drkr"/&gt;
        &lt;Constant dataType="double"&gt;5&lt;/Constant&gt;
      &lt;/Apply&gt;
    &lt;/Apply&gt;
    &lt;Constant dataType="double"&gt;1&lt;/Constant&gt;
    &lt;Apply function="if"&gt;
      &lt;Apply function="and"&gt;
        &lt;Apply function="not"&gt;
          &lt;Apply function="is.na"&gt;
            &lt;FieldRef field="drk_drkf"/&gt;
          &lt;/Apply&gt;
        &lt;/Apply&gt;
        &lt;Apply function="greaterOrEqual"&gt;
          &lt;FieldRef field="drk_drkf"/&gt;
          &lt;Constant dataType="double"&gt;5&lt;/Constant&gt;
        &lt;/Apply&gt;
      &lt;/Apply&gt;
      &lt;Constant dataType="double"&gt;1&lt;/Constant&gt;
      &lt;Apply function="if"&gt;
        &lt;Apply function="and"&gt;
          &lt;Apply function="not"&gt;
            &lt;Apply function="is.na"&gt;
              &lt;FieldRef field="drk_drksa"/&gt;
            &lt;/Apply&gt;
          &lt;/Apply&gt;
          &lt;Apply function="greaterOrEqual"&gt;
            &lt;FieldRef field="drk_drksa"/&gt;
```

```
                    <Constant dataType="double">5</Constant>
                  </Apply>
                </Apply>
                <Constant dataType="double">1</Constant>
                <Apply function="if">
                  <Apply function="and">
                    <Apply function="not">
                      <Apply function="is.na">
                        <FieldRef field="drk_drksu"/>
                      </Apply>
                    </Apply>
                    <Apply function="greaterOrEqual">
                      <FieldRef field="drk_drksu"/>
                      <Constant dataType="double">5</Constant>
                    </Apply>
                  </Apply>
                  <Constant dataType="double">1</Constant>
                  <FieldRef field="NA"/>
                </Apply>
              </Apply>
            </Apply>
          </Apply>
        </Apply>
      </Apply>
    </Apply>
  </Apply>
</Apply>
 </DerivedField>
 <DerivedField name="AlcoholMod_cat" dataType="double"
optype="continuous">
<Apply function="if">
  <Apply function="and">
    <Apply function="not">
      <Apply function="is.na">
        <FieldRef field="bingeflag"/>
      </Apply>
    </Apply>
    <Apply function="equal">
      <FieldRef field="bingeflag"/>
      <Constant dataType="double">1</Constant>
    </Apply>
  </Apply>
  <Constant dataType="double">0</Constant>
  <Apply function="if">
    <Apply function="and">
      <Apply function="and">
        <Apply function="and">
          <Apply function="and">
            <Apply function="equal">
              <FieldRef field="Sex"/>
              <Constant dataType="double">0</Constant>
            </Apply>
            <Apply function="equal">
              <FieldRef field="dev"/>
```

```
            <Constant dataType="string">dev1</Constant>
          </Apply>
        </Apply>
        <Apply function="equal">
          <FieldRef field="dany"/>
          <Constant dataType="string">dany1</Constant>
        </Apply>
      </Apply>
      <Apply function="greaterThan">
        <FieldRef field="weeklyalc"/>
        <Constant dataType="double">3</Constant>
      </Apply>
    </Apply>
    <Apply function="lessOrEqual">
      <FieldRef field="weeklyalc"/>
      <Constant dataType="double">21</Constant>
    </Apply>
  </Apply>
  <Constant dataType="double">1</Constant>
  <Apply function="if">
    <Apply function="and">
      <Apply function="and">
        <Apply function="and">
          <Apply function="and">
            <Apply function="equal">
              <FieldRef field="Sex"/>
              <Constant dataType="double">1</Constant>
            </Apply>
            <Apply function="equal">
              <FieldRef field="dev"/>
              <Constant dataType="string">dev1</Constant>
            </Apply>
          </Apply>
          <Apply function="equal">
            <FieldRef field="dany"/>
            <Constant dataType="string">dany1</Constant>
          </Apply>
        </Apply>
        <Apply function="greaterThan">
          <FieldRef field="weeklyalc"/>
          <Constant dataType="double">2</Constant>
        </Apply>
      </Apply>
      <Apply function="lessOrEqual">
        <FieldRef field="weeklyalc"/>
        <Constant dataType="double">14</Constant>
      </Apply>
    </Apply>
    <Constant dataType="double">1</Constant>
    <Apply function="if">
      <Apply function="and">
        <Apply function="and">
          <Apply function="and">
            <Apply function="equal">
```

```
        <FieldRef field="Sex"/>
        <Constant dataType="double">0</Constant>
      </Apply>
      <Apply function="equal">
        <FieldRef field="dev"/>
        <Constant dataType="string">dev1</Constant>
      </Apply>
    </Apply>
    <Apply function="equal">
      <FieldRef field="dany"/>
      <Constant dataType="string">dany1</Constant>
    </Apply>
  </Apply>
  <Apply function="lessOrEqual">
    <FieldRef field="weeklyalc"/>
    <Constant dataType="double">3</Constant>
  </Apply>
</Apply>
<Constant dataType="double">0</Constant>
<Apply function="if">
  <Apply function="and">
    <Apply function="and">
      <Apply function="and">
        <Apply function="equal">
          <FieldRef field="Sex"/>
          <Constant dataType="double">1</Constant>
        </Apply>
        <Apply function="equal">
          <FieldRef field="dev"/>
          <Constant dataType="string">dev1</Constant>
        </Apply>
      </Apply>
      <Apply function="equal">
        <FieldRef field="dany"/>
        <Constant dataType="string">dany1</Constant>
      </Apply>
    </Apply>
    <Apply function="lessOrEqual">
      <FieldRef field="weeklyalc"/>
      <Constant dataType="double">2</Constant>
    </Apply>
  </Apply>
  <Constant dataType="double">0</Constant>
  <Apply function="if">
    <Apply function="and">
      <Apply function="and">
        <Apply function="and">
          <Apply function="equal">
            <FieldRef field="Sex"/>
            <Constant dataType="double">0</Constant>
          </Apply>
          <Apply function="equal">
            <FieldRef field="dev"/>
            <Constant dataType="string">dev1</Constant>
```

```
          </Apply>
        </Apply>
        <Apply function="equal">
          <FieldRef field="dany"/>
          <Constant dataType="string">dany1</Constant>
        </Apply>
      </Apply>
      <Apply function="greaterThan">
        <FieldRef field="weeklyalc"/>
        <Constant dataType="double">21</Constant>
      </Apply>
    </Apply>
    <Constant dataType="double">0</Constant>
    <Apply function="if">
      <Apply function="and">
        <Apply function="and">
          <Apply function="and">
            <Apply function="equal">
              <FieldRef field="Sex"/>
              <Constant dataType="double">1</Constant>
            </Apply>
            <Apply function="equal">
              <FieldRef field="dev"/>
              <Constant dataType="string">dev1</Constant>
            </Apply>
          </Apply>
          <Apply function="equal">
            <FieldRef field="dany"/>
            <Constant dataType="string">dany1</Constant>
          </Apply>
        </Apply>
        <Apply function="greaterThan">
          <FieldRef field="weeklyalc"/>
          <Constant dataType="double">14</Constant>
        </Apply>
      </Apply>
      <Constant dataType="double">0</Constant>
      <Apply function="if">
        <Apply function="or">
          <Apply function="equal">
            <FieldRef field="dev"/>
            <Constant dataType="string">dev2</Constant>
          </Apply>
          <Apply function="equal">
            <FieldRef field="dany"/>
            <Constant dataType="string">dany2</Constant>
          </Apply>
        </Apply>
        <Constant dataType="double">0</Constant>
        <FieldRef field="NA"/>
      </Apply>
    </Apply>
  </Apply>
</Apply>
```

```
      &lt;/Apply&gt;
    &lt;/Apply&gt;
  &lt;/Apply&gt;
&lt;/Apply&gt;
 &lt;/DerivedField&gt;
 <DerivedField name="AlcoholHeavy_cat" dataType="double"
optype="continuous">
&lt;Apply function="if"&gt;
  &lt;Apply function="or"&gt;
    &lt;Apply function="and"&gt;
      &lt;Apply function="not"&gt;
        &lt;Apply function="is.na"&gt;
          &lt;FieldRef field="bingeflag"/&gt;
        &lt;/Apply&gt;
      &lt;/Apply&gt;
      &lt;Apply function="equal"&gt;
        &lt;FieldRef field="bingeflag"/&gt;
        &lt;Constant dataType="double"&gt;1&lt;/Constant&gt;
      &lt;/Apply&gt;
    &lt;/Apply&gt;
    &lt;Apply function="and"&gt;
      &lt;Apply function="and"&gt;
        &lt;Apply function="and"&gt;
          &lt;Apply function="equal"&gt;
            &lt;FieldRef field="Sex"/&gt;
            &lt;Constant dataType="double"&gt;0&lt;/Constant&gt;
          &lt;/Apply&gt;
          &lt;Apply function="equal"&gt;
            &lt;FieldRef field="dev"/&gt;
            &lt;Constant dataType="string"&gt;dev1&lt;/Constant&gt;
          &lt;/Apply&gt;
        &lt;/Apply&gt;
        &lt;Apply function="equal"&gt;
          &lt;FieldRef field="dany"/&gt;
          &lt;Constant dataType="string"&gt;dany1&lt;/Constant&gt;
        &lt;/Apply&gt;
      &lt;/Apply&gt;
      &lt;Apply function="greaterThan"&gt;
        &lt;FieldRef field="weeklyalc"/&gt;
        &lt;Constant dataType="double"&gt;21&lt;/Constant&gt;
      &lt;/Apply&gt;
    &lt;/Apply&gt;
  &lt;/Apply&gt;
  &lt;Constant dataType="double"&gt;1&lt;/Constant&gt;
  &lt;Apply function="if"&gt;
    &lt;Apply function="and"&gt;
      &lt;Apply function="and"&gt;
        &lt;Apply function="and"&gt;
          &lt;Apply function="equal"&gt;
            &lt;FieldRef field="Sex"/&gt;
            &lt;Constant dataType="double"&gt;1&lt;/Constant&gt;
          &lt;/Apply&gt;
          &lt;Apply function="equal"&gt;
            &lt;FieldRef field="dev"/&gt;
```

```
        <Constant dataType="string">dev1</Constant>
      </Apply>
    </Apply>
    <Apply function="equal">
      <FieldRef field="dany"/>
      <Constant dataType="string">dany1</Constant>
    </Apply>
  </Apply>
  <Apply function="greaterThan">
    <FieldRef field="weeklyalc"/>
    <Constant dataType="double">14</Constant>
  </Apply>
</Apply>
<Constant dataType="double">1</Constant>
<Apply function="if">
  <Apply function="and">
    <Apply function="and">
      <Apply function="and">
        <Apply function="equal">
          <FieldRef field="Sex"/>
          <Constant dataType="double">0</Constant>
        </Apply>
        <Apply function="equal">
          <FieldRef field="dev"/>
          <Constant dataType="string">dev1</Constant>
        </Apply>
      </Apply>
      <Apply function="equal">
        <FieldRef field="dany"/>
        <Constant dataType="string">dany1</Constant>
      </Apply>
    </Apply>
    <Apply function="lessOrEqual">
      <FieldRef field="weeklyalc"/>
      <Constant dataType="double">21</Constant>
    </Apply>
  </Apply>
  <Constant dataType="double">0</Constant>
  <Apply function="if">
    <Apply function="and">
      <Apply function="and">
        <Apply function="and">
          <Apply function="equal">
            <FieldRef field="Sex"/>
            <Constant dataType="double">1</Constant>
          </Apply>
          <Apply function="equal">
            <FieldRef field="dev"/>
            <Constant dataType="string">dev1</Constant>
          </Apply>
        </Apply>
        <Apply function="equal">
          <FieldRef field="dany"/>
          <Constant dataType="string">dany1</Constant>
```

```
            </Apply>
          </Apply>
          <Apply function="lessOrEqual">
            <FieldRef field="weeklyalc"/>
            <Constant dataType="double">14</Constant>
          </Apply>
        </Apply>
        <Constant dataType="double">0</Constant>
        <Apply function="if">
          <Apply function="or">
            <Apply function="equal">
              <FieldRef field="dev"/>
              <Constant dataType="string">dev2</Constant>
            </Apply>
            <Apply function="equal">
              <FieldRef field="dany"/>
              <Constant dataType="string">dany2</Constant>
            </Apply>
          </Apply>
          <Constant dataType="double">0</Constant>
          <FieldRef field="NA"/>
        </Apply>
      </Apply>
    </Apply>
  </Apply>
</Apply>
 </DerivedField>
 <DerivedField name="djuice" dataType="double" optype="continuous">
<Apply function="if">
  <Apply function="equal">
    <FieldRef field="juiu"/>
    <Constant dataType="string">juid</Constant>
  </Apply>
  <FieldRef field="jui"/>
  <Apply function="if">
    <Apply function="equal">
      <FieldRef field="juiu"/>
      <Constant dataType="string">juiw</Constant>
    </Apply>
    <Apply function="/">
      <FieldRef field="jui"/>
      <Constant dataType="double">7</Constant>
    </Apply>
    <Apply function="if">
      <Apply function="equal">
        <FieldRef field="juiu"/>
        <Constant dataType="string">juim</Constant>
      </Apply>
      <Apply function="/">
        <FieldRef field="jui"/>
        <Constant dataType="double">30</Constant>
      </Apply>
      <Apply function="if">
        <Apply function="equal">
```

```
          <FieldRef field="juiu"/>
          <Constant dataType="string">juiy</Constant>
        </Apply>
        <Apply function="/">
          <FieldRef field="jui"/>
          <Constant dataType="double">365</Constant>
        </Apply>
        <Constant dataType="double">0</Constant>
      </Apply>
    </Apply>
  </Apply>
</Apply>
 </DerivedField>
 <DerivedField name="dfruit" dataType="double" optype="continuous">
<Apply function="if">
  <Apply function="equal">
    <FieldRef field="frtu"/>
    <Constant dataType="string">frtd</Constant>
  </Apply>
  <FieldRef field="frt"/>
  <Apply function="if">
    <Apply function="equal">
      <FieldRef field="frtu"/>
      <Constant dataType="string">frtw</Constant>
    </Apply>
    <Apply function="/">
      <FieldRef field="frt"/>
      <Constant dataType="double">7</Constant>
    </Apply>
    <Apply function="if">
      <Apply function="equal">
        <FieldRef field="frtu"/>
        <Constant dataType="string">frtm</Constant>
      </Apply>
      <Apply function="/">
        <FieldRef field="frt"/>
        <Constant dataType="double">30</Constant>
      </Apply>
      <Apply function="if">
        <Apply function="equal">
          <FieldRef field="frtu"/>
          <Constant dataType="string">frty</Constant>
        </Apply>
        <Apply function="/">
          <FieldRef field="frt"/>
          <Constant dataType="double">365</Constant>
        </Apply>
        <Constant dataType="double">0</Constant>
      </Apply>
    </Apply>
  </Apply>
</Apply>
 </DerivedField>
 <DerivedField name="dsalad" dataType="double" optype="continuous">
```

```
<Apply function="if">
  <Apply function="equal">
    <FieldRef field="salu"/>
    <Constant dataType="string">sald</Constant>
  </Apply>
  <FieldRef field="sal"/>
  <Apply function="if">
    <Apply function="equal">
      <FieldRef field="salu"/>
      <Constant dataType="string">salw</Constant>
    </Apply>
    <Apply function="/">
      <FieldRef field="sal"/>
      <Constant dataType="double">7</Constant>
    </Apply>
    <Apply function="if">
      <Apply function="equal">
        <FieldRef field="salu"/>
        <Constant dataType="string">salm</Constant>
      </Apply>
      <Apply function="/">
        <FieldRef field="sal"/>
        <Constant dataType="double">30</Constant>
      </Apply>
      <Apply function="if">
        <Apply function="equal">
          <FieldRef field="salu"/>
          <Constant dataType="string">saly</Constant>
        </Apply>
        <Apply function="/">
          <FieldRef field="sal"/>
          <Constant dataType="double">365</Constant>
        </Apply>
        <Constant dataType="double">0</Constant>
      </Apply>
    </Apply>
  </Apply>
</Apply>
 </DerivedField>
 <DerivedField name="dpotato" dataType="double" optype="continuous">
<Apply function="if">
  <Apply function="equal">
    <FieldRef field="potu"/>
    <Constant dataType="string">potd</Constant>
  </Apply>
  <FieldRef field="pot"/>
  <Apply function="if">
    <Apply function="equal">
      <FieldRef field="potu"/>
      <Constant dataType="string">potw</Constant>
    </Apply>
    <Apply function="/">
      <FieldRef field="pot"/>
      <Constant dataType="double">7</Constant>
```

```
    </Apply>
    <Apply function="if">
      <Apply function="equal">
        <FieldRef field="potu"/>
        <Constant dataType="string">potm</Constant>
      </Apply>
      <Apply function="/">
        <FieldRef field="pot"/>
        <Constant dataType="double">30</Constant>
      </Apply>
      <Apply function="if">
        <Apply function="equal">
          <FieldRef field="potu"/>
          <Constant dataType="string">poty</Constant>
        </Apply>
        <Apply function="/">
          <FieldRef field="pot"/>
          <Constant dataType="double">365</Constant>
        </Apply>
        <Constant dataType="double">0</Constant>
      </Apply>
    </Apply>
  </Apply>
</Apply>
 </DerivedField>
 <DerivedField name="dcarrot" dataType="double" optype="continuous">
<Apply function="if">
  <Apply function="equal">
    <FieldRef field="caru"/>
    <Constant dataType="string">card</Constant>
  </Apply>
  <FieldRef field="car"/>
  <Apply function="if">
    <Apply function="equal">
      <FieldRef field="caru"/>
      <Constant dataType="string">carw</Constant>
    </Apply>
    <Apply function="/">
      <FieldRef field="car"/>
      <Constant dataType="double">7</Constant>
    </Apply>
    <Apply function="if">
      <Apply function="equal">
        <FieldRef field="caru"/>
        <Constant dataType="string">carm</Constant>
      </Apply>
      <Apply function="/">
        <FieldRef field="car"/>
        <Constant dataType="double">30</Constant>
      </Apply>
      <Apply function="if">
        <Apply function="equal">
          <FieldRef field="caru"/>
          <Constant dataType="string">cary</Constant>
```

```
        </Apply>
        <Apply function="/">
          <FieldRef field="car"/>
          <Constant dataType="double">365</Constant>
        </Apply>
        <Constant dataType="double">0</Constant>
      </Apply>
    </Apply>
  </Apply>
</Apply>
 </DerivedField>
 <DerivedField name="dveg" dataType="double" optype="continuous">
<Apply function="if">
  <Apply function="equal">
    <FieldRef field="vegu"/>
    <Constant dataType="string">vegd</Constant>
  </Apply>
  <FieldRef field="veg"/>
  <Apply function="if">
    <Apply function="equal">
      <FieldRef field="vegu"/>
      <Constant dataType="string">vegw</Constant>
    </Apply>
    <Apply function="/">
      <FieldRef field="veg"/>
      <Constant dataType="double">7</Constant>
    </Apply>
    <Apply function="if">
      <Apply function="equal">
        <FieldRef field="vegu"/>
        <Constant dataType="string">vegm</Constant>
      </Apply>
      <Apply function="/">
        <FieldRef field="veg"/>
        <Constant dataType="double">30</Constant>
      </Apply>
      <Apply function="if">
        <Apply function="equal">
          <FieldRef field="vegu"/>
          <Constant dataType="string">vegy</Constant>
        </Apply>
        <Apply function="/">
          <FieldRef field="veg"/>
          <Constant dataType="double">365</Constant>
        </Apply>
        <Constant dataType="double">0</Constant>
      </Apply>
    </Apply>
  </Apply>
</Apply>
 </DerivedField>
 <DerivedField name="fruitnvegraw" dataType="double" optype="continuous">
<Apply function="+">
  <Apply function="+">
```

```
    <Apply function="+">
      <Apply function="+">
        <FieldRef field="dfruit"/>
        <FieldRef field="dsalad"/>
      </Apply>
      <FieldRef field="dpotato"/>
    </Apply>
    <FieldRef field="dcarrot"/>
  </Apply>
  <FieldRef field="dveg"/>
</Apply>
 </DerivedField>
 <DerivedField name="fruitnveg" dataType="double" optype="continuous">
<Apply function="if">
  <Apply function="and">
    <Apply function="greaterThan">
      <FieldRef field="fruitnvegraw"/>
      <Constant dataType="double">8</Constant>
    </Apply>
    <Apply function="lessThan">
      <FieldRef field="fruitnvegraw"/>
      <Constant dataType="double">98</Constant>
    </Apply>
  </Apply>
  <Constant dataType="double">8</Constant>
  <FieldRef field="fruitnvegraw"/>
</Apply>
 </DerivedField>
 <DerivedField name="nocarrotflag" dataType="double" optype="continuous">
<Apply function="if">
  <Apply function="equal">
    <Apply function="*">
      <FieldRef field="dcarrot"/>
      <Constant dataType="double">7</Constant>
    </Apply>
    <Constant dataType="double">0</Constant>
  </Apply>
  <Constant dataType="double">1</Constant>
  <Apply function="if">
    <Apply function="greaterOrEqual">
      <Apply function="*">
        <FieldRef field="dcarrot"/>
        <Constant dataType="double">7</Constant>
      </Apply>
      <Constant dataType="double">1</Constant>
    </Apply>
    <Constant dataType="double">0</Constant>
    <Constant dataType="double">0</Constant>
  </Apply>
</Apply>
 </DerivedField>
 <DerivedField name="highpotatoflag" dataType="double"
optype="continuous">
<Apply function="if">
```

```
    <Apply function="and">
      <Apply function="equal">
        <FieldRef field="Sex"/>
        <Constant dataType="double">0</Constant>
      </Apply>
      <Apply function="greaterOrEqual">
        <Apply function="*">
          <FieldRef field="dpotato"/>
          <Constant dataType="double">7</Constant>
        </Apply>
        <Constant dataType="double">7</Constant>
      </Apply>
    </Apply>
    <Constant dataType="double">1</Constant>
    <Apply function="if">
      <Apply function="and">
        <Apply function="equal">
          <FieldRef field="Sex"/>
          <Constant dataType="double">0</Constant>
        </Apply>
        <Apply function="greaterOrEqual">
          <Apply function="*">
            <FieldRef field="dpotato"/>
            <Constant dataType="double">7</Constant>
          </Apply>
          <Constant dataType="double">5</Constant>
        </Apply>
      </Apply>
      <Constant dataType="double">1</Constant>
      <Constant dataType="double">0</Constant>
    </Apply>
  </Apply>
   </DerivedField>
   <DerivedField name="highjuice" dataType="double" optype="continuous">
  <Apply function="if">
    <Apply function="equal">
      <FieldRef field="djuice"/>
      <Constant dataType="double">2</Constant>
    </Apply>
    <Constant dataType="double">1</Constant>
    <Apply function="if">
      <Apply function="equal">
        <FieldRef field="djuice"/>
        <Constant dataType="double">3</Constant>
      </Apply>
      <Constant dataType="double">2</Constant>
      <Apply function="if">
        <Apply function="equal">
          <FieldRef field="djuice"/>
          <Constant dataType="double">4</Constant>
        </Apply>
        <Constant dataType="double">3</Constant>
        <Apply function="if">
          <Apply function="equal">
```

```
          <FieldRef field="djuice"/>
          <Constant dataType="double">5</Constant>
        </Apply>
        <Constant dataType="double">4</Constant>
        <Apply function="if">
          <Apply function="equal">
            <FieldRef field="djuice"/>
            <Constant dataType="double">5</Constant>
          </Apply>
          <Constant dataType="double">4</Constant>
          <Apply function="if">
            <Apply function="equal">
              <FieldRef field="djuice"/>
              <Constant dataType="double">6</Constant>
            </Apply>
            <Constant dataType="double">5</Constant>
            <Apply function="if">
              <Apply function="equal">
                <FieldRef field="djuice"/>
                <Constant dataType="double">1</Constant>
              </Apply>
              <Constant dataType="double">0</Constant>
              <Apply function="if">
                <Apply function="equal">
                  <FieldRef field="djuice"/>
                  <Constant dataType="double">0</Constant>
                </Apply>
                <Constant dataType="double">0</Constant>
                <Constant dataType="double">0</Constant>
              </Apply>
            </Apply>
          </Apply>
        </Apply>
      </Apply>
    </Apply>
  </Apply>
</Apply>
 </DerivedField>
 <DerivedField name="dietraw" dataType="double" optype="continuous">
<Apply function="-">
  <Apply function="-">
    <Apply function="-">
      <FieldRef field="fruitnveg"/>
      <Apply function="*">
        <Constant dataType="double">2</Constant>
        <FieldRef field="highpotatoflag"/>
      </Apply>
    </Apply>
    <Apply function="*">
      <Constant dataType="double">2</Constant>
      <FieldRef field="nocarrotflag"/>
    </Apply>
  </Apply>
  <Apply function="*">
```

```
    <Constant dataType="double">2</Constant>
    <FieldRef field="highjuice"/>
  </Apply>
</Apply>
 </DerivedField>
 <DerivedField name="DietScore_cont" dataType="double"
optype="continuous">
<Apply function="if">
  <Apply function="lessThan">
    <FieldRef field="dietraw"/>
    <Constant dataType="double">0</Constant>
  </Apply>
  <Constant dataType="double">0</Constant>
  <Apply function="if">
    <Apply function="greaterThan">
      <FieldRef field="dietraw"/>
      <Constant dataType="double">10</Constant>
    </Apply>
    <Constant dataType="double">10</Constant>
    <FieldRef field="dietraw"/>
  </Apply>
</Apply>
 </DerivedField>
 <DerivedField name="HeartDis_cat" dataType="double" optype="continuous">
<Apply function="if">
  <Apply function="equal">
    <FieldRef field="hd"/>
    <Constant dataType="string">hd1</Constant>
  </Apply>
  <Constant dataType="double">1</Constant>
  <Apply function="if">
    <Apply function="equal">
      <FieldRef field="hd"/>
      <Constant dataType="string">hd2</Constant>
    </Apply>
    <Constant dataType="double">0</Constant>
    <FieldRef field="NA"/>
  </Apply>
</Apply>
 </DerivedField>
 <DerivedField name="Diabetes_cat" dataType="double" optype="continuous">
<Apply function="if">
  <Apply function="equal">
    <FieldRef field="diab"/>
    <Constant dataType="string">diab1</Constant>
  </Apply>
  <Constant dataType="double">1</Constant>
  <Apply function="if">
    <Apply function="equal">
      <FieldRef field="diab"/>
      <Constant dataType="string">diab2</Constant>
    </Apply>
    <Constant dataType="double">0</Constant>
    <FieldRef field="NA"/>
```

```
  &lt;/Apply&gt;
&lt;/Apply&gt;
 &lt;/DerivedField&gt;
 &lt;DerivedField name="Stroke_cat" dataType="double" optype="continuous"&gt;
&lt;Apply function="if"&gt;
  &lt;Apply function="equal"&gt;
    &lt;FieldRef field="stk"/&gt;
    &lt;Constant dataType="string"&gt;stk1&lt;/Constant&gt;
  &lt;/Apply&gt;
  &lt;Constant dataType="double"&gt;1&lt;/Constant&gt;
  &lt;Apply function="if"&gt;
    &lt;Apply function="equal"&gt;
      &lt;FieldRef field="stk"/&gt;
      &lt;Constant dataType="string"&gt;stk2&lt;/Constant&gt;
    &lt;/Apply&gt;
    &lt;Constant dataType="double"&gt;0&lt;/Constant&gt;
    &lt;FieldRef field="NA"/&gt;
  &lt;/Apply&gt;
&lt;/Apply&gt;
 &lt;/DerivedField&gt;
 &lt;DerivedField name="Cancer_cat" dataType="double" optype="continuous"&gt;
&lt;Apply function="if"&gt;
  &lt;Apply function="equal"&gt;
    &lt;FieldRef field="can"/&gt;
    &lt;Constant dataType="string"&gt;can1&lt;/Constant&gt;
  &lt;/Apply&gt;
  &lt;Constant dataType="double"&gt;1&lt;/Constant&gt;
  &lt;Apply function="if"&gt;
    &lt;Apply function="equal"&gt;
      &lt;FieldRef field="can"/&gt;
      &lt;Constant dataType="string"&gt;can2&lt;/Constant&gt;
    &lt;/Apply&gt;
    &lt;Constant dataType="double"&gt;0&lt;/Constant&gt;
    &lt;FieldRef field="NA"/&gt;
  &lt;/Apply&gt;
&lt;/Apply&gt;
 &lt;/DerivedField&gt;
 <DerivedField name="DiabetesAge_int" dataType="double"
optype="continuous">
&lt;Apply function="*"&gt;
  &lt;FieldRef field="Diabetes_cat"/&gt;
  &lt;FieldRef field="Age_cont"/&gt;
&lt;/Apply&gt;
 &lt;/DerivedField&gt;
 <DerivedField name="CancerAge_int" dataType="double"
optype="continuous">
&lt;Apply function="*"&gt;
  &lt;FieldRef field="Cancer_cat"/&gt;
  &lt;FieldRef field="Age_cont"/&gt;
&lt;/Apply&gt;
 &lt;/DerivedField&gt;
 &lt;DerivedField name="weightkg" dataType="double" optype="continuous"&gt;
&lt;Apply function="/"&gt;
  &lt;FieldRef field="weightlb"/&gt;
```

```
  <Constant dataType="double">2.2046226218</Constant>
</Apply>
 </DerivedField>
 <DerivedField name="heightm" dataType="double" optype="continuous">
<Apply function="/">
  <Apply function="+">
    <Apply function="*">
      <FieldRef field="heightin_hft"/>
      <Constant dataType="double">12</Constant>
    </Apply>
    <FieldRef field="heightin_hin"/>
  </Apply>
  <Constant dataType="double">39.3701</Constant>
</Apply>
 </DerivedField>
 <DerivedField name="BMI_spline" dataType="double" optype="continuous">
<Apply function="if">
  <Apply function="lessOrEqual">
    <Apply function="-">
      <Apply function="/">
        <FieldRef field="weightkg"/>
        <Apply function="*">
          <FieldRef field="heightm"/>
          <FieldRef field="heightm"/>
        </Apply>
      </Apply>
      <Constant dataType="double">35</Constant>
    </Apply>
    <Constant dataType="double">0</Constant>
  </Apply>
  <Constant dataType="double">0</Constant>
  <Apply function="if">
    <Apply function="greaterThan">
      <Apply function="-">
        <Apply function="/">
          <FieldRef field="weightkg"/>
          <Apply function="*">
            <FieldRef field="heightm"/>
            <FieldRef field="heightm"/>
          </Apply>
        </Apply>
        <Constant dataType="double">35</Constant>
      </Apply>
      <Constant dataType="double">0</Constant>
    </Apply>
    <Apply function="-">
      <Apply function="/">
        <FieldRef field="weightkg"/>
        <Apply function="*">
          <FieldRef field="heightm"/>
          <FieldRef field="heightm"/>
        </Apply>
      </Apply>
      <Constant dataType="double">35</Constant>
```

```
    </Apply>
   </Apply>
  </Apply>
 </DerivedField>
</LocalTransformations>
 <GeneralRegressionModel modelType="CoxRegression"
modelName="MPoRT_FemaleModel" functionName="regression"
algorithmName="coxph" startTimeVariable="start" endTimeVariable="stop"
statusVariable="EventDeath">
  <MiningSchema>
   <MiningField name="survival" usageType="predicted"/>
   <MiningField name="Age_cont" usageType="active"/>
   <MiningField name="Age_spline" usageType="active"/>
   <MiningField name="QSLight_df" usageType="active"/>
   <MiningField name="QSHeavy_df" usageType="active"/>
   <MiningField name="PhysicalActivity_cont" usageType="active"/>
   <MiningField name="DietScore_cont" usageType="active"/>
   <MiningField name="AlcoholHeavy_cat" usageType="active"/>
   <MiningField name="AlcoholMod_cat" usageType="active"/>
   <MiningField name="DepIndMod_cat" usageType="active"/>
   <MiningField name="DepIndHigh_cat" usageType="active"/>
   <MiningField name="EduNoGrad_cat" usageType="active"/>
   <MiningField name="EduHSGrad_cat" usageType="active"/>
   <MiningField name="ImEth0To15_cat" usageType="active"/>
   <MiningField name="ImEth16To30_cat" usageType="active"/>
   <MiningField name="ImEth31To45_cat" usageType="active"/>
   <MiningField name="HeartDis_cat" usageType="active"/>
   <MiningField name="Stroke_cat" usageType="active"/>
   <MiningField name="Cancer_cat" usageType="active"/>
   <MiningField name="Diabetes_cat" usageType="active"/>
   <MiningField name="BMI_spline" usageType="active"/>
   <MiningField name="CancerAge_Int" usageType="active"/>
   <MiningField name="DiabetesAge_Int" usageType="active"/>
   <MiningField name="start" usageType="active"/>
   <MiningField name="stop" usageType="active"/>
   <MiningField name="EventDeath" usageType="active"/>
  </MiningSchema>
  <Output>
   <OutputField name="Predicted_survival" feature="predictedValue"/>
   <OutputField name="cumulativeHazard" feature="transformedValue">
    <Apply function="*">
     <Constant>-1.0</Constant>
     <Apply function="ln">
      <FieldRef field="Predicted_survival"/>
     </Apply>
    </Apply>
   </OutputField>
  </Output>
  <ParameterList>
   <Parameter name="p0" label="Age_cont"
referencePoint="54.1488503875064"/>
   <Parameter name="p1" label="Age_spline"
referencePoint="0.425166096111415"/>
```

<Parameter name="p2" label="QSLight_df"
referencePoint="0.254288098778076"/>
<Parameter name="p3" label="QSHeavy_df"
referencePoint="0.106537866589885"/>
<Parameter name="p4" label="PhysicalActivity_cont"
referencePoint="0.384627883292789"/>
<Parameter name="p5" label="DietScore_cont"
referencePoint="5.49853294572499"/>
<Parameter name="p6" label="AlcoholHeavy_cat1"
referencePoint="0.0684543381661514"/>
<Parameter name="p7" label="AlcoholMod_cat1"
referencePoint="0.209846533953671"/>
<Parameter name="p8" label="DepIndMod_cat1"
referencePoint="0.635538114524882"/>
<Parameter name="p9" label="DepIndHigh_cat1"
referencePoint="0.168108932006613"/>
<Parameter name="p10" label="EduNoGrad_cat1"
referencePoint="0.186175149194564"/>
<Parameter name="p11" label="EduHSGrad_cat1"
referencePoint="0.265105373462728"/>
<Parameter name="p12" label="ImEth0To15_cat1"
referencePoint="0.0375613833455229"/>
<Parameter name="p13" label="ImEth16To30_cat1"
referencePoint="0.0437872986411156"/>
<Parameter name="p14" label="ImEth31To45_cat1"
referencePoint="0.0547179900065762"/>
<Parameter name="p15" label="HeartDis_cat1"
referencePoint="0.0668102785989539"/>
<Parameter name="p16" label="Stroke_cat1"
referencePoint="0.0132262287424634"/>
<Parameter name="p17" label="Cancer_cat1"
referencePoint="0.0222424357894866"/>
<Parameter name="p18" label="Diabetes_cat1"
referencePoint="0.0636297147633476"/>
<Parameter name="p19" label="BMI_spline"
referencePoint="0.286680013767083"/>
<Parameter name="p20" label="CancerAge_Int"
referencePoint="1.45539528108809"/>
<Parameter name="p21" label="DiabetesAge_Int"
referencePoint="4.19181719286816"/>
</ParameterList>
<FactorList>
<Predictor name="AlcoholHeavy_cat"/>
<Predictor name="AlcoholMod_cat"/>
<Predictor name="DepIndMod_cat"/>
<Predictor name="DepIndHigh_cat"/>
<Predictor name="EduNoGrad_cat"/>
<Predictor name="EduHSGrad_cat"/>
<Predictor name="ImEth0To15_cat"/>
<Predictor name="ImEth16To30_cat"/>
<Predictor name="ImEth31To45_cat"/>
<Predictor name="HeartDis_cat"/>
<Predictor name="Stroke_cat"/>
<Predictor name="Cancer_cat"/>

```
   <Predictor name="Diabetes_cat"/>
  </FactorList>
  <CovariateList>
   <Predictor name="Age_cont"/>
   <Predictor name="Age_spline"/>
   <Predictor name="QSLight_df"/>
   <Predictor name="QSHeavy_df"/>
   <Predictor name="PhysicalActivity_cont"/>
   <Predictor name="DietScore_cont"/>
   <Predictor name="BMI_spline"/>
   <Predictor name="CancerAge_Int"/>
   <Predictor name="DiabetesAge_Int"/>
  </CovariateList>
  <PPMatrix>
   <PPCell value="1" predictorName="Age_cont" parameterName="p0"/>
   <PPCell value="1" predictorName="Age_spline" parameterName="p1"/>
   <PPCell value="1" predictorName="QSLight_df" parameterName="p2"/>
   <PPCell value="1" predictorName="QSHeavy_df" parameterName="p3"/>
   <PPCell value="1" predictorName="PhysicalActivity_cont"
parameterName="p4"/>
   <PPCell value="1" predictorName="DietScore_cont" parameterName="p5"/>
   <PPCell value="1" predictorName="AlcoholHeavy_cat"
parameterName="p6"/>
   <PPCell value="1" predictorName="AlcoholMod_cat" parameterName="p7"/>
   <PPCell value="1" predictorName="DepIndMod_cat" parameterName="p8"/>
   <PPCell value="1" predictorName="DepIndHigh_cat" parameterName="p9"/>
   <PPCell value="1" predictorName="EduNoGrad_cat" parameterName="p10"/>
   <PPCell value="1" predictorName="EduHSGrad_cat" parameterName="p11"/>
   <PPCell value="1" predictorName="ImEth0To15_cat" parameterName="p12"/>
   <PPCell value="1" predictorName="ImEth16To30_cat"
parameterName="p13"/>
   <PPCell value="1" predictorName="ImEth31To45_cat"
parameterName="p14"/>
   <PPCell value="1" predictorName="HeartDis_cat" parameterName="p15"/>
   <PPCell value="1" predictorName="Stroke_cat" parameterName="p16"/>
   <PPCell value="1" predictorName="Cancer_cat" parameterName="p17"/>
   <PPCell value="1" predictorName="Diabetes_cat" parameterName="p18"/>
   <PPCell value="1" predictorName="BMI_spline" parameterName="p19"/>
   <PPCell value="1" predictorName="CancerAge_Int" parameterName="p20"/>
   <PPCell value="1" predictorName="DiabetesAge_Int"
parameterName="p21"/>
  </PPMatrix>
  <ParamMatrix>
   <PCell parameterName="p0" df="1" beta="0.0891888015031822"/>
   <PCell parameterName="p1" df="1" beta="0.0292179374194524"/>
   <PCell parameterName="p2" df="1" beta="0.806847255361553"/>
   <PCell parameterName="p3" df="1" beta="1.18030397927685"/>
   <PCell parameterName="p4" df="1" beta="-0.928355359618802"/>
   <PCell parameterName="p5" df="1" beta="-0.0436711994153916"/>
   <PCell parameterName="p6" df="1" beta="0.0797049709902844"/>
   <PCell parameterName="p7" df="1" beta="-0.200633770475874"/>
   <PCell parameterName="p8" df="1" beta="0.0465870069699607"/>
   <PCell parameterName="p9" df="1" beta="0.204884993101581"/>
   <PCell parameterName="p10" df="1" beta="0.063130759838211"/>
```

```
   <PCell parameterName="p11" df="1" beta="0.0327085901623164"/>
   <PCell parameterName="p12" df="1" beta="-0.604221147677245"/>
   <PCell parameterName="p13" df="1" beta="-0.226038245860603"/>
   <PCell parameterName="p14" df="1" beta="-0.132506130017869"/>
   <PCell parameterName="p15" df="1" beta="0.341870654935853"/>
   <PCell parameterName="p16" df="1" beta="0.235906455174582"/>
   <PCell parameterName="p17" df="1" beta="4.85671898618751"/>
   <PCell parameterName="p18" df="1" beta="1.16018072455526"/>
   <PCell parameterName="p19" df="1" beta="0.0240673793676962"/>
   <PCell parameterName="p20" df="1" beta="-0.0525599675753628"/>
   <PCell parameterName="p21" df="1" beta="-0.00942704419043476"/>
  </ParamMatrix>
  <BaseCumHazardTables maxTime="11">
   <BaselineCell time="1" cumHazard="0.00162888831994313"/>
   <BaselineCell time="2" cumHazard="0.00387081862898266"/>
   <BaselineCell time="3" cumHazard="0.00645247012367042"/>
   <BaselineCell time="4" cumHazard="0.00929257917964735"/>
   <BaselineCell time="5" cumHazard="0.0118055068777082"/>
   <BaselineCell time="6" cumHazard="0.0142938820233747"/>
   <BaselineCell time="7" cumHazard="0.0173234744780458"/>
   <BaselineCell time="8" cumHazard="0.0200271665284219"/>
   <BaselineCell time="9" cumHazard="0.0221487624873231"/>
   <BaselineCell time="10" cumHazard="0.0245495842069407"/>
   <BaselineCell time="11" cumHazard="0.0246691941896427"/>
  </BaseCumHazardTables>
 </GeneralRegressionModel>
</PMML>
```
