# Supplementary material for: Measuring Burden of Unhealthy Behaviours Using a Multivariable Predictive Approach: Life Expectancy Lost in Canada Attributable to Smoking, Alcohol, Physical Inactivity, and Diet
Source: PLoS Med. 2016 Aug 16;13(8):e1002082. doi: 10.1371/journal.pmed.1002082 (PMC4986987; doi:10.1371/journal.pmed.1002082)
Supplement: S3 Text — (PDF) [file pmed.1002082.s017.pdf]

S3 Text. Lime questionnaire file


```
<LocalTransformations>
 <DerivedField name="Sex" dataType="double" optype="continuous">
<Apply function="if">
  <Apply function="equal">
    <FieldRef field="sex"/>
    <Constant dataType="string">fem</Constant>
  </Apply>
  <Constant dataType="double">1</Constant>
  <Apply function="if">
    <Apply function="equal">
      <FieldRef field="sex"/>
      <Constant dataType="string">male</Constant>
    </Apply>
    <Constant dataType="double">0</Constant>
  </Apply>
</Apply>
 </DerivedField>
 <DerivedField name="Age_spline" dataType="double" optype="continuous">
<Apply function="if">
  <Apply function="and">
    <Apply function="equal">
      <FieldRef field="Sex"/>
      <Constant dataType="string">fem</Constant>
    </Apply>
    <Apply function="greaterThan">
      <FieldRef field="Age"/>
      <Constant dataType="double">80</Constant>
    </Apply>
  </Apply>
  <Apply function="-">
    <FieldRef field="Age"/>
    <Constant dataType="double">80</Constant>
  </Apply>
  <Apply function="if">
    <Apply function="equal">
      <FieldRef field="Sex"/>
      <Constant dataType="string">fem</Constant>
    </Apply>
    <Constant dataType="double">0</Constant>
    <Apply function="if">
      <Apply function="and">
        <Apply function="equal">
          <FieldRef field="Sex"/>
          <Constant dataType="string">male</Constant>
        </Apply>
        <Apply function="greaterThan">
          <FieldRef field="Age"/>
          <Constant dataType="double">65</Constant>
        </Apply>
      </Apply>
      <Apply function="-">
        <FieldRef field="Age"/>
        <Constant dataType="double">65</Constant>
      </Apply>
      <Constant dataType="double">0</Constant>
    </Apply>
  </Apply>
</Apply>
 </DerivedField>
 <DerivedField name="EduNoGrad_cat" dataType="double" optype="continuous">
```

```
<Apply function="if">
  <Apply function="and">
    <Apply function="equal">
      <FieldRef field="hs"/>
      <Constant dataType="string">hs2</Constant>
    </Apply>
    <Apply function="equal">
      <FieldRef field="ed"/>
      <Constant dataType="string">ed2</Constant>
    </Apply>
  </Apply>
  <Constant dataType="double">1</Constant>
  <Constant dataType="double">0</Constant>
</Apply>
 </DerivedField>
 <DerivedField name="EduHSGrad_cat" dataType="double" optype="continuous">
<Apply function="if">
  <Apply function="or">
    <Apply function="and">
      <Apply function="equal">
        <FieldRef field="hs"/>
        <Constant dataType="string">hs1</Constant>
      </Apply>
      <Apply function="equal">
        <FieldRef field="ed"/>
        <Constant dataType="string">ed2</Constant>
      </Apply>
    </Apply>
    <Apply function="and">
      <Apply function="and">
        <Apply function="equal">
          <FieldRef field="hs"/>
          <Constant dataType="string">hs1</Constant>
        </Apply>
        <Apply function="equal">
          <FieldRef field="ed"/>
          <Constant dataType="string">ed1</Constant>
        </Apply>
      </Apply>
      <Apply function="equal">
        <FieldRef field="hdg"/>
        <Constant dataType="string">hdg1</Constant>
      </Apply>
    </Apply>
  </Apply>
  <Constant dataType="double">1</Constant>
  <Constant dataType="double">0</Constant>
</Apply>
 </DerivedField>
 <DerivedField name="DepIndHigh_cat" dataType="double" optype="continuous">
<Apply function="if">
  <Apply function="equal">
    <FieldRef field="dep"/>
    <Constant dataType="string">dep1</Constant>
  </Apply>
  <Constant dataType="double">1</Constant>
  <Constant dataType="double">0</Constant>
</Apply>
 </DerivedField>
 <DerivedField name="DepIndMod_cat" dataType="double" optype="continuous">
<Apply function="if">
```

```
  <Apply function="equal">
    <FieldRef field="dep"/>
    <Constant dataType="string">dep2</Constant>
  </Apply>
  <Constant dataType="double">1</Constant>
  <Constant dataType="double">0</Constant>
</Apply>
 </DerivedField>
 <DerivedField name="ImEth0To15_cat" dataType="double" optype="continuous">
<Apply function="if">
  <Apply function="and">
    <Apply function="and">
      <Apply function="equal">
        <FieldRef field="imm"/>
        <Constant dataType="string">imm2</Constant>
      </Apply>
      <Apply function="greaterOrEqual">
        <FieldRef field="imyr"/>
        <Constant dataType="double">1</Constant>
      </Apply>
    </Apply>
    <Apply function="lessOrEqual">
      <FieldRef field="imyr"/>
      <Constant dataType="double">15</Constant>
    </Apply>
  </Apply>
  <Constant dataType="double">1</Constant>
  <Apply function="if">
    <Apply function="or">
      <Apply function="equal">
        <FieldRef field="imm"/>
        <Constant dataType="string">imm1</Constant>
      </Apply>
      <Apply function="greaterThan">
        <FieldRef field="imyr"/>
        <Constant dataType="double">15</Constant>
      </Apply>
    </Apply>
    <Constant dataType="double">0</Constant>
    <Constant dataType="double">0</Constant>
  </Apply>
</Apply>
 </DerivedField>
 <DerivedField name="ImEth16To30_cat" dataType="double" optype="continuous">
<Apply function="if">
  <Apply function="and">
    <Apply function="and">
      <Apply function="equal">
        <FieldRef field="imm"/>
        <Constant dataType="string">imm2</Constant>
      </Apply>
      <Apply function="greaterThan">
        <FieldRef field="imyr"/>
        <Constant dataType="double">15</Constant>
      </Apply>
    </Apply>
    <Apply function="lessOrEqual">
      <FieldRef field="imyr"/>
      <Constant dataType="double">30</Constant>
    </Apply>
  </Apply>
```

```
    <Constant dataType="double">1</Constant>
    <Apply function="if">
      <Apply function="or">
        <Apply function="or">
          <Apply function="equal">
            <FieldRef field="imm"/>
            <Constant dataType="string">imm1</Constant>
          </Apply>
          <Apply function="lessOrEqual">
            <FieldRef field="imyr"/>
            <Constant dataType="double">15</Constant>
          </Apply>
        </Apply>
        <Apply function="greaterThan">
          <FieldRef field="imyr"/>
          <Constant dataType="double">30</Constant>
        </Apply>
      </Apply>
      <Constant dataType="double">0</Constant>
      <Constant dataType="double">0</Constant>
    </Apply>
  </Apply>
   </DerivedField>
   <DerivedField name="ImEth31To45_cat" dataType="double" optype="continuous">
  <Apply function="if">
    <Apply function="and">
      <Apply function="and">
        <Apply function="equal">
          <FieldRef field="imm"/>
          <Constant dataType="string">imm2</Constant>
        </Apply>
        <Apply function="greaterThan">
          <FieldRef field="imyr"/>
          <Constant dataType="double">30</Constant>
        </Apply>
      </Apply>
      <Apply function="lessOrEqual">
        <FieldRef field="imyr"/>
        <Constant dataType="double">45</Constant>
      </Apply>
    </Apply>
    <Constant dataType="double">1</Constant>
    <Apply function="if">
      <Apply function="or">
        <Apply function="or">
          <Apply function="equal">
            <FieldRef field="imm"/>
            <Constant dataType="string">imm1</Constant>
          </Apply>
          <Apply function="lessOrEqual">
            <FieldRef field="imyr"/>
            <Constant dataType="double">30</Constant>
          </Apply>
        </Apply>
        <Apply function="greaterThan">
          <FieldRef field="imyr"/>
          <Constant dataType="double">45</Constant>
        </Apply>
      </Apply>
      <Constant dataType="double">0</Constant>
      <Constant dataType="double">0</Constant>
```

```
   </Apply>
 </Apply>
  </DerivedField>
  <DerivedField name="formerlightflag" dataType="double" optype="continuous">
 <Apply function="if">
   <Apply function="and">
     <Apply function="and">
       <Apply function="equal">
         <FieldRef field="smk"/>
         <Constant dataType="string">smk3</Constant>
       </Apply>
       <Apply function="equal">
         <FieldRef field="evdn"/>
         <Constant dataType="string">evdn1</Constant>
       </Apply>
     </Apply>
     <Apply function="lessThan">
       <FieldRef field="cigdayf"/>
       <Constant dataType="double">20</Constant>
     </Apply>
   </Apply>
   <Constant dataType="double">1</Constant>
   <Apply function="if">
     <Apply function="and">
       <Apply function="and">
         <Apply function="equal">
           <FieldRef field="smk"/>
           <Constant dataType="string">smk3</Constant>
         </Apply>
         <Apply function="equal">
           <FieldRef field="evdn"/>
           <Constant dataType="string">evdn2</Constant>
         </Apply>
       </Apply>
       <Apply function="equal">
         <FieldRef field="s100"/>
         <Constant dataType="string">s1001</Constant>
       </Apply>
     </Apply>
     <Constant dataType="double">1</Constant>
     <Constant dataType="double">0</Constant>
   </Apply>
 </Apply>
  </DerivedField>
  <DerivedField name="formerheavyflag" dataType="double" optype="continuous">
 <Apply function="if">
   <Apply function="and">
     <Apply function="and">
       <Apply function="equal">
         <FieldRef field="smk"/>
         <Constant dataType="string">smk3</Constant>
       </Apply>
       <Apply function="equal">
         <FieldRef field="evdn"/>
         <Constant dataType="string">evdn1</Constant>
       </Apply>
     </Apply>
     <Apply function="greaterOrEqual">
       <FieldRef field="cigdayf"/>
       <Constant dataType="double">20</Constant>
     </Apply>
```

```
    &lt;/Apply&gt;
    &lt;Constant dataType="double"&gt;1&lt;/Constant&gt;
    &lt;Constant dataType="double"&gt;0&lt;/Constant&gt;
  &lt;/Apply&gt;
   &lt;/DerivedField&gt;
   &lt;DerivedField name="quittime" dataType="double" optype="continuous"&gt;
  &lt;Apply function="if"&gt;
    &lt;Apply function="or"&gt;
      &lt;Apply function="equal"&gt;
        &lt;FieldRef field="formerlightflag"/&gt;
        &lt;Constant dataType="double"&gt;0&lt;/Constant&gt;
      &lt;/Apply&gt;
      &lt;Apply function="equal"&gt;
        &lt;FieldRef field="formerheavyflag"/&gt;
        &lt;Constant dataType="double"&gt;0&lt;/Constant&gt;
      &lt;/Apply&gt;
    &lt;/Apply&gt;
    &lt;Constant dataType="double"&gt;0&lt;/Constant&gt;
    &lt;Apply function="if"&gt;
      &lt;Apply function="or"&gt;
        &lt;Apply function="equal"&gt;
          &lt;FieldRef field="smk"/&gt;
          &lt;Constant dataType="string"&gt;smk1&lt;/Constant&gt;
        &lt;/Apply&gt;
        &lt;Apply function="="&gt;
          &lt;Apply function="and"&gt;
            &lt;Apply function="and"&gt;
              &lt;Apply function="equal"&gt;
                &lt;FieldRef field="smk"/&gt;
                &lt;Constant dataType="string"&gt;smk3&lt;/Constant&gt;
              &lt;/Apply&gt;
              &lt;Apply function="equal"&gt;
                &lt;FieldRef field="evdn"/&gt;
                &lt;Constant dataType="string"&gt;evdn2&lt;/Constant&gt;
              &lt;/Apply&gt;
            &lt;/Apply&gt;
            &lt;FieldRef field="s100"/&gt;
          &lt;/Apply&gt;
          &lt;Constant dataType="string"&gt;s1002&lt;/Constant&gt;
        &lt;/Apply&gt;
      &lt;/Apply&gt;
      &lt;Constant dataType="double"&gt;0&lt;/Constant&gt;
      &lt;Apply function="if"&gt;
        &lt;Apply function="equal"&gt;
          &lt;FieldRef field="stpn"/&gt;
          &lt;Constant dataType="string"&gt;stpn1&lt;/Constant&gt;
        &lt;/Apply&gt;
        &lt;Constant dataType="double"&gt;0&lt;/Constant&gt;
        &lt;Apply function="if"&gt;
          &lt;Apply function="equal"&gt;
            &lt;FieldRef field="stpn"/&gt;
            &lt;Constant dataType="string"&gt;stpn2&lt;/Constant&gt;
          &lt;/Apply&gt;
          &lt;Constant dataType="double"&gt;1&lt;/Constant&gt;
          &lt;Apply function="if"&gt;
            &lt;Apply function="equal"&gt;
              &lt;FieldRef field="stpn"/&gt;
              &lt;Constant dataType="string"&gt;stpn3&lt;/Constant&gt;
            &lt;/Apply&gt;
            &lt;Constant dataType="double"&gt;2&lt;/Constant&gt;
            &lt;Apply function="if"&gt;
```

```
          <Apply function="equal">
            <FieldRef field="stpn"/>
            <Constant dataType="string">stpn4</Constant>
          </Apply>
          <FieldRef field="stpny"/>
          <FieldRef field="NA"/>
        </Apply>
      </Apply>
    </Apply>
  </Apply>
 </Apply>
</Apply>
 </DerivedField>
 <DerivedField name="smk_lightraw" dataType="double" optype="continuous">
<Apply function="if">
  <Apply function="and">
    <Apply function="equal">
      <FieldRef field="smk"/>
      <Constant dataType="string">smk1</Constant>
    </Apply>
    <Apply function="lessThan">
      <FieldRef field="cigdayd"/>
      <Constant dataType="double">20</Constant>
    </Apply>
  </Apply>
  <Constant dataType="double">1</Constant>
  <Apply function="if">
    <Apply function="equal">
      <FieldRef field="smk"/>
      <Constant dataType="string">smk2</Constant>
    </Apply>
    <Constant dataType="double">1</Constant>
    <Apply function="if">
      <Apply function="and">
        <Apply function="and">
          <Apply function="equal">
            <FieldRef field="smk"/>
            <Constant dataType="string">smk3</Constant>
          </Apply>
          <Apply function="equal">
            <FieldRef field="evdn"/>
            <Constant dataType="string">evdn1</Constant>
          </Apply>
        </Apply>
        <Apply function="lessThan">
          <FieldRef field="cigdayf"/>
          <Constant dataType="double">20</Constant>
        </Apply>
      </Apply>
      <Constant dataType="double">1</Constant>
      <Apply function="if">
        <Apply function="and">
          <Apply function="and">
            <Apply function="equal">
              <FieldRef field="smk"/>
              <Constant dataType="string">smk3</Constant>
            </Apply>
            <Apply function="equal">
              <FieldRef field="evdn"/>
              <Constant dataType="string">evdn2</Constant>
            </Apply>
```

```
          </Apply>
          <Apply function="equal">
            <FieldRef field="s100"/>
            <Constant dataType="string">s1001</Constant>
          </Apply>
        </Apply>
        <Constant dataType="double">1</Constant>
        <Constant dataType="double">0</Constant>
      </Apply>
    </Apply>
  </Apply>
</Apply>
 </DerivedField>
 <DerivedField name="smk_heavyraw" dataType="double" optype="continuous">
<Apply function="if">
  <Apply function="and">
    <Apply function="equal">
      <FieldRef field="smk"/>
      <Constant dataType="string">smk1</Constant>
    </Apply>
    <Apply function="greaterOrEqual">
      <FieldRef field="cigdayd"/>
      <Constant dataType="double">20</Constant>
    </Apply>
  </Apply>
  <Constant dataType="double">1</Constant>
  <Apply function="if">
    <Apply function="and">
      <Apply function="and">
        <Apply function="equal">
          <FieldRef field="smk"/>
          <Constant dataType="string">smk3</Constant>
        </Apply>
        <Apply function="equal">
          <FieldRef field="evdn"/>
          <Constant dataType="string">evdn1</Constant>
        </Apply>
      </Apply>
      <Apply function="greaterOrEqual">
        <FieldRef field="cigdayf"/>
        <Constant dataType="double">20</Constant>
      </Apply>
    </Apply>
    <Constant dataType="double">1</Constant>
    <Constant dataType="double">0</Constant>
  </Apply>
</Apply>
 </DerivedField>
 <DerivedField name="QSLight_df" dataType="double" optype="continuous">
<Apply function="if">
  <Apply function="and">
    <Apply function="equal">
      <FieldRef field="formerlightflag"/>
      <Constant dataType="double">1</Constant>
    </Apply>
    <Apply function="equal">
      <FieldRef field="sex"/>
      <Constant dataType="string">fem</Constant>
    </Apply>
  </Apply>
  <Apply function="exp">
```

```
    &lt;Apply function="/"&gt;
      &lt;FieldRef field="quittime"/&gt;
      &lt;Constant dataType="double"&gt;26&lt;/Constant&gt;
    &lt;/Apply&gt;
  &lt;/Apply&gt;
  &lt;Apply function="if"&gt;
    &lt;Apply function="and"&gt;
      &lt;Apply function="equal"&gt;
        &lt;FieldRef field="formerlightflag"/&gt;
        &lt;Constant dataType="double"&gt;1&lt;/Constant&gt;
      &lt;/Apply&gt;
      &lt;Apply function="equal"&gt;
        &lt;FieldRef field="sex"/&gt;
        &lt;Constant dataType="string"&gt;male&lt;/Constant&gt;
      &lt;/Apply&gt;
    &lt;/Apply&gt;
    &lt;Apply function="exp"&gt;
      &lt;Apply function="/"&gt;
        &lt;FieldRef field="quittime"/&gt;
        &lt;Constant dataType="double"&gt;15&lt;/Constant&gt;
      &lt;/Apply&gt;
    &lt;/Apply&gt;
    &lt;FieldRef field="smk_lightraw"/&gt;
  &lt;/Apply&gt;
&lt;/Apply&gt;
 &lt;/DerivedField&gt;
 &lt;DerivedField name="QSHeavy_df" dataType="double" optype="continuous"&gt;
&lt;Apply function="if"&gt;
  &lt;Apply function="and"&gt;
    &lt;Apply function="equal"&gt;
      &lt;FieldRef field="formerheavyflag"/&gt;
      &lt;Constant dataType="double"&gt;1&lt;/Constant&gt;
    &lt;/Apply&gt;
    &lt;Apply function="equal"&gt;
      &lt;FieldRef field="sex"/&gt;
      &lt;Constant dataType="string"&gt;fem&lt;/Constant&gt;
    &lt;/Apply&gt;
  &lt;/Apply&gt;
  &lt;Apply function="exp"&gt;
    &lt;Apply function="/"&gt;
      &lt;FieldRef field="quittime"/&gt;
      &lt;Constant dataType="double"&gt;26&lt;/Constant&gt;
    &lt;/Apply&gt;
  &lt;/Apply&gt;
  &lt;Apply function="if"&gt;
    &lt;Apply function="and"&gt;
      &lt;Apply function="equal"&gt;
        &lt;FieldRef field="formerheavyflag"/&gt;
        &lt;Constant dataType="double"&gt;1&lt;/Constant&gt;
      &lt;/Apply&gt;
      &lt;Apply function="equal"&gt;
        &lt;FieldRef field="sex"/&gt;
        &lt;Constant dataType="string"&gt;male&lt;/Constant&gt;
      &lt;/Apply&gt;
    &lt;/Apply&gt;
    &lt;Apply function="exp"&gt;
      &lt;Apply function="/"&gt;
        &lt;FieldRef field="quittime"/&gt;
        &lt;Constant dataType="double"&gt;15&lt;/Constant&gt;
      &lt;/Apply&gt;
    &lt;/Apply&gt;
```

```
    <FieldRef field="smk_heavyraw"/>
  </Apply>
</Apply>
 </DerivedField>
 <DerivedField name="walking" dataType="double" optype="continuous">
<Apply function="if">
  <Apply function="equal">
    <FieldRef field="lpa_lpa1"/>
    <Constant dataType="string">Yes</Constant>
  </Apply>
  <Constant dataType="double">1</Constant>
  <Constant dataType="double">0</Constant>
</Apply>
 </DerivedField>
 <DerivedField name="walking_t" dataType="double" optype="continuous">
<Apply function="if">
  <Apply function="is.na">
    <FieldRef field="lpat_lpa1"/>
  </Apply>
  <Constant dataType="double">0</Constant>
  <FieldRef field="lpat_lpa1"/>
</Apply>
 </DerivedField>
 <DerivedField name="walking_h" dataType="double" optype="continuous">
<Apply function="if">
  <Apply function="is.na">
    <FieldRef field="lpam_lpa1"/>
  </Apply>
  <Constant dataType="double">0</Constant>
  <Apply function="if">
    <Apply function="equal">
      <FieldRef field="lpam_lpa1"/>
      <Constant dataType="string">lpa1</Constant>
    </Apply>
    <Constant dataType="double">0.2167</Constant>
    <Apply function="if">
      <Apply function="equal">
        <FieldRef field="lpam_lpa1"/>
        <Constant dataType="string">lpa2</Constant>
      </Apply>
      <Constant dataType="double">0.3833</Constant>
      <Apply function="if">
        <Apply function="equal">
          <FieldRef field="lpam_lpa1"/>
          <Constant dataType="string">lpa3</Constant>
        </Apply>
        <Constant dataType="double">0.75</Constant>
        <Apply function="if">
          <Apply function="equal">
            <FieldRef field="lpam_lpa1"/>
            <Constant dataType="string">lpa4</Constant>
          </Apply>
          <Constant dataType="double">1</Constant>
          <Constant dataType="double">0</Constant>
        </Apply>
      </Apply>
    </Apply>
  </Apply>
</Apply>
 </DerivedField>
 <DerivedField name="garden" dataType="double" optype="continuous">
```

```
<Apply function="if">
  <Apply function="equal">
    <FieldRef field="lpa_lpa2"/>
    <Constant dataType="string">Yes</Constant>
  </Apply>
  <Constant dataType="double">1</Constant>
  <Constant dataType="double">0</Constant>
</Apply>
 </DerivedField>
 <DerivedField name="garden_t" dataType="double" optype="continuous">
<Apply function="if">
  <Apply function="is.na">
    <FieldRef field="lpat_lpa2"/>
  </Apply>
  <Constant dataType="double">0</Constant>
  <FieldRef field="lpat_lpa2"/>
</Apply>
 </DerivedField>
 <DerivedField name="garden_h" dataType="double" optype="continuous">
<Apply function="if">
  <Apply function="is.na">
    <FieldRef field="lpam_lpa2"/>
  </Apply>
  <Constant dataType="double">0</Constant>
  <Apply function="if">
    <Apply function="equal">
      <FieldRef field="lpam_lpa2"/>
      <Constant dataType="string">lpa15</Constant>
    </Apply>
    <Constant dataType="double">0.2167</Constant>
    <Apply function="if">
      <Apply function="equal">
        <FieldRef field="lpam_lpa2"/>
        <Constant dataType="string">lpa30</Constant>
      </Apply>
      <Constant dataType="double">0.3833</Constant>
      <Apply function="if">
        <Apply function="equal">
          <FieldRef field="lpam_lpa2"/>
          <Constant dataType="string">lpa60</Constant>
        </Apply>
        <Constant dataType="double">0.75</Constant>
        <Apply function="if">
          <Apply function="equal">
            <FieldRef field="lpam_lpa2"/>
            <Constant dataType="string">lpa61</Constant>
          </Apply>
          <Constant dataType="double">1</Constant>
          <Constant dataType="double">0</Constant>
        </Apply>
      </Apply>
    </Apply>
  </Apply>
</Apply>
 </DerivedField>
 <DerivedField name="swim" dataType="double" optype="continuous">
<Apply function="if">
  <Apply function="equal">
    <FieldRef field="lpa_lpa3"/>
    <Constant dataType="string">Yes</Constant>
  </Apply>
```

```
    <Constant dataType="double">1</Constant>
    <Constant dataType="double">0</Constant>
  </Apply>
   </DerivedField>
   <DerivedField name="swim_t" dataType="double" optype="continuous">
  <Apply function="if">
    <Apply function="is.na">
      <FieldRef field="lpat_lpa3"/>
    </Apply>
    <Constant dataType="double">0</Constant>
    <FieldRef field="lpat_lpa3"/>
  </Apply>
   </DerivedField>
   <DerivedField name="swim_h" dataType="double" optype="continuous">
  <Apply function="if">
    <Apply function="is.na">
      <FieldRef field="lpam_lpa3"/>
    </Apply>
    <Constant dataType="double">0</Constant>
    <Apply function="if">
      <Apply function="equal">
        <FieldRef field="lpam_lpa3"/>
        <Constant dataType="string">lpa15</Constant>
      </Apply>
      <Constant dataType="double">0.2167</Constant>
      <Apply function="if">
        <Apply function="equal">
          <FieldRef field="lpam_lpa3"/>
          <Constant dataType="string">lpa30</Constant>
        </Apply>
        <Constant dataType="double">0.3833</Constant>
        <Apply function="if">
          <Apply function="equal">
            <FieldRef field="lpam_lpa3"/>
            <Constant dataType="string">lpa60</Constant>
          </Apply>
          <Constant dataType="double">0.75</Constant>
          <Apply function="if">
            <Apply function="equal">
              <FieldRef field="lpam_lpa3"/>
              <Constant dataType="string">lpa61</Constant>
            </Apply>
            <Constant dataType="double">1</Constant>
            <Constant dataType="double">0</Constant>
          </Apply>
        </Apply>
      </Apply>
    </Apply>
  </Apply>
   </DerivedField>
   <DerivedField name="bike" dataType="double" optype="continuous">
  <Apply function="if">
    <Apply function="equal">
      <FieldRef field="lpa_lpa4"/>
      <Constant dataType="string">Yes</Constant>
    </Apply>
    <Constant dataType="double">1</Constant>
    <Constant dataType="double">0</Constant>
  </Apply>
   </DerivedField>
   <DerivedField name="bike_t" dataType="double" optype="continuous">
```

```
<Apply function="if">
  <Apply function="is.na">
    <FieldRef field="lpat_lpa4"/>
  </Apply>
  <Constant dataType="double">0</Constant>
  <FieldRef field="lpat_lpa4"/>
</Apply>
 </DerivedField>
 <DerivedField name="bike_h" dataType="double" optype="continuous">
<Apply function="if">
  <Apply function="is.na">
    <FieldRef field="lpam_lpa4"/>
  </Apply>
  <Constant dataType="double">0</Constant>
  <Apply function="if">
    <Apply function="equal">
      <FieldRef field="lpam_lpa4"/>
      <Constant dataType="string">lpa15</Constant>
    </Apply>
    <Constant dataType="double">0.2167</Constant>
    <Apply function="if">
      <Apply function="equal">
        <FieldRef field="lpam_lpa4"/>
        <Constant dataType="string">lpa30</Constant>
      </Apply>
      <Constant dataType="double">0.3833</Constant>
      <Apply function="if">
        <Apply function="equal">
          <FieldRef field="lpam_lpa4"/>
          <Constant dataType="string">lpa60</Constant>
        </Apply>
        <Constant dataType="double">0.75</Constant>
        <Apply function="if">
          <Apply function="equal">
            <FieldRef field="lpam_lpa4"/>
            <Constant dataType="string">lpa61</Constant>
          </Apply>
          <Constant dataType="double">1</Constant>
          <Constant dataType="double">0</Constant>
        </Apply>
      </Apply>
    </Apply>
  </Apply>
</Apply>
 </DerivedField>
 <DerivedField name="dance" dataType="double" optype="continuous">
<Apply function="if">
  <Apply function="equal">
    <FieldRef field="lpa_lpa5"/>
    <Constant dataType="string">Yes</Constant>
  </Apply>
  <Constant dataType="double">1</Constant>
  <Constant dataType="double">0</Constant>
</Apply>
 </DerivedField>
 <DerivedField name="dance_t" dataType="double" optype="continuous">
<Apply function="if">
  <Apply function="is.na">
    <FieldRef field="lpat_lpa5"/>
  </Apply>
  <Constant dataType="double">0</Constant>
```

```
  <FieldRef field="lpat_lpa5"/>
</Apply>
 </DerivedField>
 <DerivedField name="dance_h" dataType="double" optype="continuous">
<Apply function="if">
  <Apply function="is.na">
    <FieldRef field="lpam_lpa5"/>
  </Apply>
  <Constant dataType="double">0</Constant>
  <Apply function="if">
    <Apply function="equal">
      <FieldRef field="lpam_lpa5"/>
      <Constant dataType="string">lpa15</Constant>
    </Apply>
    <Constant dataType="double">0.2167</Constant>
    <Apply function="if">
      <Apply function="equal">
        <FieldRef field="lpam_lpa5"/>
        <Constant dataType="string">lpa30</Constant>
      </Apply>
      <Constant dataType="double">0.3833</Constant>
      <Apply function="if">
        <Apply function="equal">
          <FieldRef field="lpam_lpa5"/>
          <Constant dataType="string">lpa60</Constant>
        </Apply>
        <Constant dataType="double">0.75</Constant>
        <Apply function="if">
          <Apply function="equal">
            <FieldRef field="lpam_lpa5"/>
            <Constant dataType="string">lpa61</Constant>
          </Apply>
          <Constant dataType="double">1</Constant>
          <Constant dataType="double">0</Constant>
        </Apply>
      </Apply>
    </Apply>
  </Apply>
</Apply>
 </DerivedField>
 <DerivedField name="hexercises" dataType="double" optype="continuous">
<Apply function="if">
  <Apply function="equal">
    <FieldRef field="lpa_lpa6"/>
    <Constant dataType="string">Yes</Constant>
  </Apply>
  <Constant dataType="double">1</Constant>
  <Constant dataType="double">0</Constant>
</Apply>
 </DerivedField>
 <DerivedField name="hexercises_t" dataType="double" optype="continuous">
<Apply function="if">
  <Apply function="is.na">
    <FieldRef field="lpat_lpa6"/>
  </Apply>
  <Constant dataType="double">0</Constant>
  <FieldRef field="lpat_lpa6"/>
</Apply>
 </DerivedField>
 <DerivedField name="hexercises_h" dataType="double" optype="continuous">
<Apply function="if">
```

```
    <Apply function="is.na">
      <FieldRef field="lpam_lpa6"/>
    </Apply>
    <Constant dataType="double">0</Constant>
    <Apply function="if">
      <Apply function="equal">
        <FieldRef field="lpam_lpa6"/>
        <Constant dataType="string">lpa15</Constant>
      </Apply>
      <Constant dataType="double">0.2167</Constant>
      <Apply function="if">
        <Apply function="equal">
          <FieldRef field="lpam_lpa6"/>
          <Constant dataType="string">lpa30</Constant>
        </Apply>
        <Constant dataType="double">0.3833</Constant>
        <Apply function="if">
          <Apply function="equal">
            <FieldRef field="lpam_lpa6"/>
            <Constant dataType="string">lpa60</Constant>
          </Apply>
          <Constant dataType="double">0.75</Constant>
          <Apply function="if">
            <Apply function="equal">
              <FieldRef field="lpam_lpa6"/>
              <Constant dataType="string">lpa61</Constant>
            </Apply>
            <Constant dataType="double">1</Constant>
            <Constant dataType="double">0</Constant>
          </Apply>
        </Apply>
      </Apply>
    </Apply>
  </Apply>
   </DerivedField>
   <DerivedField name="hockey" dataType="double" optype="continuous">
  <Apply function="if">
    <Apply function="equal">
      <FieldRef field="lpa_lpa7"/>
      <Constant dataType="string">Yes</Constant>
    </Apply>
    <Constant dataType="double">1</Constant>
    <Constant dataType="double">0</Constant>
  </Apply>
   </DerivedField>
   <DerivedField name="hockey_t" dataType="double" optype="continuous">
  <Apply function="if">
    <Apply function="is.na">
      <FieldRef field="lpat_lpa7"/>
    </Apply>
    <Constant dataType="double">0</Constant>
    <FieldRef field="lpat_lpa7"/>
  </Apply>
   </DerivedField>
   <DerivedField name="hockey_h" dataType="double" optype="continuous">
  <Apply function="if">
    <Apply function="is.na">
      <FieldRef field="lpam_lpa7"/>
    </Apply>
    <Constant dataType="double">0</Constant>
    <Apply function="if">
```

```
    <Apply function="equal">
      <FieldRef field="lpam_lpa7"/>
      <Constant dataType="string">lpa15</Constant>
    </Apply>
    <Constant dataType="double">0.2167</Constant>
    <Apply function="if">
      <Apply function="equal">
        <FieldRef field="lpam_lpa7"/>
        <Constant dataType="string">lpa30</Constant>
      </Apply>
      <Constant dataType="double">0.3833</Constant>
      <Apply function="if">
        <Apply function="equal">
          <FieldRef field="lpam_lpa7"/>
          <Constant dataType="string">lpa60</Constant>
        </Apply>
        <Constant dataType="double">0.75</Constant>
        <Apply function="if">
          <Apply function="equal">
            <FieldRef field="lpam_lpa7"/>
            <Constant dataType="string">lpa61</Constant>
          </Apply>
          <Constant dataType="double">1</Constant>
          <Constant dataType="double">0</Constant>
        </Apply>
      </Apply>
    </Apply>
  </Apply>
</Apply>
 </DerivedField>
 <DerivedField name="skate" dataType="double" optype="continuous">
<Apply function="if">
  <Apply function="equal">
    <FieldRef field="lpa_lpa8"/>
    <Constant dataType="string">Yes</Constant>
  </Apply>
  <Constant dataType="double">1</Constant>
  <Constant dataType="double">0</Constant>
</Apply>
 </DerivedField>
 <DerivedField name="skate_t" dataType="double" optype="continuous">
<Apply function="if">
  <Apply function="is.na">
    <FieldRef field="lpat_lpa8"/>
  </Apply>
  <Constant dataType="double">0</Constant>
  <FieldRef field="lpat_lpa8"/>
</Apply>
 </DerivedField>
 <DerivedField name="skate_h" dataType="double" optype="continuous">
<Apply function="if">
  <Apply function="is.na">
    <FieldRef field="lpam_lpa8"/>
  </Apply>
  <Constant dataType="double">0</Constant>
  <Apply function="if">
    <Apply function="equal">
      <FieldRef field="lpam_lpa8"/>
      <Constant dataType="string">lpa15</Constant>
    </Apply>
    <Constant dataType="double">0.2167</Constant>
```

```
    <Apply function="if">
      <Apply function="equal">
        <FieldRef field="lpam_lpa8"/>
        <Constant dataType="string">lpa30</Constant>
      </Apply>
      <Constant dataType="double">0.3833</Constant>
      <Apply function="if">
        <Apply function="equal">
          <FieldRef field="lpam_lpa8"/>
          <Constant dataType="string">lpa60</Constant>
        </Apply>
        <Constant dataType="double">0.75</Constant>
        <Apply function="if">
          <Apply function="equal">
            <FieldRef field="lpam_lpa8"/>
            <Constant dataType="string">lpa61</Constant>
          </Apply>
          <Constant dataType="double">1</Constant>
          <Constant dataType="double">0</Constant>
        </Apply>
      </Apply>
    </Apply>
  </Apply>
</Apply>
 </DerivedField>
 <DerivedField name="inline" dataType="double" optype="continuous">
<Apply function="if">
  <Apply function="equal">
    <FieldRef field="lpa_lpa9"/>
    <Constant dataType="string">Yes</Constant>
  </Apply>
  <Constant dataType="double">1</Constant>
  <Constant dataType="double">0</Constant>
</Apply>
 </DerivedField>
 <DerivedField name="inline_t" dataType="double" optype="continuous">
<Apply function="if">
  <Apply function="is.na">
    <FieldRef field="lpat_lpa9"/>
  </Apply>
  <Constant dataType="double">0</Constant>
  <FieldRef field="lpat_lpa9"/>
</Apply>
 </DerivedField>
 <DerivedField name="inline_h" dataType="double" optype="continuous">
<Apply function="if">
  <Apply function="is.na">
    <FieldRef field="lpam_lpa9"/>
  </Apply>
  <Constant dataType="double">0</Constant>
  <Apply function="if">
    <Apply function="equal">
      <FieldRef field="lpam_lpa9"/>
      <Constant dataType="string">lpa15</Constant>
    </Apply>
    <Constant dataType="double">0.2167</Constant>
    <Apply function="if">
      <Apply function="equal">
        <FieldRef field="lpam_lpa9"/>
        <Constant dataType="string">lpa30</Constant>
      </Apply>
```

```
      <Constant dataType="double">0.3833</Constant>
      <Apply function="if">
        <Apply function="equal">
          <FieldRef field="lpam_lpa9"/>
          <Constant dataType="string">lpa60</Constant>
        </Apply>
        <Constant dataType="double">0.75</Constant>
        <Apply function="if">
          <Apply function="equal">
            <FieldRef field="lpat_lpa9"/>
            <Constant dataType="string">lpa61</Constant>
          </Apply>
          <Constant dataType="double">1</Constant>
          <Constant dataType="double">0</Constant>
        </Apply>
      </Apply>
    </Apply>
  </Apply>
</Apply>
 </DerivedField>
 <DerivedField name="jogrun" dataType="double" optype="continuous">
<Apply function="if">
  <Apply function="equal">
    <FieldRef field="lpa_lpa10"/>
    <Constant dataType="string">Yes</Constant>
  </Apply>
  <Constant dataType="double">1</Constant>
  <Constant dataType="double">0</Constant>
</Apply>
 </DerivedField>
 <DerivedField name="jogrun_t" dataType="double" optype="continuous">
<Apply function="if">
  <Apply function="is.na">
    <FieldRef field="lpat_lpa10"/>
  </Apply>
  <Constant dataType="double">0</Constant>
  <FieldRef field="lpat_lpa10"/>
</Apply>
 </DerivedField>
 <DerivedField name="jogrun_h" dataType="double" optype="continuous">
<Apply function="if">
  <Apply function="is.na">
    <FieldRef field="lpat_lpa10"/>
  </Apply>
  <Constant dataType="double">0</Constant>
  <Apply function="if">
    <Apply function="equal">
      <FieldRef field="lpat_lpa10"/>
      <Constant dataType="string">lpa15</Constant>
    </Apply>
    <Constant dataType="double">0.2167</Constant>
    <Apply function="if">
      <Apply function="equal">
        <FieldRef field="lpat_lpa10"/>
        <Constant dataType="string">lpa30</Constant>
      </Apply>
      <Constant dataType="double">0.3833</Constant>
      <Apply function="if">
        <Apply function="equal">
          <FieldRef field="lpat_lpa10"/>
          <Constant dataType="string">lpa60</Constant>
```

```
        </Apply>
        <Constant dataType="double">0.75</Constant>
        <Apply function="if">
          <Apply function="equal">
            <FieldRef field="lpat_lpa10"/>
            <Constant dataType="string">lpa61</Constant>
          </Apply>
          <Constant dataType="double">1</Constant>
          <Constant dataType="double">0</Constant>
        </Apply>
      </Apply>
    </Apply>
  </Apply>
</Apply>
 </DerivedField>
 <DerivedField name="golf" dataType="double" optype="continuous">
<Apply function="if">
  <Apply function="equal">
    <FieldRef field="lpa_lpa11"/>
    <Constant dataType="string">Yes</Constant>
  </Apply>
  <Constant dataType="double">1</Constant>
  <Constant dataType="double">0</Constant>
</Apply>
 </DerivedField>
 <DerivedField name="golf_t" dataType="double" optype="continuous">
<Apply function="if">
  <Apply function="is.na">
    <FieldRef field="lpat_lpa11"/>
  </Apply>
  <Constant dataType="double">0</Constant>
  <FieldRef field="lpat_lpa11"/>
</Apply>
 </DerivedField>
 <DerivedField name="golf_h" dataType="double" optype="continuous">
<Apply function="if">
  <Apply function="is.na">
    <FieldRef field="lpam_lpa11"/>
  </Apply>
  <Constant dataType="double">0</Constant>
  <Apply function="if">
    <Apply function="equal">
      <FieldRef field="lpam_lpa11"/>
      <Constant dataType="string">lpa15</Constant>
    </Apply>
    <Constant dataType="double">0.2167</Constant>
    <Apply function="if">
      <Apply function="equal">
        <FieldRef field="lpam_lpa11"/>
        <Constant dataType="string">lpa30</Constant>
      </Apply>
      <Constant dataType="double">0.3833</Constant>
      <Apply function="if">
        <Apply function="equal">
          <FieldRef field="lpam_lpa11"/>
          <Constant dataType="string">lpa60</Constant>
        </Apply>
        <Constant dataType="double">0.75</Constant>
        <Apply function="if">
          <Apply function="equal">
            <FieldRef field="lpam_lpa11"/>
```

```
            <Constant dataType="string">lpa61</Constant>
          </Apply>
          <Constant dataType="double">1</Constant>
          <Constant dataType="double">0</Constant>
        </Apply>
      </Apply>
    </Apply>
  </Apply>
</Apply>
 </DerivedField>
 <DerivedField name="aerobics" dataType="double" optype="continuous">
<Apply function="if">
  <Apply function="equal">
    <FieldRef field="lpa_lpa12"/>
    <Constant dataType="string">Yes</Constant>
  </Apply>
  <Constant dataType="double">1</Constant>
  <Constant dataType="double">0</Constant>
</Apply>
 </DerivedField>
 <DerivedField name="aerobics_t" dataType="double" optype="continuous">
<Apply function="if">
  <Apply function="is.na">
    <FieldRef field="lpat_lpa12"/>
  </Apply>
  <Constant dataType="double">0</Constant>
  <FieldRef field="lpat_lpa12"/>
</Apply>
 </DerivedField>
 <DerivedField name="aerobics_h" dataType="double" optype="continuous">
<Apply function="if">
  <Apply function="is.na">
    <FieldRef field="lpam_lpa12"/>
  </Apply>
  <Constant dataType="double">0</Constant>
  <Apply function="if">
    <Apply function="equal">
      <FieldRef field="lpam_lpa12"/>
      <Constant dataType="string">lpa15</Constant>
    </Apply>
    <Constant dataType="double">0.2167</Constant>
    <Apply function="if">
      <Apply function="equal">
        <FieldRef field="lpam_lpa12"/>
        <Constant dataType="string">lpa30</Constant>
      </Apply>
      <Constant dataType="double">0.3833</Constant>
      <Apply function="if">
        <Apply function="equal">
          <FieldRef field="lpam_lpa12"/>
          <Constant dataType="string">lpa60</Constant>
        </Apply>
        <Constant dataType="double">0.75</Constant>
        <Apply function="if">
          <Apply function="equal">
            <FieldRef field="lpam_lpa12"/>
            <Constant dataType="string">lpa61</Constant>
          </Apply>
          <Constant dataType="double">1</Constant>
          <Constant dataType="double">0</Constant>
        </Apply>
```

```
      </Apply>
    </Apply>
  </Apply>
</Apply>
 </DerivedField>
 <DerivedField name="ski" dataType="double" optype="continuous">
<Apply function="if">
  <Apply function="equal">
    <FieldRef field="lpa_lpa13"/>
    <Constant dataType="string">Yes</Constant>
  </Apply>
  <Constant dataType="double">1</Constant>
  <Constant dataType="double">0</Constant>
</Apply>
 </DerivedField>
 <DerivedField name="ski_t" dataType="double" optype="continuous">
<Apply function="if">
  <Apply function="is.na">
    <FieldRef field="lpat_lpa13"/>
  </Apply>
  <Constant dataType="double">0</Constant>
  <FieldRef field="lpat_lpa13"/>
</Apply>
 </DerivedField>
 <DerivedField name="ski_h" dataType="double" optype="continuous">
<Apply function="if">
  <Apply function="is.na">
    <FieldRef field="lpam_lpa13"/>
  </Apply>
  <Constant dataType="double">0</Constant>
  <Apply function="if">
    <Apply function="equal">
      <FieldRef field="lpam_lpa13"/>
      <Constant dataType="string">lpa15</Constant>
    </Apply>
    <Constant dataType="double">0.2167</Constant>
    <Apply function="if">
      <Apply function="equal">
        <FieldRef field="lpam_lpa13"/>
        <Constant dataType="string">lpa30</Constant>
      </Apply>
      <Constant dataType="double">0.3833</Constant>
      <Apply function="if">
        <Apply function="equal">
          <FieldRef field="lpam_lpa13"/>
          <Constant dataType="string">lpa60</Constant>
        </Apply>
        <Constant dataType="double">0.75</Constant>
        <Apply function="if">
          <Apply function="equal">
            <FieldRef field="lpam_lpa13"/>
            <Constant dataType="string">lpa61</Constant>
          </Apply>
          <Constant dataType="double">1</Constant>
          <Constant dataType="double">0</Constant>
        </Apply>
      </Apply>
    </Apply>
  </Apply>
</Apply>
 </DerivedField>
```

```
 <DerivedField name="bowl" dataType="double" optype="continuous">
<Apply function="if">
  <Apply function="equal">
    <FieldRef field="lpa_lpa14"/>
    <Constant dataType="string">Yes</Constant>
  </Apply>
  <Constant dataType="double">1</Constant>
  <Constant dataType="double">0</Constant>
</Apply>
 </DerivedField>
 <DerivedField name="bowl_t" dataType="double" optype="continuous">
<Apply function="if">
  <Apply function="is.na">
    <FieldRef field="lpat_lpa14"/>
  </Apply>
  <Constant dataType="double">0</Constant>
  <FieldRef field="lpat_lpa14"/>
</Apply>
 </DerivedField>
 <DerivedField name="bowl_h" dataType="double" optype="continuous">
<Apply function="if">
  <Apply function="is.na">
    <FieldRef field="lpam_lpa14"/>
  </Apply>
  <Constant dataType="double">0</Constant>
  <Apply function="if">
    <Apply function="equal">
      <FieldRef field="lpam_lpa14"/>
      <Constant dataType="string">lpa15</Constant>
    </Apply>
    <Constant dataType="double">0.2167</Constant>
    <Apply function="if">
      <Apply function="equal">
        <FieldRef field="lpam_lpa14"/>
        <Constant dataType="string">lpa30</Constant>
      </Apply>
      <Constant dataType="double">0.3833</Constant>
      <Apply function="if">
        <Apply function="equal">
          <FieldRef field="lpam_lpa14"/>
          <Constant dataType="string">lpa60</Constant>
        </Apply>
        <Constant dataType="double">0.75</Constant>
        <Apply function="if">
          <Apply function="equal">
            <FieldRef field="lpam_lpa14"/>
            <Constant dataType="string">lpa61</Constant>
          </Apply>
          <Constant dataType="double">1</Constant>
          <Constant dataType="double">0</Constant>
        </Apply>
      </Apply>
    </Apply>
  </Apply>
</Apply>
 </DerivedField>
 <DerivedField name="baseball" dataType="double" optype="continuous">
<Apply function="if">
  <Apply function="equal">
    <FieldRef field="lpa_lpa15"/>
    <Constant dataType="string">Yes</Constant>
```

```
  </Apply>
  <Constant dataType="double">1</Constant>
  <Constant dataType="double">0</Constant>
</Apply>
 </DerivedField>
 <DerivedField name="baseball_t" dataType="double" optype="continuous">
<Apply function="if">
  <Apply function="is.na">
    <FieldRef field="lpat_lpa15"/>
  </Apply>
  <Constant dataType="double">0</Constant>
  <FieldRef field="lpat_lpa15"/>
</Apply>
 </DerivedField>
 <DerivedField name="baseball_h" dataType="double" optype="continuous">
<Apply function="if">
  <Apply function="is.na">
    <FieldRef field="lpam_lpa15"/>
  </Apply>
  <Constant dataType="double">0</Constant>
  <Apply function="if">
    <Apply function="equal">
      <FieldRef field="lpam_lpa15"/>
      <Constant dataType="string">lpa15</Constant>
    </Apply>
    <Constant dataType="double">0.2167</Constant>
    <Apply function="if">
      <Apply function="equal">
        <FieldRef field="lpam_lpa15"/>
        <Constant dataType="string">lpa30</Constant>
      </Apply>
      <Constant dataType="double">0.3833</Constant>
      <Apply function="if">
        <Apply function="equal">
          <FieldRef field="lpam_lpa15"/>
          <Constant dataType="string">lpa60</Constant>
        </Apply>
        <Constant dataType="double">0.75</Constant>
        <Apply function="if">
          <Apply function="equal">
            <FieldRef field="lpam_lpa15"/>
            <Constant dataType="string">lpa61</Constant>
          </Apply>
          <Constant dataType="double">1</Constant>
          <Constant dataType="double">0</Constant>
        </Apply>
      </Apply>
    </Apply>
  </Apply>
</Apply>
 </DerivedField>
 <DerivedField name="tennis" dataType="double" optype="continuous">
<Apply function="if">
  <Apply function="equal">
    <FieldRef field="lpa_lpa16"/>
    <Constant dataType="string">Yes</Constant>
  </Apply>
  <Constant dataType="double">1</Constant>
  <Constant dataType="double">0</Constant>
</Apply>
 </DerivedField>
```

```
 <DerivedField name="tennis_t" dataType="double" optype="continuous">
<Apply function="if">
  <Apply function="is.na">
    <FieldRef field="lpat_lpa16"/>
  </Apply>
  <Constant dataType="double">0</Constant>
  <FieldRef field="lpat_lpa16"/>
</Apply>
 </DerivedField>
 <DerivedField name="tennis_h" dataType="double" optype="continuous">
<Apply function="if">
  <Apply function="is.na">
    <FieldRef field="lpam_lpa16"/>
  </Apply>
  <Constant dataType="double">0</Constant>
  <Apply function="if">
    <Apply function="equal">
      <FieldRef field="lpam_lpa16"/>
      <Constant dataType="string">lpa15</Constant>
    </Apply>
    <Constant dataType="double">0.2167</Constant>
    <Apply function="if">
      <Apply function="equal">
        <FieldRef field="lpam_lpa16"/>
        <Constant dataType="string">lpa30</Constant>
      </Apply>
      <Constant dataType="double">0.3833</Constant>
      <Apply function="if">
        <Apply function="equal">
          <FieldRef field="lpam_lpa16"/>
          <Constant dataType="string">lpa60</Constant>
        </Apply>
        <Constant dataType="double">0.75</Constant>
        <Apply function="if">
          <Apply function="equal">
            <FieldRef field="lpam_lpa16"/>
            <Constant dataType="string">lpa61</Constant>
          </Apply>
          <Constant dataType="double">1</Constant>
          <Constant dataType="double">0</Constant>
        </Apply>
      </Apply>
    </Apply>
  </Apply>
</Apply>
 </DerivedField>
 <DerivedField name="weights" dataType="double" optype="continuous">
<Apply function="if">
  <Apply function="equal">
    <FieldRef field="lpa_lpa17"/>
    <Constant dataType="string">Yes</Constant>
  </Apply>
  <Constant dataType="double">1</Constant>
  <Constant dataType="double">0</Constant>
</Apply>
 </DerivedField>
 <DerivedField name="weights_t" dataType="double" optype="continuous">
<Apply function="if">
  <Apply function="is.na">
    <FieldRef field="lpat_lpa17"/>
  </Apply>
```

```
  <Constant dataType="double">0</Constant>
  <FieldRef field="lpat_lpa17"/>
</Apply>
 </DerivedField>
 <DerivedField name="weights_h" dataType="double" optype="continuous">
<Apply function="if">
  <Apply function="is.na">
    <FieldRef field="lpam_lpa17"/>
  </Apply>
  <Constant dataType="double">0</Constant>
  <Apply function="if">
    <Apply function="equal">
      <FieldRef field="lpam_lpa17"/>
      <Constant dataType="string">lpa15</Constant>
    </Apply>
    <Constant dataType="double">0.2167</Constant>
    <Apply function="if">
      <Apply function="equal">
        <FieldRef field="lpam_lpa17"/>
        <Constant dataType="string">lpa30</Constant>
      </Apply>
      <Constant dataType="double">0.3833</Constant>
      <Apply function="if">
        <Apply function="equal">
          <FieldRef field="lpam_lpa17"/>
          <Constant dataType="string">lpa60</Constant>
        </Apply>
        <Constant dataType="double">0.75</Constant>
        <Apply function="if">
          <Apply function="equal">
            <FieldRef field="lpam_lpa17"/>
            <Constant dataType="string">lpa61</Constant>
          </Apply>
          <Constant dataType="double">1</Constant>
          <Constant dataType="double">0</Constant>
        </Apply>
      </Apply>
    </Apply>
  </Apply>
</Apply>
 </DerivedField>
 <DerivedField name="fishing" dataType="double" optype="continuous">
<Apply function="if">
  <Apply function="equal">
    <FieldRef field="lpa_lpa18"/>
    <Constant dataType="string">Yes</Constant>
  </Apply>
  <Constant dataType="double">1</Constant>
  <Constant dataType="double">0</Constant>
</Apply>
 </DerivedField>
 <DerivedField name="fishing_t" dataType="double" optype="continuous">
<Apply function="if">
  <Apply function="is.na">
    <FieldRef field="lpat_lpa18"/>
  </Apply>
  <Constant dataType="double">0</Constant>
  <FieldRef field="lpat_lpa18"/>
</Apply>
 </DerivedField>
 <DerivedField name="fishing_h" dataType="double" optype="continuous">
```

```
<Apply function="if">
  <Apply function="is.na">
    <FieldRef field="lpam_lpa18"/>
  </Apply>
  <Constant dataType="double">0</Constant>
  <Apply function="if">
    <Apply function="equal">
      <FieldRef field="lpam_lpa18"/>
      <Constant dataType="string">lpa15</Constant>
    </Apply>
    <Constant dataType="double">0.2167</Constant>
    <Apply function="if">
      <Apply function="equal">
        <FieldRef field="lpam_lpa18"/>
        <Constant dataType="string">lpa30</Constant>
      </Apply>
      <Constant dataType="double">0.3833</Constant>
      <Apply function="if">
        <Apply function="equal">
          <FieldRef field="lpam_lpa18"/>
          <Constant dataType="string">lpa60</Constant>
        </Apply>
        <Constant dataType="double">0.75</Constant>
        <Apply function="if">
          <Apply function="equal">
            <FieldRef field="lpam_lpa18"/>
            <Constant dataType="string">lpa61</Constant>
          </Apply>
          <Constant dataType="double">1</Constant>
          <Constant dataType="double">0</Constant>
        </Apply>
      </Apply>
    </Apply>
  </Apply>
</Apply>
 </DerivedField>
 <DerivedField name="volleyball" dataType="double" optype="continuous">
<Apply function="if">
  <Apply function="equal">
    <FieldRef field="lpa_lpa19"/>
    <Constant dataType="string">Yes</Constant>
  </Apply>
  <Constant dataType="double">1</Constant>
  <Constant dataType="double">0</Constant>
</Apply>
 </DerivedField>
 <DerivedField name="volleyball_t" dataType="double" optype="continuous">
<Apply function="if">
  <Apply function="is.na">
    <FieldRef field="lpat_lpa19"/>
  </Apply>
  <Constant dataType="double">0</Constant>
  <FieldRef field="lpat_lpa19"/>
</Apply>
 </DerivedField>
 <DerivedField name="volleyball_h" dataType="double" optype="continuous">
<Apply function="if">
  <Apply function="is.na">
    <FieldRef field="lpam_lpa19"/>
  </Apply>
  <Constant dataType="double">0</Constant>
```

```
  <Apply function="if">
    <Apply function="equal">
      <FieldRef field="lpam_lpa19"/>
      <Constant dataType="string">lpa15</Constant>
    </Apply>
    <Constant dataType="double">0.2167</Constant>
    <Apply function="if">
      <Apply function="equal">
        <FieldRef field="lpam_lpa19"/>
        <Constant dataType="string">lpa30</Constant>
      </Apply>
      <Constant dataType="double">0.3833</Constant>
      <Apply function="if">
        <Apply function="equal">
          <FieldRef field="lpam_lpa19"/>
          <Constant dataType="string">lpa60</Constant>
        </Apply>
        <Constant dataType="double">0.75</Constant>
        <Apply function="if">
          <Apply function="equal">
            <FieldRef field="lpam_lpa19"/>
            <Constant dataType="string">lpa61</Constant>
          </Apply>
          <Constant dataType="double">1</Constant>
          <Constant dataType="double">0</Constant>
        </Apply>
      </Apply>
    </Apply>
  </Apply>
</Apply>
 </DerivedField>
 <DerivedField name="basketball" dataType="double" optype="continuous">
<Apply function="if">
  <Apply function="equal">
    <FieldRef field="lpa_lpa20"/>
    <Constant dataType="string">Yes</Constant>
  </Apply>
  <Constant dataType="double">1</Constant>
  <Constant dataType="double">0</Constant>
</Apply>
 </DerivedField>
 <DerivedField name="basketball_t" dataType="double" optype="continuous">
<Apply function="if">
  <Apply function="is.na">
    <FieldRef field="lpat_lpa20"/>
  </Apply>
  <Constant dataType="double">0</Constant>
  <FieldRef field="lpat_lpa20"/>
</Apply>
 </DerivedField>
 <DerivedField name="basketball_h" dataType="double" optype="continuous">
<Apply function="if">
  <Apply function="is.na">
    <FieldRef field="lpam_lpa20"/>
  </Apply>
  <Constant dataType="double">0</Constant>
  <Apply function="if">
    <Apply function="equal">
      <FieldRef field="lpam_lpa20"/>
      <Constant dataType="string">lpa15</Constant>
    </Apply>
```

```
    <Constant dataType="double">0.2167</Constant>
    <Apply function="if">
      <Apply function="equal">
        <FieldRef field="lpam_lpa20"/>
        <Constant dataType="string">lpa30</Constant>
      </Apply>
      <Constant dataType="double">0.3833</Constant>
      <Apply function="if">
        <Apply function="equal">
          <FieldRef field="lpam_lpa20"/>
          <Constant dataType="string">lpa60</Constant>
        </Apply>
        <Constant dataType="double">0.75</Constant>
        <Apply function="if">
          <Apply function="equal">
            <FieldRef field="lpam_lpa20"/>
            <Constant dataType="string">lpa61</Constant>
          </Apply>
          <Constant dataType="double">1</Constant>
          <Constant dataType="double">0</Constant>
        </Apply>
      </Apply>
    </Apply>
  </Apply>
</Apply>
 </DerivedField>
 <DerivedField name="soccer" dataType="double" optype="continuous">
<Apply function="if">
  <Apply function="equal">
    <FieldRef field="lpa_lpa21"/>
    <Constant dataType="string">Yes</Constant>
  </Apply>
  <Constant dataType="double">1</Constant>
  <Constant dataType="double">0</Constant>
</Apply>
 </DerivedField>
 <DerivedField name="soccer_t" dataType="double" optype="continuous">
<Apply function="if">
  <Apply function="is.na">
    <FieldRef field="lpat_lpa21"/>
  </Apply>
  <Constant dataType="double">0</Constant>
  <FieldRef field="lpat_lpa21"/>
</Apply>
 </DerivedField>
 <DerivedField name="soccer_h" dataType="double" optype="continuous">
<Apply function="if">
  <Apply function="is.na">
    <FieldRef field="lpam_lpa21"/>
  </Apply>
  <Constant dataType="double">0</Constant>
  <Apply function="if">
    <Apply function="equal">
      <FieldRef field="lpam_lpa21"/>
      <Constant dataType="string">lpa15</Constant>
    </Apply>
    <Constant dataType="double">0.2167</Constant>
    <Apply function="if">
      <Apply function="equal">
        <FieldRef field="lpam_lpa21"/>
        <Constant dataType="string">lpa30</Constant>
```

```
      </Apply>
      <Constant dataType="double">0.3833</Constant>
      <Apply function="if">
        <Apply function="equal">
          <FieldRef field="lpam_lpa21"/>
          <Constant dataType="string">lpa60</Constant>
        </Apply>
        <Constant dataType="double">0.75</Constant>
        <Apply function="if">
          <Apply function="equal">
            <FieldRef field="lpam_lpa21"/>
            <Constant dataType="string">lpa61</Constant>
          </Apply>
          <Constant dataType="double">1</Constant>
          <Constant dataType="double">0</Constant>
        </Apply>
      </Apply>
    </Apply>
  </Apply>
</Apply>
 </DerivedField>
 <DerivedField name="other" dataType="double" optype="continuous">
<Apply function="if">
  <Apply function="equal">
    <FieldRef field="lpa_lpa22"/>
    <Constant dataType="string">Yes</Constant>
  </Apply>
  <Constant dataType="double">1</Constant>
  <Constant dataType="double">0</Constant>
</Apply>
 </DerivedField>
 <DerivedField name="other_t" dataType="double" optype="continuous">
<Apply function="if">
  <Apply function="is.na">
    <FieldRef field="lpat_lpa22"/>
  </Apply>
  <Constant dataType="double">0</Constant>
  <FieldRef field="lpat_lpa22"/>
</Apply>
 </DerivedField>
 <DerivedField name="other_h" dataType="double" optype="continuous">
<Apply function="if">
  <Apply function="is.na">
    <FieldRef field="lpam_lpa22"/>
  </Apply>
  <Constant dataType="double">0</Constant>
  <Apply function="if">
    <Apply function="equal">
      <FieldRef field="lpam_lpa22"/>
      <Constant dataType="string">lpa15</Constant>
    </Apply>
    <Constant dataType="double">0.2167</Constant>
    <Apply function="if">
      <Apply function="equal">
        <FieldRef field="lpat_lpa22"/>
        <Constant dataType="string">lpa30</Constant>
      </Apply>
      <Constant dataType="double">0.3833</Constant>
      <Apply function="if">
        <Apply function="equal">
          <FieldRef field="lpam_lpa22"/>
```

```
            <Constant dataType="string">lpa60</Constant>
          </Apply>
          <Constant dataType="double">0.75</Constant>
          <Apply function="if">
            <Apply function="equal">
              <FieldRef field="lpam_lpa22"/>
              <Constant dataType="string">lpa61</Constant>
            </Apply>
            <Constant dataType="double">1</Constant>
            <Constant dataType="double">0</Constant>
          </Apply>
        </Apply>
      </Apply>
    </Apply>
  </Apply>
 </DerivedField>
 <DerivedField name="PhysicalActivityraw1" dataType="double" optype="continuous">
  <Apply function="+">
    <Apply function="+">
      <Apply function="+">
        <Apply function="+">
          <Apply function="+">
            <Apply function="+">
              <Apply function="+">
                <Apply function="+">
                  <Apply function="+">
                    <Apply function="+">
                      <Apply function="+">
                        <Apply function="+">
                          <Apply function="+">
                            <Apply function="+">
                              <Apply function="+">
                                <Apply function="+">
                                  <Apply function="+">
                                    <Apply function="+">
                                      <Apply function="+">
                                        <Apply function="+">
                                          <Apply function="+">
                                            <Apply function="/">
                                              <Apply function="*">
                                                <Apply function="*">
                                                  <Apply function="*">
                                                    <FieldRef field="walking"/>
                                                    <FieldRef field="walking_h"/>
                                                  </Apply>
                                                  <Constant
dataType="double">3</Constant>
                                                </Apply>
                                                <FieldRef field="walking_t"/>
                                              </Apply>
                                              <Constant dataType="double">90</Constant>
                                            </Apply>
                                            <Apply function="/">
                                              <Apply function="*">
                                                <Apply function="*">
                                                  <Apply function="*">
                                                    <FieldRef field="garden"/>
                                                    <FieldRef field="garden_h"/>
                                                  </Apply>
                                                  <Constant
dataType="double">3</Constant>
```

```
              </Apply>
              <FieldRef field="garden_t"/>
            </Apply>
            <Constant dataType="double">90</Constant>
          </Apply>
        </Apply>
        <Apply function="/">
          <Apply function="*">
            <Apply function="*">
              <Apply function="*">
                <FieldRef field="swim"/>
                <FieldRef field="swim_h"/>
              </Apply>
              <Constant dataType="double">3</Constant>
            </Apply>
            <FieldRef field="swim_t"/>
          </Apply>
          <Constant dataType="double">90</Constant>
        </Apply>
      </Apply>
      <Apply function="/">
        <Apply function="*">
          <Apply function="*">
            <Apply function="*">
              <FieldRef field="bike"/>
              <FieldRef field="bike_h"/>
            </Apply>
            <Constant dataType="double">4</Constant>
          </Apply>
          <FieldRef field="bike_t"/>
        </Apply>
        <Constant dataType="double">90</Constant>
      </Apply>
    </Apply>
    <Apply function="/">
      <Apply function="*">
        <Apply function="*">
          <Apply function="*">
            <FieldRef field="dance"/>
            <FieldRef field="dance_h"/>
          </Apply>
          <Constant dataType="double">3</Constant>
        </Apply>
        <FieldRef field="dance_t"/>
      </Apply>
      <Constant dataType="double">90</Constant>
    </Apply>
  </Apply>
  <Apply function="/">
    <Apply function="*">
      <Apply function="*">
        <Apply function="*">
          <FieldRef field="hexercises"/>
          <FieldRef field="hexercises_h"/>
        </Apply>
        <Constant dataType="double">3</Constant>
      </Apply>
      <FieldRef field="hexercises_t"/>
    </Apply>
    <Constant dataType="double">90</Constant>
  </Apply>
```

```
          </Apply>
          <Apply function="/">
            <Apply function="*">
              <Apply function="*">
                <Apply function="*">
                  <FieldRef field="hockey"/>
                  <FieldRef field="hockey_h"/>
                </Apply>
                <Constant dataType="double">6</Constant>
              </Apply>
              <FieldRef field="hockey_t"/>
            </Apply>
            <Constant dataType="double">90</Constant>
          </Apply>
        </Apply>
        <Apply function="/">
          <Apply function="*">
            <Apply function="*">
              <Apply function="*">
                <FieldRef field="skate"/>
                <FieldRef field="skate_h"/>
              </Apply>
              <Constant dataType="double">4</Constant>
            </Apply>
            <FieldRef field="skate_t"/>
          </Apply>
          <Constant dataType="double">90</Constant>
        </Apply>
      </Apply>
      <Apply function="/">
        <Apply function="*">
          <Apply function="*">
            <Apply function="*">
              <FieldRef field="inline"/>
              <FieldRef field="inline_h"/>
            </Apply>
            <Constant dataType="double">5</Constant>
          </Apply>
          <FieldRef field="inline_t"/>
        </Apply>
        <Constant dataType="double">90</Constant>
      </Apply>
    </Apply>
    <Apply function="/">
      <Apply function="*">
        <Apply function="*">
          <Apply function="*">
            <FieldRef field="jogrun"/>
            <FieldRef field="jogrun_h"/>
          </Apply>
          <Constant dataType="double">9.5</Constant>
        </Apply>
        <FieldRef field="jogrun_t"/>
      </Apply>
      <Constant dataType="double">90</Constant>
    </Apply>
  </Apply>
  <Apply function="/">
    <Apply function="*">
      <Apply function="*">
        <Apply function="*">
```

```
                <FieldRef field="golf"/>
                <FieldRef field="golf_h"/>
              </Apply>
              <Constant dataType="double">4</Constant>
            </Apply>
            <FieldRef field="golf_t"/>
          </Apply>
          <Constant dataType="double">90</Constant>
        </Apply>
      </Apply>
      <Apply function="/">
        <Apply function="*">
          <Apply function="*">
            <Apply function="*">
              <FieldRef field="aerobics"/>
              <FieldRef field="aerobics_h"/>
            </Apply>
            <Constant dataType="double">4</Constant>
          </Apply>
          <FieldRef field="aerobics_t"/>
        </Apply>
        <Constant dataType="double">90</Constant>
      </Apply>
    </Apply>
    <Apply function="/">
      <Apply function="*">
        <Apply function="*">
          <Apply function="*">
            <FieldRef field="ski"/>
            <FieldRef field="ski_h"/>
          </Apply>
          <Constant dataType="double">4</Constant>
        </Apply>
        <FieldRef field="ski_t"/>
      </Apply>
      <Constant dataType="double">90</Constant>
    </Apply>
  </Apply>
  <Apply function="/">
    <Apply function="*">
      <Apply function="*">
        <Apply function="*">
          <FieldRef field="bowl"/>
          <FieldRef field="bowl_h"/>
        </Apply>
        <Constant dataType="double">2</Constant>
      </Apply>
      <FieldRef field="bowl_t"/>
    </Apply>
    <Constant dataType="double">90</Constant>
  </Apply>
</Apply>
<Apply function="/">
  <Apply function="*">
    <Apply function="*">
      <Apply function="*">
        <FieldRef field="baseball"/>
        <FieldRef field="baseball_h"/>
      </Apply>
      <Constant dataType="double">3</Constant>
    </Apply>
```

```
            <FieldRef field="baseball_t"/>
          </Apply>
          <Constant dataType="double">90</Constant>
        </Apply>
      </Apply>
      <Apply function="/">
        <Apply function="*">
          <Apply function="*">
            <Apply function="*">
              <FieldRef field="tennis"/>
              <FieldRef field="tennis_h"/>
            </Apply>
            <Constant dataType="double">4</Constant>
          </Apply>
          <FieldRef field="tennis_t"/>
        </Apply>
        <Constant dataType="double">90</Constant>
      </Apply>
    </Apply>
    <Apply function="/">
      <Apply function="*">
        <Apply function="*">
          <Apply function="*">
            <FieldRef field="weights"/>
            <FieldRef field="weights_h"/>
          </Apply>
          <Constant dataType="double">3</Constant>
        </Apply>
        <FieldRef field="weights_t"/>
      </Apply>
      <Constant dataType="double">90</Constant>
    </Apply>
  </Apply>
  <Apply function="/">
    <Apply function="*">
      <Apply function="*">
        <Apply function="*">
          <FieldRef field="fishing"/>
          <FieldRef field="fishing_h"/>
        </Apply>
        <Constant dataType="double">3</Constant>
      </Apply>
      <FieldRef field="fishing_t"/>
    </Apply>
    <Constant dataType="double">90</Constant>
  </Apply>
</Apply>
<Apply function="/">
  <Apply function="*">
    <Apply function="*">
      <Apply function="*">
        <FieldRef field="volleyball"/>
        <FieldRef field="volleyball_h"/>
      </Apply>
      <Constant dataType="double">5</Constant>
    </Apply>
    <FieldRef field="volleyball_t"/>
  </Apply>
  <Constant dataType="double">90</Constant>
</Apply>
</Apply>
```

```
      <Apply function="/">
        <Apply function="*">
          <Apply function="*">
            <Apply function="*">
              <FieldRef field="basketball"/>
              <FieldRef field="basketball_h"/>
            </Apply>
            <Constant dataType="double">6</Constant>
          </Apply>
          <FieldRef field="basketball_t"/>
        </Apply>
        <Constant dataType="double">90</Constant>
      </Apply>
    </Apply>
    <Apply function="/">
      <Apply function="*">
        <Apply function="*">
          <Apply function="*">
            <FieldRef field="soccer"/>
            <FieldRef field="soccer_h"/>
          </Apply>
          <Constant dataType="double">5</Constant>
        </Apply>
        <FieldRef field="soccer_t"/>
      </Apply>
      <Constant dataType="double">90</Constant>
    </Apply>
  </Apply>
  <Apply function="/">
    <Apply function="*">
      <Apply function="*">
        <Apply function="*">
          <FieldRef field="other"/>
          <FieldRef field="other_h"/>
        </Apply>
        <Constant dataType="double">4</Constant>
      </Apply>
      <FieldRef field="other_t"/>
    </Apply>
    <Constant dataType="double">90</Constant>
  </Apply>
</Apply>
 </DerivedField>
 <DerivedField name="PhysicalActivityraw2" dataType="double" optype="continuous">
<Apply function="if">
  <Apply function="greaterThan">
    <FieldRef field="PhysicalActivityraw1"/>
    <Constant dataType="double">10</Constant>
  </Apply>
  <Constant dataType="double">10</Constant>
  <FieldRef field="PhysicalActivityraw1"/>
</Apply>
 </DerivedField>
 <DerivedField name="PhysicalActivity_cont" dataType="double" optype="continuous">
<Apply function="ln">
  <FieldRef field="PhysicalActivityraw2"/>
</Apply>
 </DerivedField>
 <DerivedField name="weeklyalc" dataType="double" optype="continuous">
<Apply function="if">
  <Apply function="equal">
```

```
    <FieldRef field="dev"/>
    <Constant dataType="string">dev2</Constant>
  </Apply>
  <FieldRef field="NA"/>
  <Apply function="if">
    <Apply function="and">
      <Apply function="equal">
        <FieldRef field="dev"/>
        <Constant dataType="string">dev1</Constant>
      </Apply>
      <Apply function="equal">
        <FieldRef field="dany"/>
        <Constant dataType="string">dany1</Constant>
      </Apply>
    </Apply>
    <Apply function="+">
      <Apply function="+">
        <Apply function="+">
          <Apply function="+">
            <Apply function="+">
              <Apply function="+">
                <FieldRef field="drk_drkm"/>
                <FieldRef field="drk_drkt"/>
              </Apply>
              <FieldRef field="drk_drkw"/>
            </Apply>
            <FieldRef field="drk_drkr"/>
          </Apply>
          <FieldRef field="drk_drkf"/>
        </Apply>
        <FieldRef field="drk_drksa"/>
      </Apply>
      <FieldRef field="drk_drksu"/>
    </Apply>
    <Constant dataType="double">0</Constant>
  </Apply>
</Apply>
 </DerivedField>
 <DerivedField name="bingeflag" dataType="double" optype="continuous">
<Apply function="if">
  <Apply function="equal">
    <FieldRef field="dev"/>
    <Constant dataType="string">dev2</Constant>
  </Apply>
  <FieldRef field="NA"/>
  <Apply function="if">
    <Apply function="and">
      <Apply function="not">
        <Apply function="is.na">
          <FieldRef field="db"/>
        </Apply>
      </Apply>
      <Apply function="or">
        <Apply function="equal">
          <FieldRef field="db"/>
          <Constant dataType="string">db5</Constant>
        </Apply>
        <Apply function="equal">
          <FieldRef field="db"/>
          <Constant dataType="string">db6</Constant>
        </Apply>
```

```
    </Apply>
  </Apply>
  <Constant dataType="double">1</Constant>
  <Apply function="if">
    <Apply function="and">
      <Apply function="not">
        <Apply function="is.na">
          <FieldRef field="drk_drkm"/>
        </Apply>
      </Apply>
      <Apply function="greaterOrEqual">
        <FieldRef field="drk_drkm"/>
        <Constant dataType="double">5</Constant>
      </Apply>
    </Apply>
    <Constant dataType="double">1</Constant>
    <Apply function="if">
      <Apply function="and">
        <Apply function="not">
          <Apply function="is.na">
            <FieldRef field="drk_drkt"/>
          </Apply>
        </Apply>
        <FieldRef field="drk_drkt"/>
      </Apply>
      <Constant dataType="double">1</Constant>
      <Apply function="if">
        <Apply function="and">
          <Apply function="not">
            <Apply function="is.na">
              <FieldRef field="drk_drkw"/>
            </Apply>
          </Apply>
          <Apply function="greaterOrEqual">
            <FieldRef field="drk_drkw"/>
            <Constant dataType="double">5</Constant>
          </Apply>
        </Apply>
        <Constant dataType="double">1</Constant>
        <Apply function="if">
          <Apply function="and">
            <Apply function="not">
              <Apply function="is.na">
                <FieldRef field="drk_drkr"/>
              </Apply>
            </Apply>
            <Apply function="greaterOrEqual">
              <FieldRef field="drk_drkr"/>
              <Constant dataType="double">5</Constant>
            </Apply>
          </Apply>
          <Constant dataType="double">1</Constant>
          <Apply function="if">
            <Apply function="and">
              <Apply function="not">
                <Apply function="is.na">
                  <FieldRef field="drk_drkf"/>
                </Apply>
              </Apply>
              <Apply function="greaterOrEqual">
                <FieldRef field="drk_drkf"/>
```

```
                  <Constant dataType="double">5</Constant>
                </Apply>
              </Apply>
              <Constant dataType="double">1</Constant>
              <Apply function="if">
                <Apply function="and">
                  <Apply function="not">
                    <Apply function="is.na">
                      <FieldRef field="drk_drksa"/>
                    </Apply>
                  </Apply>
                  <Apply function="greaterOrEqual">
                    <FieldRef field="drk_drksa"/>
                    <Constant dataType="double">5</Constant>
                  </Apply>
                </Apply>
                <Constant dataType="double">1</Constant>
                <Apply function="if">
                  <Apply function="and">
                    <Apply function="not">
                      <Apply function="is.na">
                        <FieldRef field="drk_drksu"/>
                      </Apply>
                    </Apply>
                    <Apply function="greaterOrEqual">
                      <FieldRef field="drk_drksu"/>
                      <Constant dataType="double">5</Constant>
                    </Apply>
                  </Apply>
                  <Constant dataType="double">1</Constant>
                  <FieldRef field="NA"/>
                </Apply>
              </Apply>
            </Apply>
          </Apply>
        </Apply>
      </Apply>
    </Apply>
  </Apply>
</Apply>
 </DerivedField>
 <DerivedField name="AlcoholMod_cat" dataType="double" optype="continuous">
<Apply function="if">
  <Apply function="and">
    <Apply function="not">
      <Apply function="is.na">
        <FieldRef field="bingeflag"/>
      </Apply>
    </Apply>
    <Apply function="equal">
      <FieldRef field="bingeflag"/>
      <Constant dataType="double">1</Constant>
    </Apply>
  </Apply>
  <Constant dataType="double">0</Constant>
  <Apply function="if">
    <Apply function="and">
      <Apply function="and">
        <Apply function="and">
          <Apply function="and">
            <Apply function="equal">
```

```
          <FieldRef field="Sex"/>
          <Constant dataType="double">0</Constant>
        </Apply>
        <Apply function="equal">
          <FieldRef field="dev"/>
          <Constant dataType="string">dev1</Constant>
        </Apply>
      </Apply>
      <Apply function="equal">
        <FieldRef field="dany"/>
        <Constant dataType="string">dany1</Constant>
      </Apply>
    </Apply>
    <Apply function="greaterThan">
      <FieldRef field="weeklyalc"/>
      <Constant dataType="double">3</Constant>
    </Apply>
  </Apply>
  <Apply function="lessOrEqual">
    <FieldRef field="weeklyalc"/>
    <Constant dataType="double">21</Constant>
  </Apply>
</Apply>
<Constant dataType="double">1</Constant>
<Apply function="if">
  <Apply function="and">
    <Apply function="and">
      <Apply function="and">
        <Apply function="and">
          <Apply function="equal">
            <FieldRef field="Sex"/>
            <Constant dataType="double">1</Constant>
          </Apply>
          <Apply function="equal">
            <FieldRef field="dev"/>
            <Constant dataType="string">dev1</Constant>
          </Apply>
        </Apply>
        <Apply function="equal">
          <FieldRef field="dany"/>
          <Constant dataType="string">dany1</Constant>
        </Apply>
      </Apply>
      <Apply function="greaterThan">
        <FieldRef field="weeklyalc"/>
        <Constant dataType="double">2</Constant>
      </Apply>
    </Apply>
    <Apply function="lessOrEqual">
      <FieldRef field="weeklyalc"/>
      <Constant dataType="double">14</Constant>
    </Apply>
  </Apply>
  <Constant dataType="double">1</Constant>
  <Apply function="if">
    <Apply function="and">
      <Apply function="and">
        <Apply function="and">
          <Apply function="equal">
            <FieldRef field="Sex"/>
            <Constant dataType="double">0</Constant>
```

```
        </Apply>
        <Apply function="equal">
          <FieldRef field="dev"/>
          <Constant dataType="string">dev1</Constant>
        </Apply>
      </Apply>
      <Apply function="equal">
        <FieldRef field="dany"/>
        <Constant dataType="string">dany1</Constant>
      </Apply>
    </Apply>
    <Apply function="lessOrEqual">
      <FieldRef field="weeklyalc"/>
      <Constant dataType="double">3</Constant>
    </Apply>
  </Apply>
  <Constant dataType="double">0</Constant>
  <Apply function="if">
    <Apply function="and">
      <Apply function="and">
        <Apply function="and">
          <Apply function="equal">
            <FieldRef field="Sex"/>
            <Constant dataType="double">1</Constant>
          </Apply>
          <Apply function="equal">
            <FieldRef field="dev"/>
            <Constant dataType="string">dev1</Constant>
          </Apply>
        </Apply>
        <Apply function="equal">
          <FieldRef field="dany"/>
          <Constant dataType="string">dany1</Constant>
        </Apply>
      </Apply>
      <Apply function="lessOrEqual">
        <FieldRef field="weeklyalc"/>
        <Constant dataType="double">2</Constant>
      </Apply>
    </Apply>
    <Constant dataType="double">0</Constant>
    <Apply function="if">
      <Apply function="and">
        <Apply function="and">
          <Apply function="and">
            <Apply function="equal">
              <FieldRef field="Sex"/>
              <Constant dataType="double">0</Constant>
            </Apply>
            <Apply function="equal">
              <FieldRef field="dev"/>
              <Constant dataType="string">dev1</Constant>
            </Apply>
          </Apply>
          <Apply function="equal">
            <FieldRef field="dany"/>
            <Constant dataType="string">dany1</Constant>
          </Apply>
        </Apply>
        <Apply function="greaterThan">
          <FieldRef field="weeklyalc"/>
```

```
                <Constant dataType="double">21</Constant>
              </Apply>
            </Apply>
            <Constant dataType="double">0</Constant>
            <Apply function="if">
              <Apply function="and">
                <Apply function="and">
                  <Apply function="and">
                    <Apply function="equal">
                      <FieldRef field="Sex"/>
                      <Constant dataType="double">1</Constant>
                    </Apply>
                    <Apply function="equal">
                      <FieldRef field="dev"/>
                      <Constant dataType="string">dev1</Constant>
                    </Apply>
                  </Apply>
                  <Apply function="equal">
                    <FieldRef field="dany"/>
                    <Constant dataType="string">dany1</Constant>
                  </Apply>
                </Apply>
                <Apply function="greaterThan">
                  <FieldRef field="weeklyalc"/>
                  <Constant dataType="double">14</Constant>
                </Apply>
              </Apply>
              <Constant dataType="double">0</Constant>
              <Apply function="if">
                <Apply function="or">
                  <Apply function="equal">
                    <FieldRef field="dev"/>
                    <Constant dataType="string">dev2</Constant>
                  </Apply>
                  <Apply function="equal">
                    <FieldRef field="dany"/>
                    <Constant dataType="string">dany2</Constant>
                  </Apply>
                </Apply>
                <Constant dataType="double">0</Constant>
                <FieldRef field="NA"/>
              </Apply>
            </Apply>
          </Apply>
        </Apply>
      </Apply>
    </Apply>
  </Apply>
</Apply>
 </DerivedField>
 <DerivedField name="AlcoholHeavy_cat" dataType="double" optype="continuous">
<Apply function="if">
  <Apply function="or">
    <Apply function="and">
      <Apply function="not">
        <Apply function="is.na">
          <FieldRef field="bingeflag"/>
        </Apply>
      </Apply>
      <Apply function="equal">
        <FieldRef field="bingeflag"/>
```

```
        <Constant dataType="double">1</Constant>
      </Apply>
    </Apply>
    <Apply function="and">
      <Apply function="and">
        <Apply function="and">
          <Apply function="equal">
            <FieldRef field="Sex"/>
            <Constant dataType="double">0</Constant>
          </Apply>
          <Apply function="equal">
            <FieldRef field="dev"/>
            <Constant dataType="string">dev1</Constant>
          </Apply>
        </Apply>
        <Apply function="equal">
          <FieldRef field="dany"/>
          <Constant dataType="string">dany1</Constant>
        </Apply>
      </Apply>
      <Apply function="greaterThan">
        <FieldRef field="weeklyalc"/>
        <Constant dataType="double">21</Constant>
      </Apply>
    </Apply>
  </Apply>
  <Constant dataType="double">1</Constant>
  <Apply function="if">
    <Apply function="and">
      <Apply function="and">
        <Apply function="and">
          <Apply function="equal">
            <FieldRef field="Sex"/>
            <Constant dataType="double">1</Constant>
          </Apply>
          <Apply function="equal">
            <FieldRef field="dev"/>
            <Constant dataType="string">dev1</Constant>
          </Apply>
        </Apply>
        <Apply function="equal">
          <FieldRef field="dany"/>
          <Constant dataType="string">dany1</Constant>
        </Apply>
      </Apply>
      <Apply function="greaterThan">
        <FieldRef field="weeklyalc"/>
        <Constant dataType="double">14</Constant>
      </Apply>
    </Apply>
    <Constant dataType="double">1</Constant>
    <Apply function="if">
      <Apply function="and">
        <Apply function="and">
          <Apply function="and">
            <Apply function="equal">
              <FieldRef field="Sex"/>
              <Constant dataType="double">0</Constant>
            </Apply>
            <Apply function="equal">
              <FieldRef field="dev"/>
```

```
              <Constant dataType="string">dev1</Constant>
            </Apply>
          </Apply>
          <Apply function="equal">
            <FieldRef field="dany"/>
            <Constant dataType="string">dany1</Constant>
          </Apply>
        </Apply>
        <Apply function="lessOrEqual">
          <FieldRef field="weeklyalc"/>
          <Constant dataType="double">21</Constant>
        </Apply>
      </Apply>
      <Constant dataType="double">0</Constant>
      <Apply function="if">
        <Apply function="and">
          <Apply function="and">
            <Apply function="and">
              <Apply function="equal">
                <FieldRef field="Sex"/>
                <Constant dataType="double">1</Constant>
              </Apply>
              <Apply function="equal">
                <FieldRef field="dev"/>
                <Constant dataType="string">dev1</Constant>
              </Apply>
            </Apply>
            <Apply function="equal">
              <FieldRef field="dany"/>
              <Constant dataType="string">dany1</Constant>
            </Apply>
          </Apply>
          <Apply function="lessOrEqual">
            <FieldRef field="weeklyalc"/>
            <Constant dataType="double">14</Constant>
          </Apply>
        </Apply>
        <Constant dataType="double">0</Constant>
        <Apply function="if">
          <Apply function="or">
            <Apply function="equal">
              <FieldRef field="dev"/>
              <Constant dataType="string">dev2</Constant>
            </Apply>
            <Apply function="equal">
              <FieldRef field="dany"/>
              <Constant dataType="string">dany2</Constant>
            </Apply>
          </Apply>
          <Constant dataType="double">0</Constant>
          <FieldRef field="NA"/>
        </Apply>
      </Apply>
    </Apply>
  </Apply>
</Apply>
 </DerivedField>
 <DerivedField name="djuice" dataType="double" optype="continuous">
<Apply function="if">
  <Apply function="equal">
    <FieldRef field="juiu"/>
```

```
    <Constant dataType="string">juid</Constant>
  </Apply>
  <FieldRef field="jui"/>
  <Apply function="if">
    <Apply function="equal">
      <FieldRef field="juiu"/>
      <Constant dataType="string">juiw</Constant>
    </Apply>
    <Apply function="/">
      <FieldRef field="jui"/>
      <Constant dataType="double">7</Constant>
    </Apply>
    <Apply function="if">
      <Apply function="equal">
        <FieldRef field="juiu"/>
        <Constant dataType="string">juim</Constant>
      </Apply>
      <Apply function="/">
        <FieldRef field="jui"/>
        <Constant dataType="double">30</Constant>
      </Apply>
      <Apply function="if">
        <Apply function="equal">
          <FieldRef field="juiu"/>
          <Constant dataType="string">juiy</Constant>
        </Apply>
        <Apply function="/">
          <FieldRef field="jui"/>
          <Constant dataType="double">365</Constant>
        </Apply>
        <Constant dataType="double">0</Constant>
      </Apply>
    </Apply>
  </Apply>
</Apply>
 </DerivedField>
 <DerivedField name="dfruit" dataType="double" optype="continuous">
<Apply function="if">
  <Apply function="equal">
    <FieldRef field="frtu"/>
    <Constant dataType="string">frtd</Constant>
  </Apply>
  <FieldRef field="frt"/>
  <Apply function="if">
    <Apply function="equal">
      <FieldRef field="frtu"/>
      <Constant dataType="string">frtw</Constant>
    </Apply>
    <Apply function="/">
      <FieldRef field="frt"/>
      <Constant dataType="double">7</Constant>
    </Apply>
    <Apply function="if">
      <Apply function="equal">
        <FieldRef field="frtu"/>
        <Constant dataType="string">frtm</Constant>
      </Apply>
      <Apply function="/">
        <FieldRef field="frt"/>
        <Constant dataType="double">30</Constant>
      </Apply>
```

```
      <Apply function="if">
        <Apply function="equal">
          <FieldRef field="frtu"/>
          <Constant dataType="string">frty</Constant>
        </Apply>
        <Apply function="/">
          <FieldRef field="frt"/>
          <Constant dataType="double">365</Constant>
        </Apply>
        <Constant dataType="double">0</Constant>
      </Apply>
    </Apply>
  </Apply>
</Apply>
 </DerivedField>
 <DerivedField name="dsalad" dataType="double" optype="continuous">
<Apply function="if">
  <Apply function="equal">
    <FieldRef field="salu"/>
    <Constant dataType="string">sald</Constant>
  </Apply>
  <FieldRef field="sal"/>
  <Apply function="if">
    <Apply function="equal">
      <FieldRef field="salu"/>
      <Constant dataType="string">salw</Constant>
    </Apply>
    <Apply function="/">
      <FieldRef field="sal"/>
      <Constant dataType="double">7</Constant>
    </Apply>
    <Apply function="if">
      <Apply function="equal">
        <FieldRef field="salu"/>
        <Constant dataType="string">salm</Constant>
      </Apply>
      <Apply function="/">
        <FieldRef field="sal"/>
        <Constant dataType="double">30</Constant>
      </Apply>
      <Apply function="if">
        <Apply function="equal">
          <FieldRef field="salu"/>
          <Constant dataType="string">saly</Constant>
        </Apply>
        <Apply function="/">
          <FieldRef field="sal"/>
          <Constant dataType="double">365</Constant>
        </Apply>
        <Constant dataType="double">0</Constant>
      </Apply>
    </Apply>
  </Apply>
</Apply>
 </DerivedField>
 <DerivedField name="dpotato" dataType="double" optype="continuous">
<Apply function="if">
  <Apply function="equal">
    <FieldRef field="potu"/>
    <Constant dataType="string">potd</Constant>
  </Apply>
```

```
  <FieldRef field="pot"/>
  <Apply function="if">
    <Apply function="equal">
      <FieldRef field="potu"/>
      <Constant dataType="string">potw</Constant>
    </Apply>
    <Apply function="/">
      <FieldRef field="pot"/>
      <Constant dataType="double">7</Constant>
    </Apply>
    <Apply function="if">
      <Apply function="equal">
        <FieldRef field="potu"/>
        <Constant dataType="string">potm</Constant>
      </Apply>
      <Apply function="/">
        <FieldRef field="pot"/>
        <Constant dataType="double">30</Constant>
      </Apply>
      <Apply function="if">
        <Apply function="equal">
          <FieldRef field="potu"/>
          <Constant dataType="string">poty</Constant>
        </Apply>
        <Apply function="/">
          <FieldRef field="pot"/>
          <Constant dataType="double">365</Constant>
        </Apply>
        <Constant dataType="double">0</Constant>
      </Apply>
    </Apply>
  </Apply>
</Apply>
 </DerivedField>
 <DerivedField name="dcarrot" dataType="double" optype="continuous">
<Apply function="if">
  <Apply function="equal">
    <FieldRef field="caru"/>
    <Constant dataType="string">card</Constant>
  </Apply>
  <FieldRef field="car"/>
  <Apply function="if">
    <Apply function="equal">
      <FieldRef field="caru"/>
      <Constant dataType="string">carw</Constant>
    </Apply>
    <Apply function="/">
      <FieldRef field="car"/>
      <Constant dataType="double">7</Constant>
    </Apply>
    <Apply function="if">
      <Apply function="equal">
        <FieldRef field="caru"/>
        <Constant dataType="string">carm</Constant>
      </Apply>
      <Apply function="/">
        <FieldRef field="car"/>
        <Constant dataType="double">30</Constant>
      </Apply>
      <Apply function="if">
        <Apply function="equal">
```

```
          <FieldRef field="caru"/>
          <Constant dataType="string">cary</Constant>
        </Apply>
        <Apply function="/">
          <FieldRef field="car"/>
          <Constant dataType="double">365</Constant>
        </Apply>
        <Constant dataType="double">0</Constant>
      </Apply>
    </Apply>
  </Apply>
</Apply>
 </DerivedField>
 <DerivedField name="dveg" dataType="double" optype="continuous">
<Apply function="if">
  <Apply function="equal">
    <FieldRef field="vegu"/>
    <Constant dataType="string">vegd</Constant>
  </Apply>
  <FieldRef field="veg"/>
  <Apply function="if">
    <Apply function="equal">
      <FieldRef field="vegu"/>
      <Constant dataType="string">vegw</Constant>
    </Apply>
    <Apply function="/">
      <FieldRef field="veg"/>
      <Constant dataType="double">7</Constant>
    </Apply>
    <Apply function="if">
      <Apply function="equal">
        <FieldRef field="vegu"/>
        <Constant dataType="string">vegm</Constant>
      </Apply>
      <Apply function="/">
        <FieldRef field="veg"/>
        <Constant dataType="double">30</Constant>
      </Apply>
      <Apply function="if">
        <Apply function="equal">
          <FieldRef field="vegu"/>
          <Constant dataType="string">vegy</Constant>
        </Apply>
        <Apply function="/">
          <FieldRef field="veg"/>
          <Constant dataType="double">365</Constant>
        </Apply>
        <Constant dataType="double">0</Constant>
      </Apply>
    </Apply>
  </Apply>
</Apply>
 </DerivedField>
 <DerivedField name="fruitnvegraw" dataType="double" optype="continuous">
<Apply function="+">
  <Apply function="+">
    <Apply function="+">
      <Apply function="+">
        <FieldRef field="dfruit"/>
        <FieldRef field="dsalad"/>
      </Apply>
```

```
      <FieldRef field="dpotato"/>
    </Apply>
    <FieldRef field="dcarrot"/>
  </Apply>
  <FieldRef field="dveg"/>
</Apply>
 </DerivedField>
 <DerivedField name="fruitnveg" dataType="double" optype="continuous">
<Apply function="if">
  <Apply function="and">
    <Apply function="greaterThan">
      <FieldRef field="fruitnvegraw"/>
      <Constant dataType="double">8</Constant>
    </Apply>
    <Apply function="lessThan">
      <FieldRef field="fruitnvegraw"/>
      <Constant dataType="double">98</Constant>
    </Apply>
  </Apply>
  <Constant dataType="double">8</Constant>
  <FieldRef field="fruitnvegraw"/>
</Apply>
 </DerivedField>
 <DerivedField name="nocarrotflag" dataType="double" optype="continuous">
<Apply function="if">
  <Apply function="equal">
    <Apply function="*">
      <FieldRef field="dcarrot"/>
      <Constant dataType="double">7</Constant>
    </Apply>
    <Constant dataType="double">0</Constant>
  </Apply>
  <Constant dataType="double">1</Constant>
  <Apply function="if">
    <Apply function="greaterOrEqual">
      <Apply function="*">
        <FieldRef field="dcarrot"/>
        <Constant dataType="double">7</Constant>
      </Apply>
      <Constant dataType="double">1</Constant>
    </Apply>
    <Constant dataType="double">0</Constant>
    <Constant dataType="double">0</Constant>
  </Apply>
</Apply>
 </DerivedField>
 <DerivedField name="highpotatoflag" dataType="double" optype="continuous">
<Apply function="if">
  <Apply function="and">
    <Apply function="equal">
      <FieldRef field="Sex"/>
      <Constant dataType="double">0</Constant>
    </Apply>
    <Apply function="greaterOrEqual">
      <Apply function="*">
        <FieldRef field="dpotato"/>
        <Constant dataType="double">7</Constant>
      </Apply>
      <Constant dataType="double">7</Constant>
    </Apply>
  </Apply>
```

```
  <Constant dataType="double">1</Constant>
  <Apply function="if">
    <Apply function="and">
      <Apply function="equal">
        <FieldRef field="Sex"/>
        <Constant dataType="double">0</Constant>
      </Apply>
      <Apply function="greaterOrEqual">
        <Apply function="*">
          <FieldRef field="dpotato"/>
          <Constant dataType="double">7</Constant>
        </Apply>
        <Constant dataType="double">5</Constant>
      </Apply>
    </Apply>
    <Constant dataType="double">1</Constant>
    <Constant dataType="double">0</Constant>
  </Apply>
</Apply>
 </DerivedField>
 <DerivedField name="highjuice" dataType="double" optype="continuous">
<Apply function="if">
  <Apply function="equal">
    <FieldRef field="djuice"/>
    <Constant dataType="double">2</Constant>
  </Apply>
  <Constant dataType="double">1</Constant>
  <Apply function="if">
    <Apply function="equal">
      <FieldRef field="djuice"/>
      <Constant dataType="double">3</Constant>
    </Apply>
    <Constant dataType="double">2</Constant>
    <Apply function="if">
      <Apply function="equal">
        <FieldRef field="djuice"/>
        <Constant dataType="double">4</Constant>
      </Apply>
      <Constant dataType="double">3</Constant>
      <Apply function="if">
        <Apply function="equal">
          <FieldRef field="djuice"/>
          <Constant dataType="double">5</Constant>
        </Apply>
        <Constant dataType="double">4</Constant>
        <Apply function="if">
          <Apply function="equal">
            <FieldRef field="djuice"/>
            <Constant dataType="double">5</Constant>
          </Apply>
          <Constant dataType="double">4</Constant>
          <Apply function="if">
            <Apply function="equal">
              <FieldRef field="djuice"/>
              <Constant dataType="double">6</Constant>
            </Apply>
            <Constant dataType="double">5</Constant>
            <Apply function="if">
              <Apply function="equal">
                <FieldRef field="djuice"/>
                <Constant dataType="double">1</Constant>
```

```
              </Apply>
              <Constant dataType="double">0</Constant>
              <Apply function="if">
                <Apply function="equal">
                  <FieldRef field="djuice"/>
                  <Constant dataType="double">0</Constant>
                </Apply>
                <Constant dataType="double">0</Constant>
                <Constant dataType="double">0</Constant>
              </Apply>
            </Apply>
          </Apply>
        </Apply>
      </Apply>
    </Apply>
  </Apply>
</Apply>
 </DerivedField>
 <DerivedField name="dietraw" dataType="double" optype="continuous">
<Apply function="-">
  <Apply function="-">
    <Apply function="-">
      <FieldRef field="fruitnveg"/>
      <Apply function="*">
        <Constant dataType="double">2</Constant>
        <FieldRef field="highpotatoflag"/>
      </Apply>
    </Apply>
    <Apply function="*">
      <Constant dataType="double">2</Constant>
      <FieldRef field="nocarrotflag"/>
    </Apply>
  </Apply>
  <Apply function="*">
    <Constant dataType="double">2</Constant>
    <FieldRef field="highjuice"/>
  </Apply>
</Apply>
 </DerivedField>
 <DerivedField name="DietScore_cont" dataType="double" optype="continuous">
<Apply function="if">
  <Apply function="lessThan">
    <FieldRef field="dietraw"/>
    <Constant dataType="double">0</Constant>
  </Apply>
  <Constant dataType="double">0</Constant>
  <Apply function="if">
    <Apply function="greaterThan">
      <FieldRef field="dietraw"/>
      <Constant dataType="double">10</Constant>
    </Apply>
    <Constant dataType="double">10</Constant>
    <FieldRef field="dietraw"/>
  </Apply>
</Apply>
 </DerivedField>
 <DerivedField name="HeartDis_cat" dataType="double" optype="continuous">
<Apply function="if">
  <Apply function="equal">
    <FieldRef field="hd"/>
    <Constant dataType="string">hd1</Constant>
```

```
  </Apply>
  <Constant dataType="double">1</Constant>
  <Apply function="if">
    <Apply function="equal">
      <FieldRef field="hd"/>
      <Constant dataType="string">hd2</Constant>
    </Apply>
    <Constant dataType="double">0</Constant>
    <FieldRef field="NA"/>
  </Apply>
</Apply>
 </DerivedField>
 <DerivedField name="Diabetes_cat" dataType="double" optype="continuous">
<Apply function="if">
  <Apply function="equal">
    <FieldRef field="diab"/>
    <Constant dataType="string">diab1</Constant>
  </Apply>
  <Constant dataType="double">1</Constant>
  <Apply function="if">
    <Apply function="equal">
      <FieldRef field="diab"/>
      <Constant dataType="string">diab2</Constant>
    </Apply>
    <Constant dataType="double">0</Constant>
    <FieldRef field="NA"/>
  </Apply>
</Apply>
 </DerivedField>
 <DerivedField name="Stroke_cat" dataType="double" optype="continuous">
<Apply function="if">
  <Apply function="equal">
    <FieldRef field="stk"/>
    <Constant dataType="string">stk1</Constant>
  </Apply>
  <Constant dataType="double">1</Constant>
  <Apply function="if">
    <Apply function="equal">
      <FieldRef field="stk"/>
      <Constant dataType="string">stk2</Constant>
    </Apply>
    <Constant dataType="double">0</Constant>
    <FieldRef field="NA"/>
  </Apply>
</Apply>
 </DerivedField>
 <DerivedField name="Cancer_cat" dataType="double" optype="continuous">
<Apply function="if">
  <Apply function="equal">
    <FieldRef field="can"/>
    <Constant dataType="string">can1</Constant>
  </Apply>
  <Constant dataType="double">1</Constant>
  <Apply function="if">
    <Apply function="equal">
      <FieldRef field="can"/>
      <Constant dataType="string">can2</Constant>
    </Apply>
    <Constant dataType="double">0</Constant>
    <FieldRef field="NA"/>
  </Apply>
```

```
</Apply>
 </DerivedField>
 <DerivedField name="DiabetesAge_int" dataType="double" optype="continuous">
<Apply function="*">
  <FieldRef field="Diabetes_cat"/>
  <FieldRef field="Age_cont"/>
</Apply>
 </DerivedField>
 <DerivedField name="CancerAge_int" dataType="double" optype="continuous">
<Apply function="*">
  <FieldRef field="Cancer_cat"/>
  <FieldRef field="Age_cont"/>
</Apply>
 </DerivedField>
 <DerivedField name="weightkg" dataType="double" optype="continuous">
<Apply function="/">
  <FieldRef field="weightlb"/>
  <Constant dataType="double">2.2046226218</Constant>
</Apply>
 </DerivedField>
 <DerivedField name="heightm" dataType="double" optype="continuous">
<Apply function="/">
  <Apply function="+">
    <Apply function="*">
      <FieldRef field="heightin_hft"/>
      <Constant dataType="double">12</Constant>
    </Apply>
    <FieldRef field="heightin_hin"/>
  </Apply>
  <Constant dataType="double">39.3701</Constant>
</Apply>
 </DerivedField>
 <DerivedField name="BMI_spline" dataType="double" optype="continuous">
<Apply function="if">
  <Apply function="lessOrEqual">
    <Apply function="-">
      <Apply function="/">
        <FieldRef field="weightkg"/>
        <Apply function="*">
          <FieldRef field="heightm"/>
          <FieldRef field="heightm"/>
        </Apply>
      </Apply>
      <Constant dataType="double">35</Constant>
    </Apply>
    <Constant dataType="double">0</Constant>
  </Apply>
  <Constant dataType="double">0</Constant>
  <Apply function="if">
    <Apply function="greaterThan">
      <Apply function="-">
        <Apply function="/">
          <FieldRef field="weightkg"/>
          <Apply function="*">
            <FieldRef field="heightm"/>
            <FieldRef field="heightm"/>
          </Apply>
        </Apply>
        <Constant dataType="double">35</Constant>
      </Apply>
      <Constant dataType="double">0</Constant>
```

```
    </Apply>
    <Apply function="-">
      <Apply function="/">
        <FieldRef field="weightkg"/>
        <Apply function="*">
          <FieldRef field="heightm"/>
          <FieldRef field="heightm"/>
        </Apply>
      </Apply>
      <Constant dataType="double">35</Constant>
    </Apply>
  </Apply>
</Apply>
 </DerivedField>
</LocalTransformations>
```
