# Supplementary material for: Measuring Burden of Unhealthy Behaviours Using a Multivariable Predictive Approach: Life Expectancy Lost in Canada Attributable to Smoking, Alcohol, Physical Inactivity, and Diet
Source: PLoS Med. 2016 Aug 16;13(8):e1002082. doi: 10.1371/journal.pmed.1002082 (PMC4986987; doi:10.1371/journal.pmed.1002082)
Supplement: S4 Text — (PDF) [file pmed.1002082.s018.pdf]

S4 Text. STROBE Statement—checklist of items that should be included in reports of observational studies

	Item No	Recommendation	
**Title and abstract**	1	(*a*) Indicate the study's design with a commonly used term in the title or the abstract	Included title and abstract
		(*b*) Provide in the abstract an informative and balanced summary of what was done and what was found	Included
**Introduction**			
Background/ rationale	2	Explain the scientific background and rationale for the investigation being reported	Included paragraphs 1-4
Objectives	3	State specific objectives, including any prespecified hypotheses	Included paragraph 5
**Methods**			
Study design	4	Present key elements of study design early in the paper	Included *Study Data* paragraph 1
Setting	5	Describe the setting, locations, and relevant dates, including periods of recruitment, exposure, follow-up, and data collection	Included *Derivation Cohort* and *Validation Cohorts*
Participants	6	(*a*) *Cohort study*—Give the eligibility criteria, and the sources and methods of selection of participants. Describe methods of follow-up<br>*Case-control study*—Give the eligibility criteria, and the sources and methods of case ascertainment and control selection. Give the rationale for the choice of cases and controls<br>*Cross-sectional study*—Give the eligibility criteria, and the sources and methods of selection of participants	Included *Study Data* paragraphs 2-3
		(*b*) *Cohort study*—For matched studies, give matching criteria and number of exposed and unexposed<br>*Case-control study*—For matched studies, give matching criteria and the number of controls per case	n/a
Variables	7	Clearly define all outcomes, exposures, predictors, potential confounders, and effect modifiers. Give diagnostic criteria, if applicable	Included *Risk factors for death*
Data sources/ measurement	8*	For each variable of interest, give sources of data and details of methods of assessment (measurement). Describe comparability of assessment methods if there is more than one group	Included *S1 Table* and *S3 Text*
Bias	9	Describe any efforts to address potential sources of bias	Included *Table 4* and *Limitations* paragraph 2
Study size	10	Explain how the study size was arrived at	All Ontario respondents of the linked Canadian Community Health Survey who met eligibility requirements were included. There were approximately 1 million person-years of follow-up and 9,900 deaths in the development and validation datasets. We calculated degrees of freedom (df) as per recommendation of Harrell: 1 df per 10 events.

Quantitative variables	11	Explain how quantitative variables were handled in the analyses. If applicable, describe which groupings were chosen and why	Included *Risk factors for death* paragraphs 2-4 and *Development of MPoRT algorithm* paragraphs 2-3
Statistical methods	12	(*a*) Describe all statistical methods, including those used to control for confounding	Included *Development of MPoRT algorithm*
		(*b*) Describe any methods used to examine subgroups and interactions	Included *Development of MPoRT algorithm* paragraph 3-4
		(*c*) Explain how missing data were addressed	Included *Development of MPoRT algorithm* paragraph 4
		(*d*) *Cohort study*—If applicable, explain how loss to follow-up was addressed<br>*Case-control study*—If applicable, explain how matching of cases and controls was addressed<br>*Cross-sectional study*—If applicable, describe analytical methods taking account of sampling strategy	n/a
		(*e*) Describe any sensitivity analyses	Included *Development of MPoRT algorithm* paragraph 3, *Validation of predictive accuracy and assessment of risk hazards*
**Results**			
Participants	13*	(a) Report numbers of individuals at each stage of study—eg numbers potentially eligible, examined for eligibility, confirmed eligible, included in the study, completing follow-up, and analysed	Included *S1 Fig*
		(b) Give reasons for non-participation at each stage	Included *S1 Fig*
		(c) Consider use of a flow diagram	Included *S1 Fig*
Descriptive data	14*	(a) Give characteristics of study participants (eg demographic, clinical, social) and information on exposures and potential confounders	Included *Table 2* and *Table 3*
		(b) Indicate number of participants with missing data for each variable of interest	Included *Table 4, S2 Table, S3 Table*, and *S4 Table*
		(c) *Cohort study*—Summarise follow-up time (eg, average and total amount)	Included *Table 4, S2 Table, S3 Table*, and *S4 Table*
Outcome data	15*	*Cohort study*—Report numbers of outcome events or summary measures over time	Included *Table 4, S2 Table, S3 Table*, and *S4 Table*
		Case-control study—Report numbers in each exposure category, or summary measures of exposure	n/a
		Cross-sectional study—Report numbers of outcome events or summary measures	n/a
Main results	16	(*a*) Give unadjusted estimates and, if applicable, confounder-adjusted estimates and their precision (eg, 95% confidence interval). Make clear which confounders were adjusted for and why they were included	Included unadjusted *Table 4, S2 Table, S3 Table*, and *S4 Table;* adjusted *S5 Table* and *S6 Table*
		(*b*) Report category boundaries when continuous variables were categorized	Included *Table 4, S2 Table, S3 Table*, and *S4 Table*
		(*c*) If relevant, consider translating estimates of relative risk into absolute risk for a meaningful time period	Included MPoRT 5-year predicted risk

Other analyses	17	Report other analyses done—eg analyses of subgroups and interactions, and sensitivity analyses	Included *S5 Table* and *S6 Table*
**Discussion**			
Key results	18	Summarise key results with reference to study objectives	Included paragraphs 1-2
Limitations	19	Discuss limitations of the study, taking into account sources of potential bias or imprecision. Discuss both direction and magnitude of any potential bias	Included *Limitations*
Interpretation	20	Give a cautious overall interpretation of results considering objectives, limitations, multiplicity of analyses, results from similar studies, and other relevant evidence	Included *Conclusions*
Generalisability	21	Discuss the generalisability (external validity) of the study results	Included *Comparison with existing approaches – Hazard ratios*
**Other information**			
Funding	22	Give the source of funding and the role of the funders for the present study and, if applicable, for the original study on which the present article is based	Included *Acknowledgements*
